# Supplementary material for: Biosynthesis of the highly oxygenated tetracyclic core skeleton of Taxol
Source: Nat Commun. 2024 Mar 15;15:2339. doi: 10.1038/s41467-024-46583-3 (PMC10942993; doi:10.1038/s41467-024-46583-3)
Supplement: Supplementary file 1 — Supplementary Information [file 41467_2024_46583_MOESM1_ESM.pdf]

Biosynthesis of the highly oxygenated tetracyclic core skeleton of Taxol

Yang *et al.*

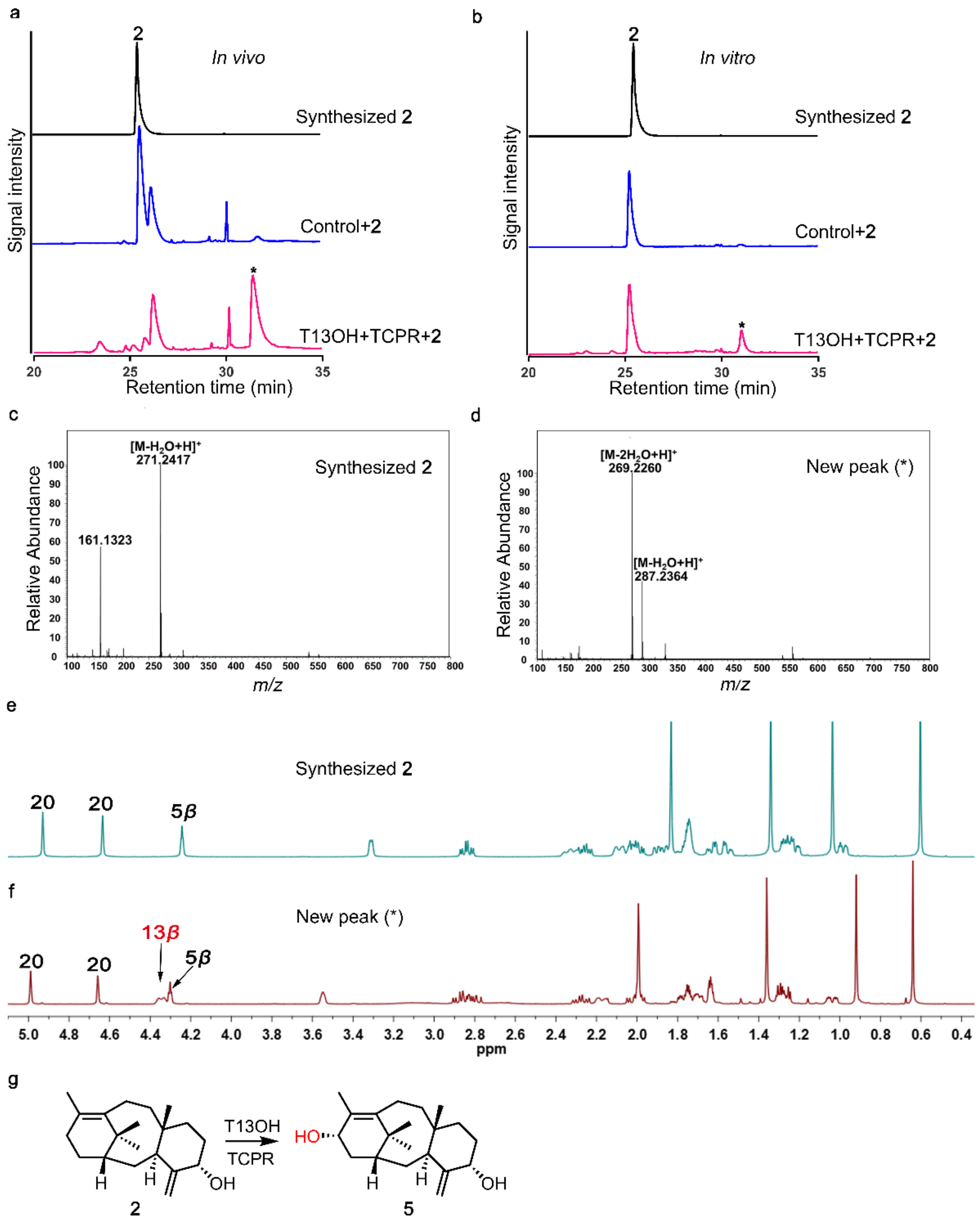

Supplementary Figure 1. The bioconversion of taxa-4(20),11(12)-diene-5α-ol (2) to taxa-4(20),11(12)-diene-5α,13α-diol (5) *via* T13OH. a-b, Gas chromatography flame ionization detector (GC-FID) analysis of *in vivo* and *in vitro* reactions by *S. cerevisiae* strain YT13OH expressing T13OH and TCPR using **2** as the substrate. Substrate was marked with compound number and product was marked with an asterisk. *S. cerevisiae* chassis strain YBD80 was used as the control for both *in vivo* and *in vitro* reactions. c, Mass spectra (Electro spray ionization, ESI) of synthesized **2** . d, Mass spectra (ESI) of the new peak (*). These above results were confirmed three times with similar results each time. e, ^{1}H- nuclear magnetic resonance (NMR) spectrum of synthesized **2.** f, ^{1}H-NMR spectrum of new peak (*).g, C13-hydroxylation reaction of **2** catalyzed by T13OH. T13OH, taxoid 13α-hydroxylase; TCPR, *Taxus* cytochrome P450 reductase. New formed hydroxyl group in compound and the change of peaks in NMR spectrum were marked with red. Source data are provided as a Source Data file.

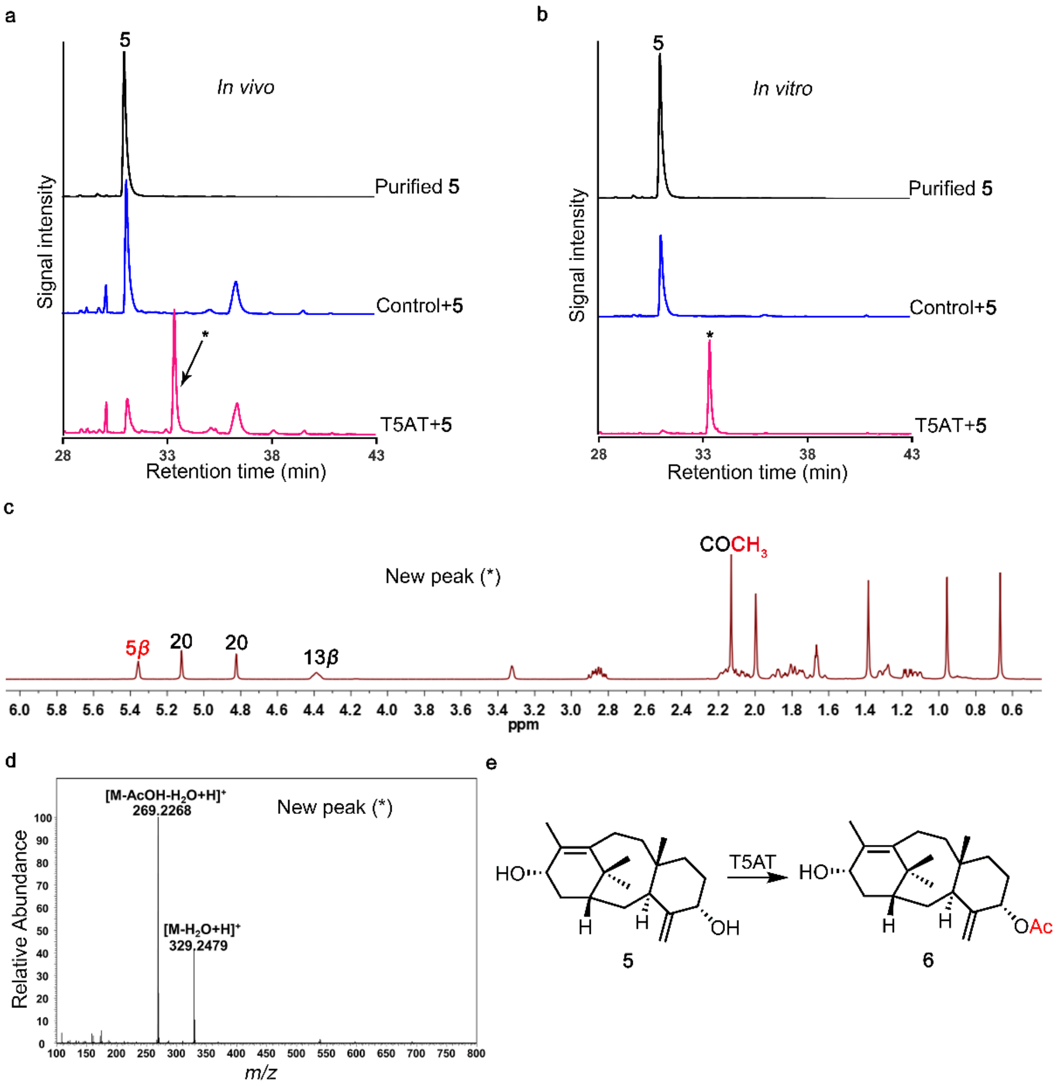

Supplementary Figure 2. The bioconversion of taxa-4(20),11(12)-diene-5α,13α-diol (5) to 5α-acetoxytaxa-4(20),11(12)-diene-13α-ol (6) *via* T5AT. a-b, GC-FID analysis of *in vivo* and *in vitro* reactions by *S. cerevisiae* strain YT5AT expressing T5AT using **5** as the substrate. Substrate was marked with compound number and product was marked with an asterisk. *S. cerevisiae* chassis strain YBD80 was used as the control for both *in vivo* and *in vitro* reactions. These above results were confirmed three times with similar results each time. c, ^{1}H-NMR spectrum of new peak (*). d, Mass spectra (ESI) of new peak (*). Mass spectra (ESI) was confirmed in three independent experiments**.** e, Acetylation reaction of 5*α*-hydroxyl in **5** catalyzed by T5AT. T5AT, taxoid 5-O-acetyltransferase. New formed group in compound and the change of peaks in NMR spectrum were marked with red. Source data are provided as a Source Data file.

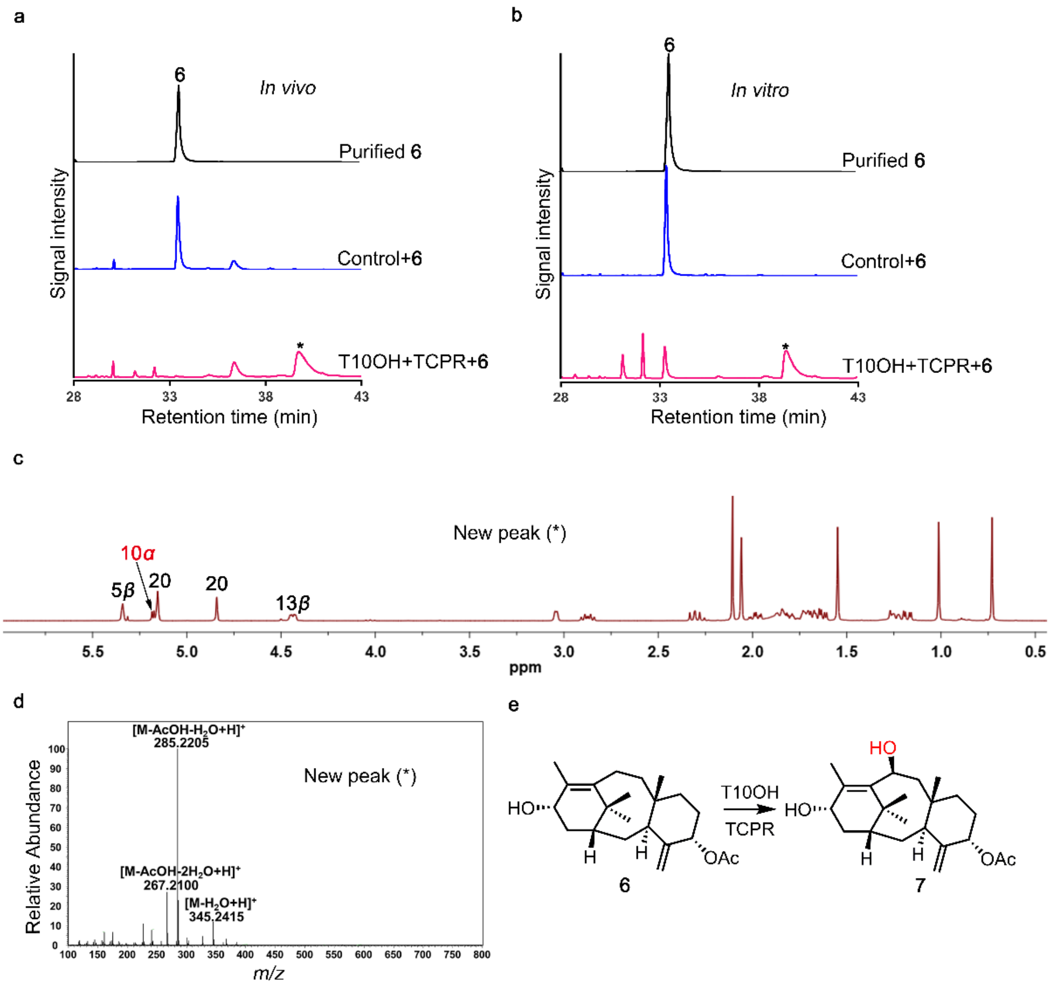

Supplementary Figure 3. The bioconversion of 5*α*-acetoxytaxa-4(20),11(12)-diene-13*α*-ol (6) to 5*α*-acetoxytaxa-4(20),11(12)-diene-10*β*,13*α*-diol (7) *via* T10OH. a-b, GC-FID analysis of *in vivo* and *in vitro* reactions for *S. cerevisiae* strain YT10OH expressing T10OH and TCPR using **6** as the substrate. Substrate was marked with compound number and product was marked with an asterisk. *S. cerevisiae* chassis strain YBD80 was used as the control for both *in vivo* and *in vitro* reactions. These above results were confirmed three times with similar results each time. c, ^{1}H-NMR spectrum of new peak (*). d, Mass spectra (ESI) of new peak (*). Mass spectra (ESI) was confirmed in three independent experiments**.** e, C10-hydroxylation reaction of **6** catalyzed by T10OH. T10OH, taxoid 10*β*-hydroxylase. New formed group in compound and the change of peaks in NMR spectrum were marked with red. Source data are provided as a Source Data file.

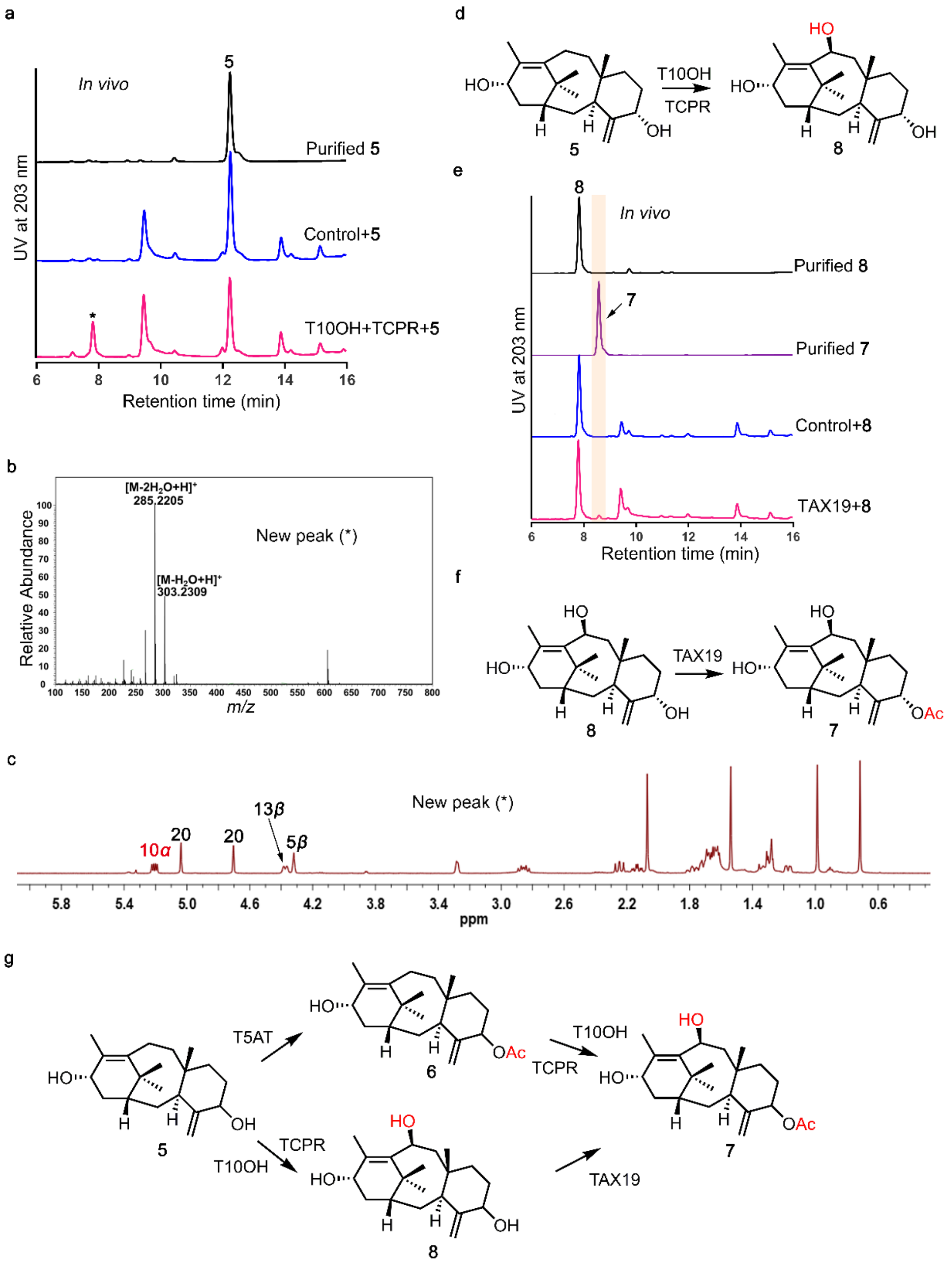

Supplementary Figure 4. The bioconversions of taxa-4(20),11(12)-diene-5α,13α-diol (5) to taxa-4(20),11(12)-diene-5*α*,10*β*,13*α*-triol (8) *via* T10OH and 8 to 5*α*-acetoxytaxa-4(20),11(12)-diene-10*β*,13*α*-diol (7) *via* TAX19. a, HPLC analysis of *in vivo* reaction for *S. cerevisiae* strain YT10OH expressing T10OH and TCPR using **5** as the substrate. *S. cerevisiae* chassis strain YBD80 was used as the control for *in vivo* reaction. Substrate was marked with compound number and product was marked with an asterisk. b, Mass spectra (ESI) of new peak (*). These above results were confirmed three times, with similar results each time. c. ^{1}H-NMR spectrum of new peak (*). d, C10-hydroxylation reaction of **5** catalyzed by T10OH. e, HPLC analysis of *in vivo* reaction for *S.*

cerevisiae strains YTAX19 expressing TAX19 using **8** as the substrate. Substrate was marked with compound number. *S. cerevisiae* chassis strain YBD80 was used as the control for *in vivo* reaction. This result was confirmed three times, with similar results each time. f, Acetylation reaction of 5*α*-hydroxyl in **8** catalyzed by TAX19. g, Two biosynthetic routes towards **7** starting from **5**. TAX19, taxoid 5-O-acetyltransferase. New formed groups in compounds and the change of peaks in NMR spectrum were marked with red. Source data are provided as a Source Data file.

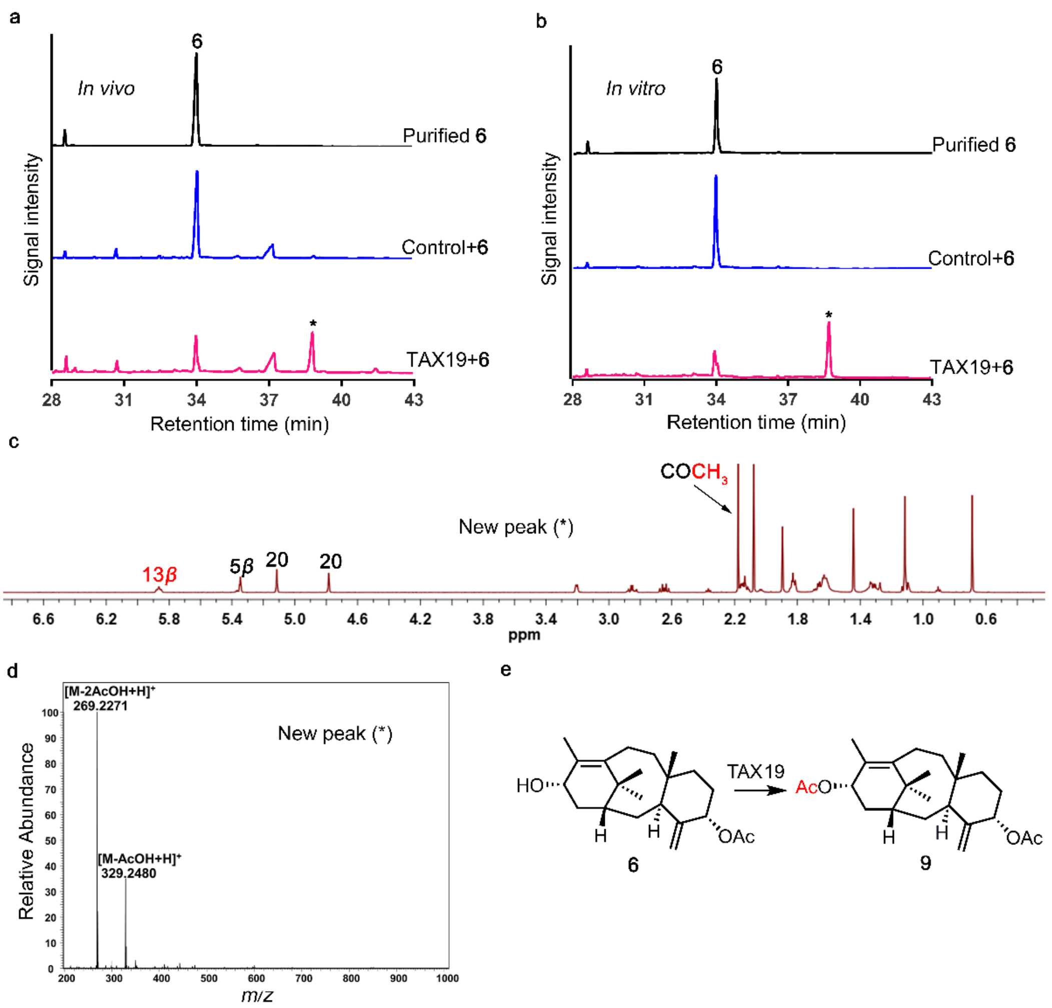

Supplementary Figure 5. The bioconversion of 5α-acetoxytaxa-4(20),11(12)-diene-13α-ol (6) to 5α,13α-diacetoxytaxa-4(20),11(12)-diene (9) *via* TAX19. a-b, GC-FID analysis of *in vivo* and *in vitro* reactions for *S. cerevisiae* strain YTAX19 expressing TAX19 using **6** as the substrate. Substrate was marked with compound number and product was marked with an asterisk. *S. cerevisiae* chassis strain YBD80 was used as the control for both *in vivo* and *in vitro* reactions. These above results were confirmed three times with similar results each time. c, ^{1}H-NMR spectrum of new peak (*). d, Mass spectra (ESI) of new peak (*). Mass spectra (ESI) was confirmed in three independent experiments**.** e, Acetylation of 13α-hydroxyl in **6** catalyzed by TAX19. New formed group in compound and the change of peaks in NMR spectrum were marked with red. Source data are provided as a Source Data file.

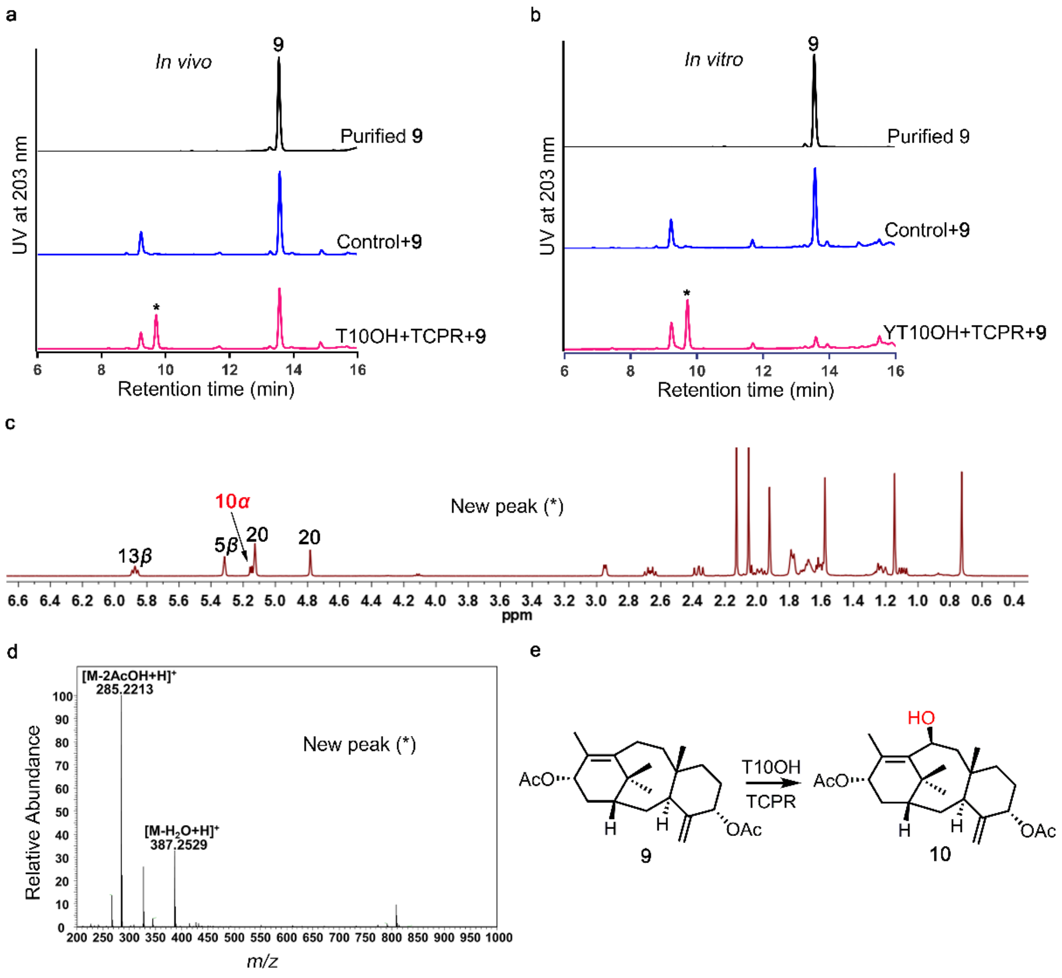

Supplementary Figure 6. The bioconversion of the di-oxygenated taxoid 5*α*,13*α*-diacetoxytaxa-4(20),11(12)-diene (9) to the tri-oxygenated taxoid 5*α*,13*α*-diacetoxytaxa-4(20),11(12)-diene-10*β*-ol (10) *via* T10OH. a-b, HPLC analysis of *in vivo* and *in vitro* reactions for *S. cerevisiae* strain YT10OH expressing T10OH and TCPR using **9** as the substrate. Substrate was marked with compound number and product was marked with an asterisk. *S. cerevisiae* chassis strain YBD80 was used as the control for both *in vivo* and *in vitro* reactions. These above results were confirmed three times, with similar results each time. c, ^{1}H-NMR spectrum of new peak (*). d, Mass spectra (ESI) of new peak (*) . Mass spectra (ESI) was confirmed in three independent experiments**.** e, C10-hydroxylation reaction of **9** catalyzed by T10OH. New formed group in compound and the change of peaks in NMR spectrum were marked with red. Source data are provided as a Source Data file.

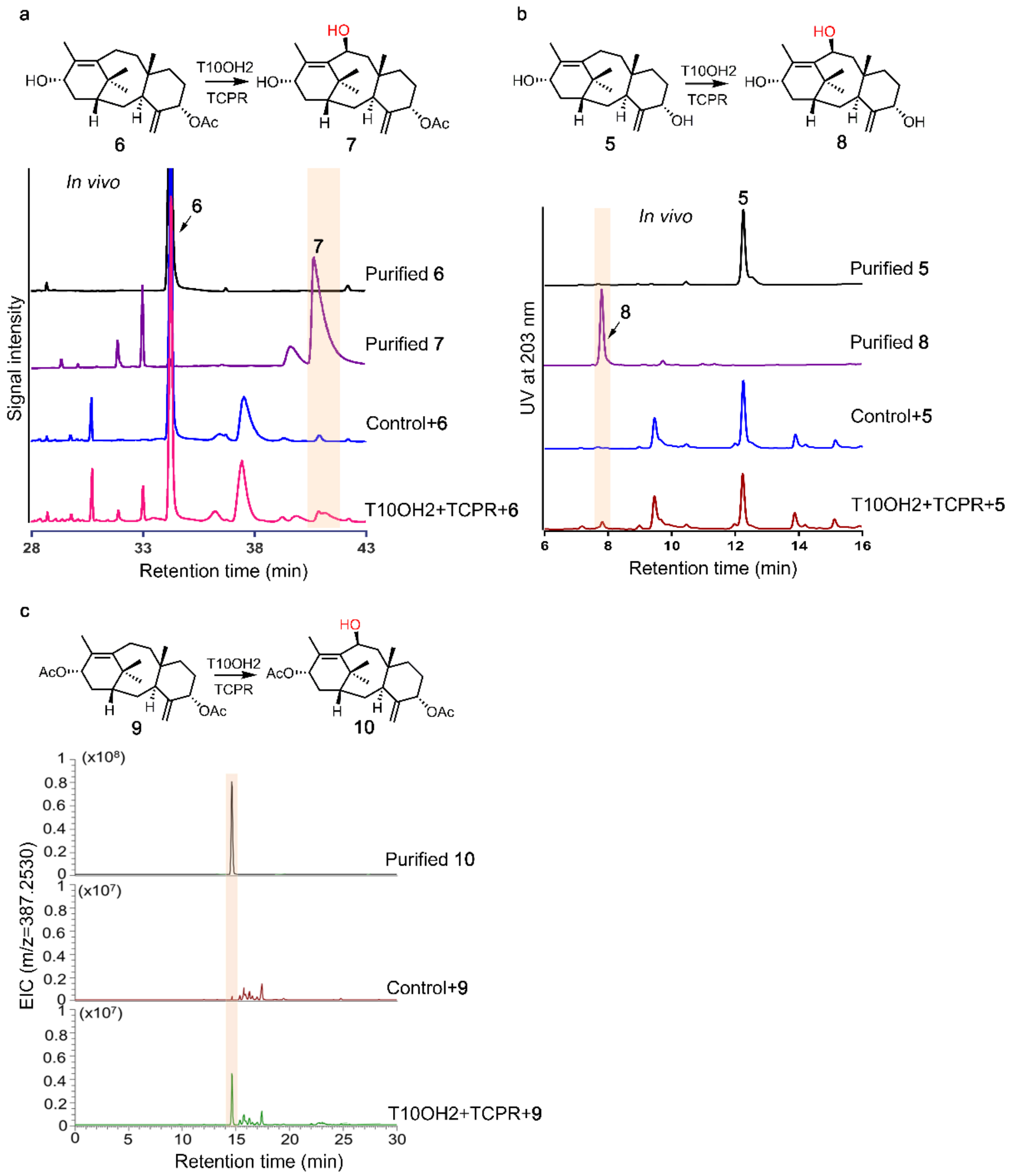

Supplementary Figure 7. Characterization of T10OH2. a, GC-FID analysis of *in vivo* reaction for *S. cerevisiae* strain YT10OH2 expressing T10OH2 and TCPR using **6** as the substrate. b, HPLC analysis of *in vivo* reaction for *S. cerevisiae* strain YT10OH2 expressing T10OH2 and TCPR using **5** as the substrate. c, LC-MS analysis of *in vivo* reaction for *S. cerevisiae* strain YT10OH2 expressing T10OH2 and TCPR using **9** as the substrate. *S. cerevisiae* strain YTCPR expressing TCPR was used as the control. These above results were confirmed three times, with similar results each time. New formed hydroxyl groups in compounds were marked with red. Source data are provided as a Source Data file.

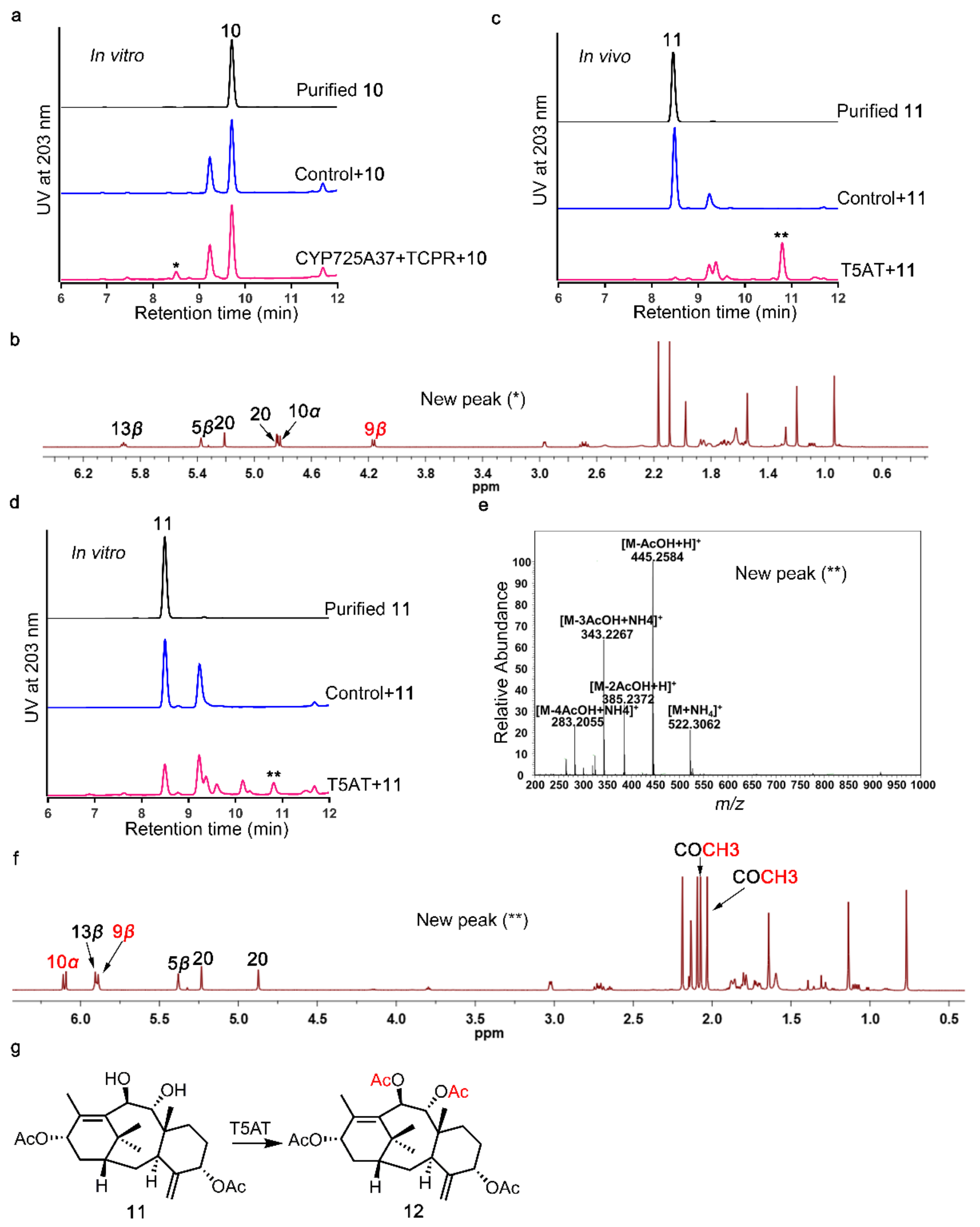

Supplementary Figure 8. Discovery and characterization of taxoid C9α hydroxylase and the bioconversion of 9,10-deacetyl taxusin (11) to taxusin (12) *via* T5AT. a, HPLC analysis of *in vitro* reaction for *S. cerevisiae* strain YCYP725A37 expressing CYP725A37 and TCPR using **10** as the substrate. Substrate was marked with compound number and product was marked with an asterisk. *S. cerevisiae* strain YTCPR expressing TCPR was used as the control. b, ^{1}H-NMR spectrum of new peak (*). See Supplementary Information for a detailed analysis of NMR results. c-d, HPLC analysis of *in vivo* and *in vitro* reactions for *S. cerevisiae* strain YT5AT expressing T5AT using **11** as the substrate. Substrate was marked with compound number and product was marked with two asterisks. *S. cerevisiae* chassis strain YBD80 was used as the control for both *in vivo* and *in vitro* reactions. e, Mass spectra (ESI) of new peak (**). These above HPLC and Mass spectra (ESI) results were confirmed three times with similar results each time. f, ^{1}H-NMR spectrum of new peak (**). g,

Acetylation reaction catalyzed by T5AT with **11** as the substrate based on MS and NMR analyses. New formed groups in compound and the change of peaks in NMR spectrum were marked with red. Source data are provided as a Source Data file.

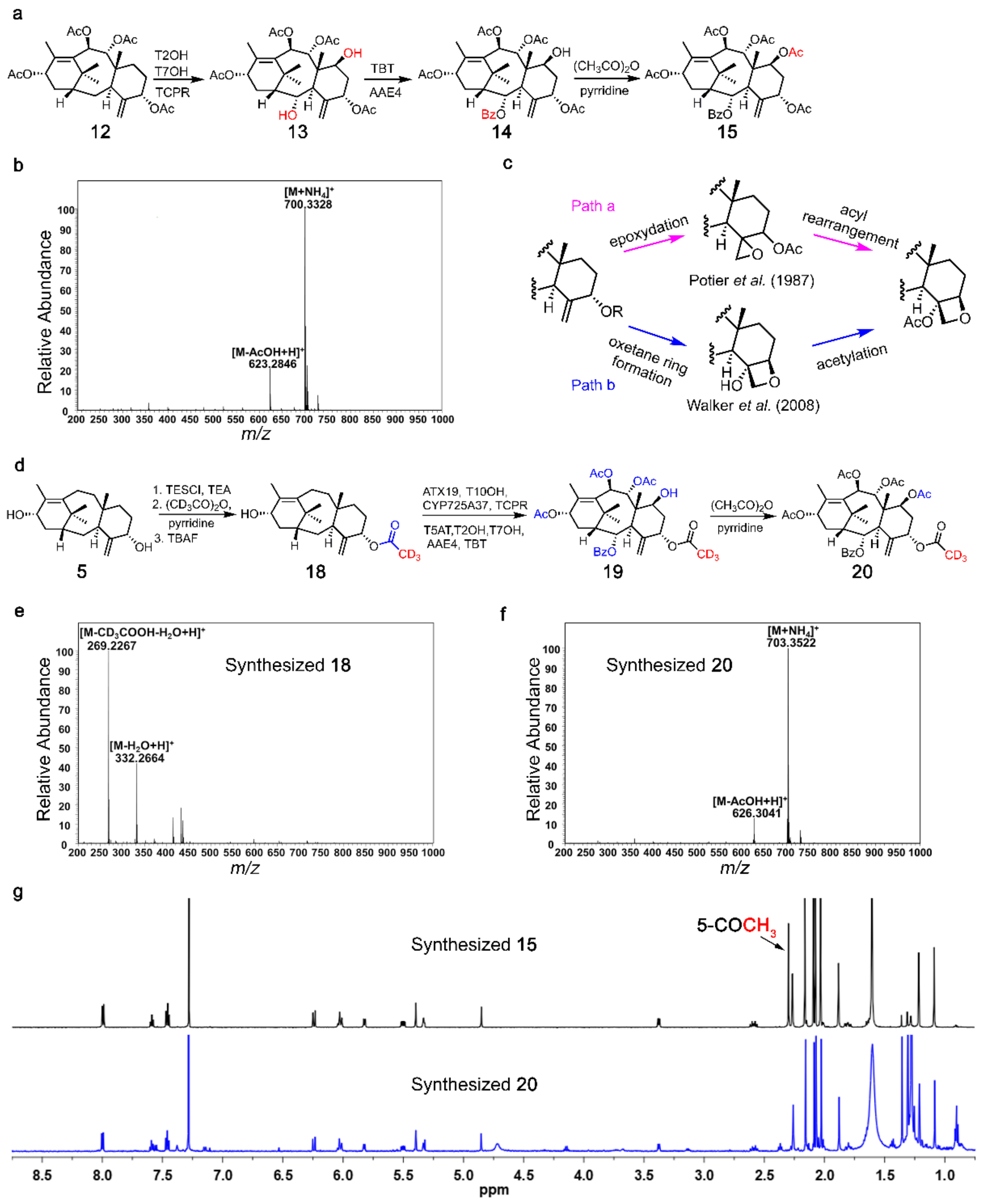

Supplementary Figure 9. CYP725A55 catalyzed the formation of oxetane ester. a, The route for the preparation of 2α-benzoyloxy-7β-acetoxytaxusin (**15**). AAE4 catalyzing benzoic acid to produce benzoyl-CoA was used to biosynthesize benzoyl-CoA for the preparation of **15**. New formed groups in compounds were marked with red. b, The MS chromatogram of synthesized **15**. c, Summary of the literature proposed biosynthetic routes for the formation of oxetane ester. d, The route for the preparation of 5α-trideuterated acetyl-2α-benzoyloxy-7β-acetoxy taxusin (**20**). New formed groups in compounds were marked with blue. ^{2}H labelled group was marked with red. e, Mass spectra (ESI) of synthesized 5α-trideuterated acetoxytaxa-4(20),11(12)-diene (**18**). f, Mass spectra (ESI) of synthesized **20**. g, Comparison of ^{1}H-NMR spectrum of **15** (up) with that of **20** (down). The change of peak in NMR spectrum was marked with red. T2OH, taxoid 2α-hydroxylase; T7OH, taxoid 7β-hydroxylase; TBT, taxoid 2α-O-benzoyltransferase.

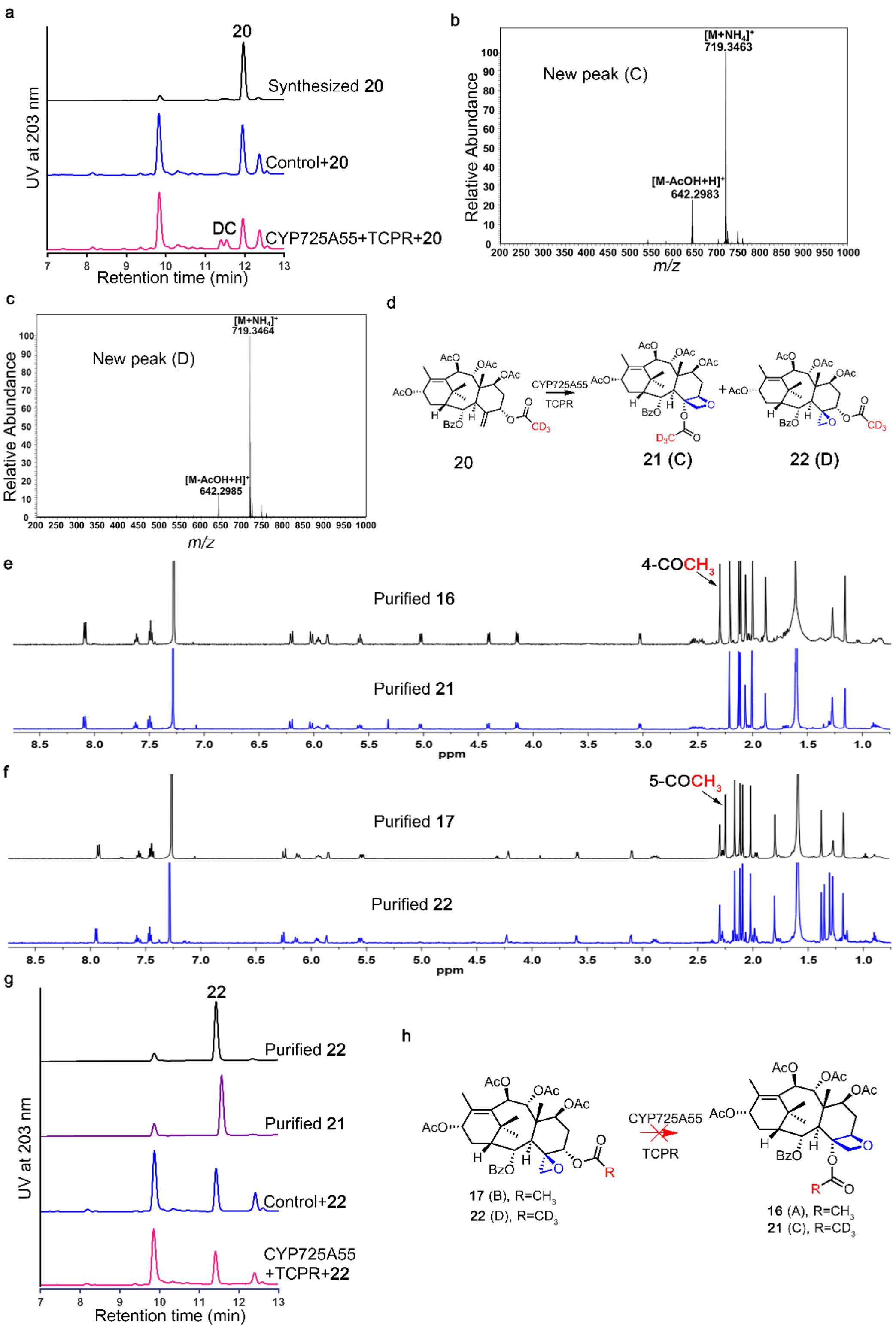

Supplementary Figure 10. Research on the source of C4-acetoxyl group. a, HPLC analysis of *in vivo* reaction for *S. cerevisiae* strain YCYP725A55 expressing CYP725A55 and TCPR using 5α-trideuterated acetyl-2α-benzoyloxy-7β-acetoxy taxusin (**20**) as the substrate. Substrate was marked with compound number and product was marked with C and D.*S. cerevisiae* strain YTCPR expressing TCPR was used as the control. b, Mass spectra (ESI) of new peak (C). c, Mass spectra

(ESI) of new peak (D). These above HPLC and MS results were confirmed three times, with similar results each time. d, Oxidation reaction catalyzed by CYP725A55 with **20** as the substrate. New formed groups in compound were marked with blue. ^{2}H labelled group was marked with red. e, Comparison of ^{1}H-NMR spectrum of 1β-dehydroxybaccatin VI (**16**) (up) with that of 1β-dehydroxy-4α-trideuterated acetyl-baccatin VI (**21**) (down). f, Comparison of ^{1}H-NMR spectrum of 2-deacetyl-2α-benzoylbaccatin I (**17**) (up) with that of 5α-trideuterated acetyl-2-deacetyl-2-benzoylbaccatin I (**22**) (down). g. HPLC analysis of *in vivo* reaction for *S. cerevisiae* strain YCYP725A55 expressing CYP725A55 and TCPR using **22** as the substrate. Substrate was marked with compound number. *S. cerevisiae* strain YTCPR expressing TCPR was used as the control. h, Proposed reaction catalyzed by CYP725A55 with **17** or **22** as the substrate. New formed groups in compound were marked with blue. Labeled or unlabeled methyl was marked with red. Arrow with a cross indicated this reaction was failed. Source data are provided as a Source Data file.

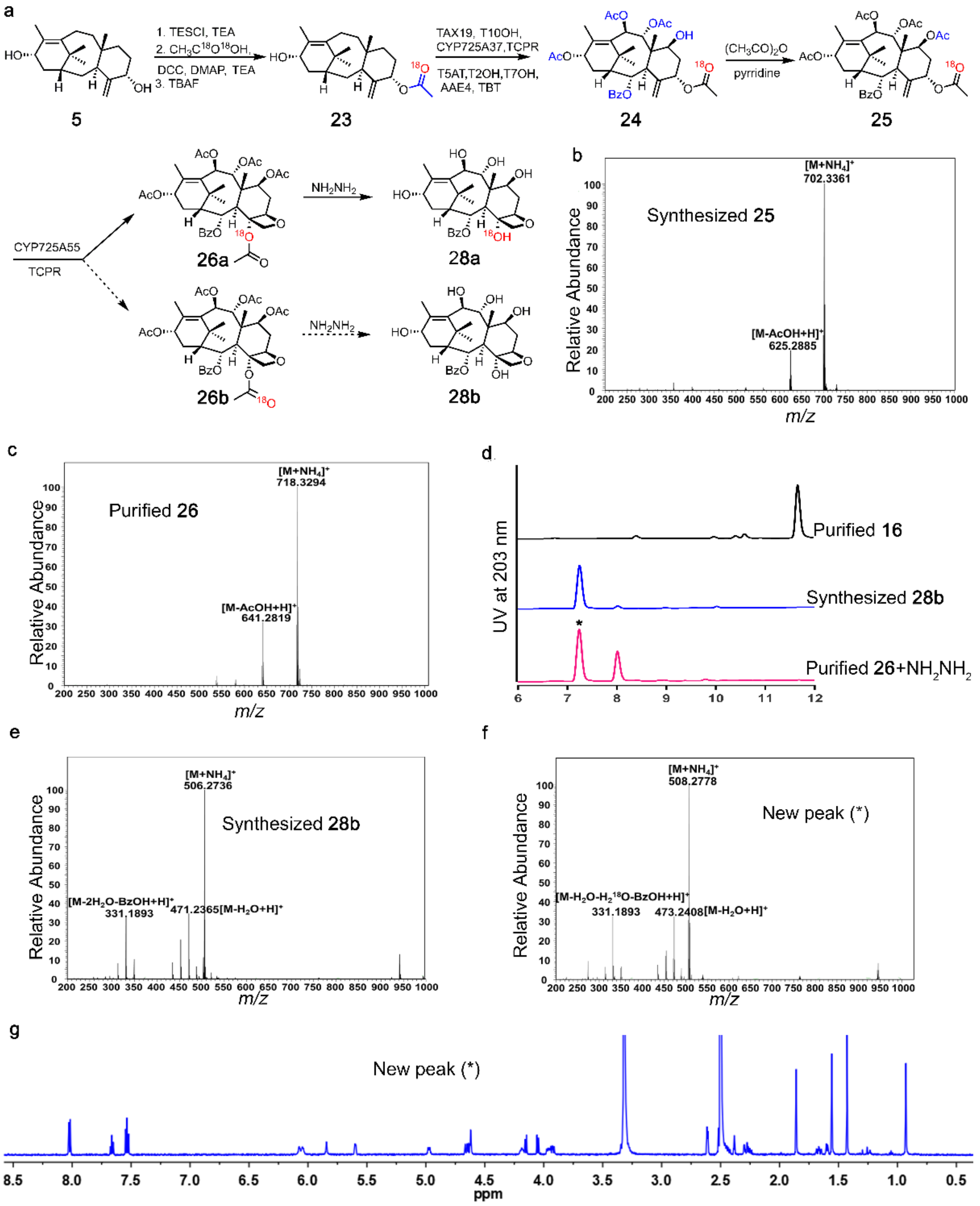

Supplementary Figure 11. Research on the acyl rearrangement process. a, The route for the verification of acyl rearrangement process. AAE4 catalyzing benzoic acid to produce benzoyl-CoA was used to biosynthesize benzoyl-CoA and NH_2NH_2 was used to remove the acetyl groups. New formed groups in compound were marked with blue. ^{18}O labelled group was marked with red. b, Mass spectra (ESI) of synthesized O^{18}-labelled 2*α*-benzoyloxy-7*β*-acetoxytaxusin (**25**). c, Mass spectra (ESI) of purified **26** (**26a** or the mixture of **26a** and **26b**). d. HPLC analysis of deacetylation experiment of the ^{18}O labeled oxetane ester product of CYP725A55 using **25** as the substrate. Regular **16** and 4,7,9,10,13-pentadeacetyl-1*β*-dehydroxybaccatin VI (**28b**) were used as standards. Product was marked with an asterisk. e, Mass spectra (ESI) of synthesized regular **28b**. f, Mass spectra (ESI) of purified new peak(*). g, ^1H-NMR spectrum of new peak (*). Source data are provided as a Source Data file.

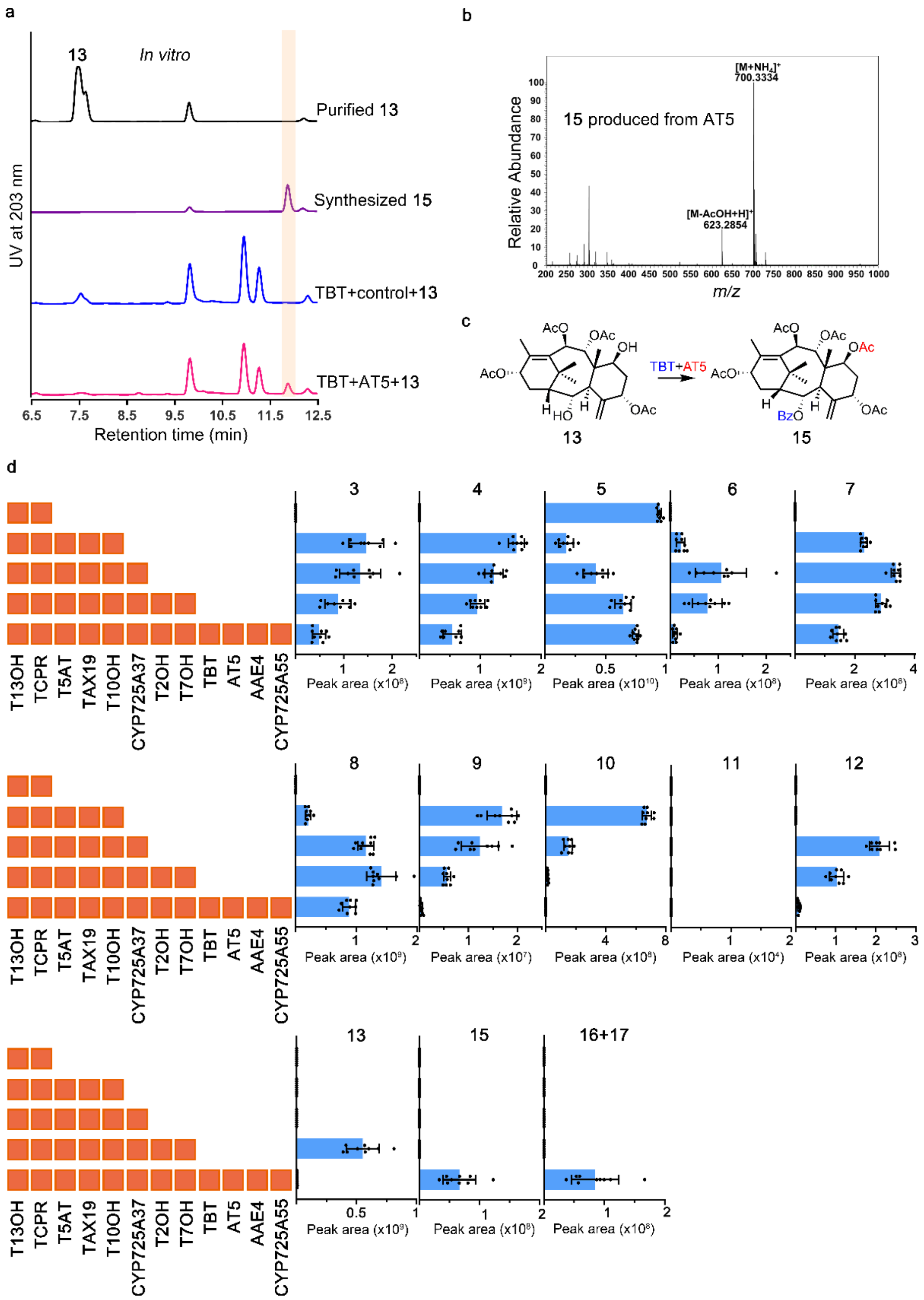

Supplementary Figure 12. Biosynthesis of 1*β*-dehydroxybaccatin VI (16) from 2. a, HPLC analysis of *in vitro* enzyme assay for AT5 combined with TBT using **13** as the substrate. Crude protein lysate from *E.coli* strain expressing empty vector was used as the control. Substrate was marked with compound number and product was marked with yellow box. b, Mass spectra (ESI) of **15** produced from AT5. These above results were confirmed three times, with similar results each time. c, Acylation reaction catalyzed by AT5 and TBT with **13** as the substrate. New formed benzoyl group in compound and corresponding enzyme were marked with blue. New formed acetyl group

in compound and corresponding enzyme were marked with red. d, Integrated peak area of EIC for intermediates produced in *S. cerevisiae* strains with co-expression of indicated enzymes and feeding of **2**. Orange block diagram indicated the expression of corresponding enzyme. Date are mean ± standard deviation; n = 9 independent examples. *S. cerevisiae* chassis strain YBD80 was used as the control. Source data are provided as a Source Data file.

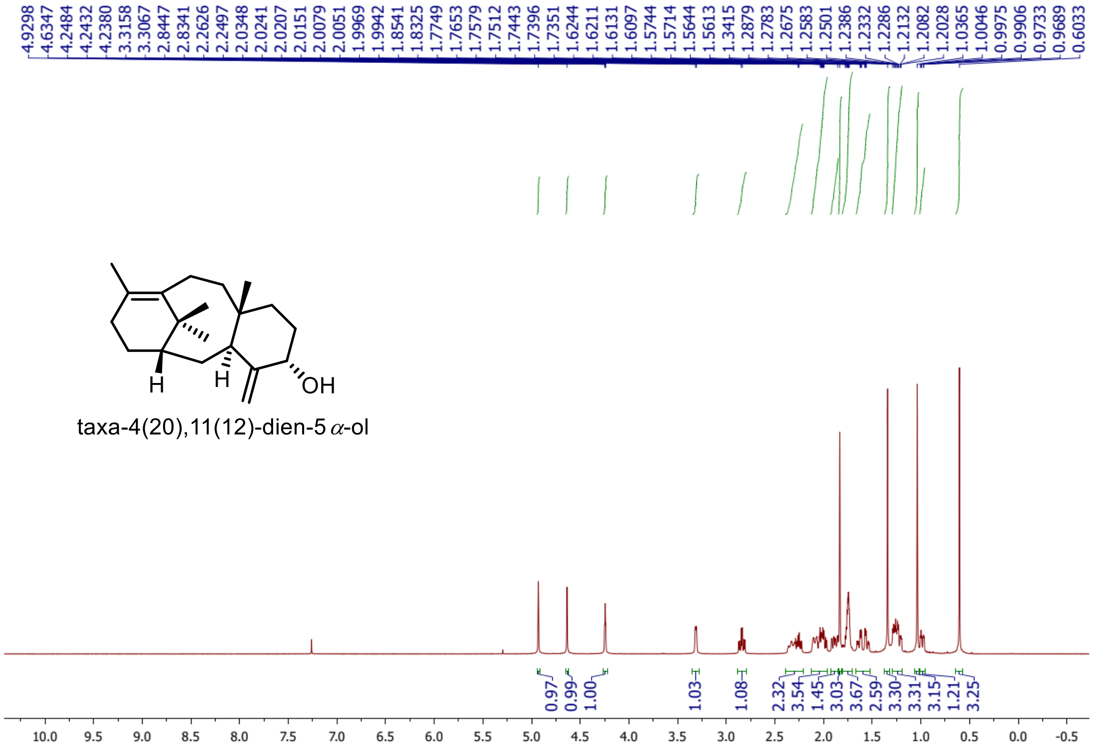

Supplementary Figure 13. **^{1}H NMR spectrum of taxa-4(20),11(12)-diene-5*α*-ol (2).**

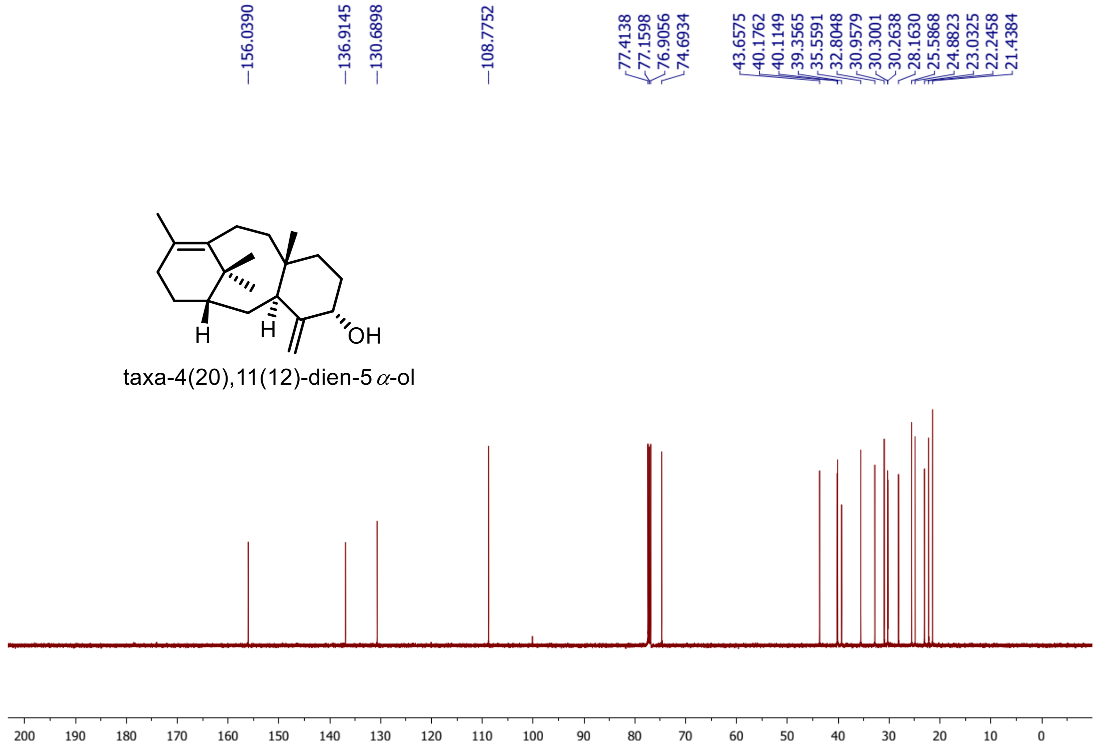

Supplementary Figure 14. ^{13}C NMR spectrum of taxa-4(20),11(12)-diene-5*α*-ol (2).

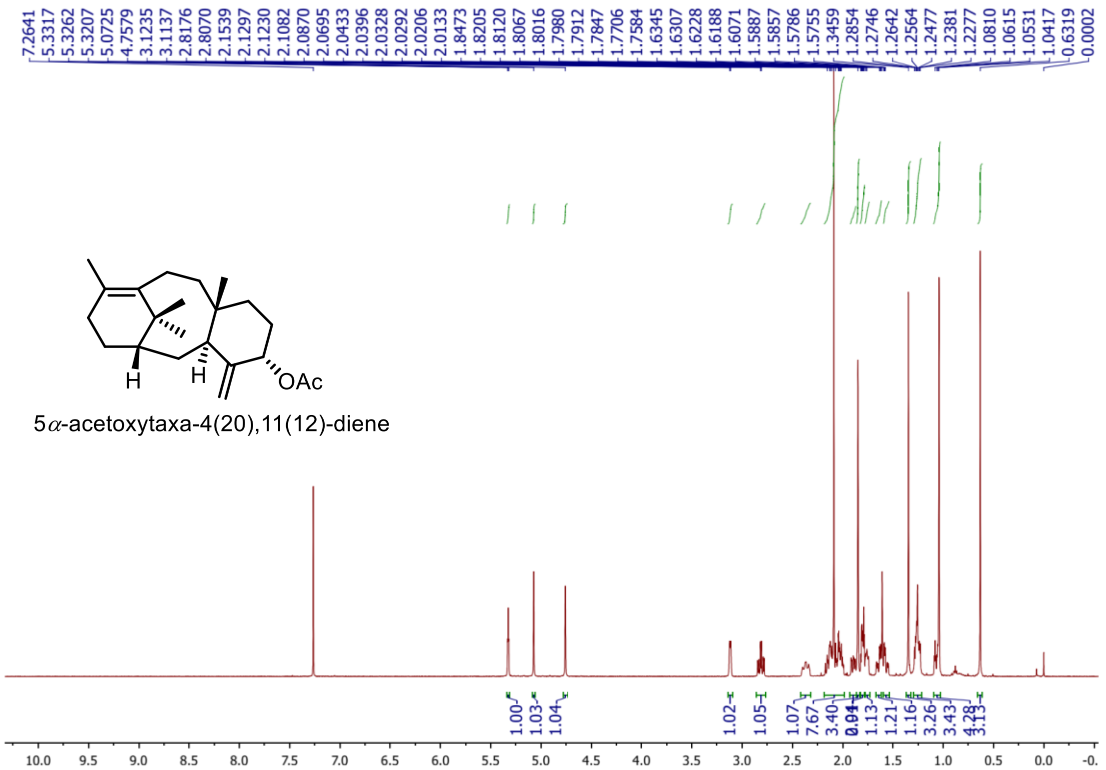

Supplementary Figure 15. ^{1}**H NMR spectrum of 5*α*-acetoxytaxa-4(20),11(12)-diene (3).**

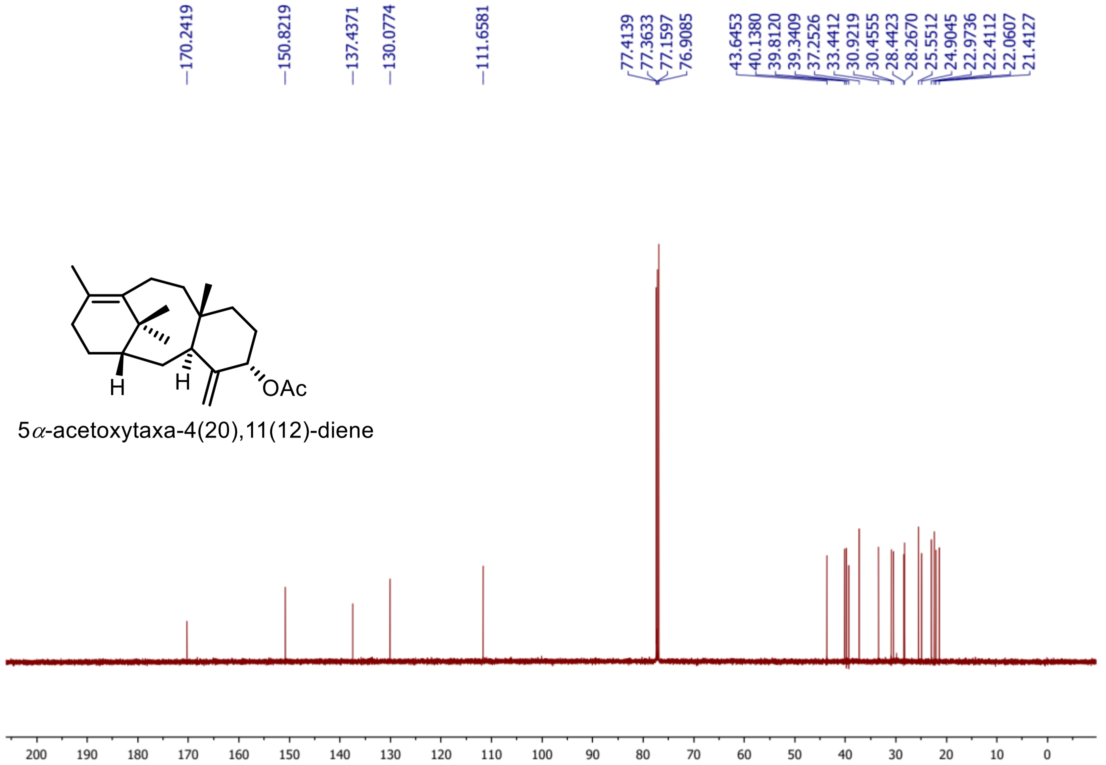

Supplementary Figure 16. ^{13}**C NMR spectrum of 5*α*-acetoxytaxa-4(20),11(12)-diene (3).**

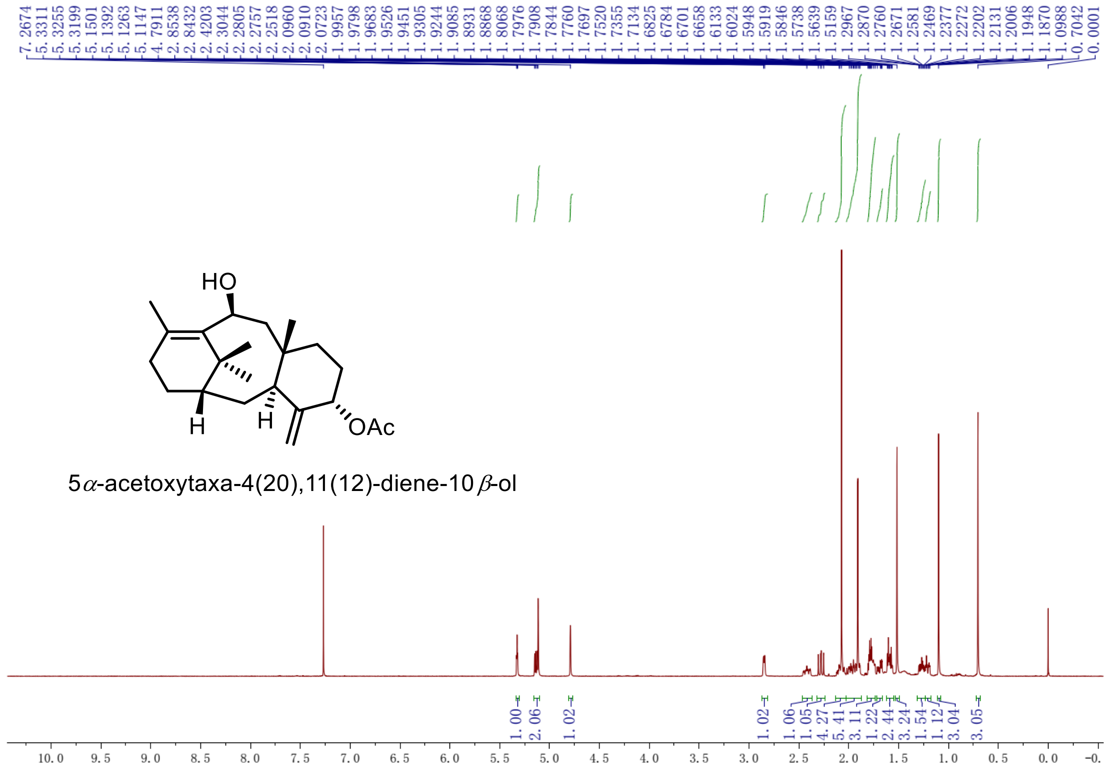

Supplementary Figure 17. **^{1}H NMR spectrum of 5*α*-acetoxytaxa-4(20), 11(12)-diene-10*β*-ol (4).**

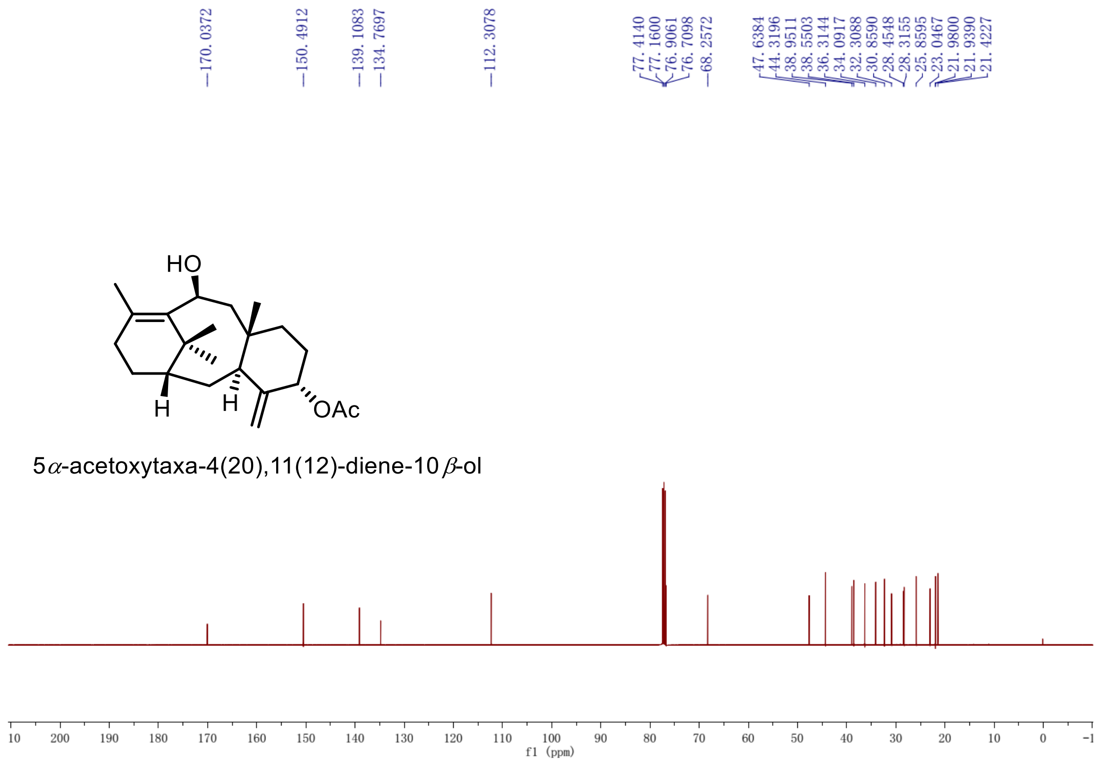

Supplementary Figure 18. **^{13}C NMR spectrum of 5*α*-acetoxytaxa-4(20), 11(12)-diene-10*β*-ol (4).**

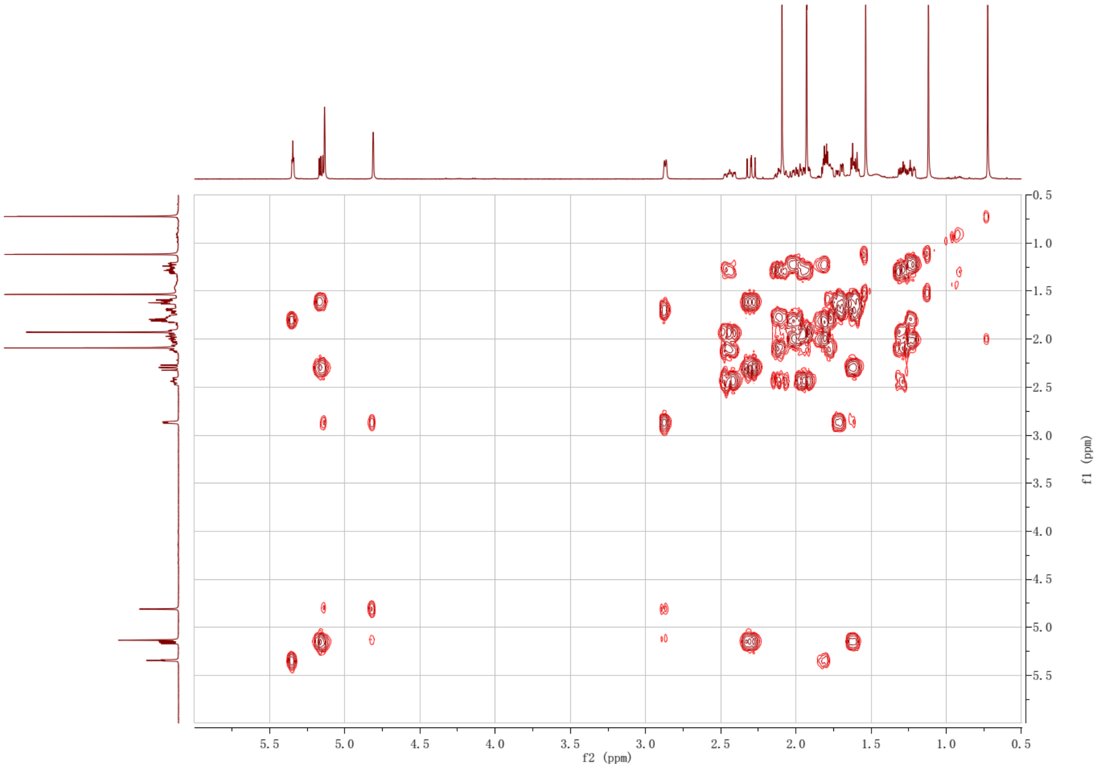

Supplementary Figure 19. 2D-COSY NMR spactrum of 5*α*-acetoxytaxa-4(20), 11(12)-diene-10*β*-ol (4).

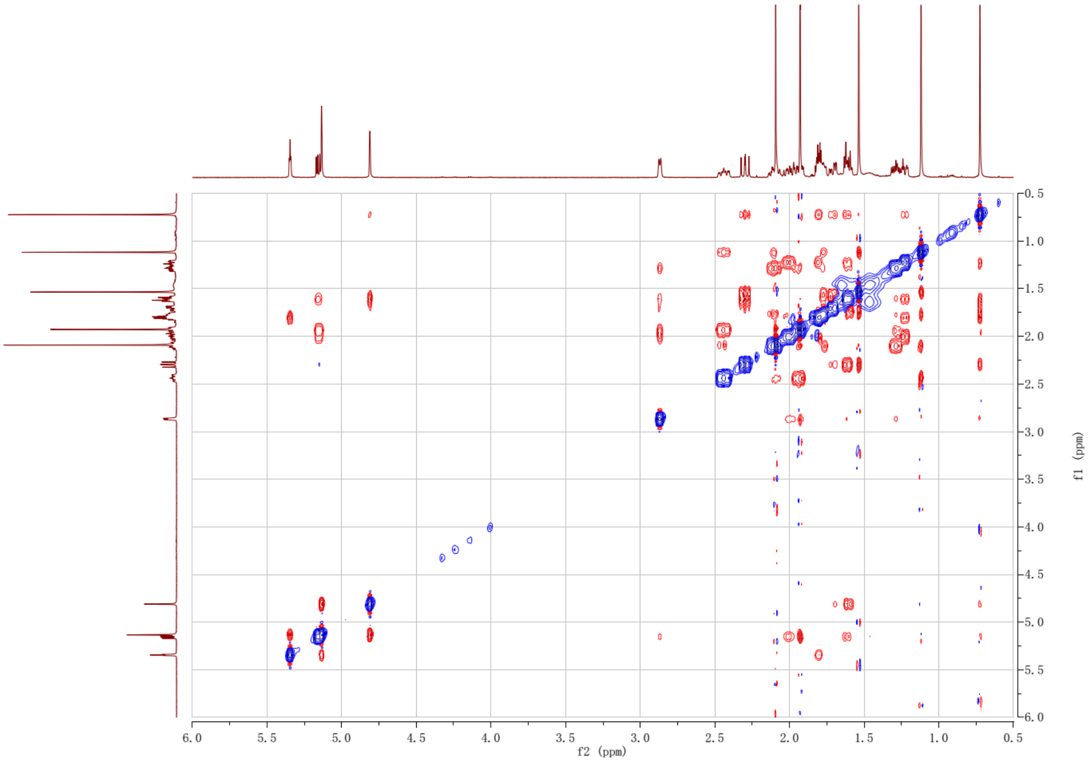

Supplementary Figure 20. 2D-NOE NMR spactrum of 5*α*-acetoxytaxa-4(20), 11(12)-diene-10*β*-ol (4).

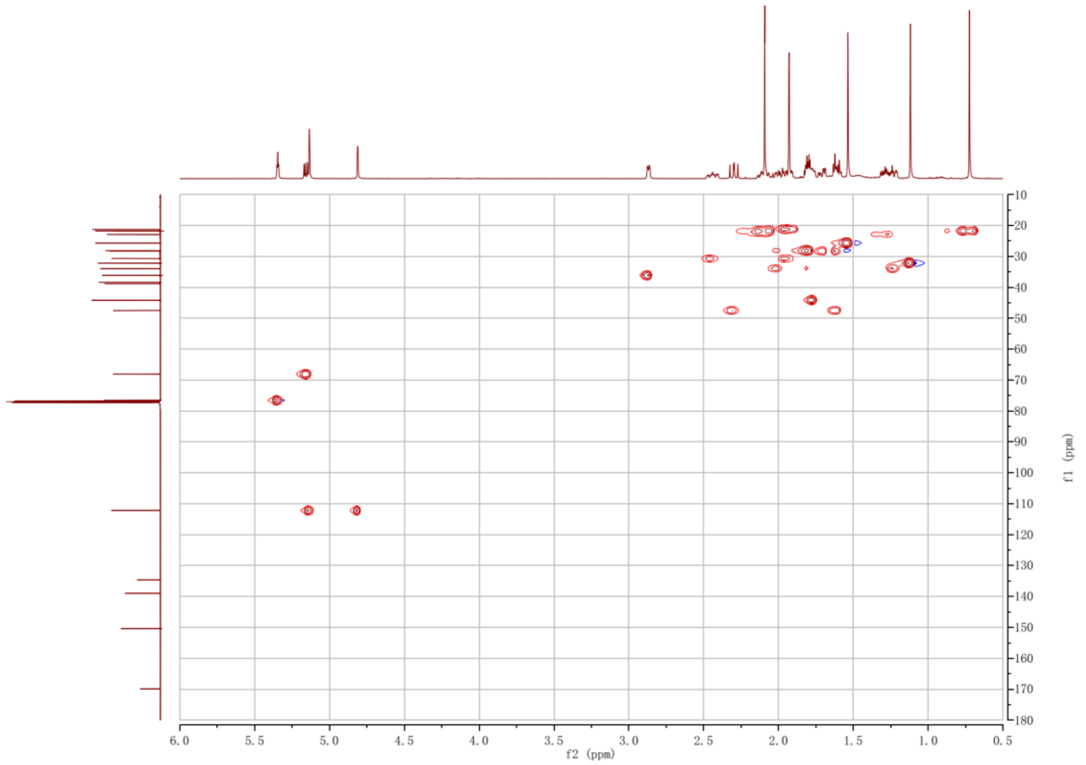

Supplementary Figure 21. 2D-HSQC NMR spactrum of 5*α*-acetoxytaxa-4(20), 11(12)-diene-10*β*-ol (4).

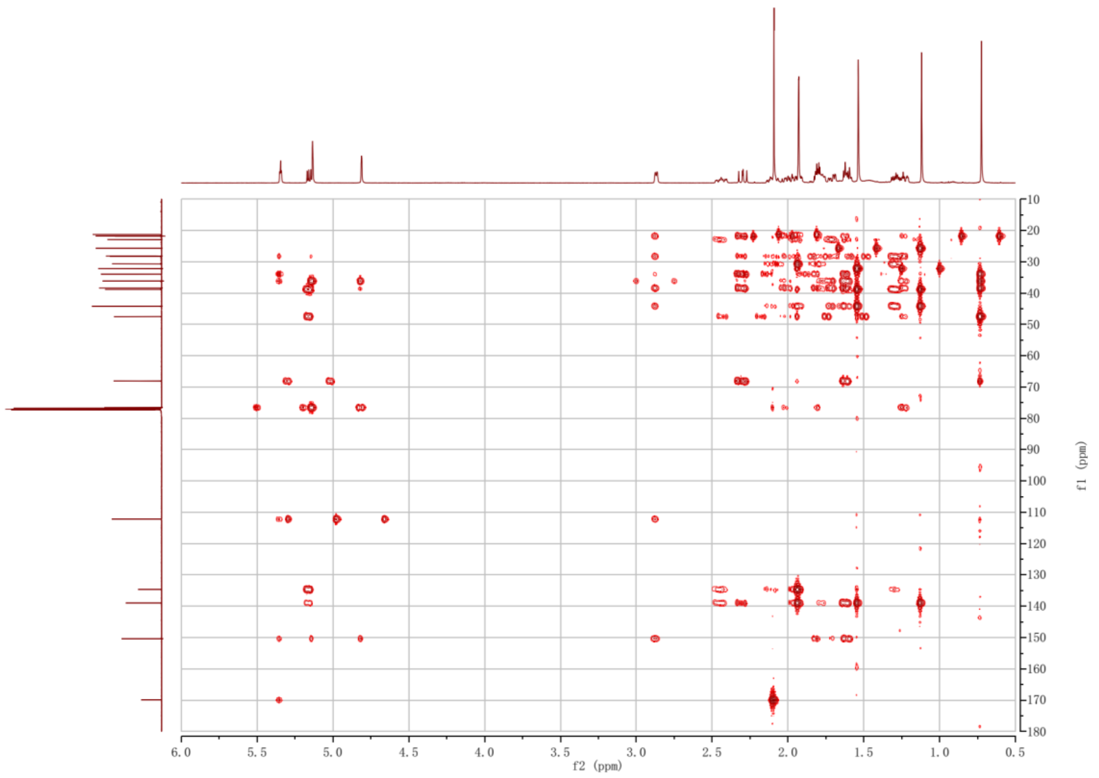

Supplementary Figure 22. 2D-HMBC NMR spactrum of 5*α*-acetoxytaxa-4(20), 11(12)-diene-10*β*-ol (4).

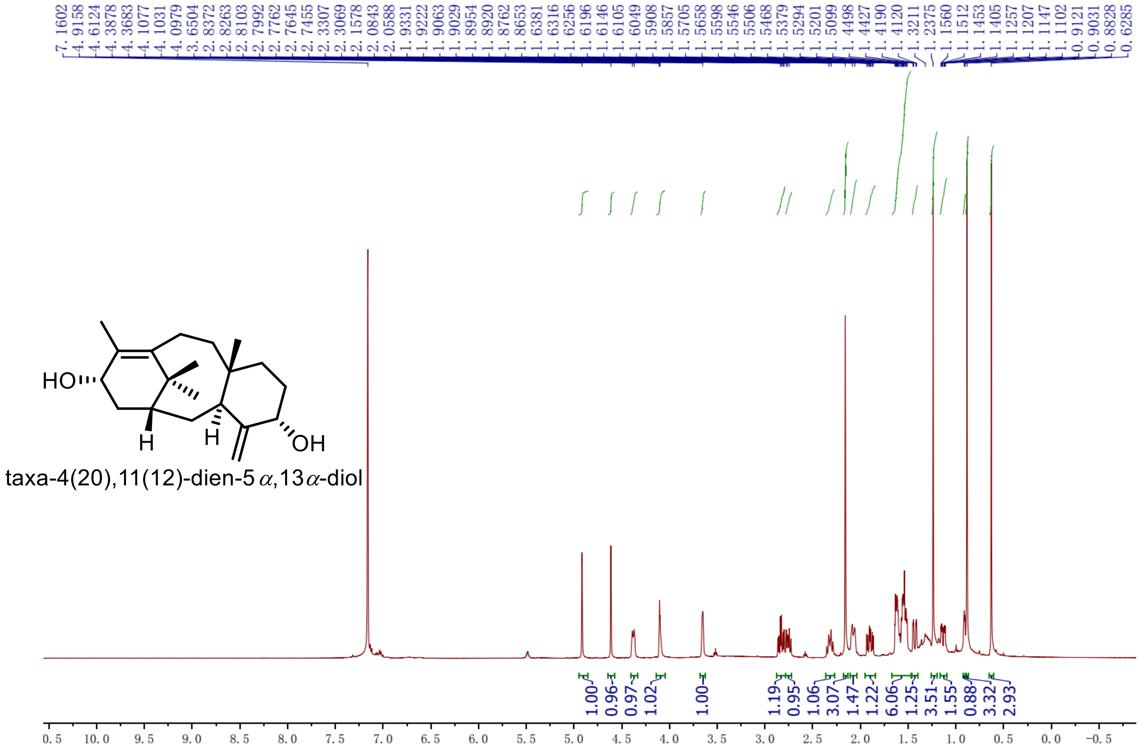

Supplementary Figure 23. ^{1}H NMR spectrum of taxa-4(20),11(12)-diene-5*α*,13*α*-diol (5).

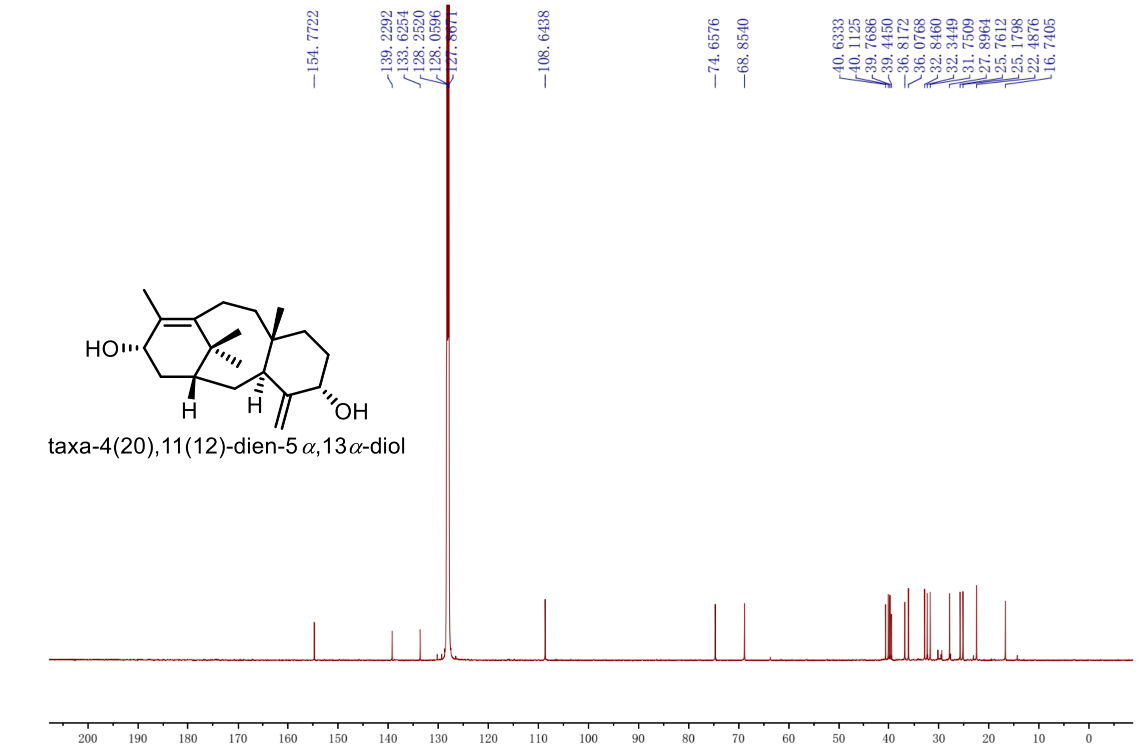

Supplementary Figure 24. ^{13}C NMR spectrum of taxa-4(20),11(12)-diene-5*α*,13*α*-diol (5).

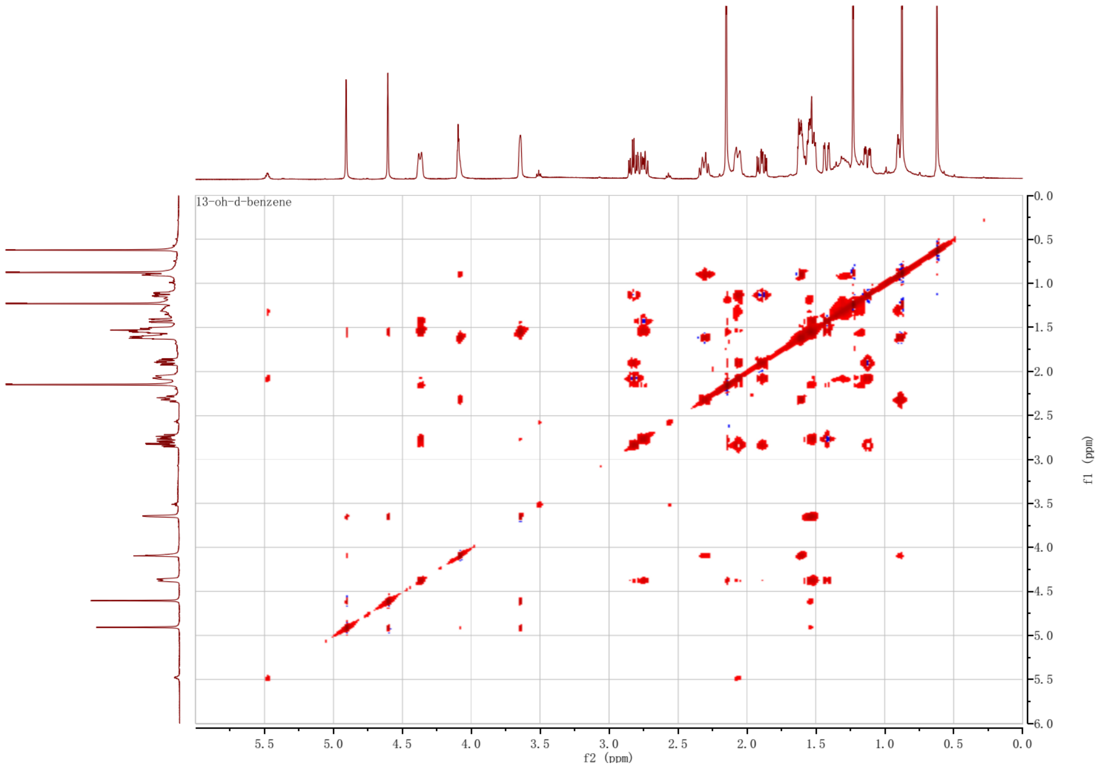

Supplementary Figure 25. 2D-COSY spectrum of taxa-4(20),11(12)-diene-5*α*,13*α*-diol (5).

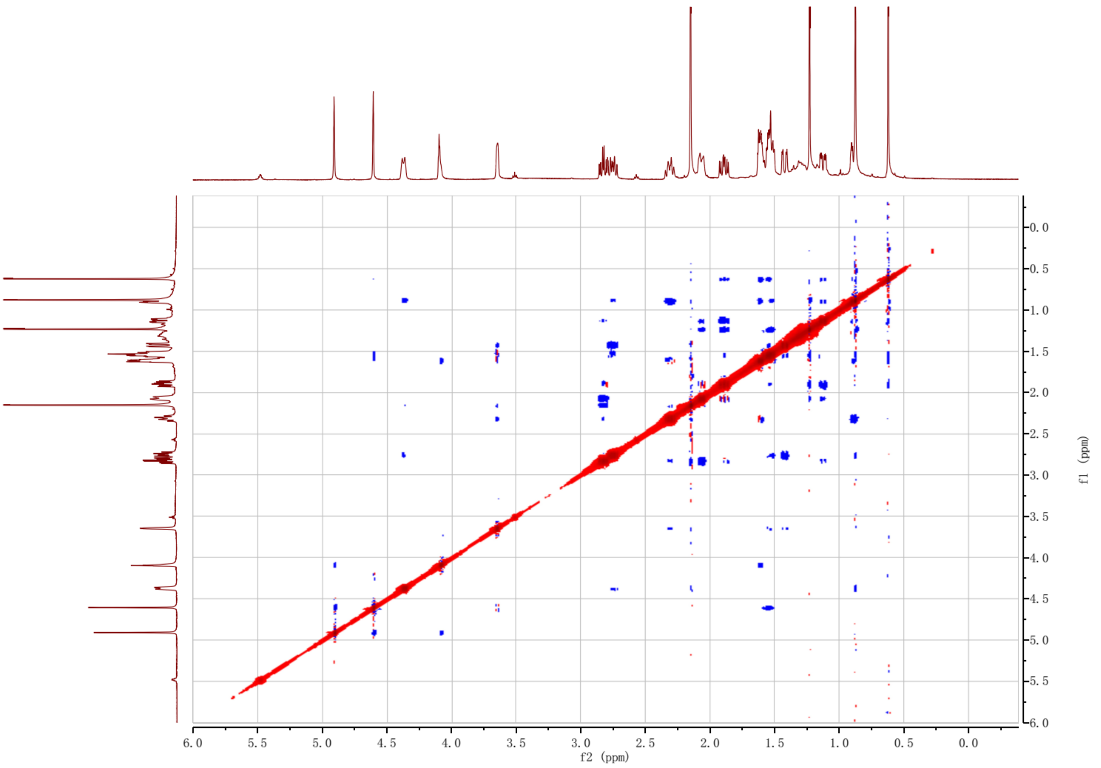

Supplementary Figure 26. 2D-NOE spectrum of taxa-4(20),11(12)-diene-5*α*,13*α*-diol (5).

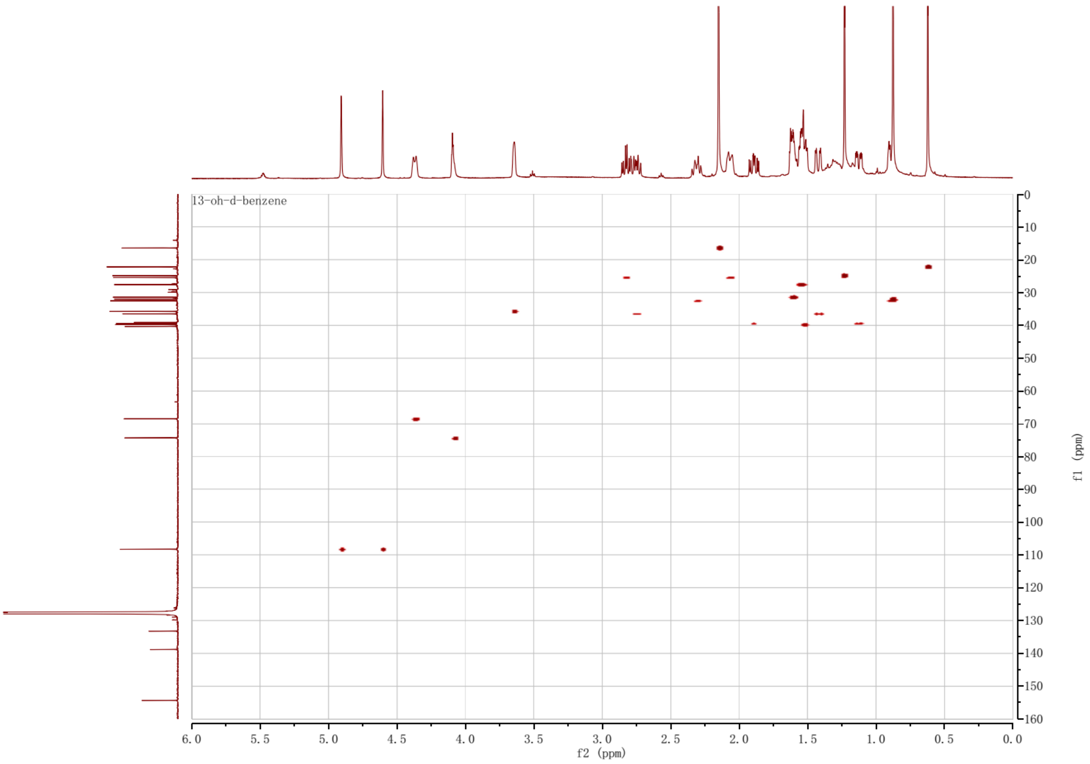

Supplementary Figure 27. 2D-HSQC spectrum of taxa-4(20),11(12)-diene-5*α*,13*α*-diol (5).

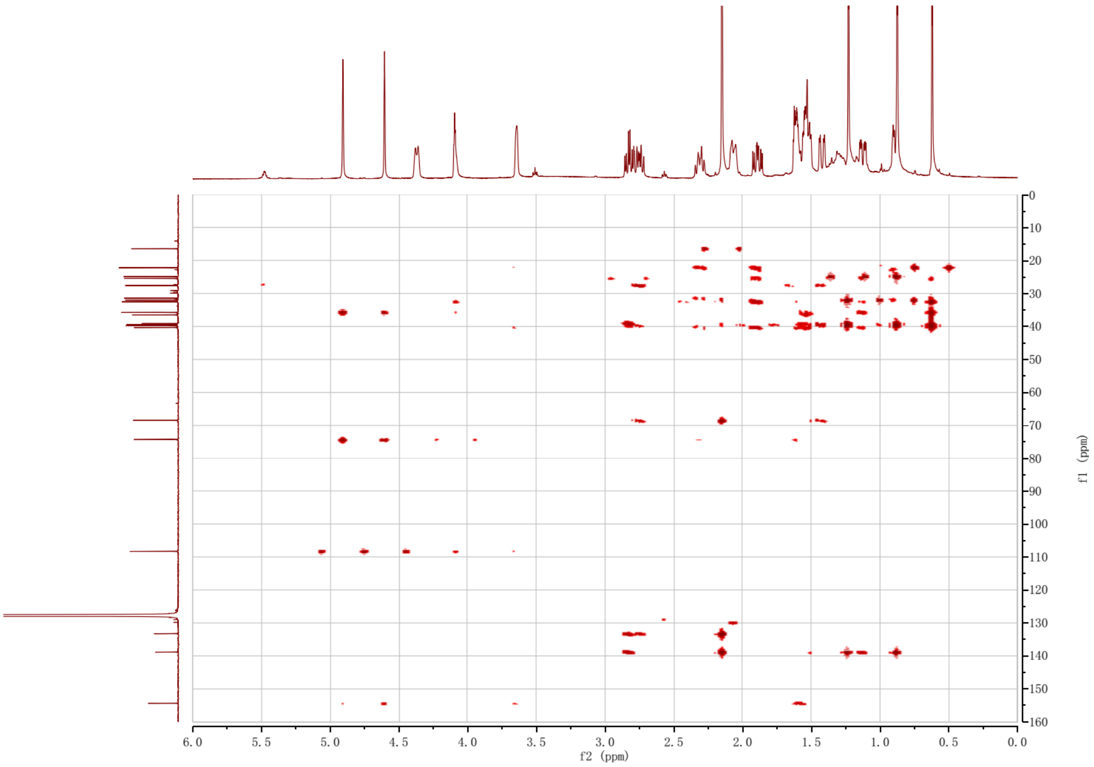

Supplementary Figure 28. 2D-HMBC spectrum of taxa-4(20),11(12)-diene-5*α*,13*α*-diol (5).

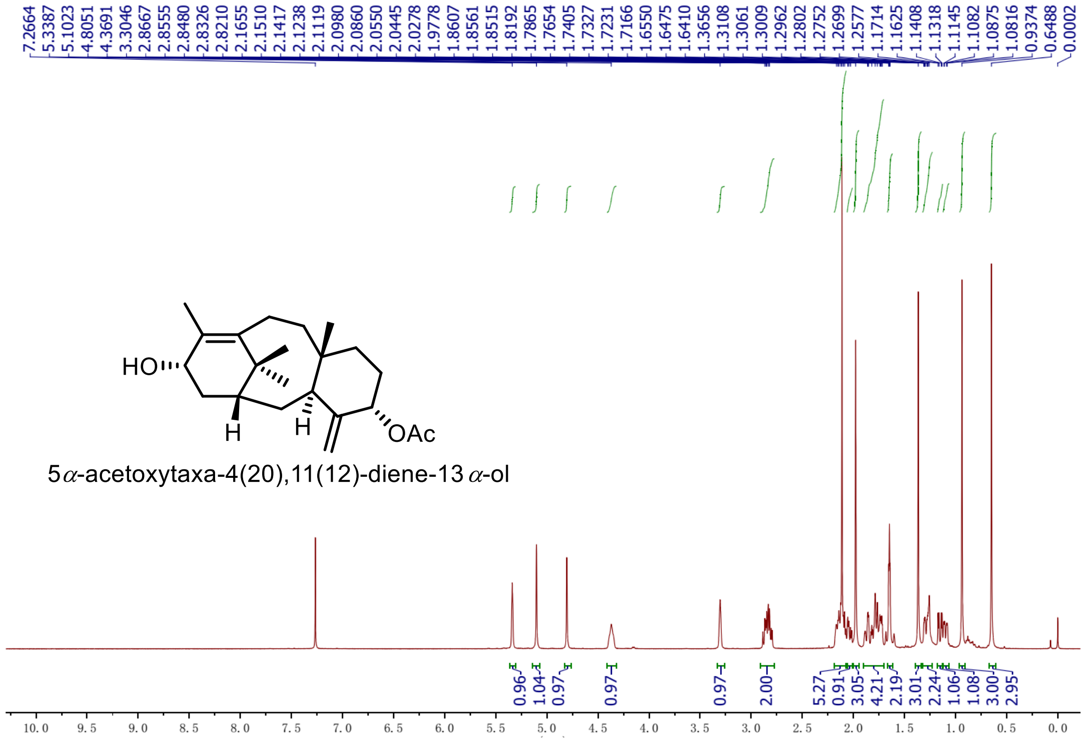

Supplementary Figure 29. ^{1}H NMR spectrum of 5α-acetoxytaxa-4(20),11(12)-diene-13α-ol (6).

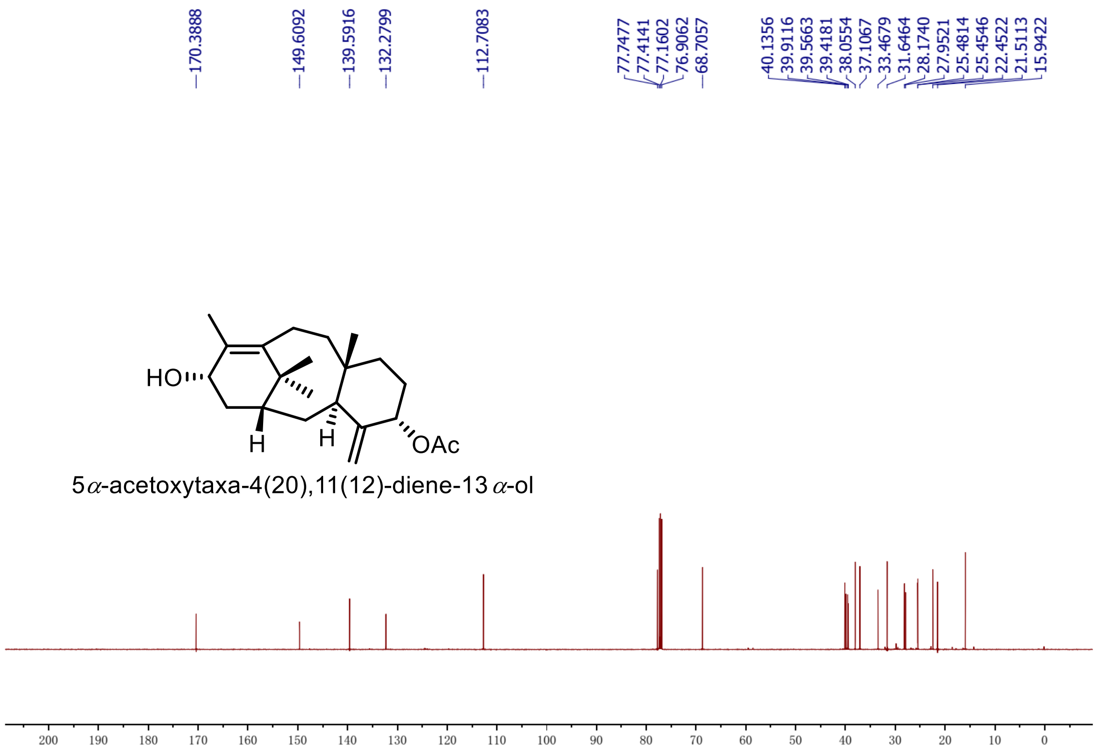

Supplementary Figure 30. ^{13}C NMR spectrum of 5α-acetoxytaxa-4(20),11(12)-diene-13α-ol (6).

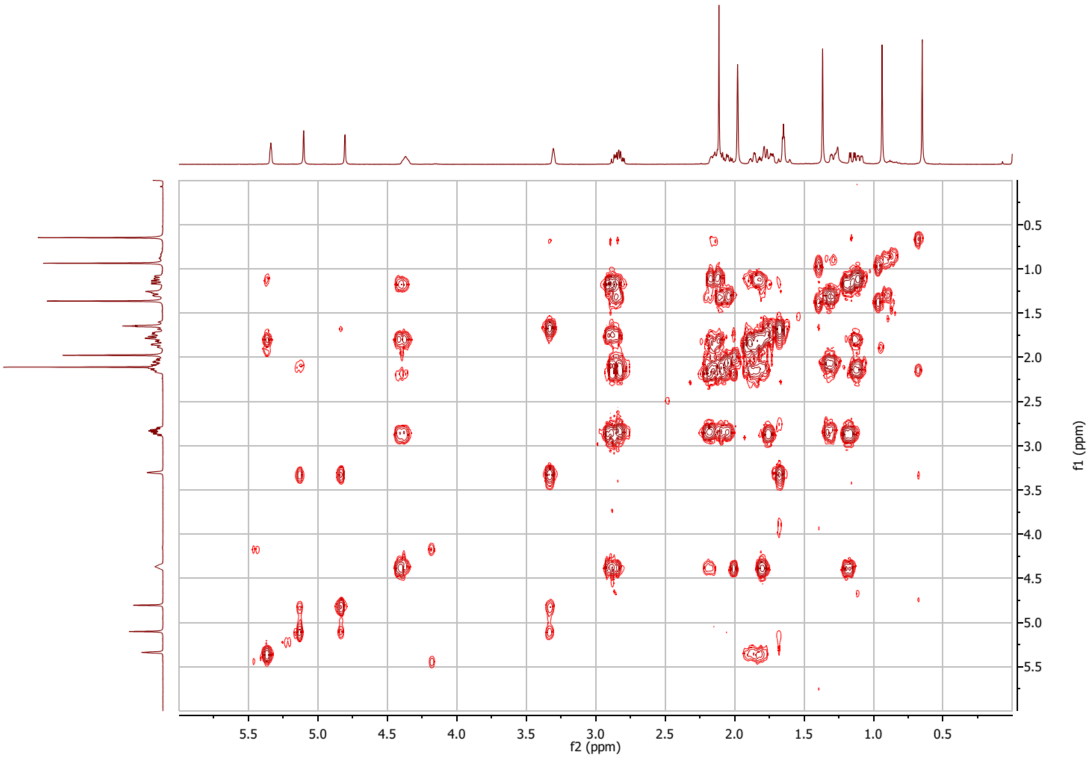

Supplementary Figure 31. 2D-COSY NMR spectrum of 5α-acetoxytaxa-4(20),11(12)-diene-13α-ol (6).

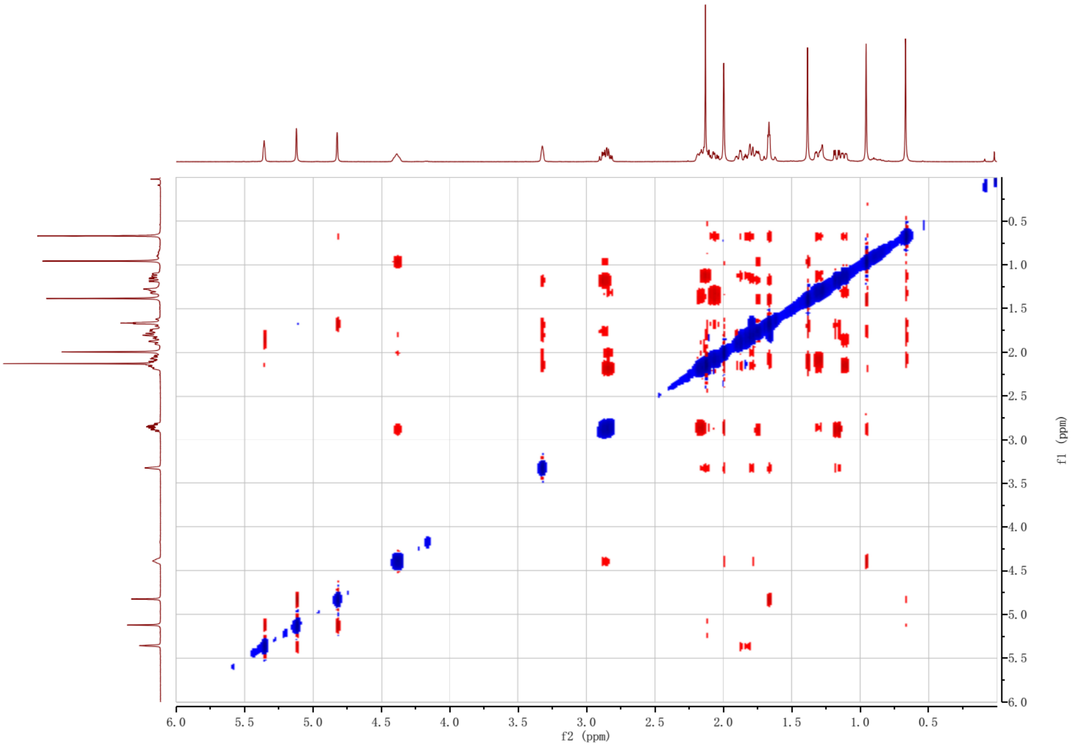

Supplementary Figure 32. 2D-NOE NMR spectrum of 5α-acetoxytaxa-4(20),11(12)-diene-13α-ol (6).

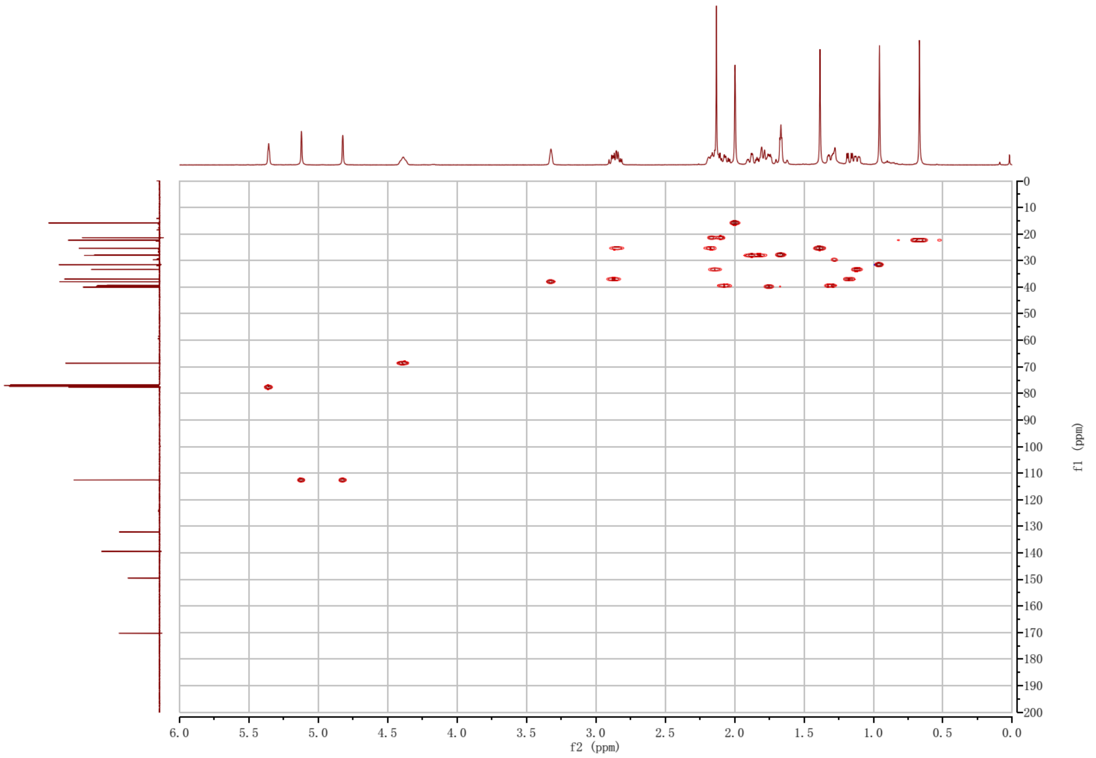

Supplementary Figure 33. 2D-HSQC NMR spectrum of 5*α*-acetoxytaxa-4(20),11(12)-diene-13*α*-ol (6).

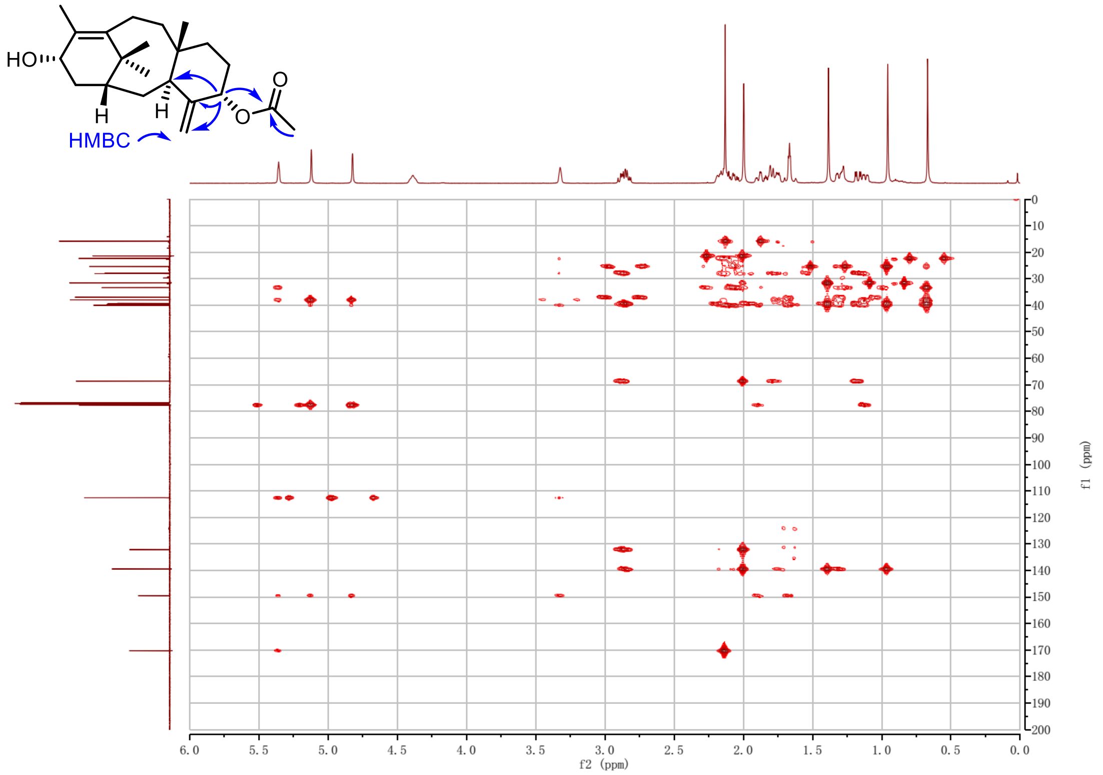

Supplementary Figure 34. 2D-HMBC NMR spectrum of 5*α*-acetoxytaxa-4(20),11(12)-diene-13*α*-ol (6).

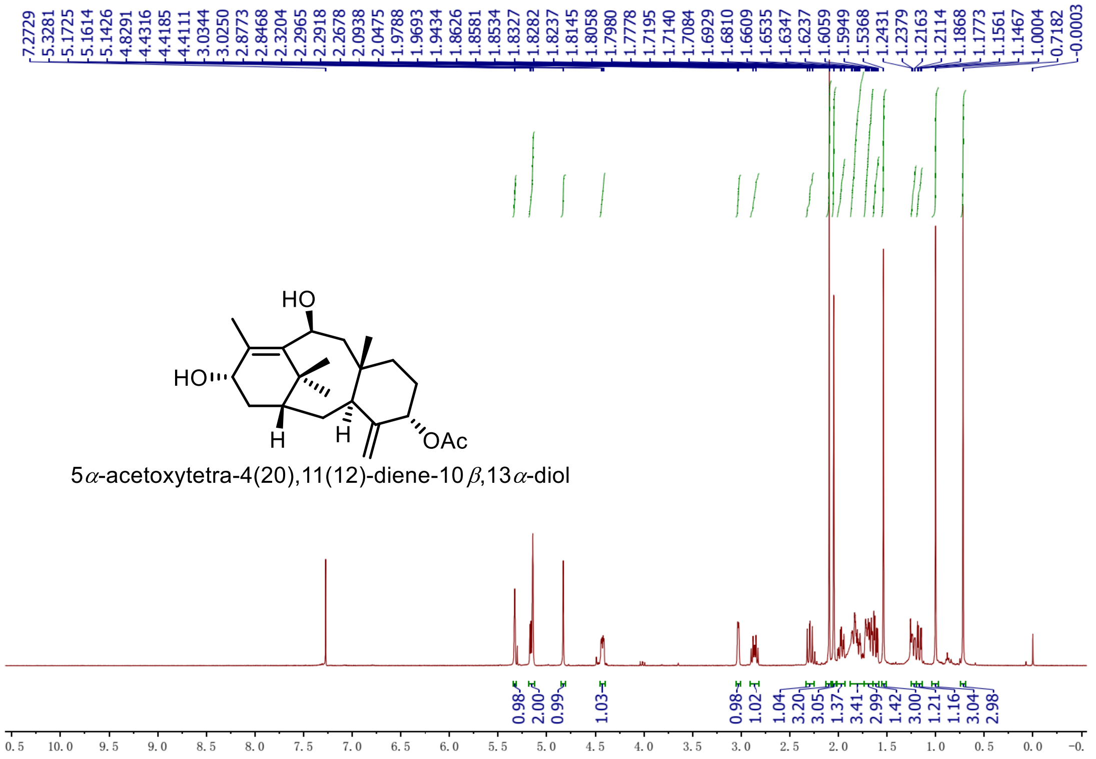

Supplementary Figure 35. ^{1}**H NMR spectrum of 5*α*-acetoxytetra-4(20), 11(12)-diene-10*β*, 13*α*-diol (7).**

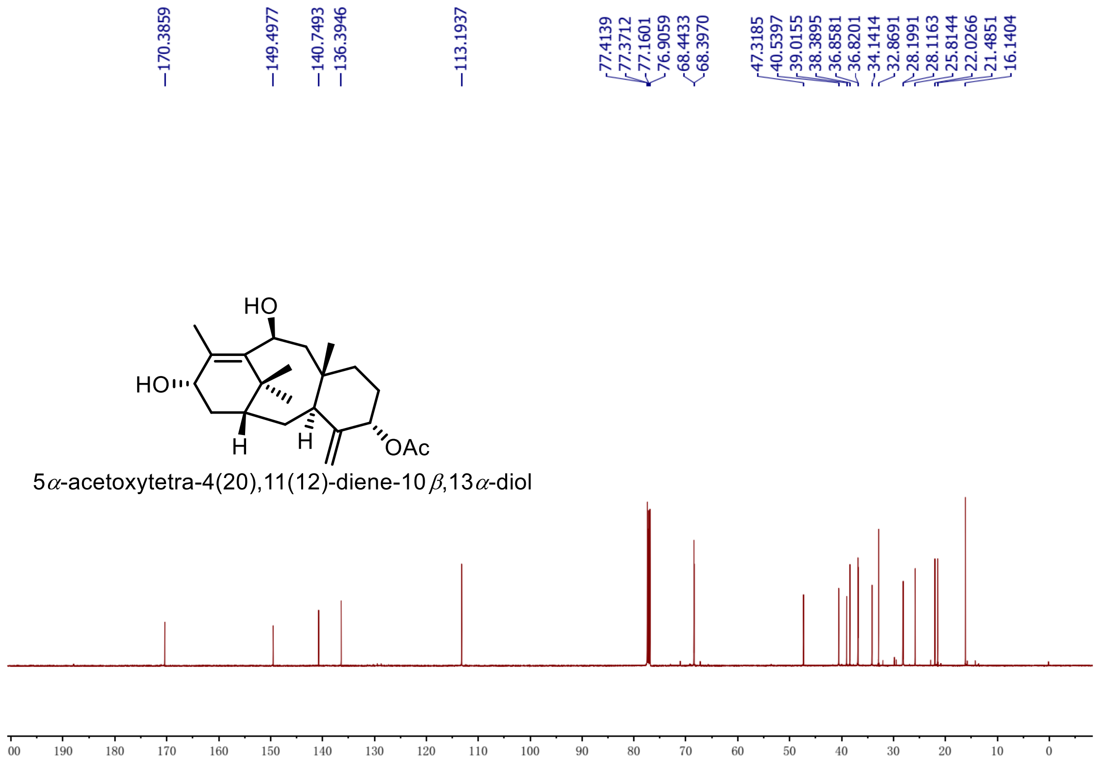

Supplementary Figure 36. ^{13}**C NMR spectrum of 5*α*-acetoxytetra-4(20), 11(12)-diene-10*β*, 13*α*-diol (7).**

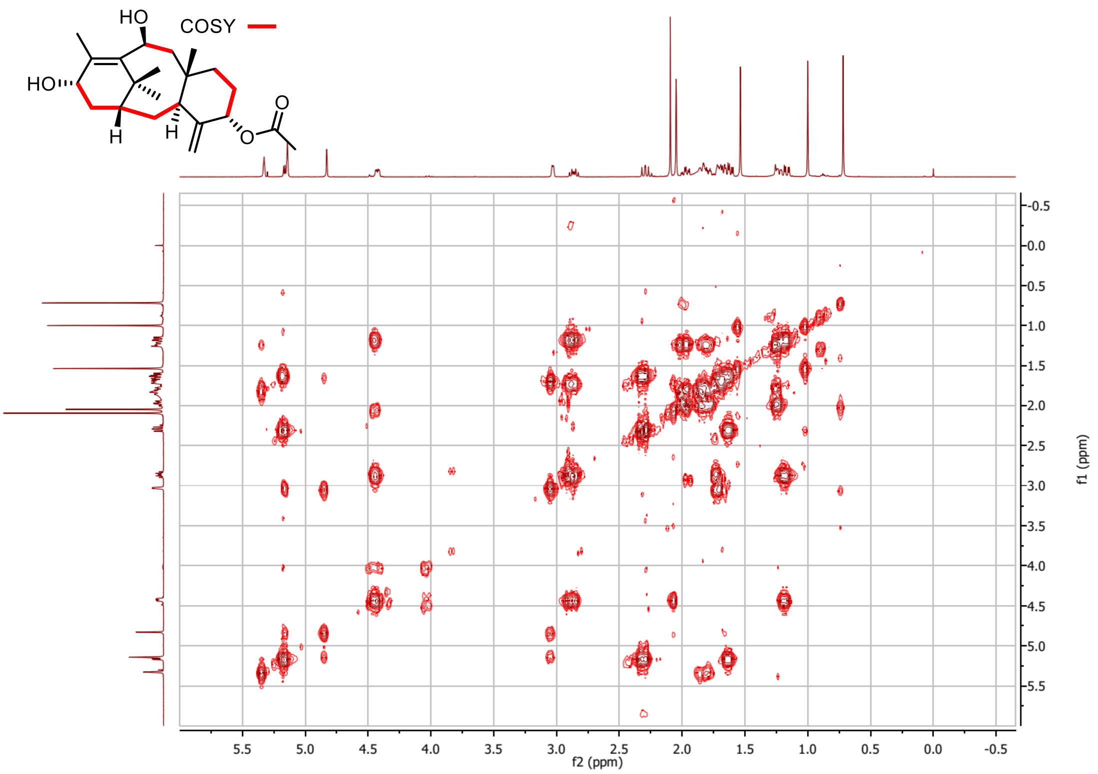

Supplementary Figure 37. 2D-COSY NMR spectrum of 5*α*-acetoxytetra-4(20), 11(12)-diene-10*β*, 13*α*-diol (7).

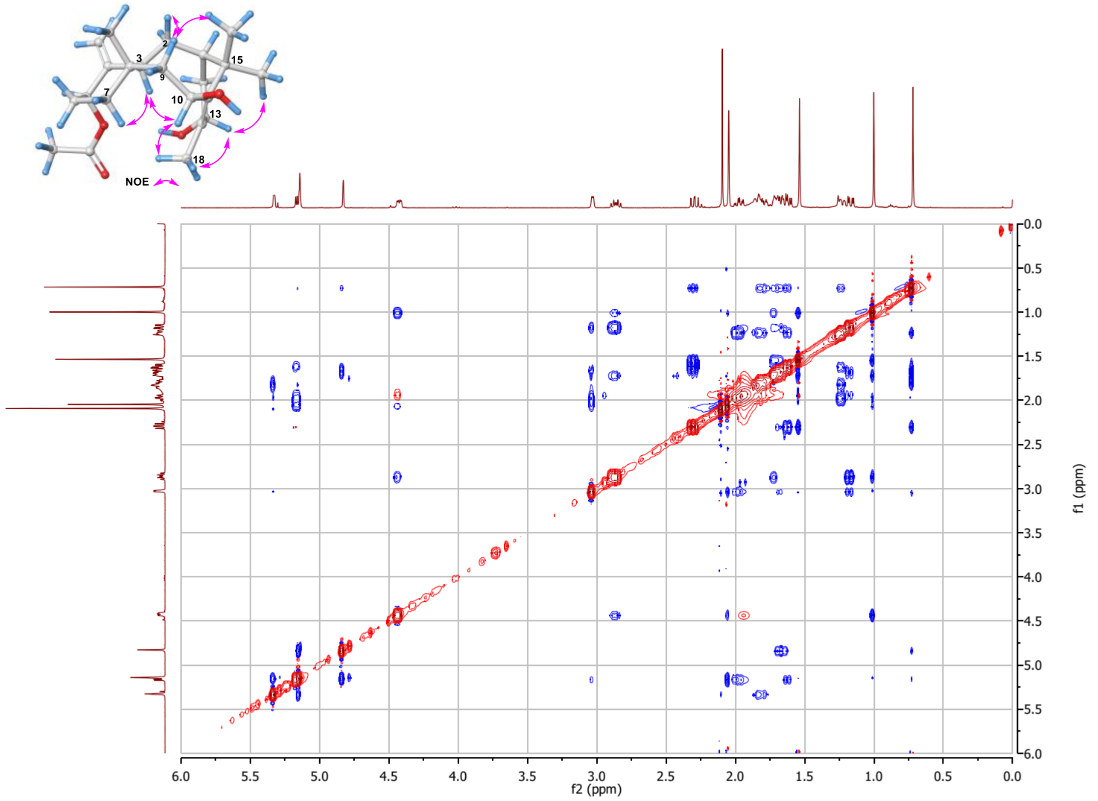

Supplementary Figure 38. 2D-NOE NMR spectrum of 5*α*-acetoxytetra-4(20), 11(12)-diene-10*β*, 13*α*-diol (7).

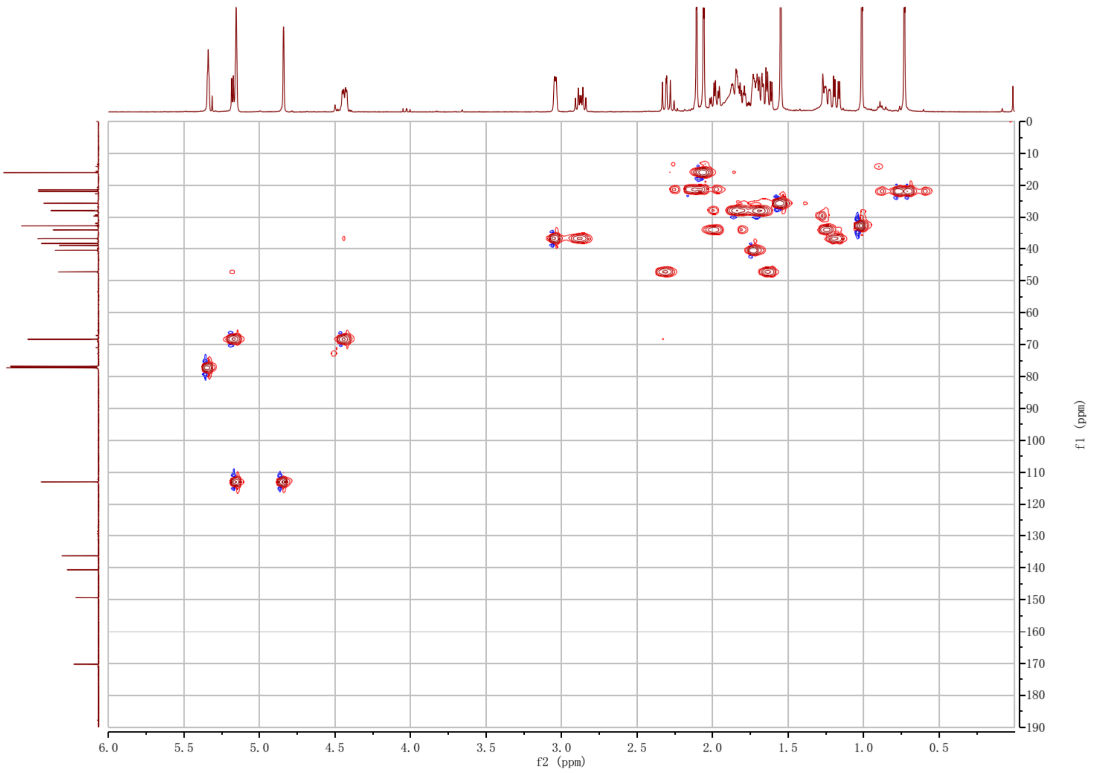

Supplementary Figure 39. 2D-HSQC NMR spectrum of 5*α*-acetoxytetra-4(20), 11(12)-diene-10*β*, 13*α*-diol (7).

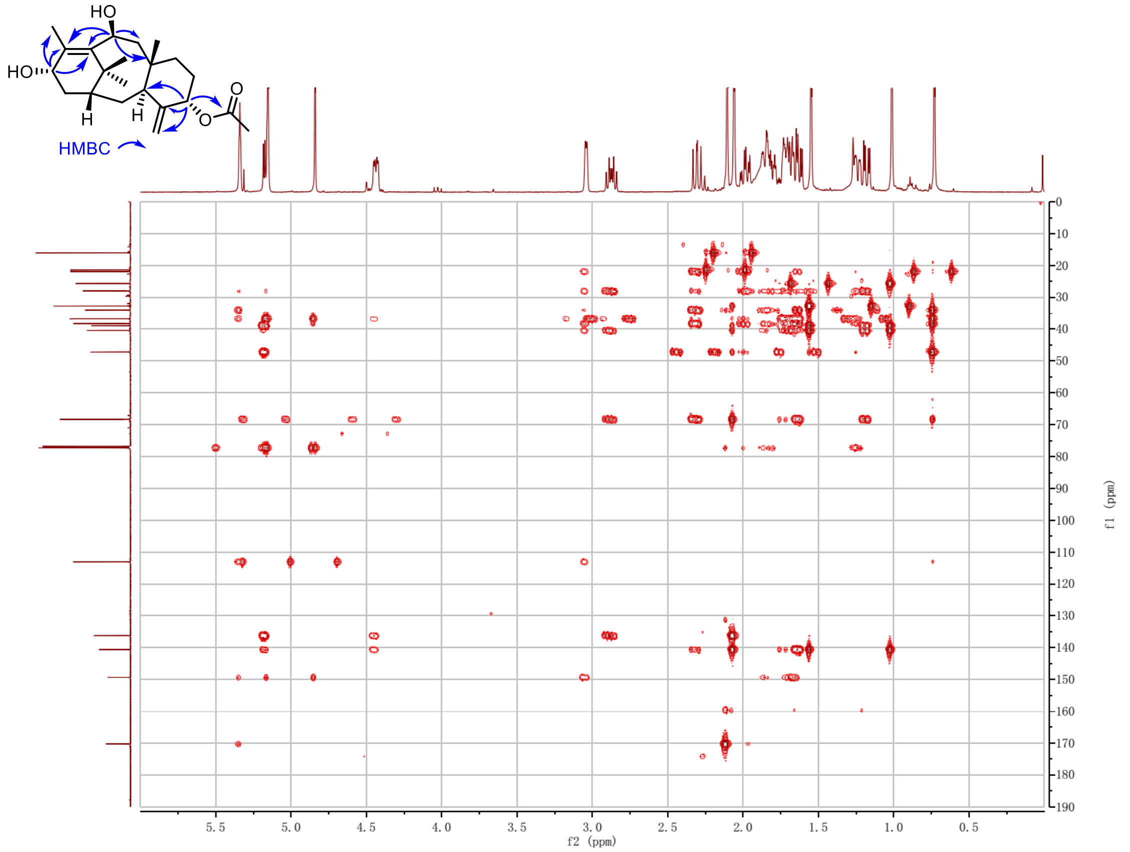

Supplementary Figure 40. 2D-HMBC NMR spectrum of 5*α*-acetoxytetra-4(20), 11(12)-diene-10*β*, 13*α*-diol (7).

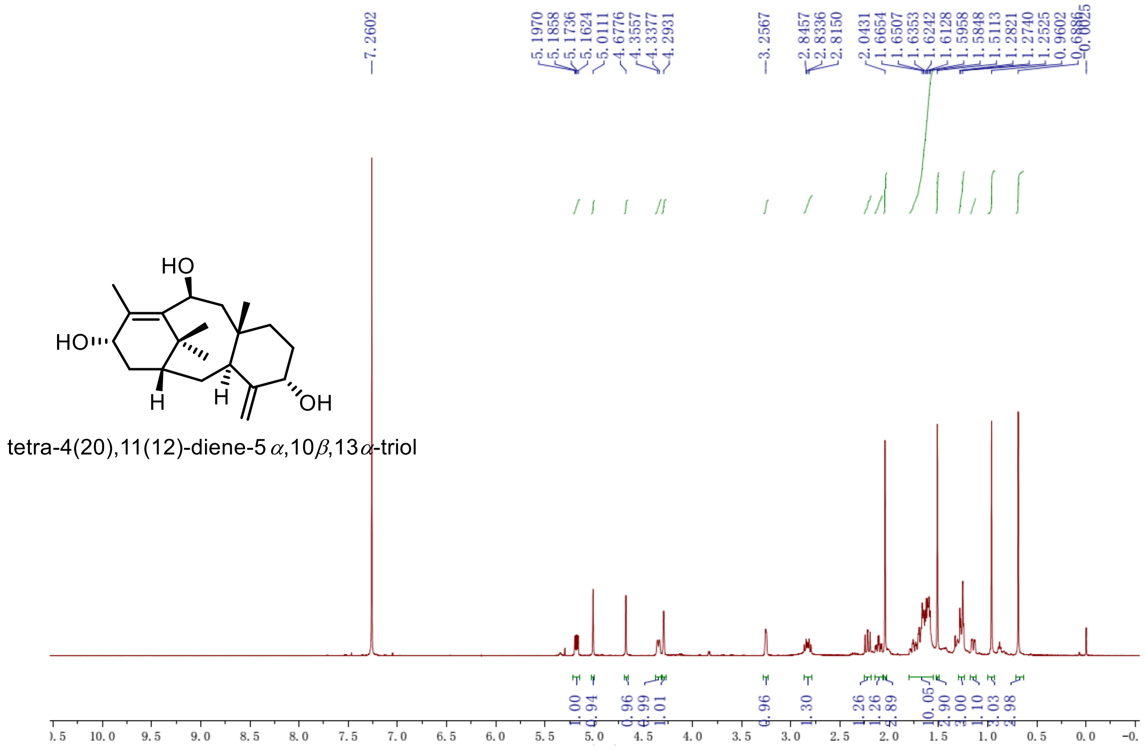

Supplementary Figure 41. ^{1}H NMR spectrum of tetra-4(20),11(12)-diene-5*α*,10*β*,13*α*–triol (8).

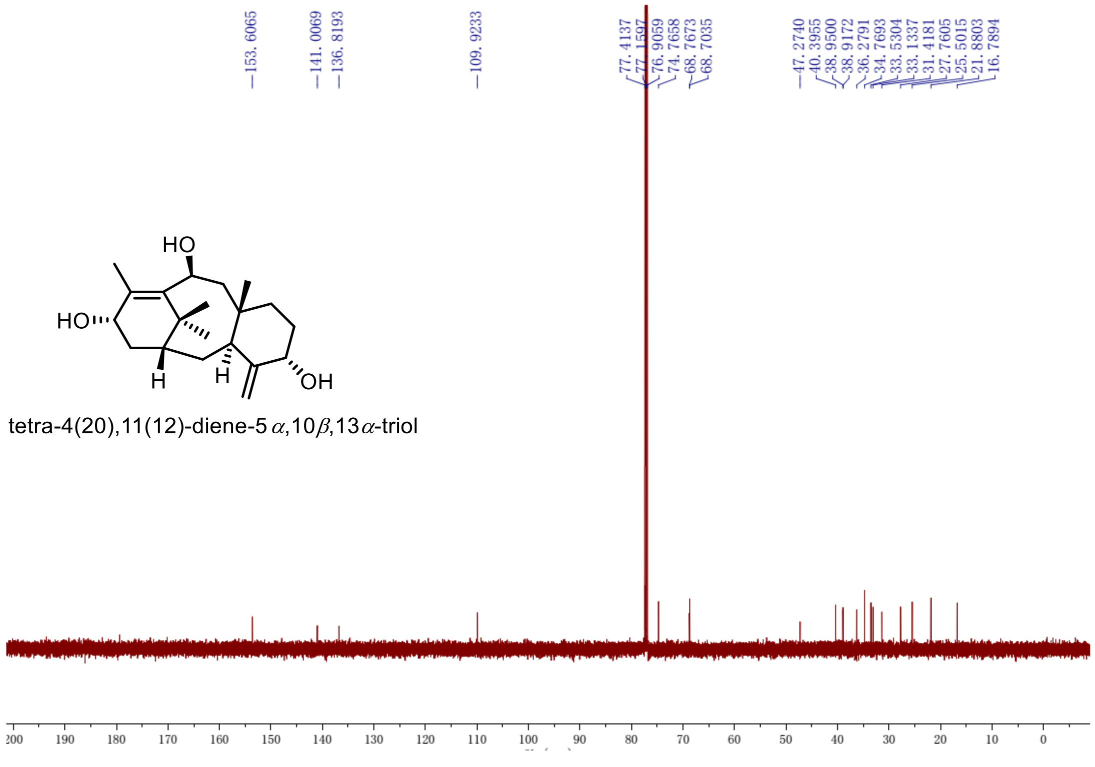

Supplementary Figure 42. ^{13}C NMR spectrum of tetra-4(20),11(12)-diene-5*α*,10*β*,13*α*–triol (8).

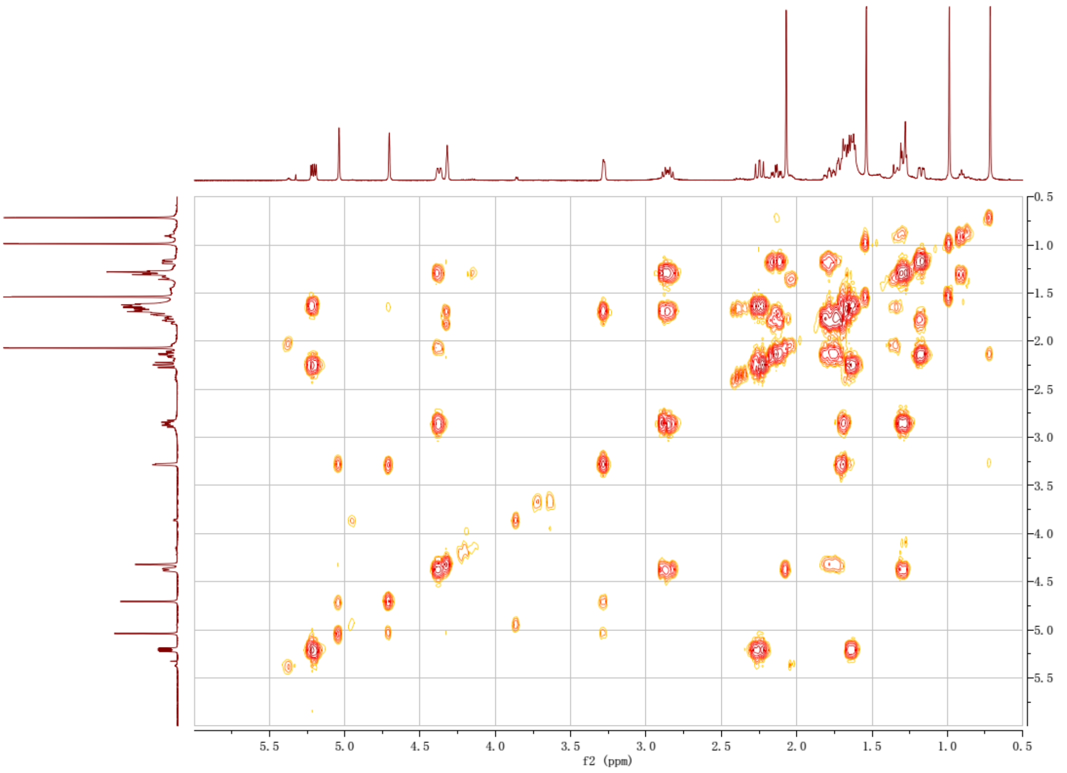

Supplementary Figure 43. 2D-COSY NMR spectrum of tetra-4(20),11(12)-diene-5α,10β,13α–triol (8).

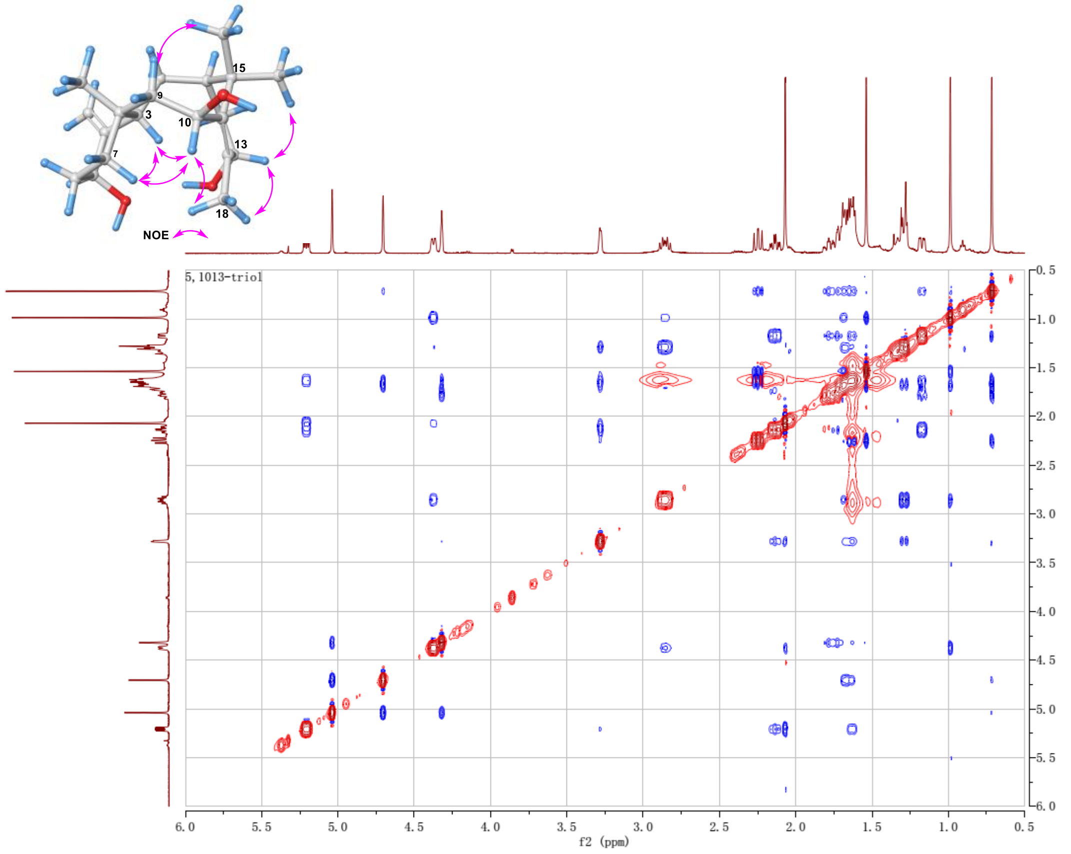

Supplementary Figure 44. 2D-NOE NMR spectrum of tetra-4(20),11(12)-diene-5α,10β,13α–triol (8).

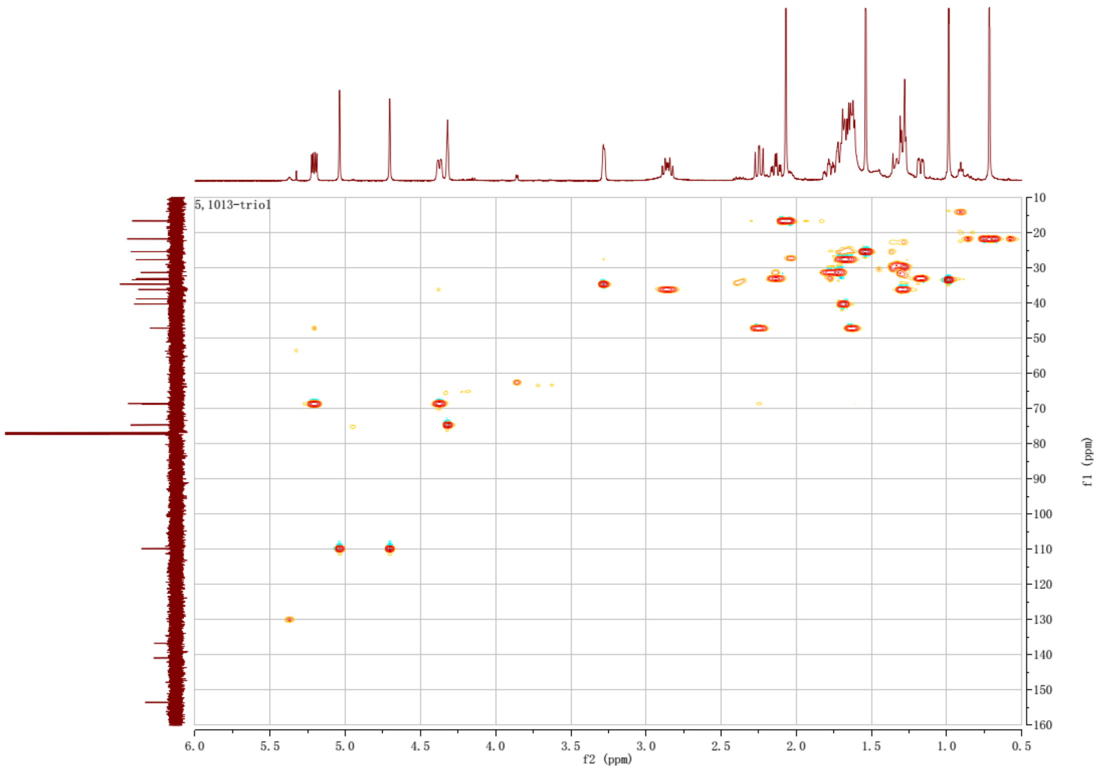

Supplementary Figure 45. 2D-HSQC NMR spectrum of tetra-4(20),11(12)-diene-5*α*,10*β*,13*α*–triol (8).

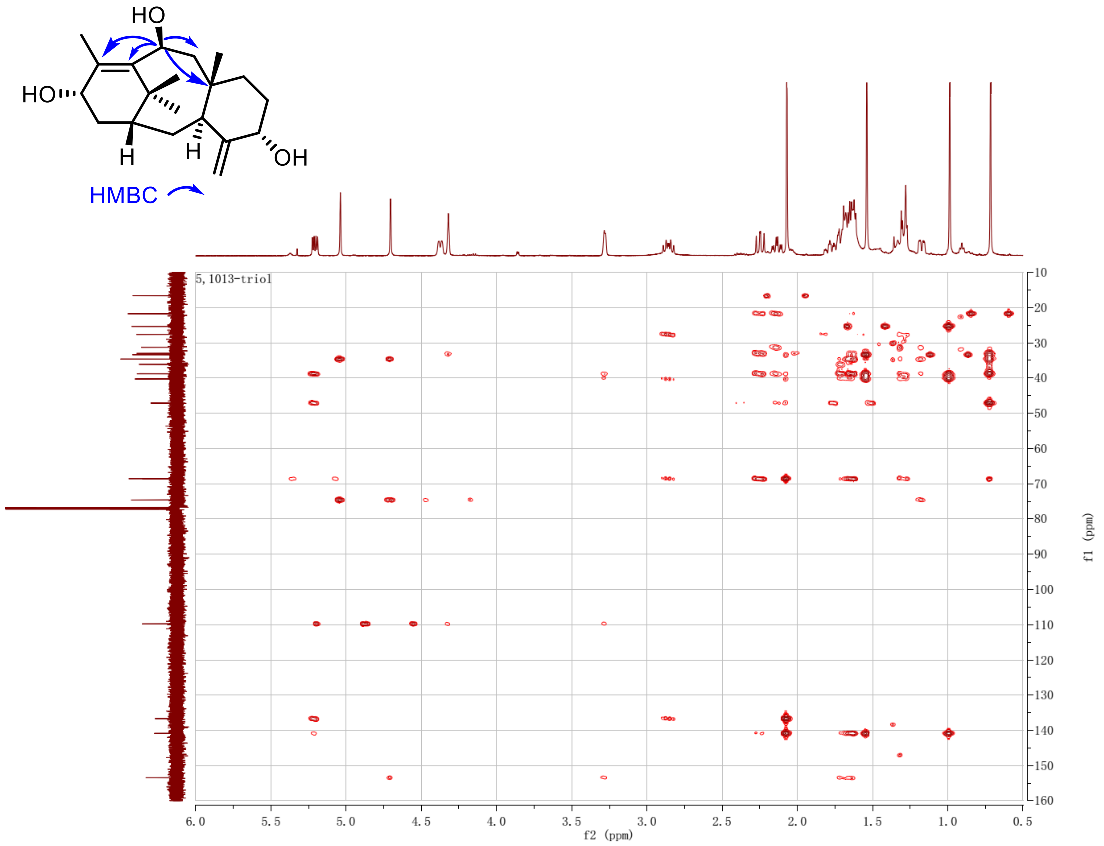

Supplementary Figure 46. 2D-HMBC NMR spectrum of tetra-4(20),11(12)-diene-5*α*,10*β*,13*α*–triol (8).

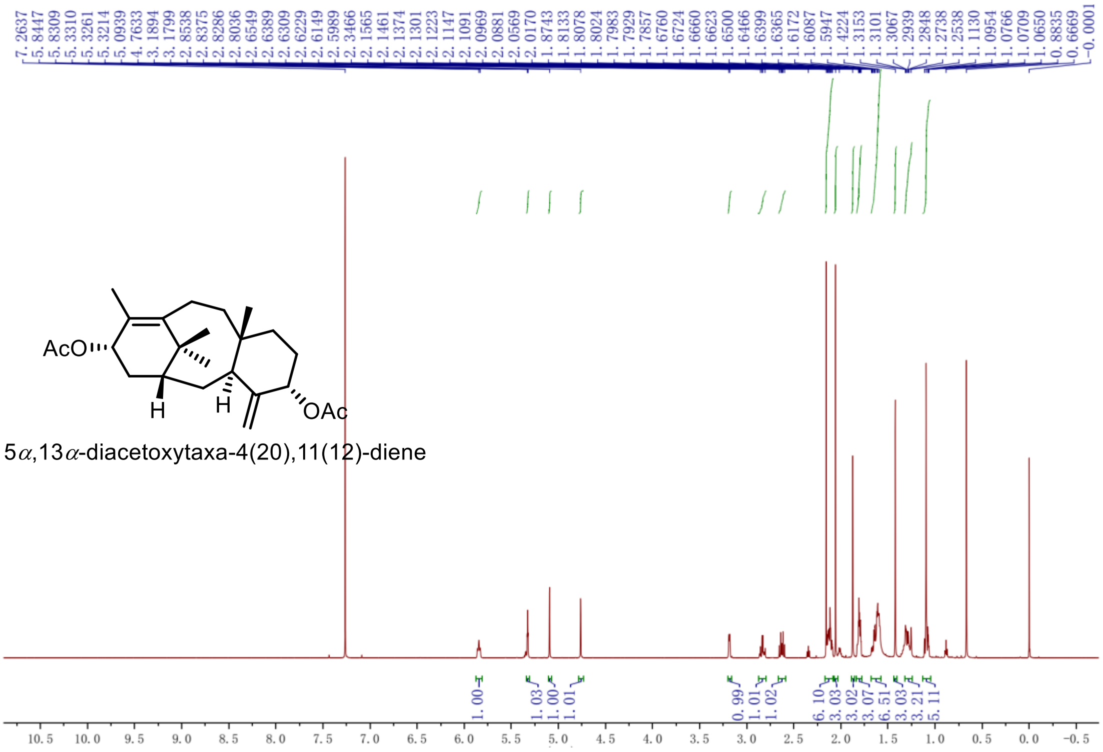

Supplementary Figure 47. ^{1}H NMR spectrum of 5*α*,13*α*-diacetoxytaxa-4(20),11(12)-diene (9).

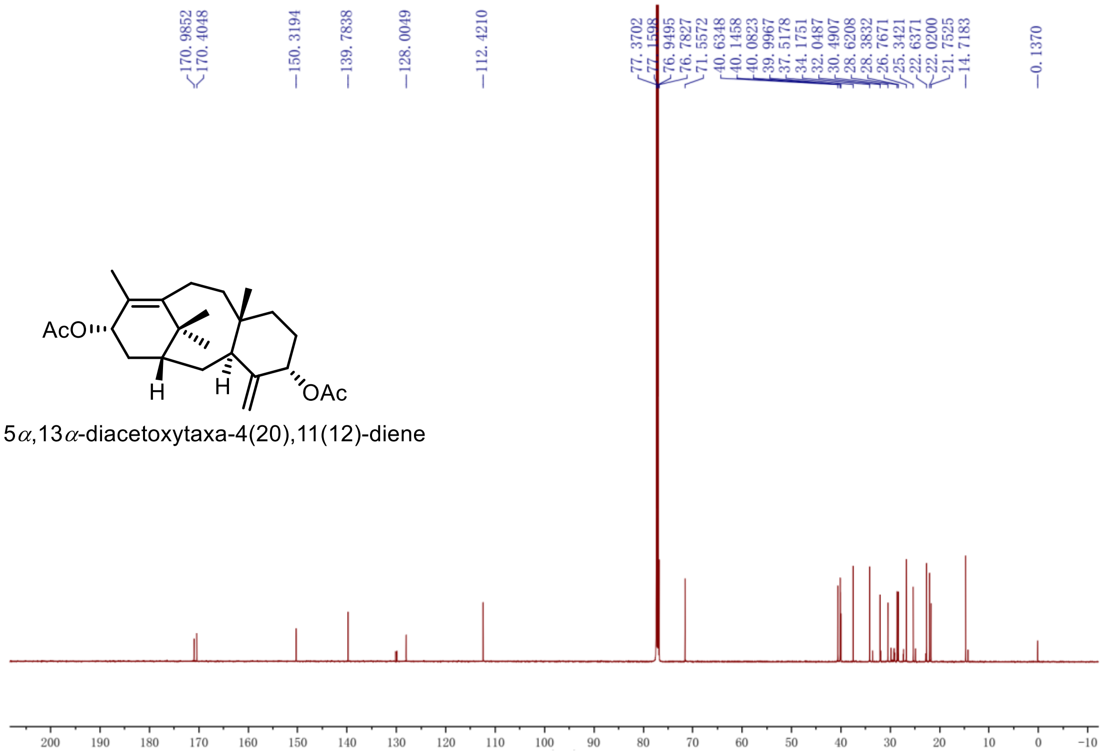

Supplementary Figure 48. ^{13}C NMR spectrum of 5*α*,13*α*-diacetoxytaxa-4(20),11(12)-diene (9).

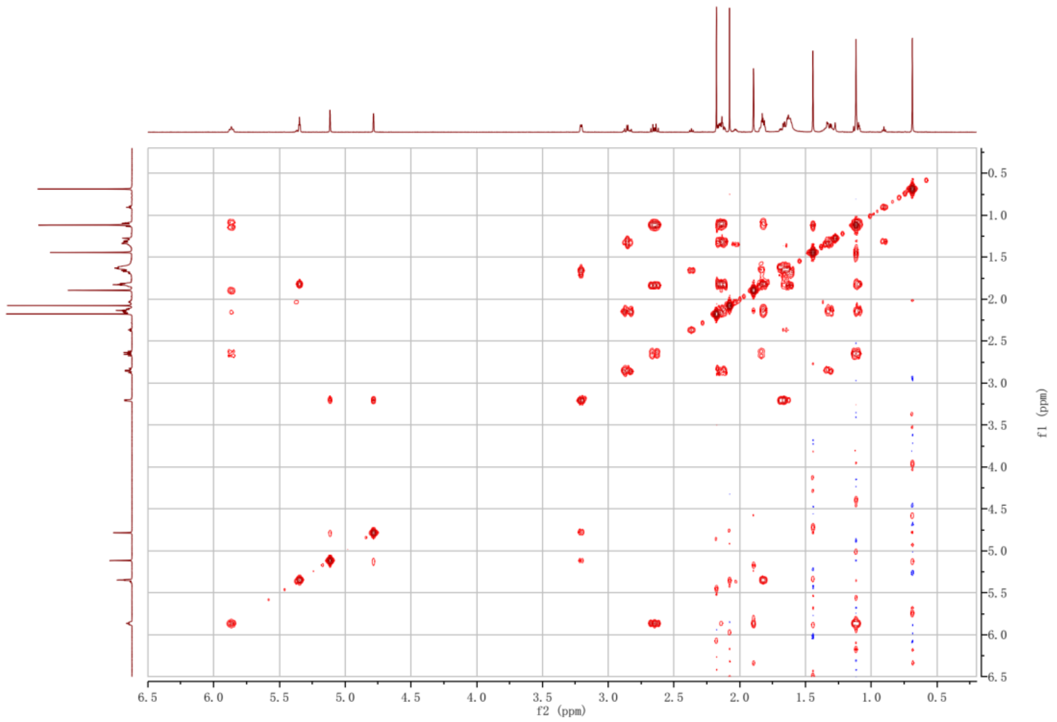

Supplementary Figure 49. 2D-COSY NMR spectrum of 5*α*,13*α*-diacetoxytaxa-4(20),11(12)-diene (9).

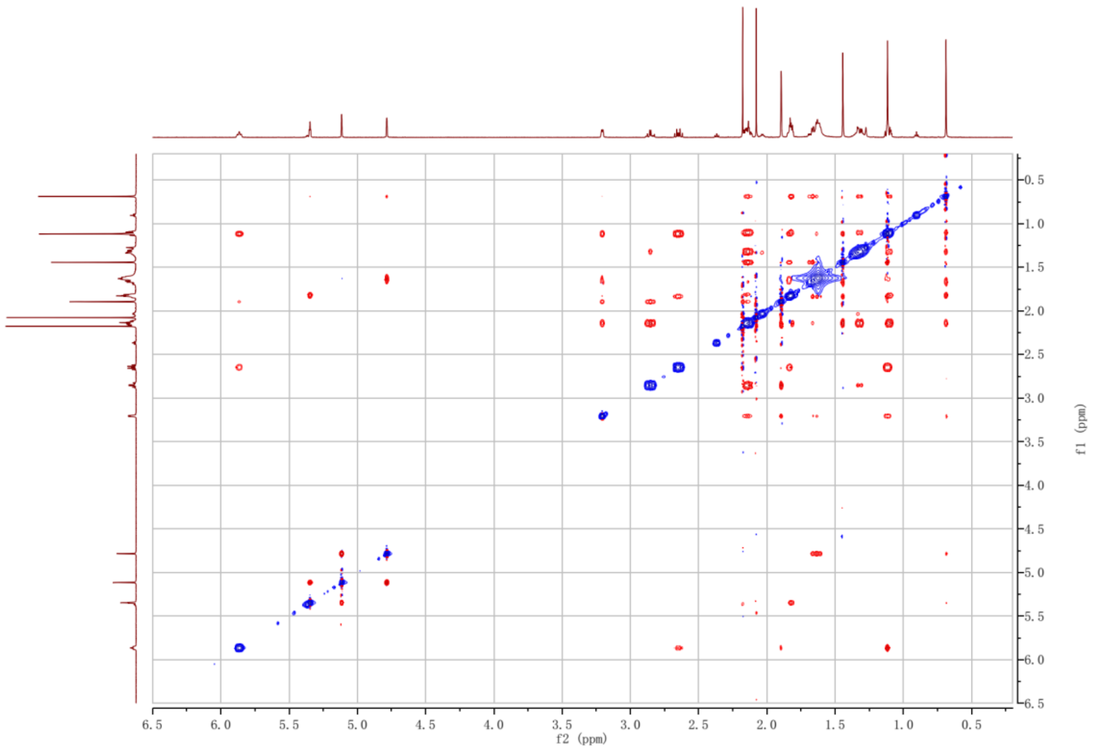

Supplementary Figure 50. 2D-NOE NMR spectrum of 5*α*,13*α*-diacetoxytaxa-4(20),11(12)-diene (9).

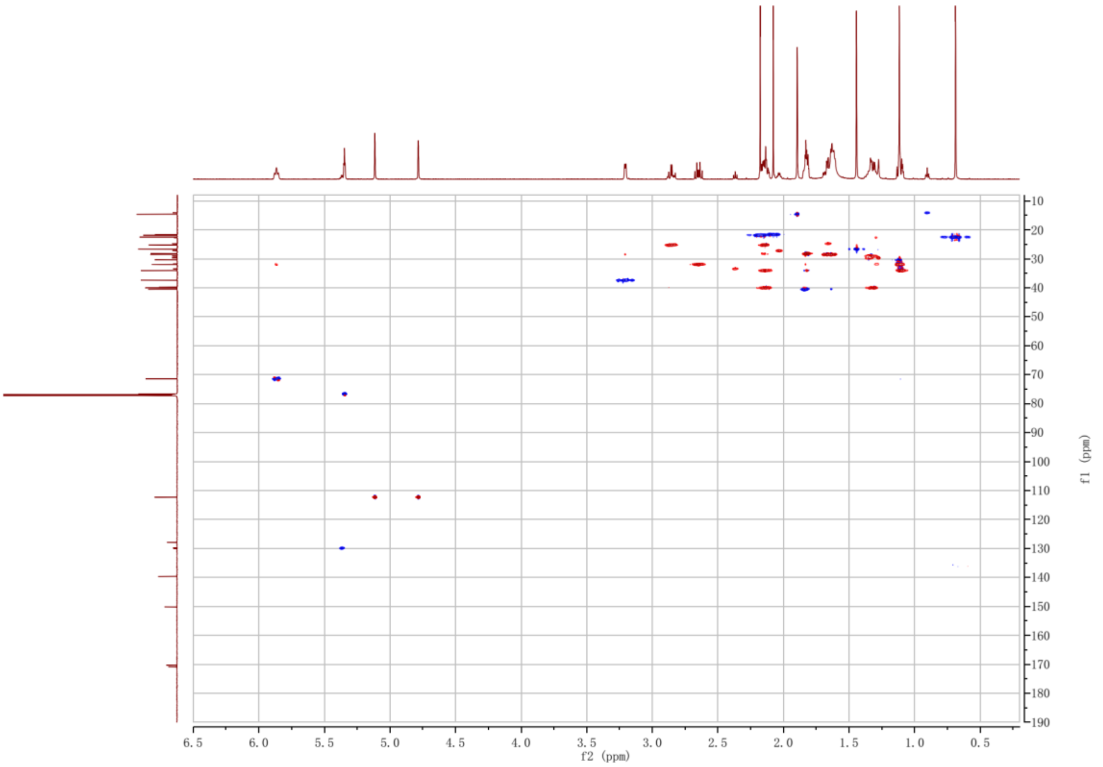

Supplementary Figure 51. 2D-HSQC NMR spectrum of 5α,13α-diacetoxytaxa-4(20),11(12)-diene (9).

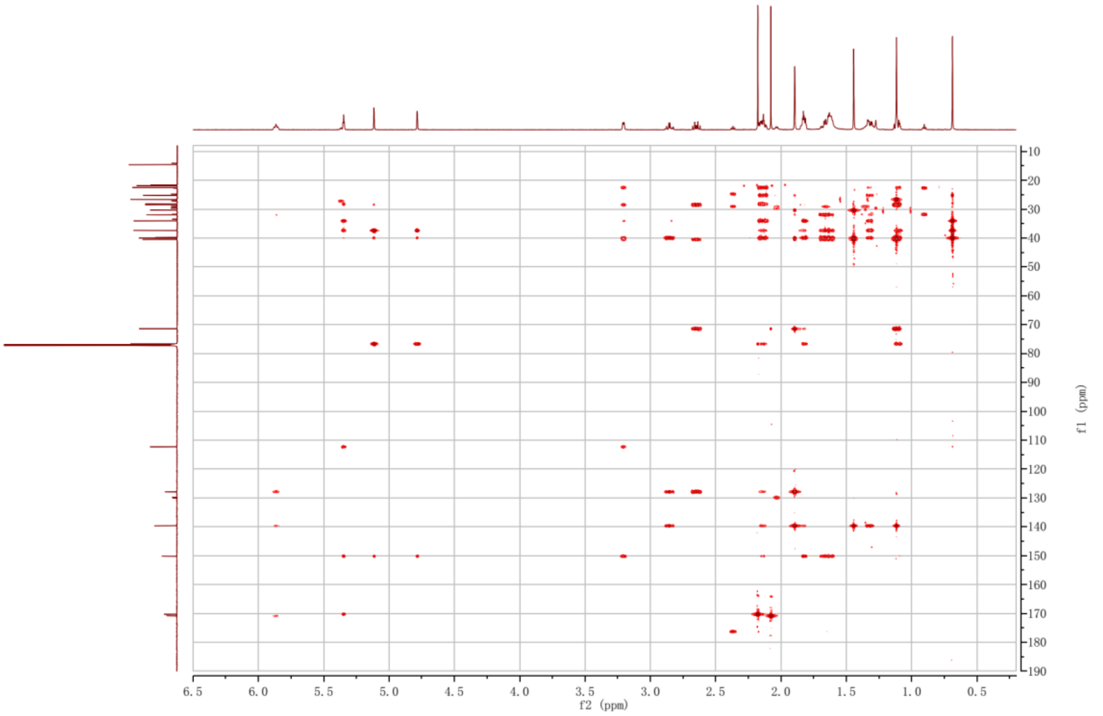

Supplementary Figure 52. 2D-HMBC NMR spectrum of 5α,13α-diacetoxytaxa-4(20),11(12)-diene (9).

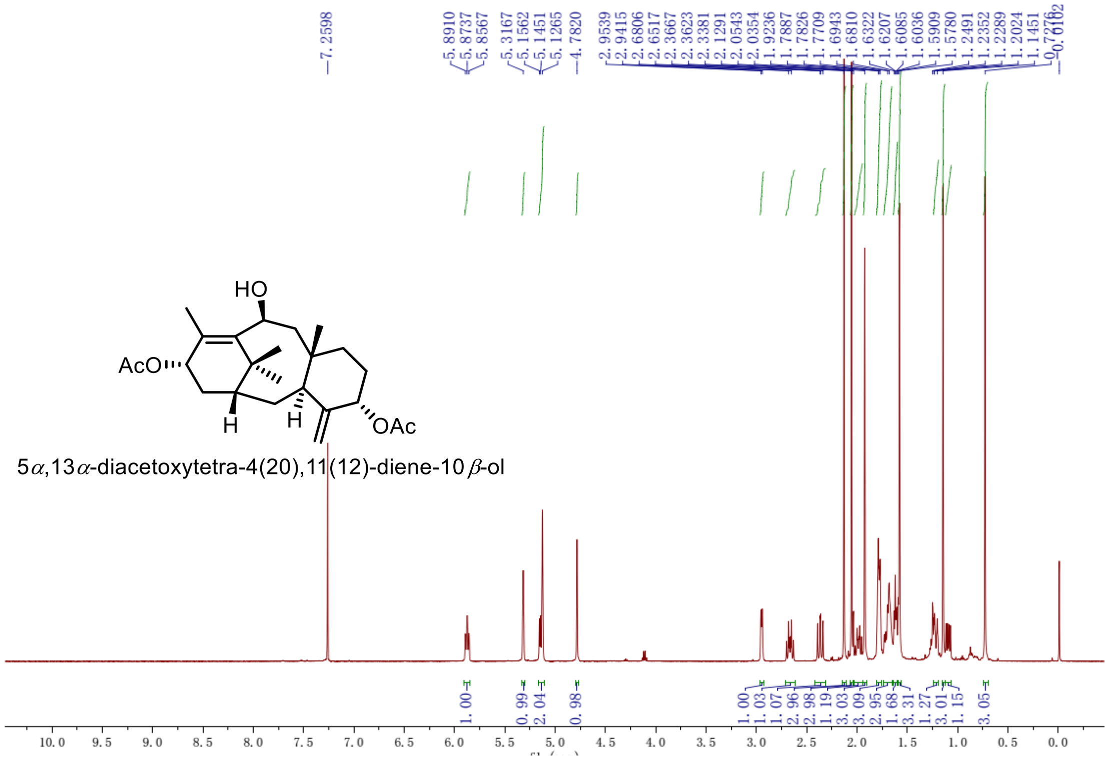

Supplementary Figure 53. ^{1}**H NMR spectrum of 5*α*,13*α*-diacetoxytaxa-4(20),11(12)-diene-10*β*-ol (10).**

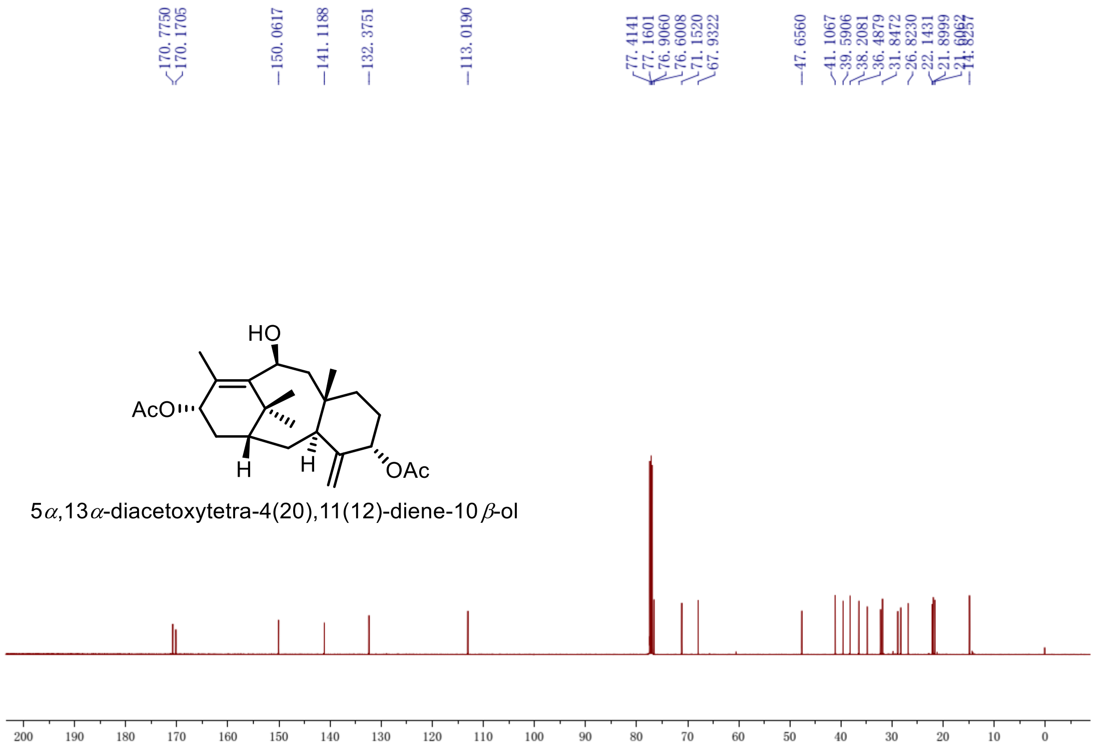

Supplementary Figure 54. ^{13}**C NMR spectrum of 5*α*,13*α*-diacetoxytaxa-4(20),11(12)-diene-10*β*-ol (10).**

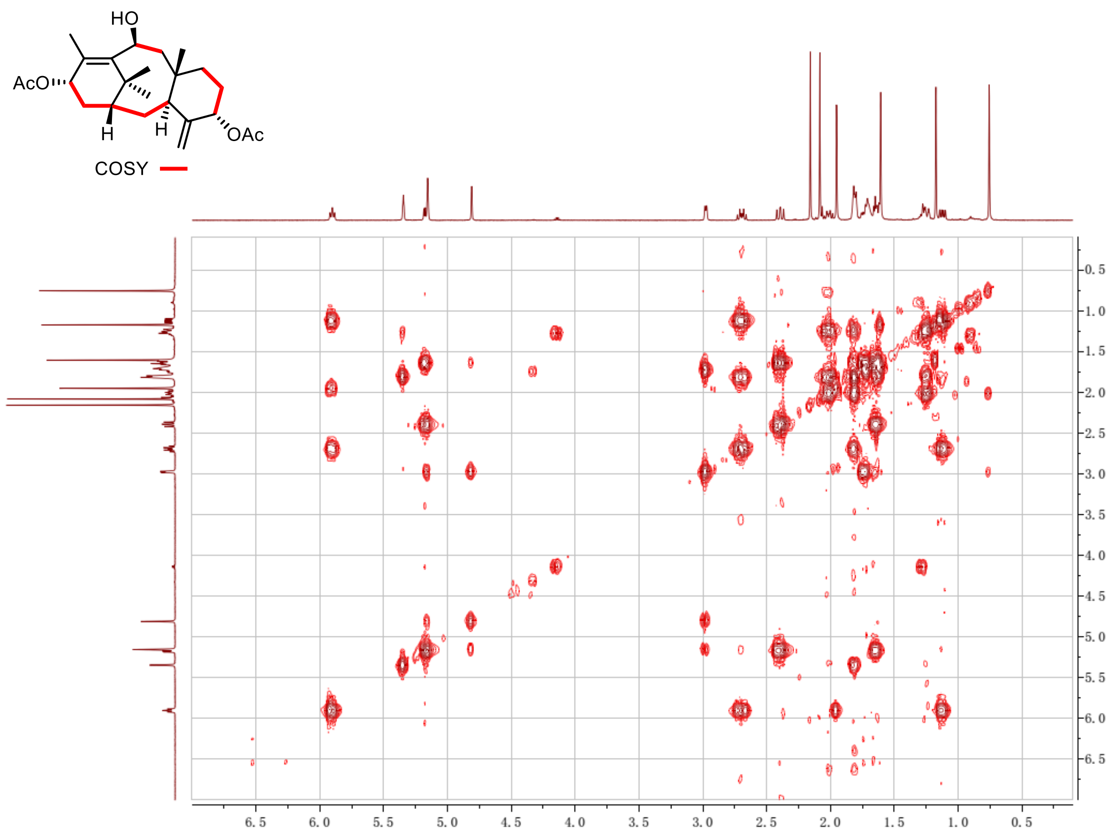

Supplementary Figure 55. 2D-COSY NMR spectrum of 5α,13α-diacetoxytaxa-4(20),11(12)-diene-10β-ol (10).

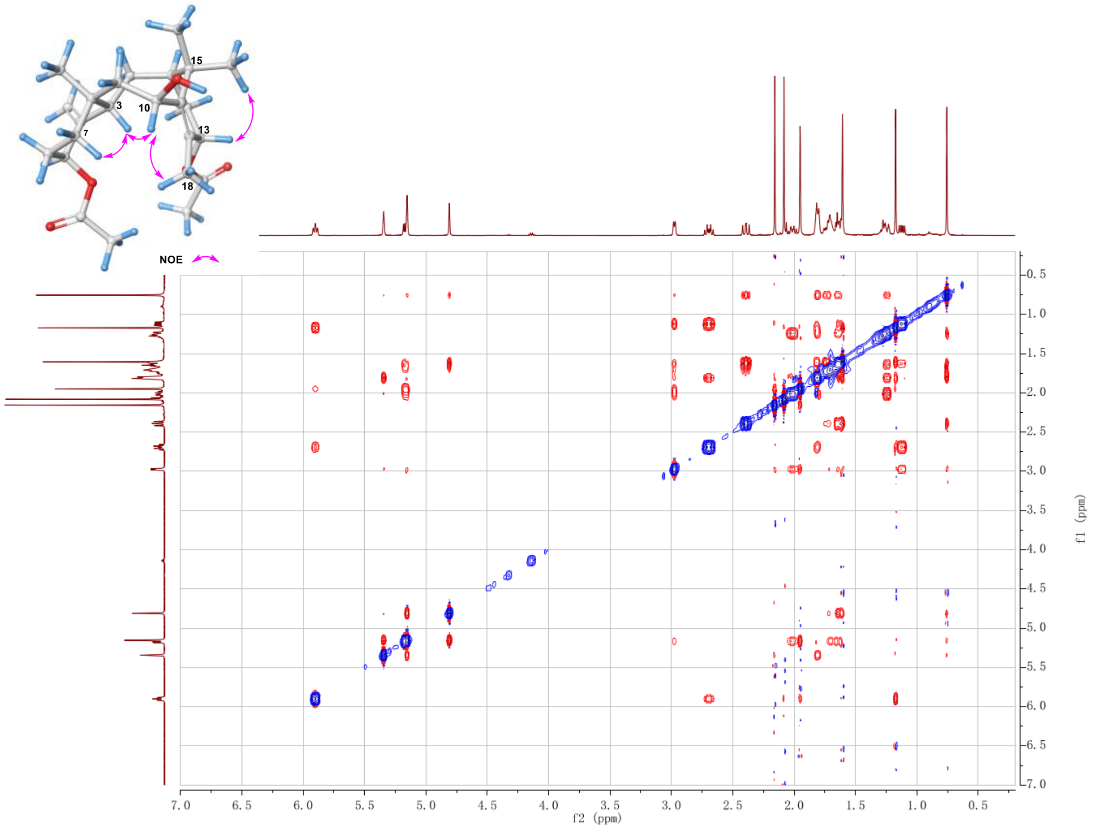

Supplementary Figure 56. 2D-NOE NMR spectrum of 5α,13α-diacetoxytaxa-4(20),11(12)-diene-10β-ol (10).

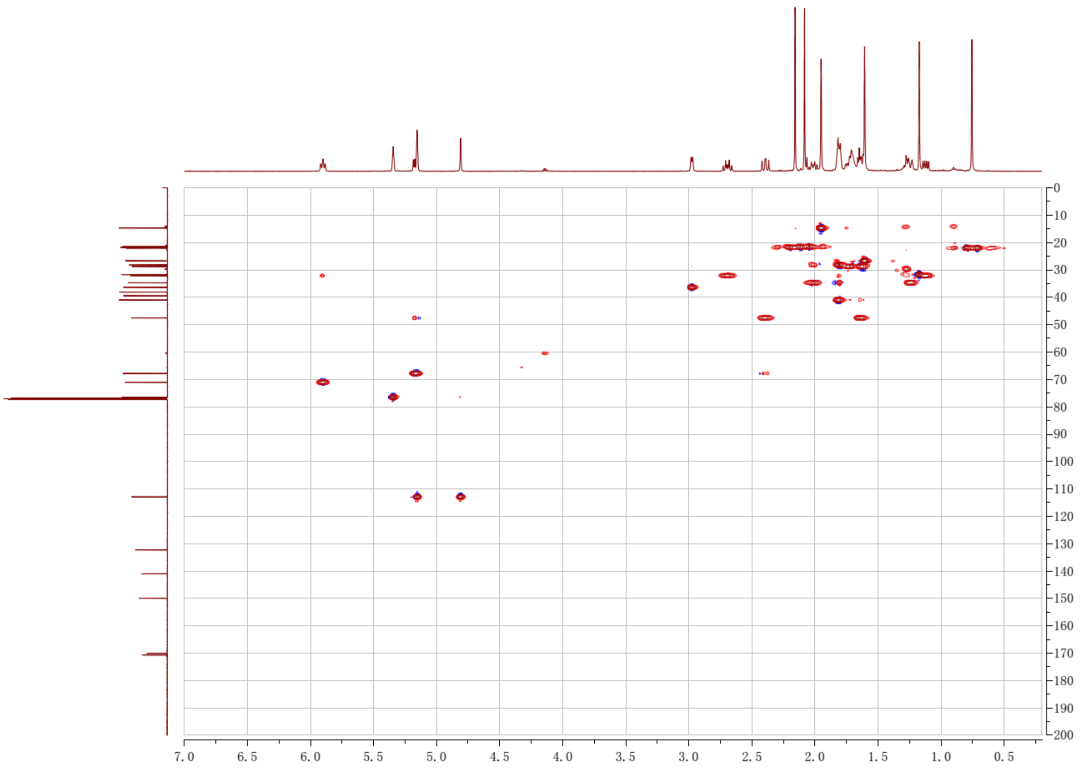

Supplementary Figure 57. 2D-HSQC NMR spectrum of 5α,13α-diacetoxytaxa-4(20),11(12)-diene-10β-ol (10).

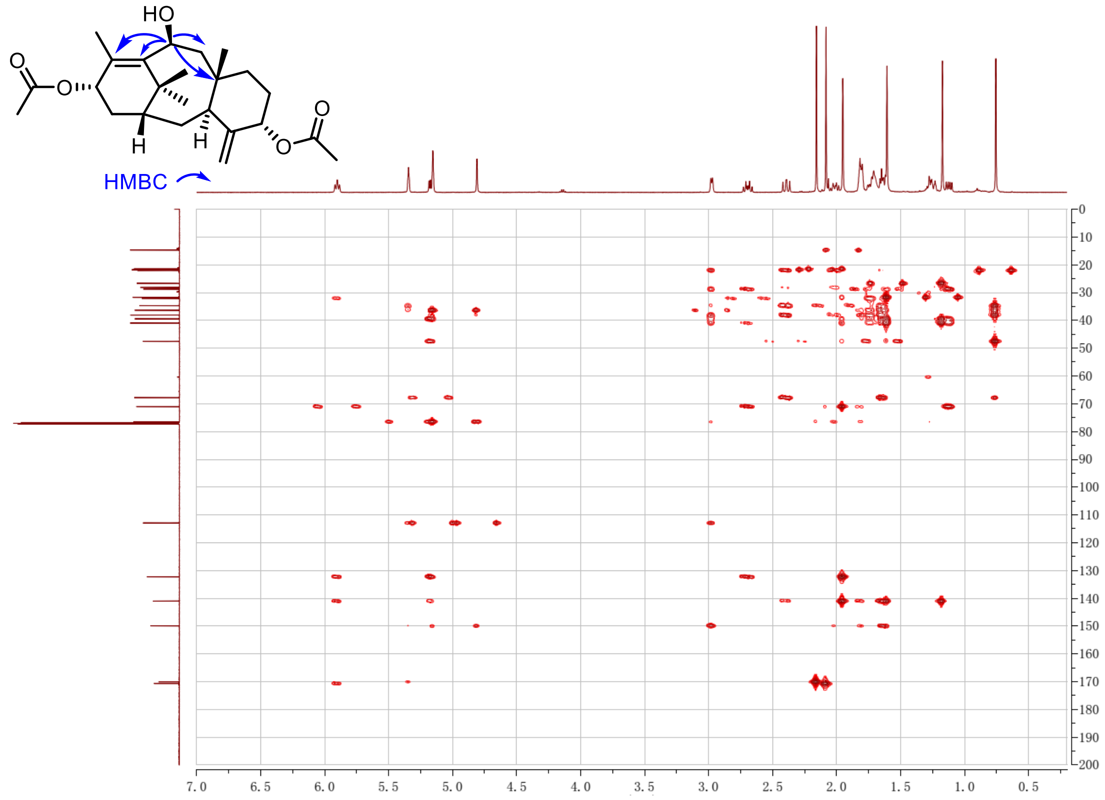

Supplementary Figure 58. 2D-HMBC NMR spectrum of 5α,13α-diacetoxytaxa-4(20),11(12)-diene-10β-ol (10).

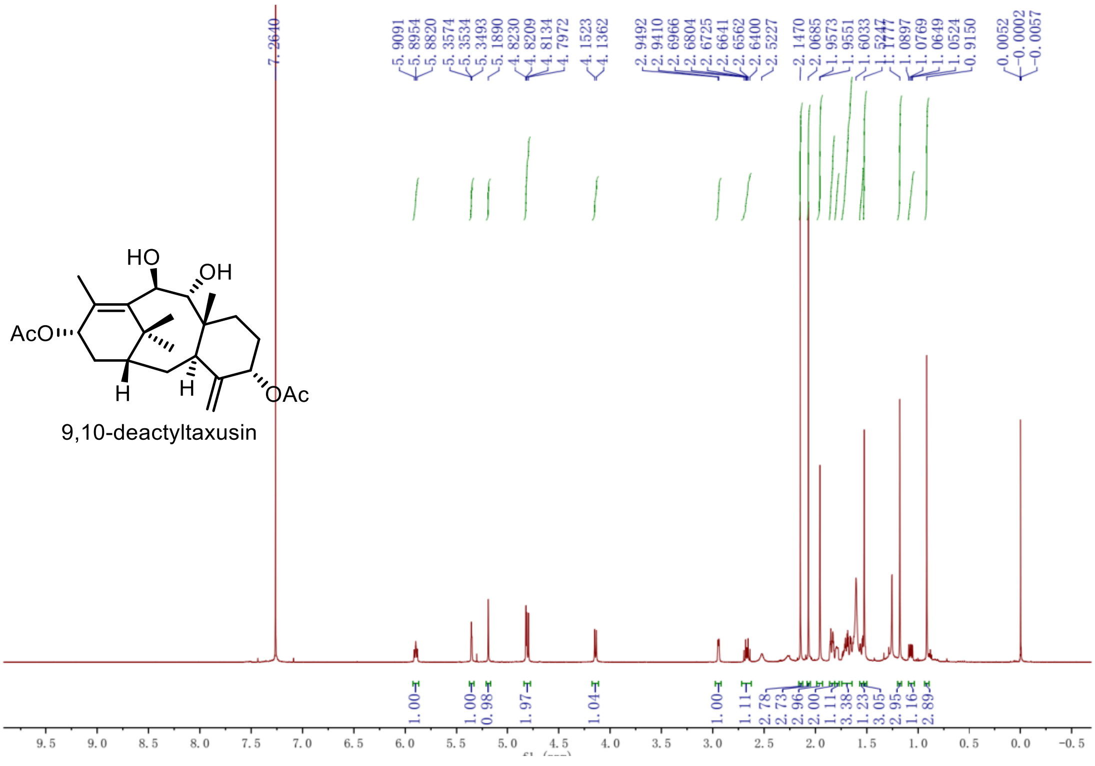

Supplementary Figure 59. ^{1}H NMR spectrum of 9,10-deactyltaxusin (11).

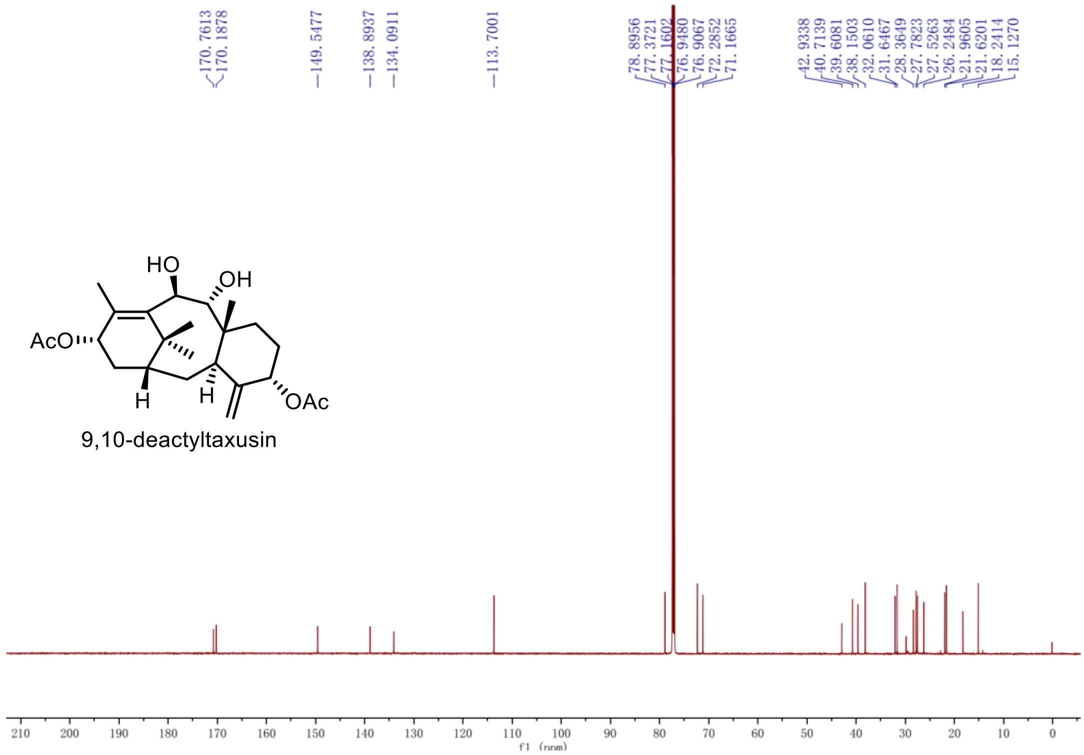

Supplementary Figure 60. ^{13}C NMR spectrum of 9,10-deactyltaxusin (11).

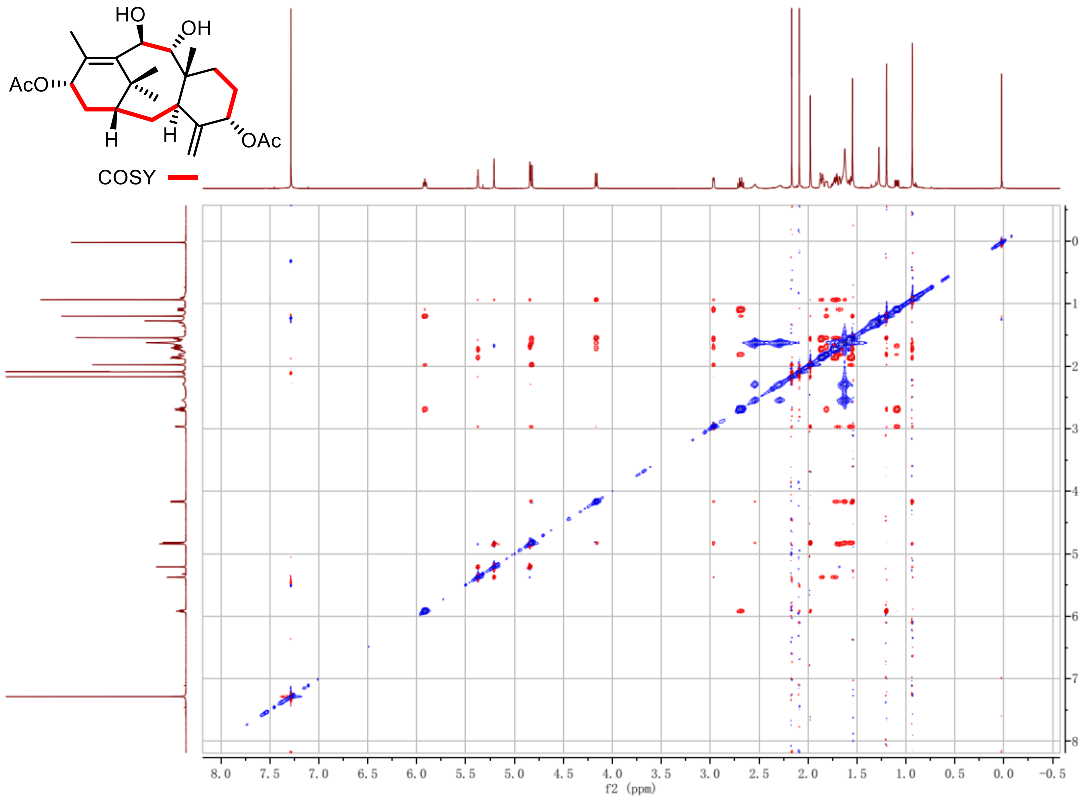

Supplementary Figure 61. 2D-COSY NMR spectrum of 9,10-deactyltaxusin (11).

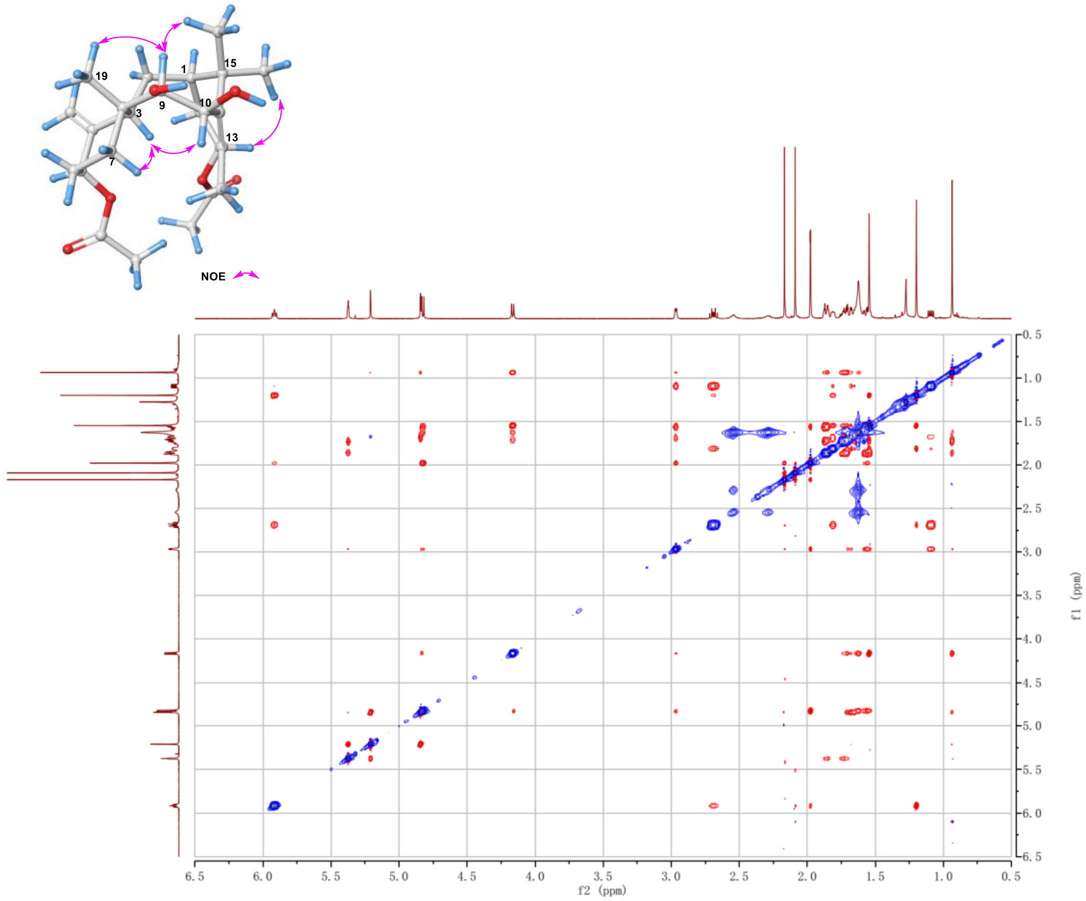

Supplementary Figure 62. 2D-NOE NMR spectrum of 9,10-deactyltaxusin (11).

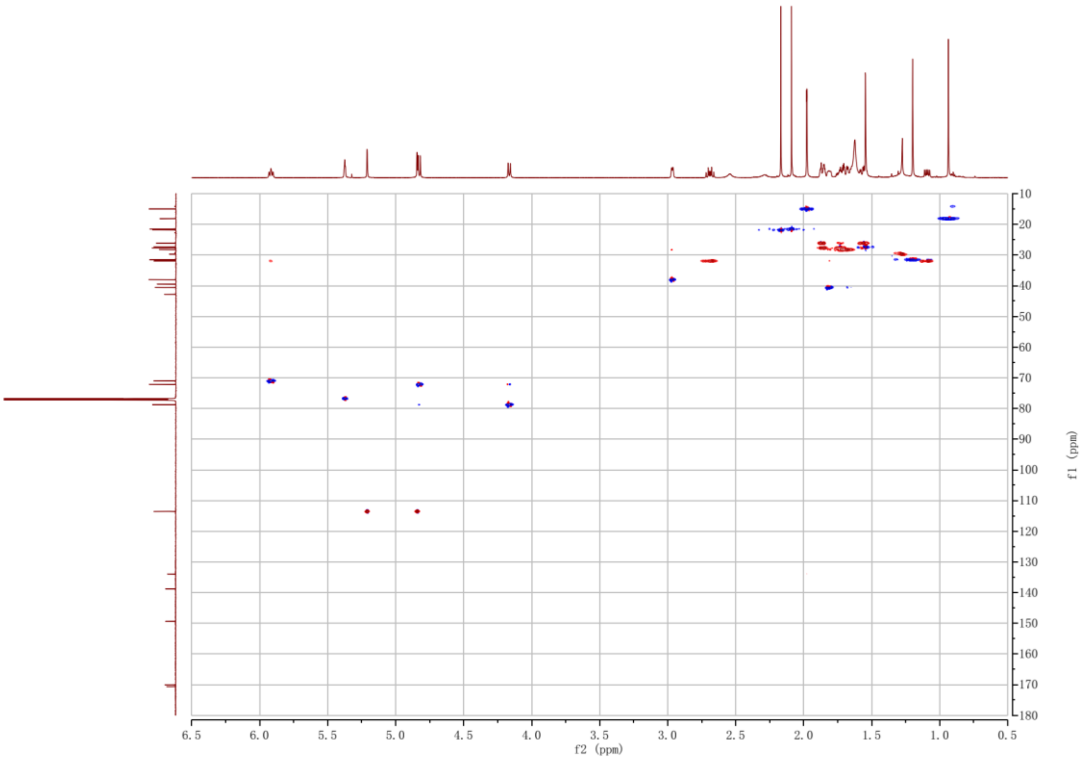

Supplementary Figure 63. 2D-HSQC NMR spectrum of 9,10-deactyltaxusin (11).

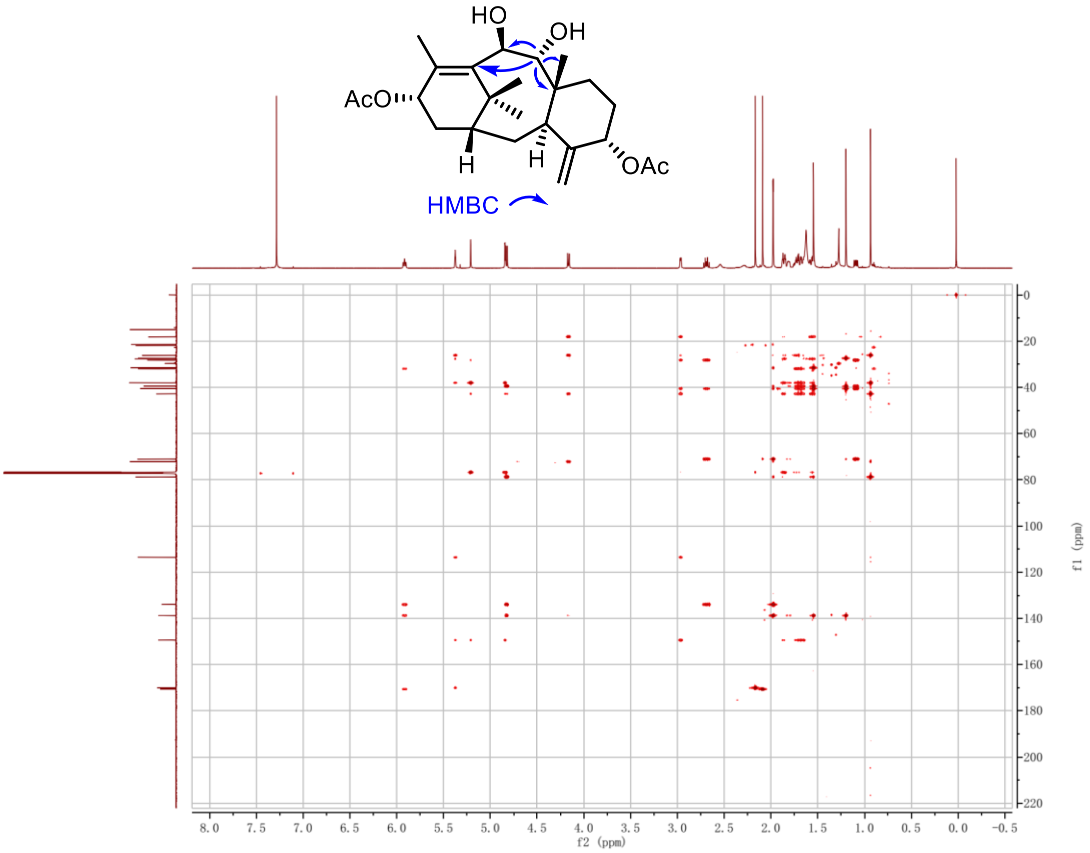

Supplementary Figure 64. 2D-HMBC NMR spectrum of 9,10-deactyltaxusin (11).

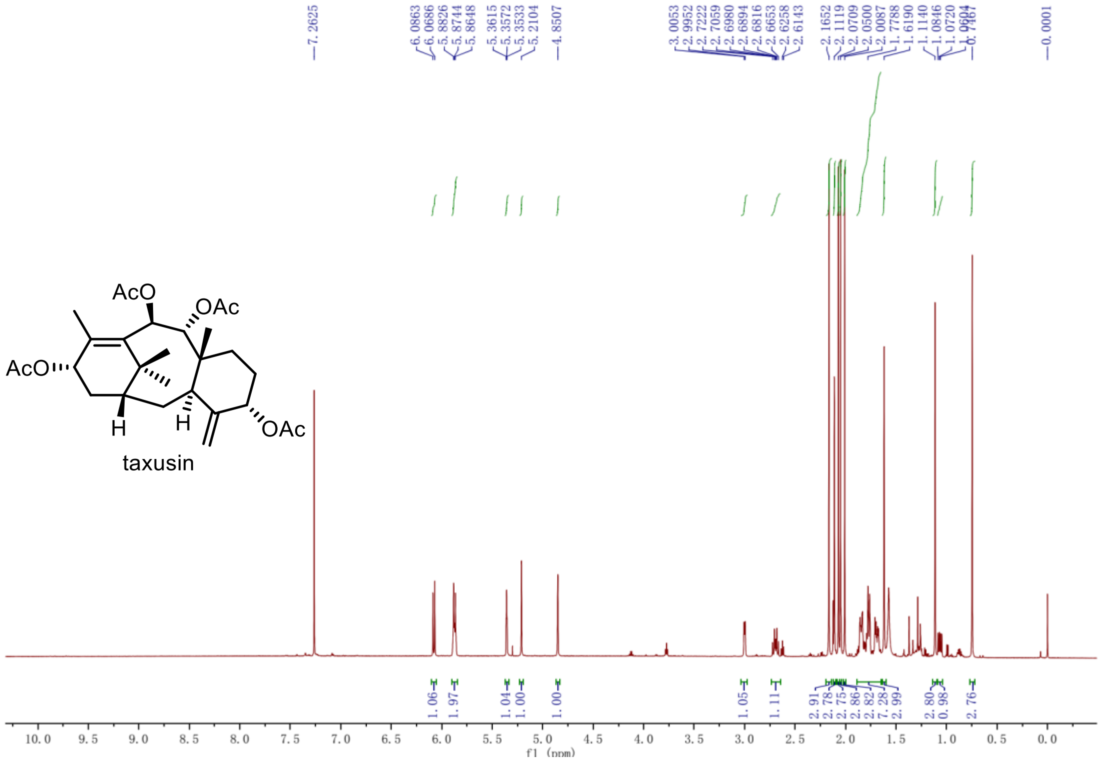

Supplementary Figure 65. ^{1}H NMR spectrum of taxusin (12).

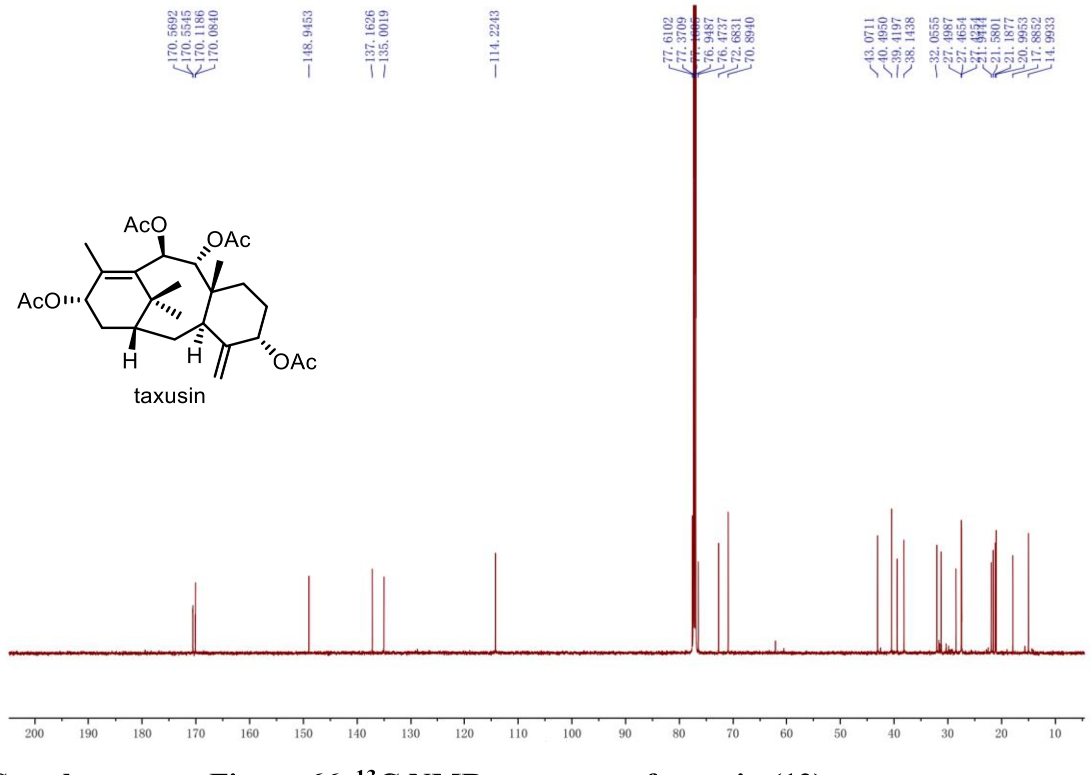

Supplementary Figure 66. ^{13}C NMR spectrum of taxusin (12).

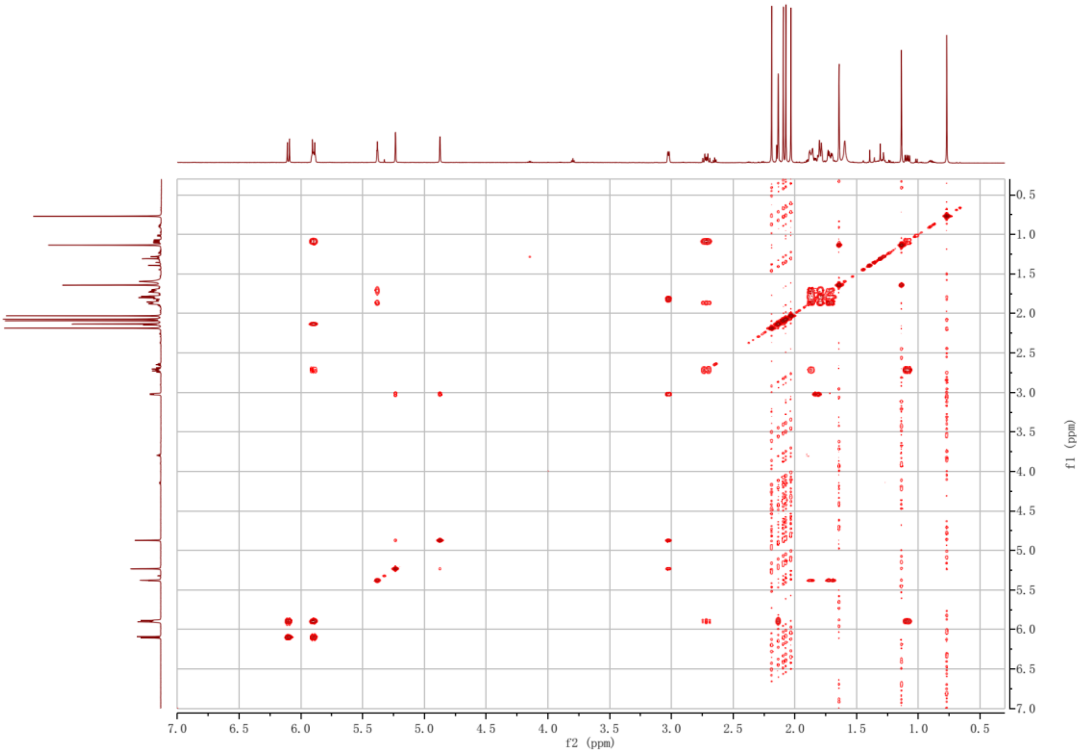

Supplementary Figure 67. 2D-COSY NMR spectrum of taxusin (12).

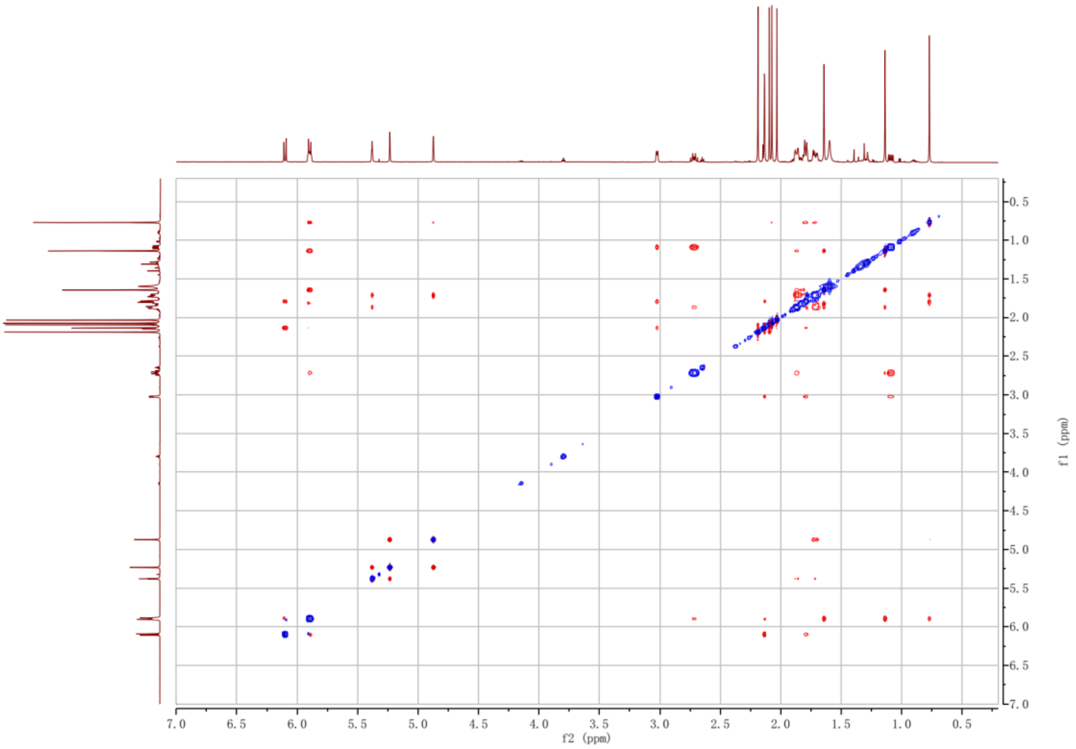

Supplementary Figure 68. 2D-NOE NMR spectrum of taxusin (12).

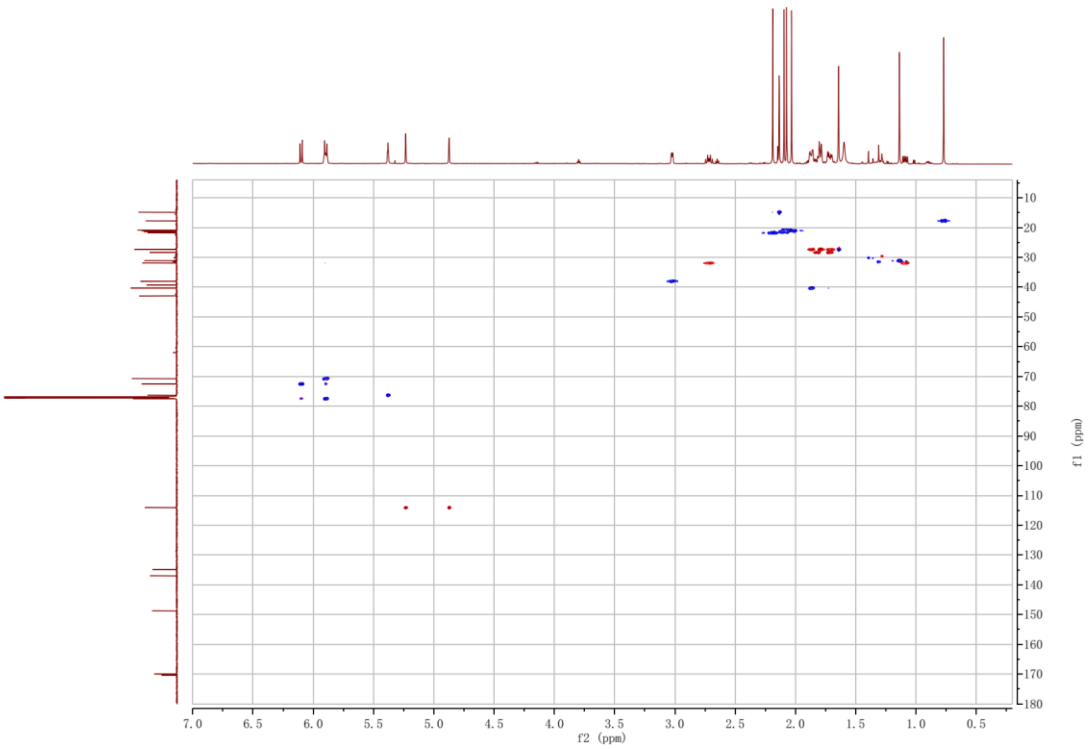

Supplementary Figure 69. 2D-HSQC NMR spectrum of taxusin (12).

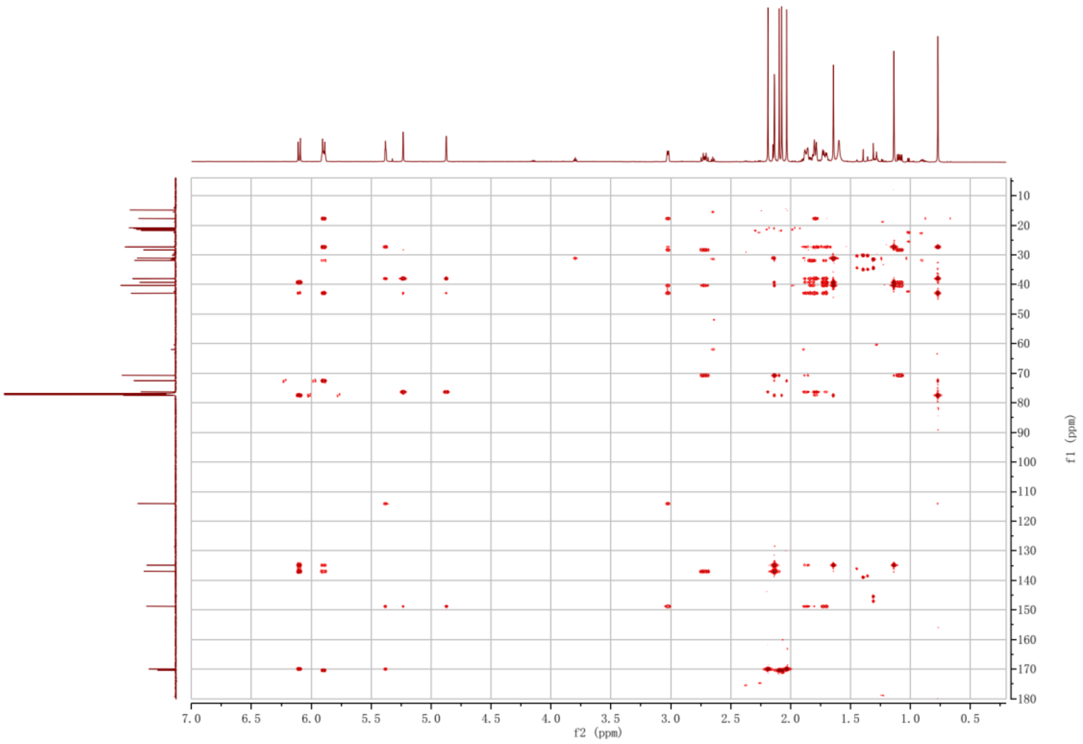

Supplementary Figure 70. 2D-HMBC NMR spectrum of taxusin (12).

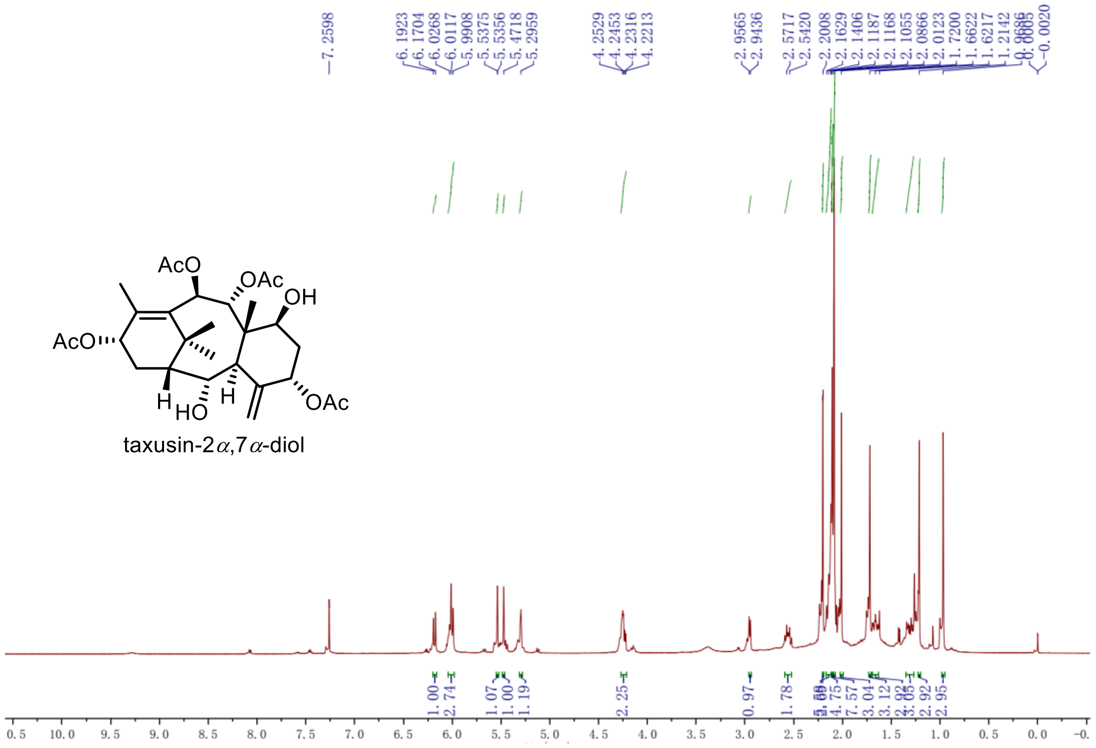

Supplementary Figure 71. ^{1}H NMR spectrum of 2α,7β-dihydroxytaxusin (13).

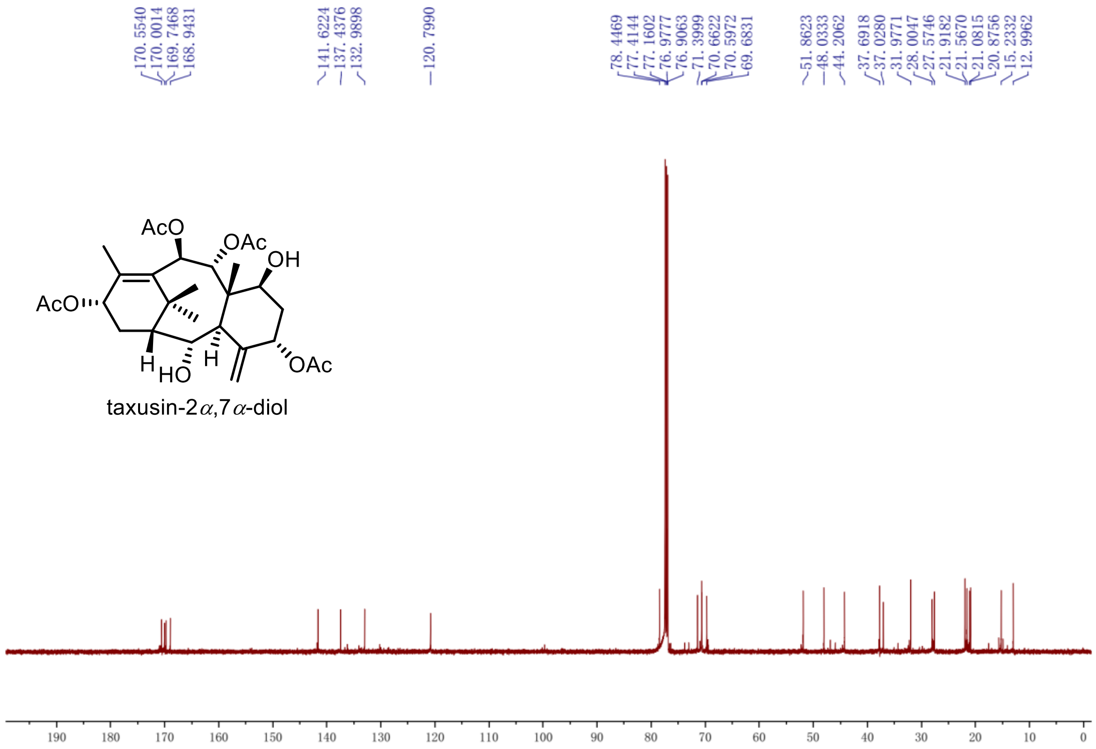

Supplementary Figure 72. ^{13}C NMR spectrum of 2α,7β-dihydroxytaxusin (13).

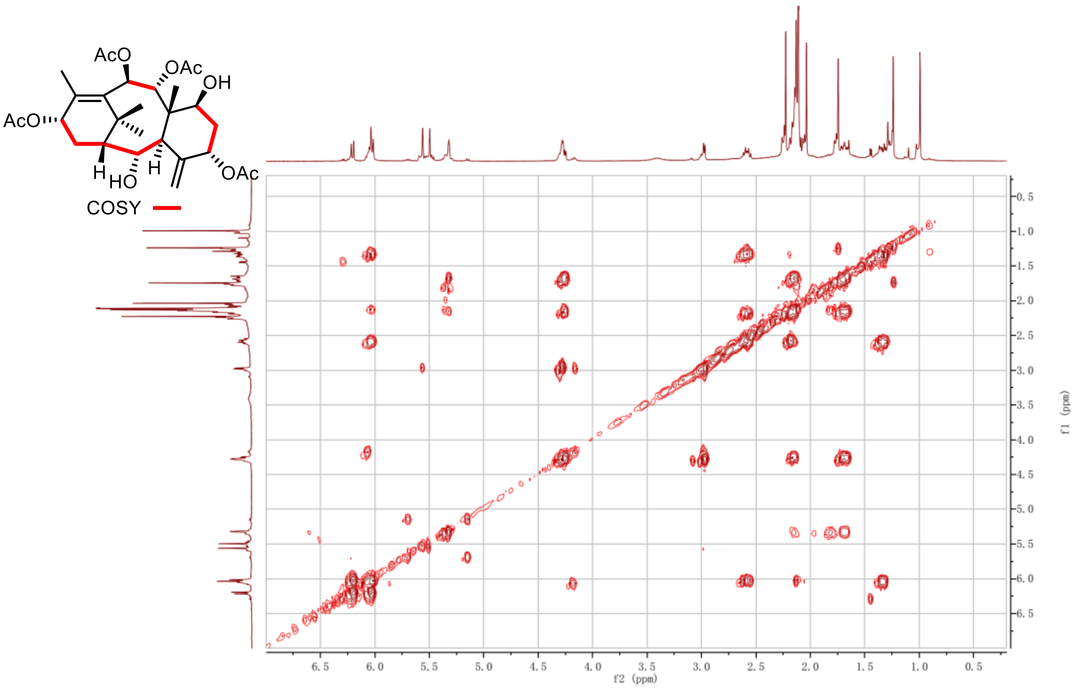

Supplementary Figure 73. 2D-COSY NMR spectrum of 2*α*,7*β*-dihydroxytaxusin (13).

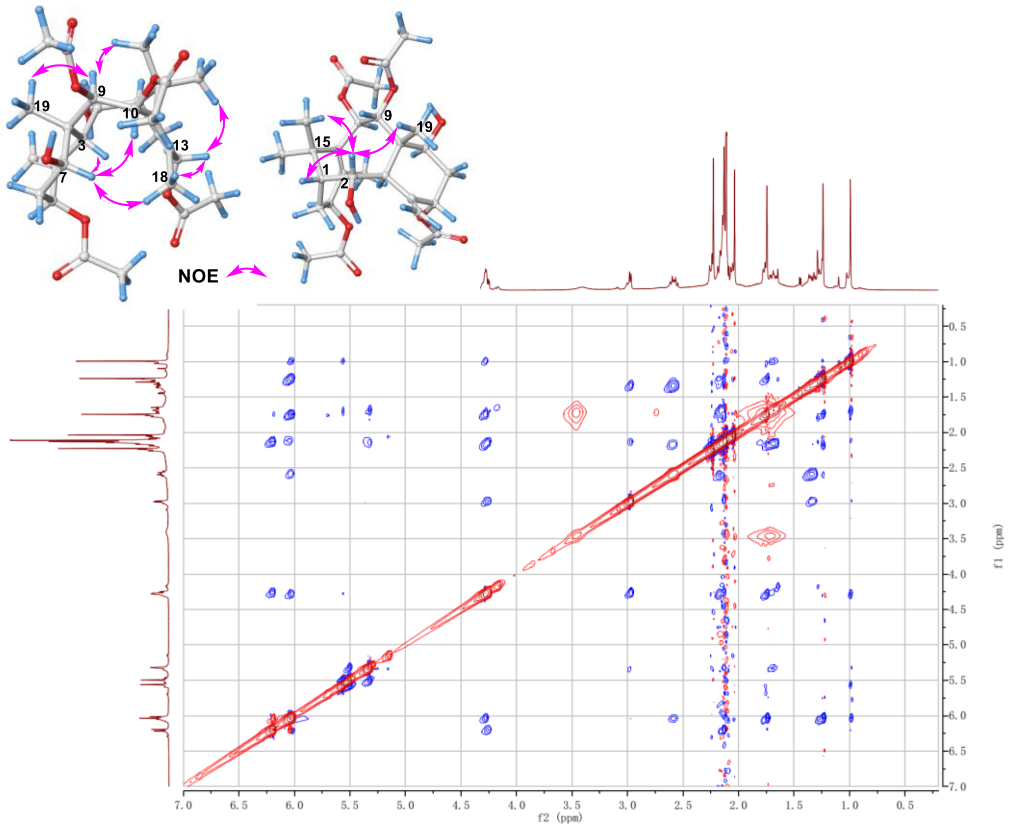

Supplementary Figure 74. 2D-NOE NMR spectrum of 2*α*,7*β*-dihydroxytaxusin (13).

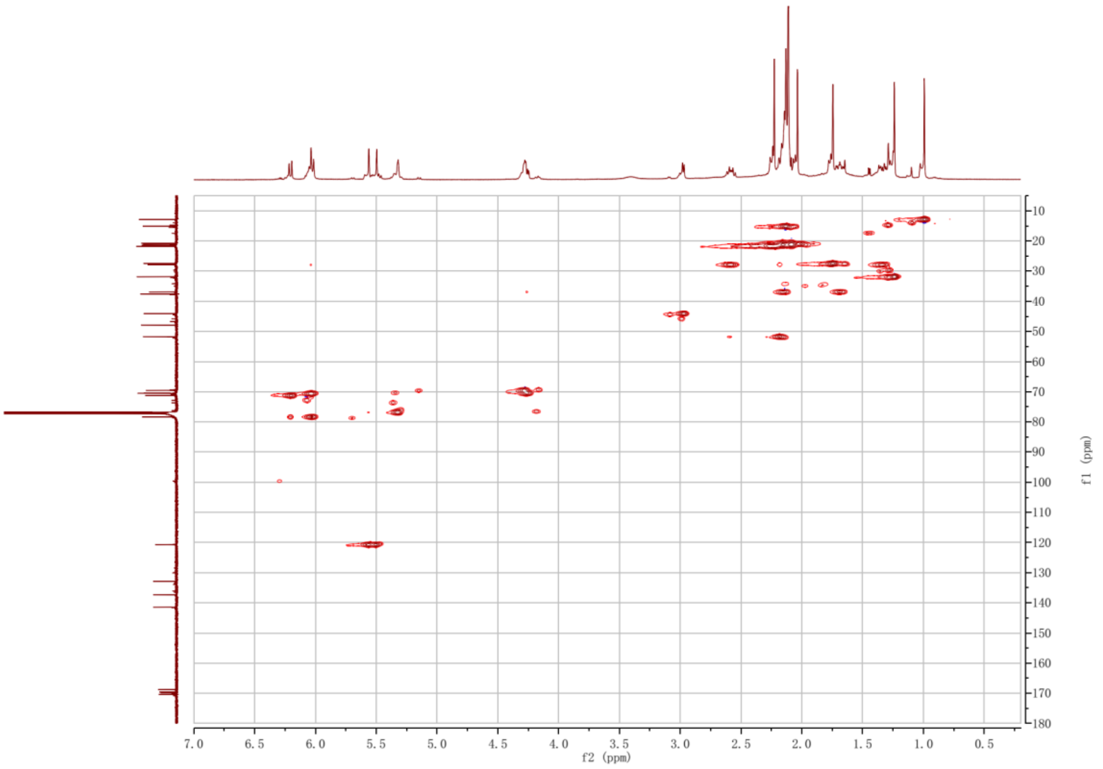

Supplementary Figure 75. 2D-HSQC NMR spectrum of 2*α*,7*β*-dihydroxytaxusin (13).

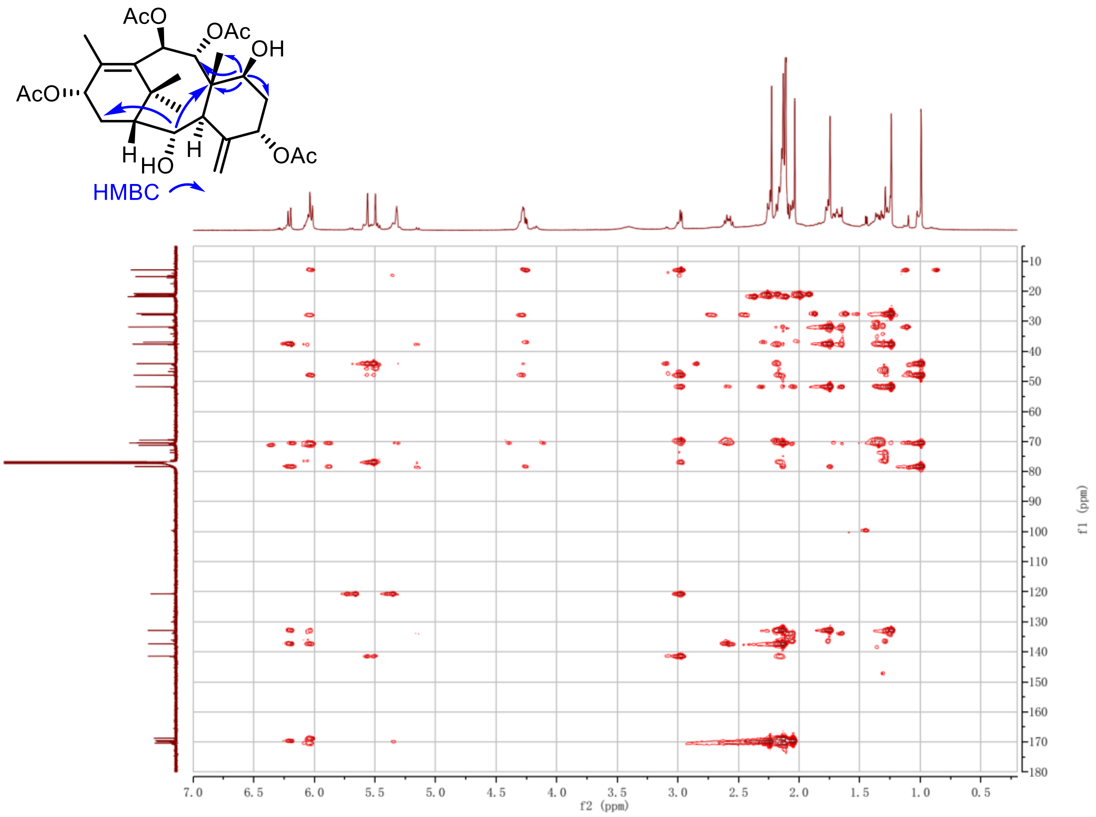

Supplementary Figure 76. 2D-HMBC NMR spectrum of 2*α*,7*β*-dihydroxytaxusin (13).

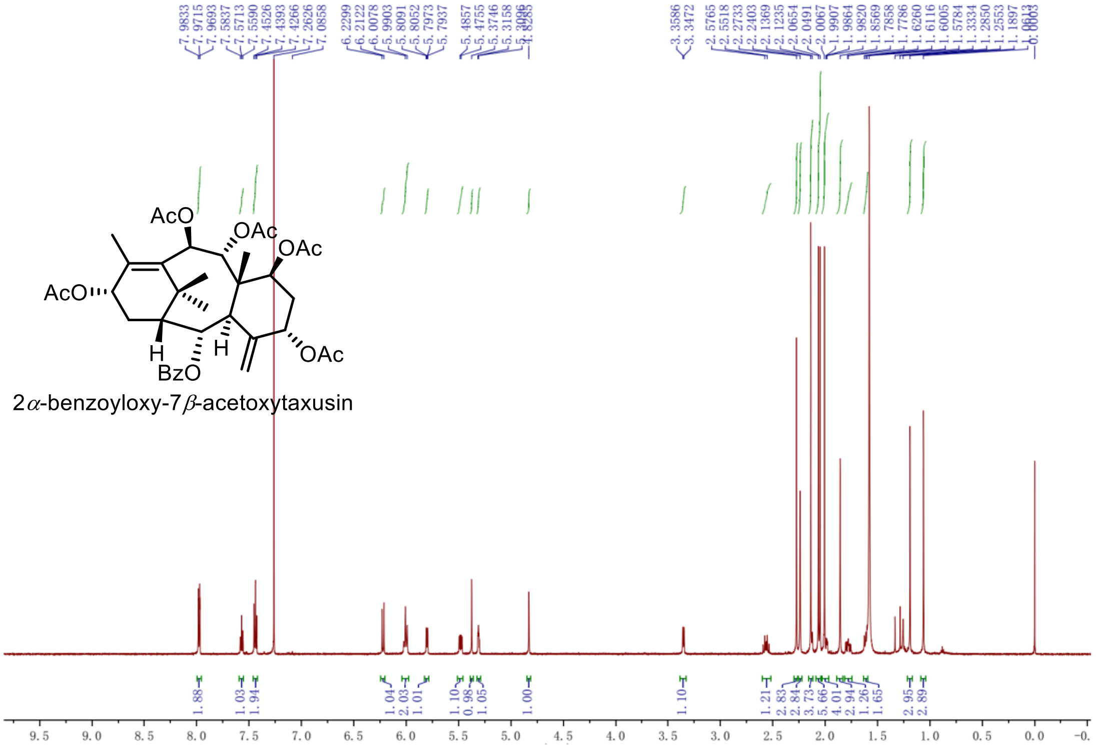

Supplementary Figure 77. ^{1}H NMR spectrum of 2α-benzoyloxy-7β-acetoxytaxusin (15).

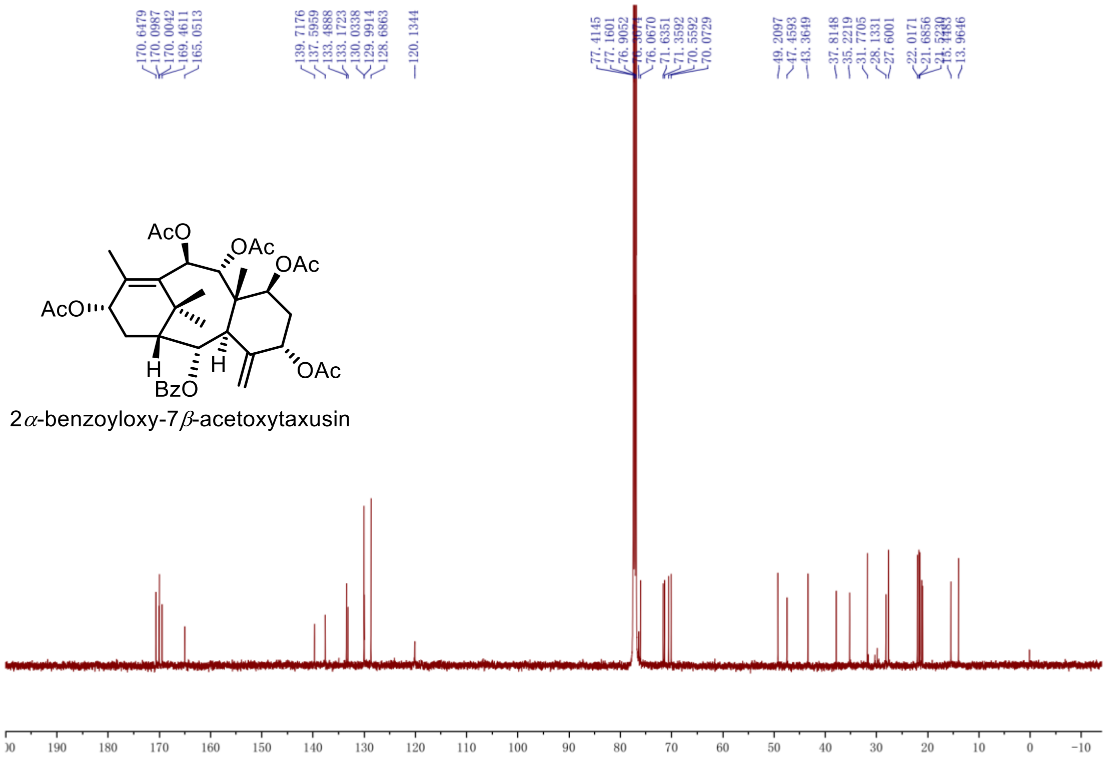

Supplementary Figure 78. ^{13}C NMR spectrum of 2α-benzoyloxy-7β-acetoxytaxusin (15).

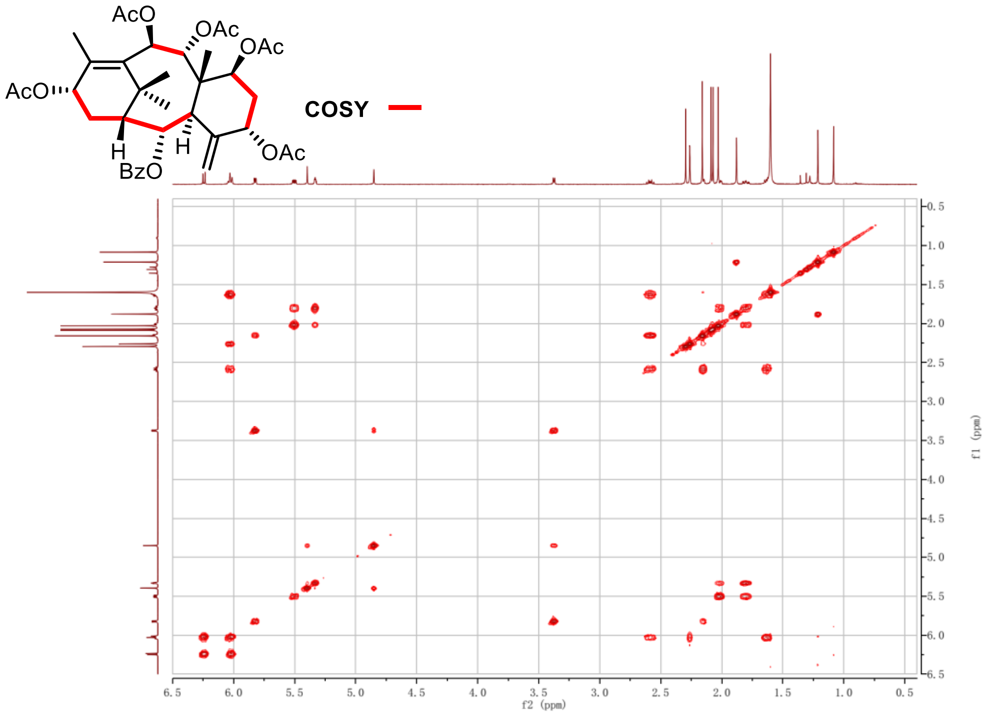

Supplementary Figure 79. 2D-COSY NMR spectrum of 2*α*-benzoyloxy-7*β*-acetoxytaxusin (15).

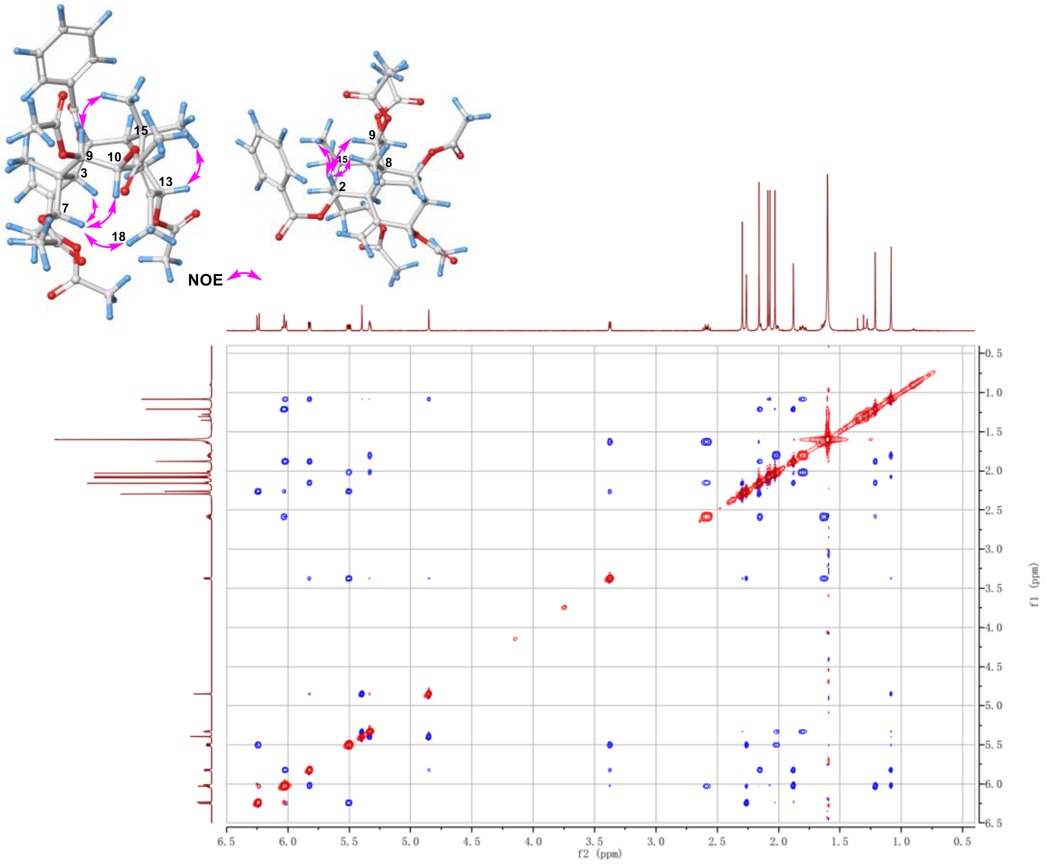

Supplementary Figure 80. 2D-NOE NMR spectrum of 2*α*-benzoyloxy-7*β*-acetoxytaxusin (15).

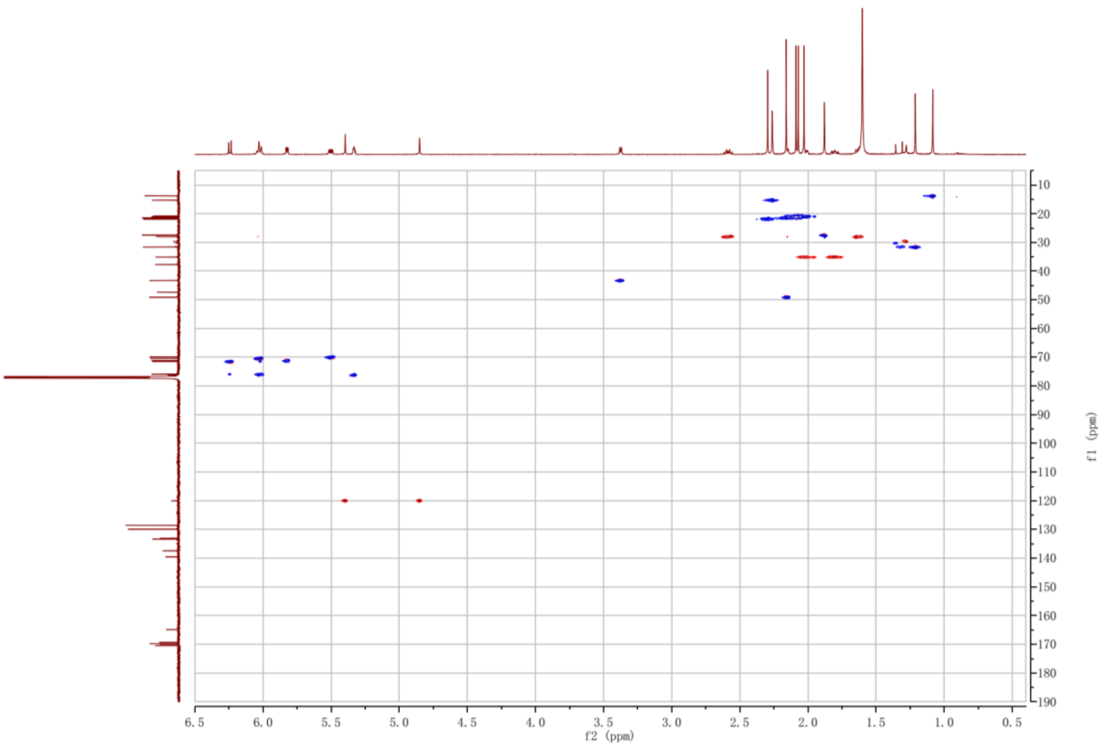

Supplementary Figure 81. 2D-HSQC NMR spectrum of 2*α*-benzoyloxy-7*β*-acetoxytaxusin (15).

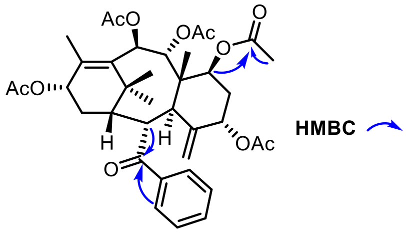

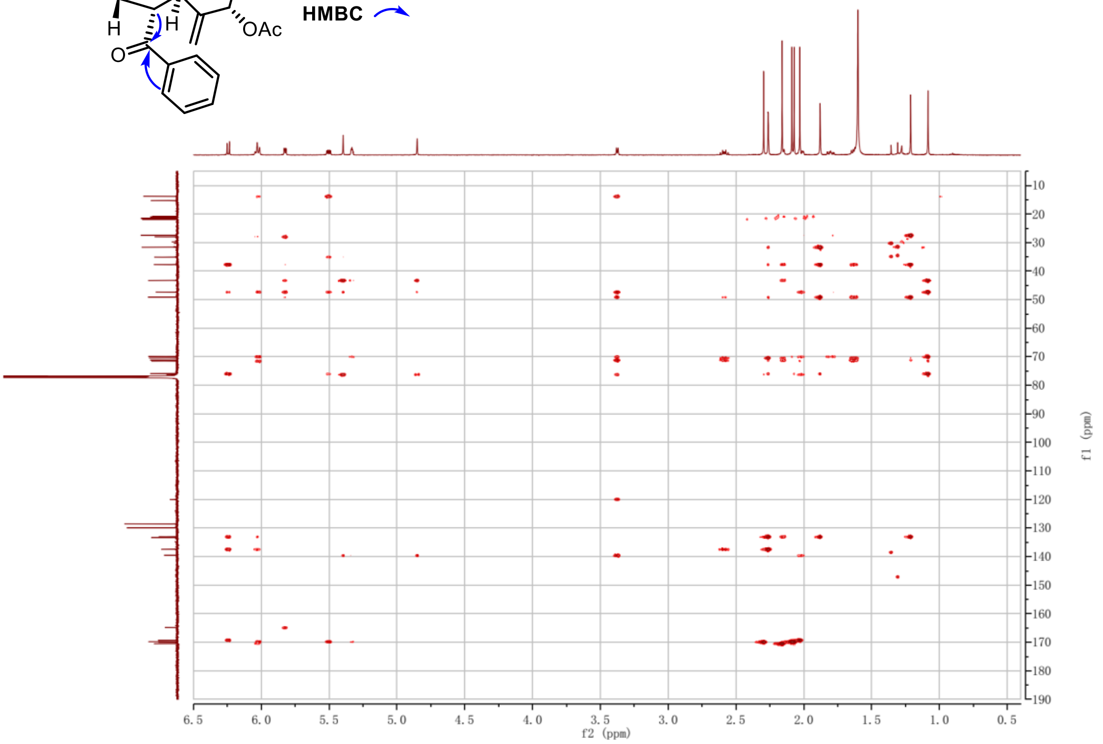

Supplementary Figure 82. 2D-HMBC NMR spectrum of 2*α*-benzoyloxy-7*β*-acetoxytaxusin (15).

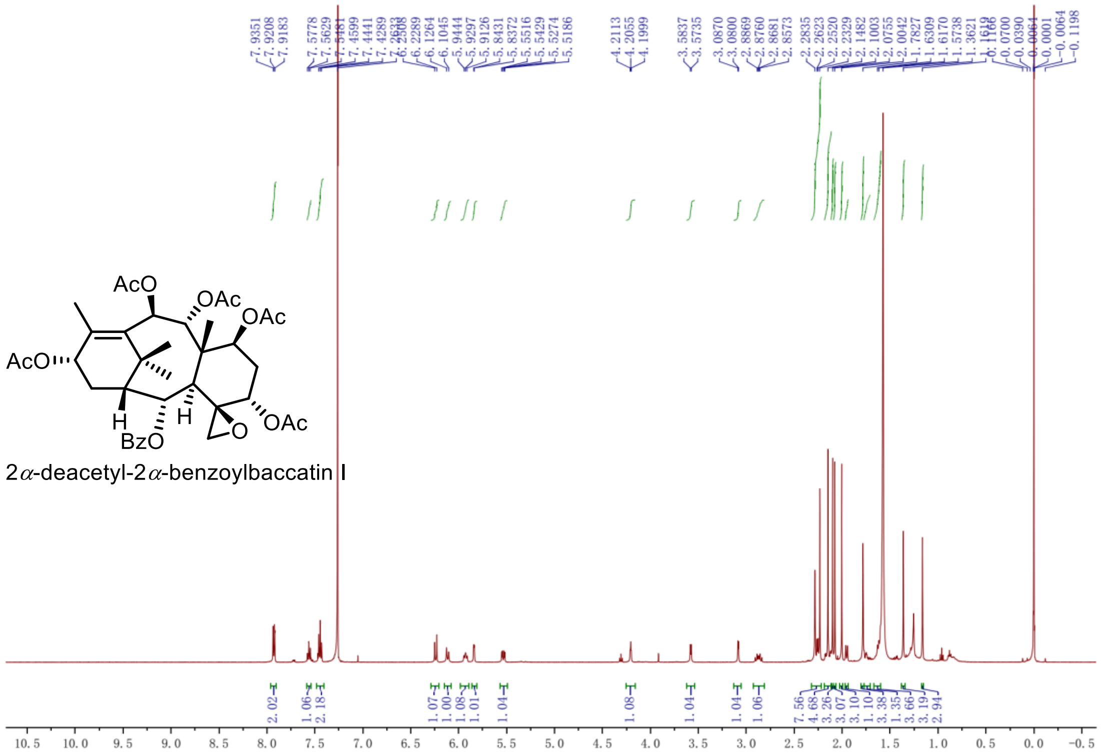

Supplementary Figure 83. ^{1}H NMR spectrum of 2*α*-deacetyl-2*α*-benzoylbaccatin I (17).

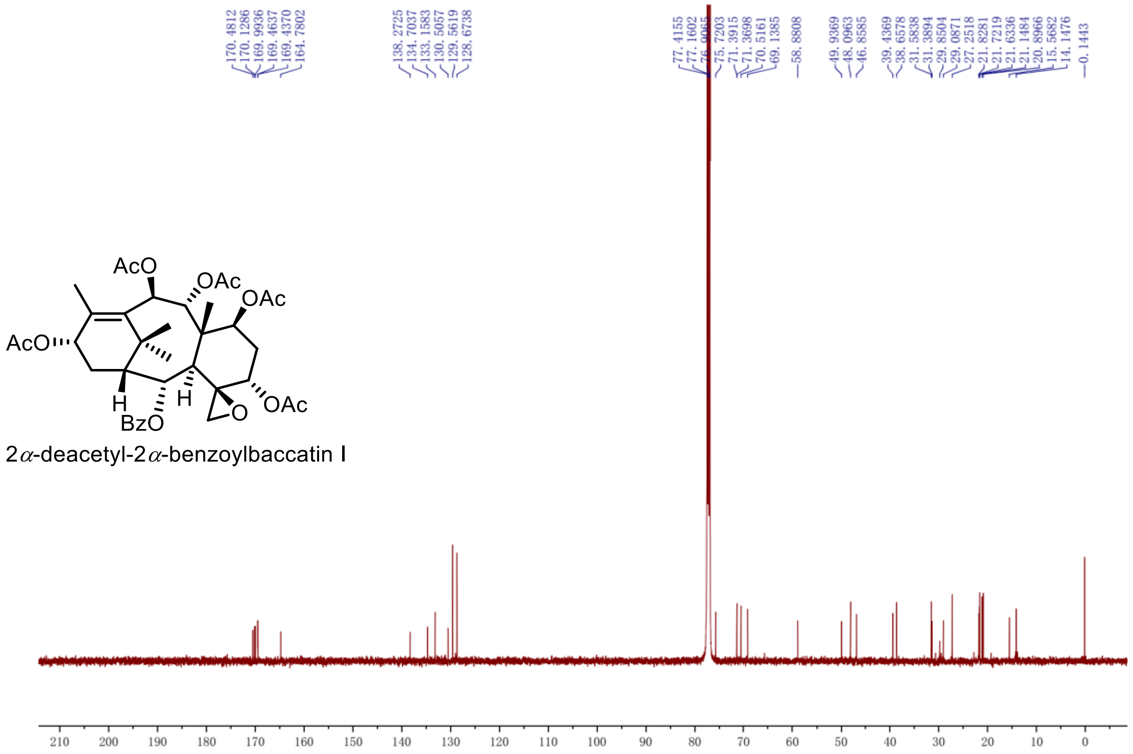

Supplementary Figure 84. ^{13}C NMR spectrum of 2*α*-deacetyl-2*α*-benzoylbaccatin I (17).

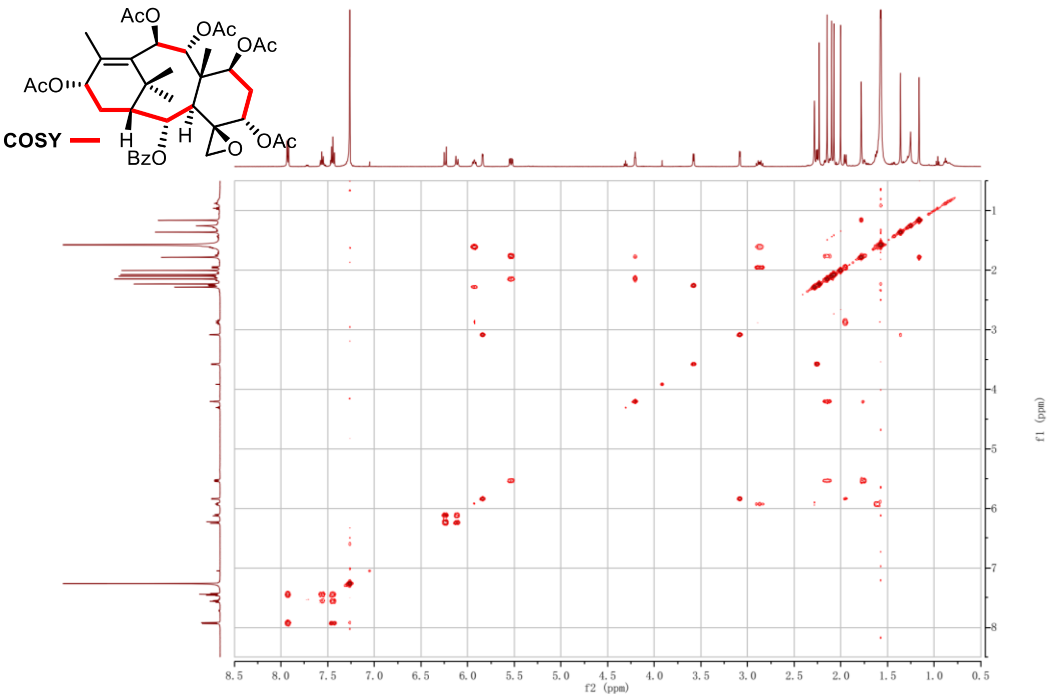

Supplementary Figure 85. 2D-COSY NMR spectrum of 2α-deacetyl-2α-benzoylbaccatin I (17).

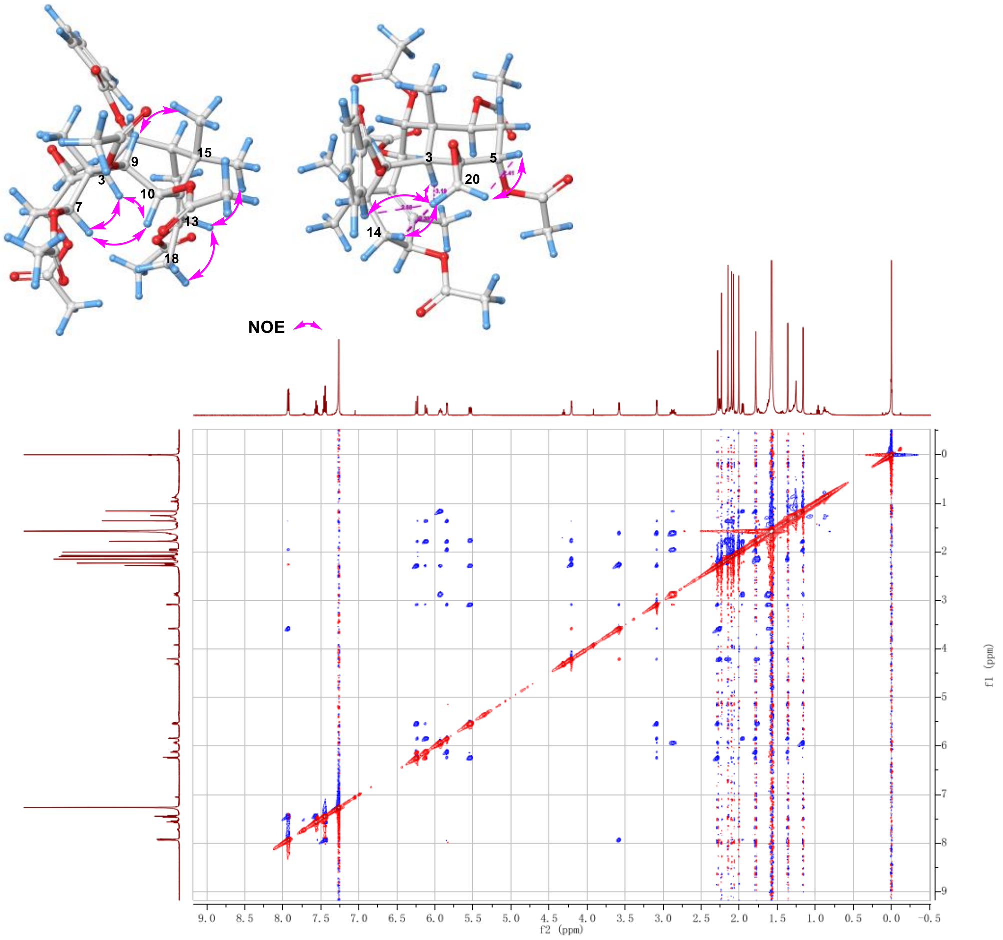

Supplementary Figure 86. 2D-NOE NMR spectrum of 2α-deacetyl-2α-benzoylbaccatin I (17).

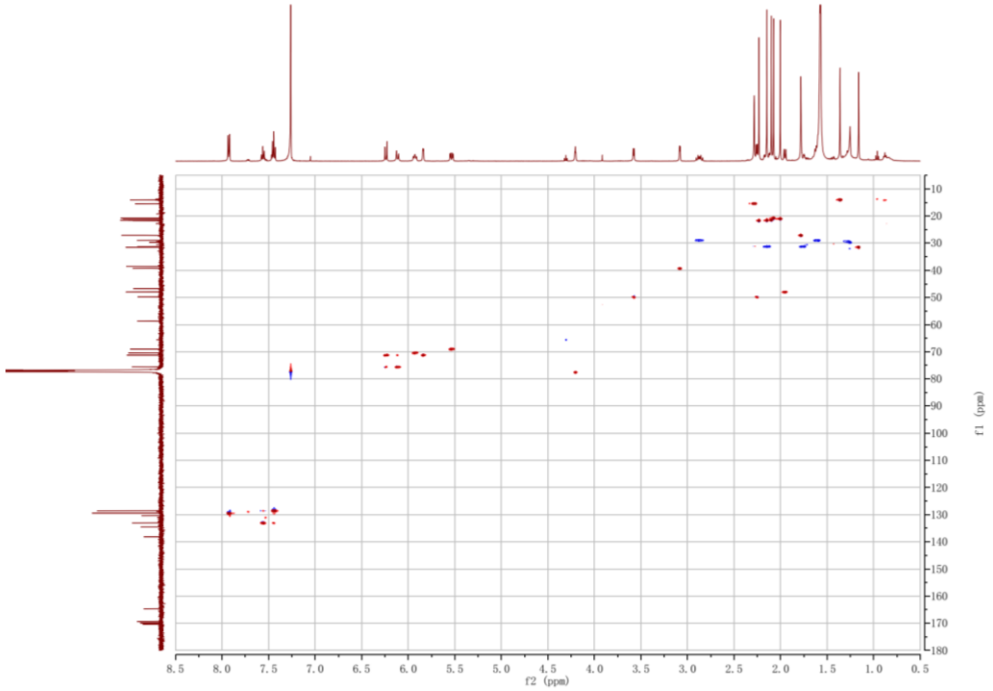

Supplementary Figure 87. 2D-HSQC NMR spectrum of 2α-deacetyl-2α-benzoylbaccatin I (17).

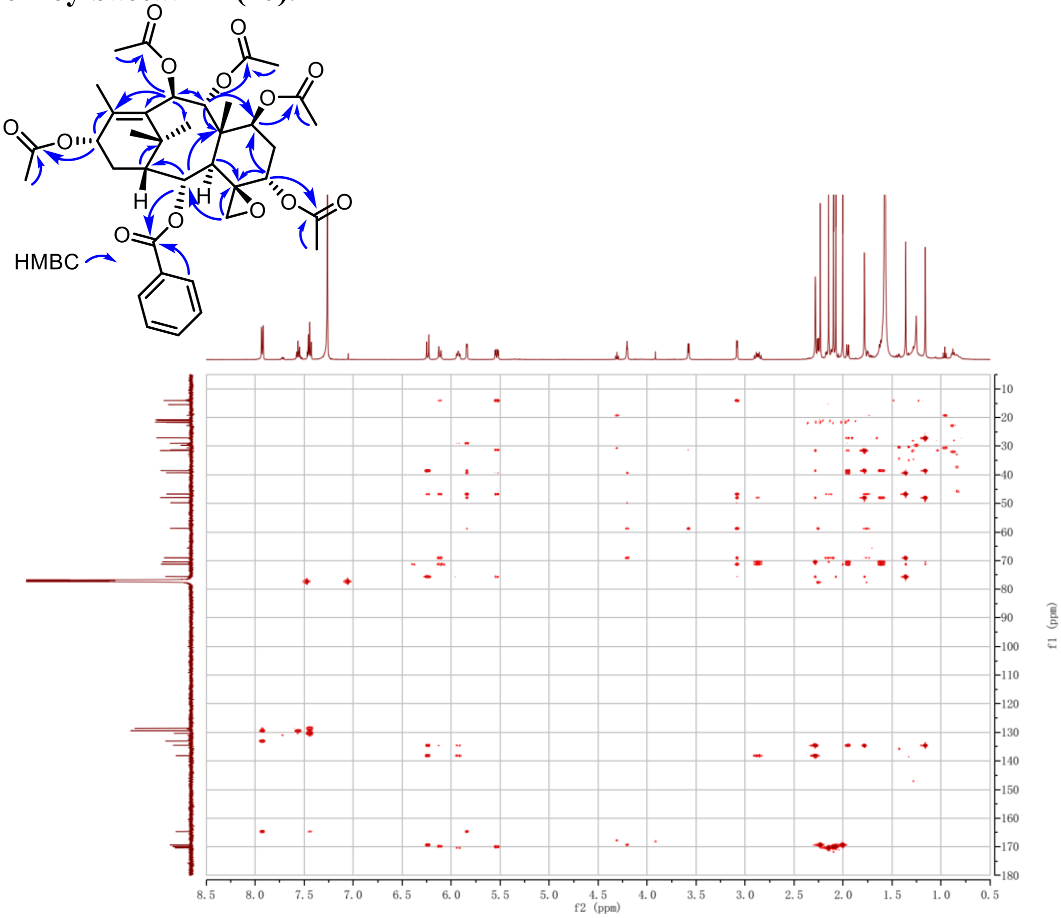

Supplementary Figure 88. 2D-HMBC NMR spectrum of 2α-deacetyl-2α-benzoylbaccatin I (17).

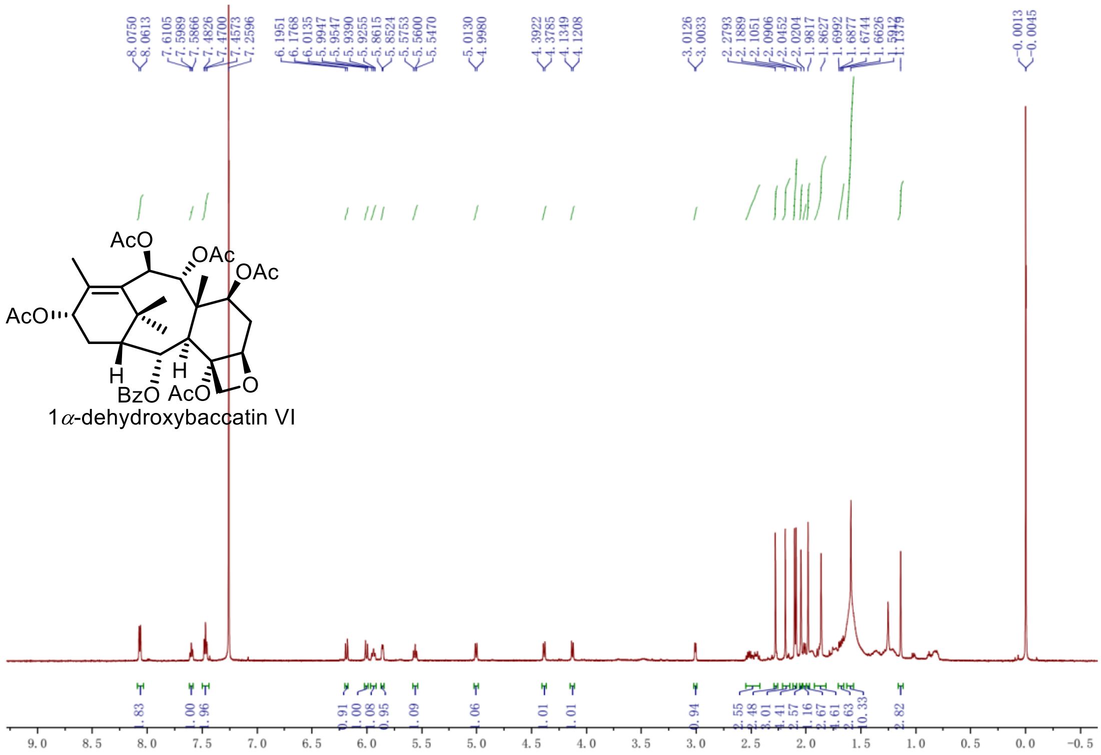

Supplementary Figure 89. ^{1}H NMR spectrum of 1*β*-dehydroxybaccatin VI (16).

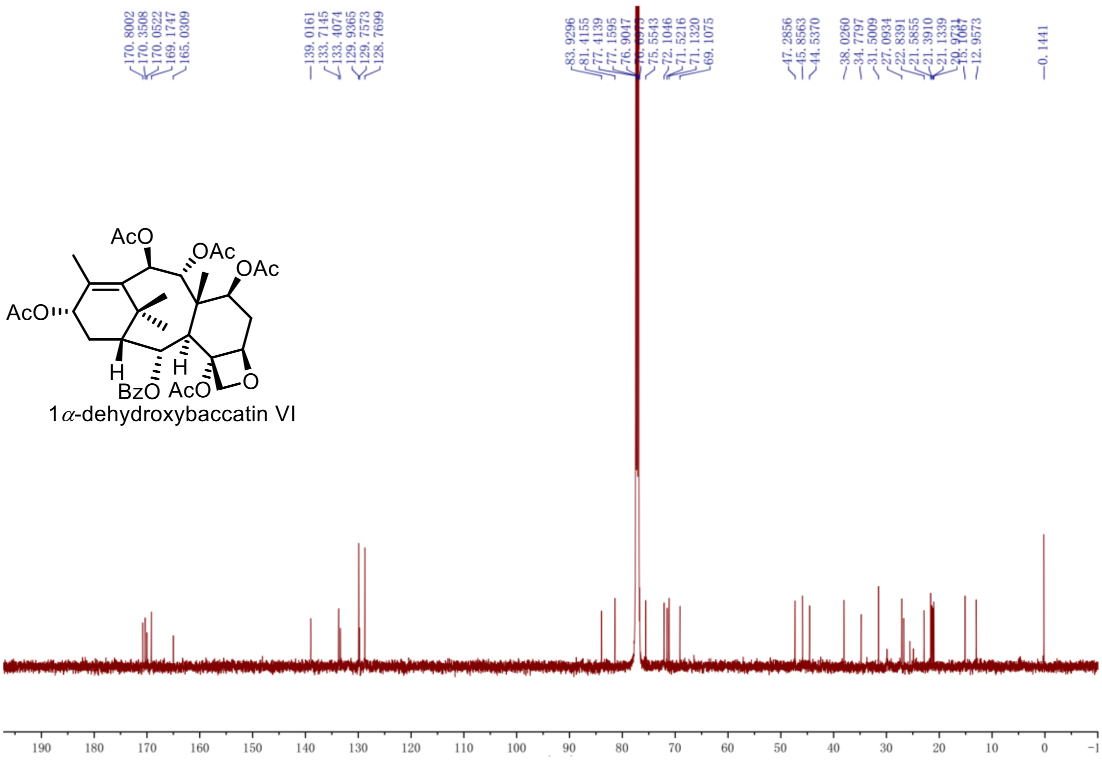

Supplementary Figure 90. ^{13}C NMR spectrum of 1*β*-dehydroxybaccatin VI (16).

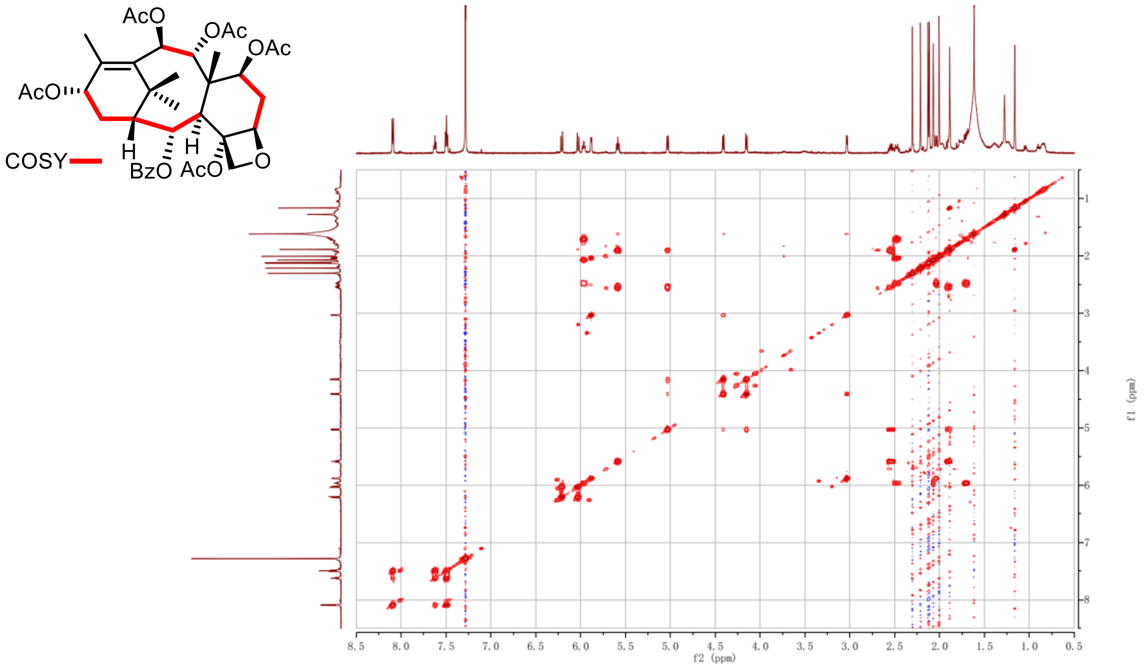

Supplementary Figure 91. 2D-COSY NMR spectrum of 1β-dehydroxybaccatin VI (16).

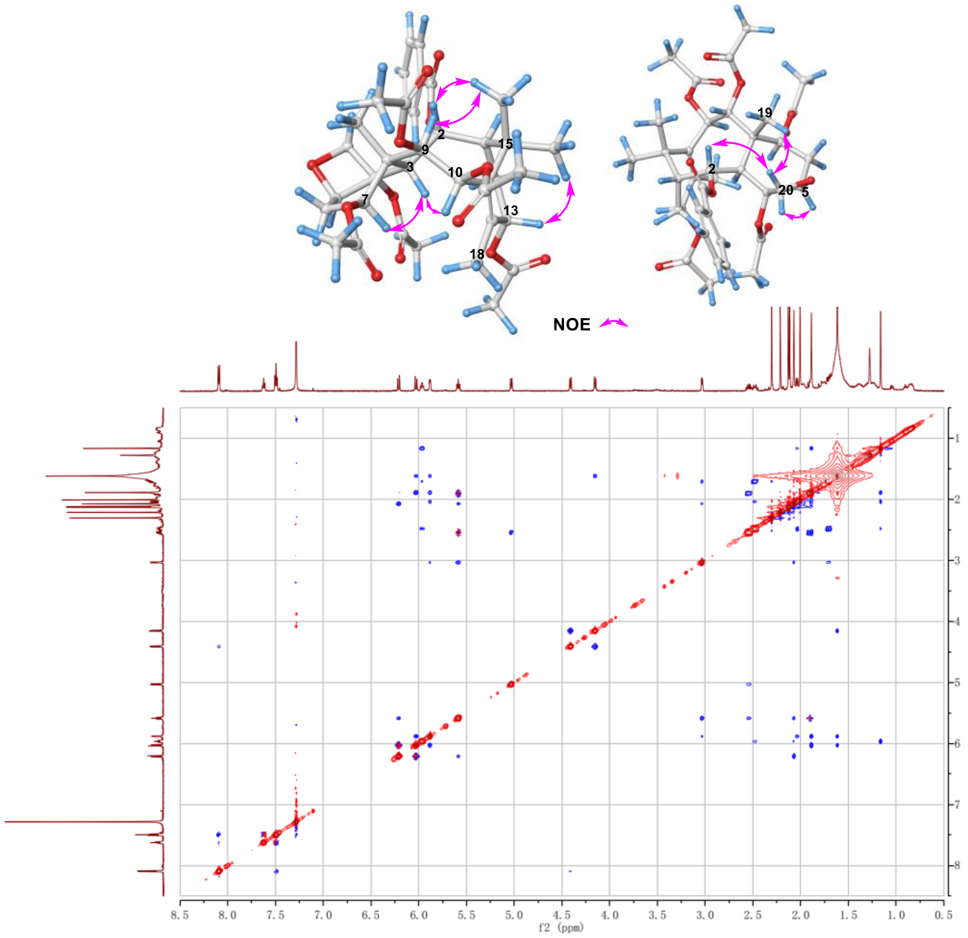

Supplementary Figure 92. 2D-NOE NMR spectrum of 1β-dehydroxybaccatin VI (16).

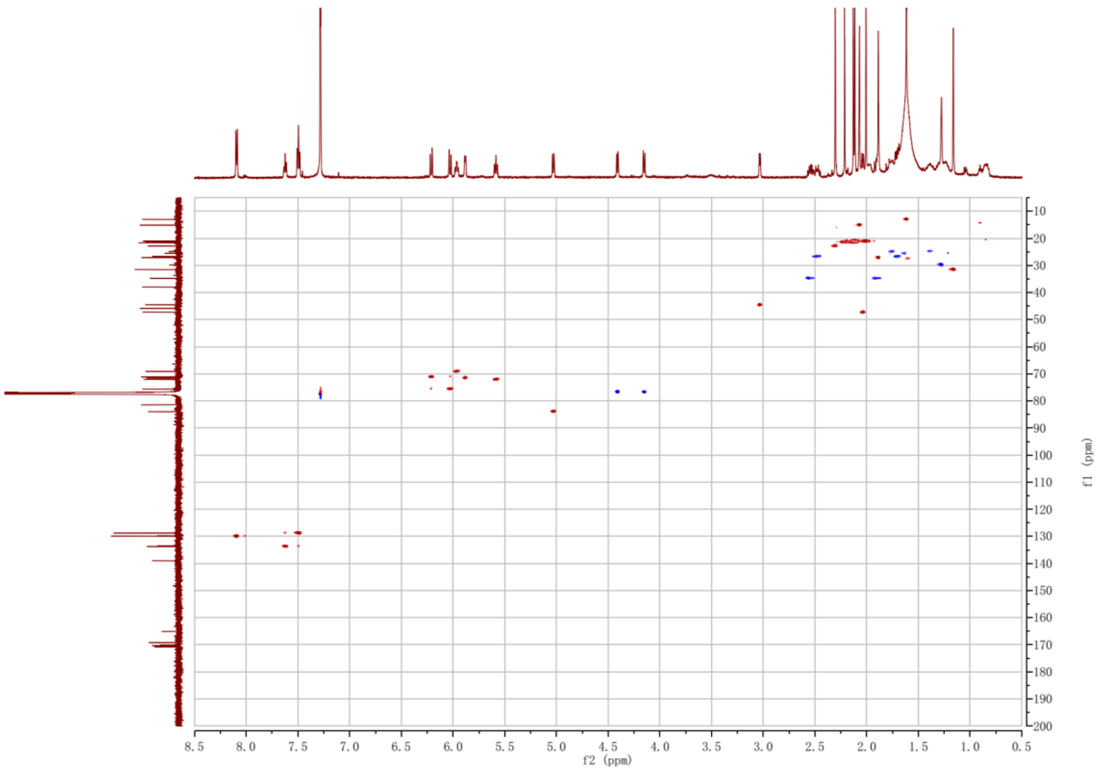

Supplementary Figure 93. 2D-HSQC NMR spectrum of 1β-dehydroxybaccatin VI (16).

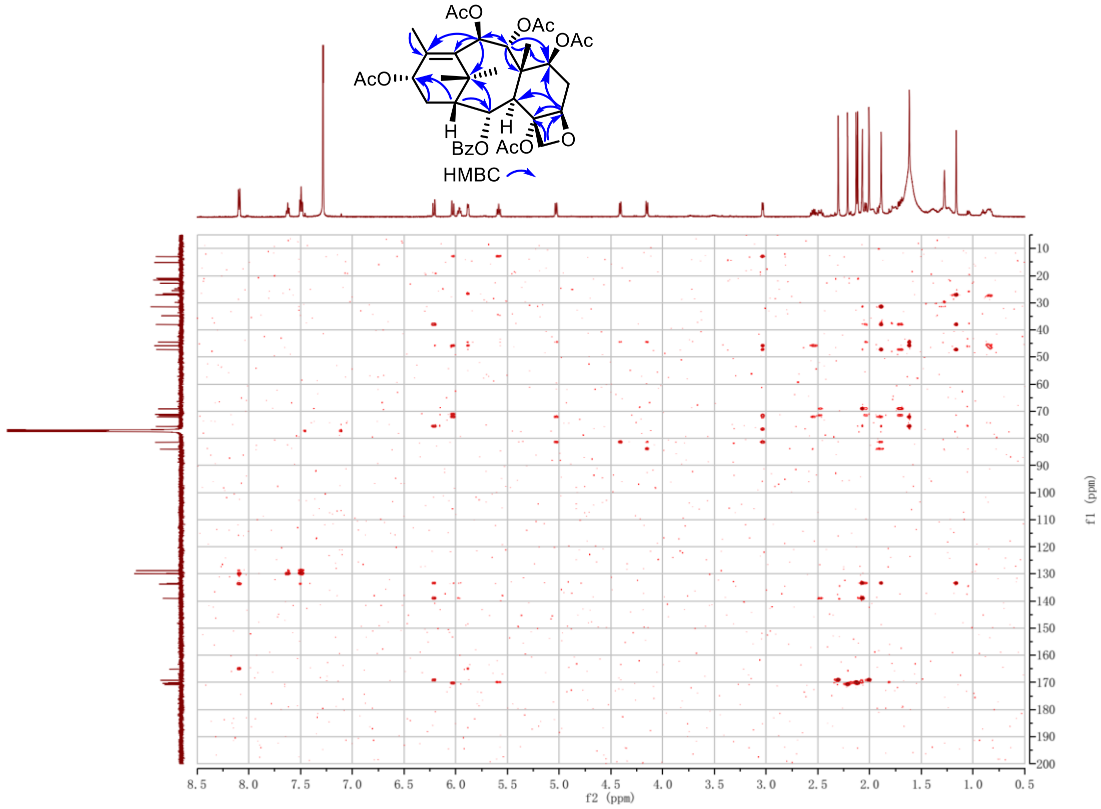

Supplementary Figure 94. 2D-HMBC NMR spectrum of 1β-dehydroxybaccatin VI (16).

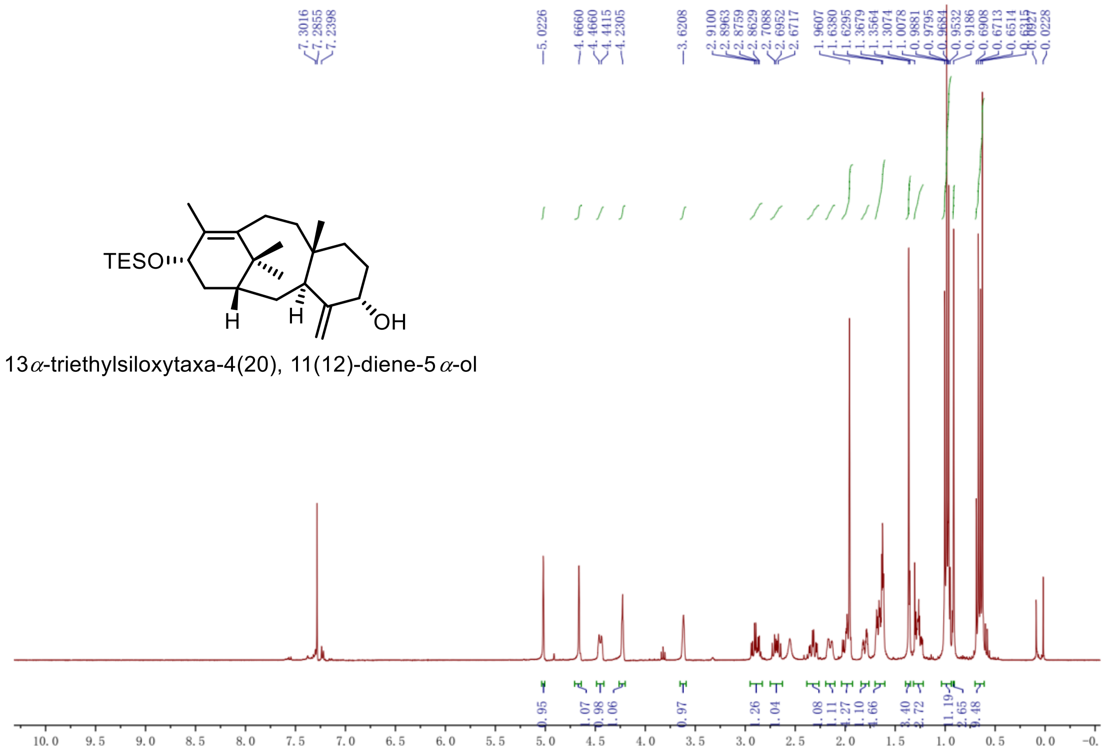

Supplementary Figure 95. ^{1}H NMR spectrum of 13α-triethylsiloxytaxa-4(20), 11(12)-diene-5α-ol (29).

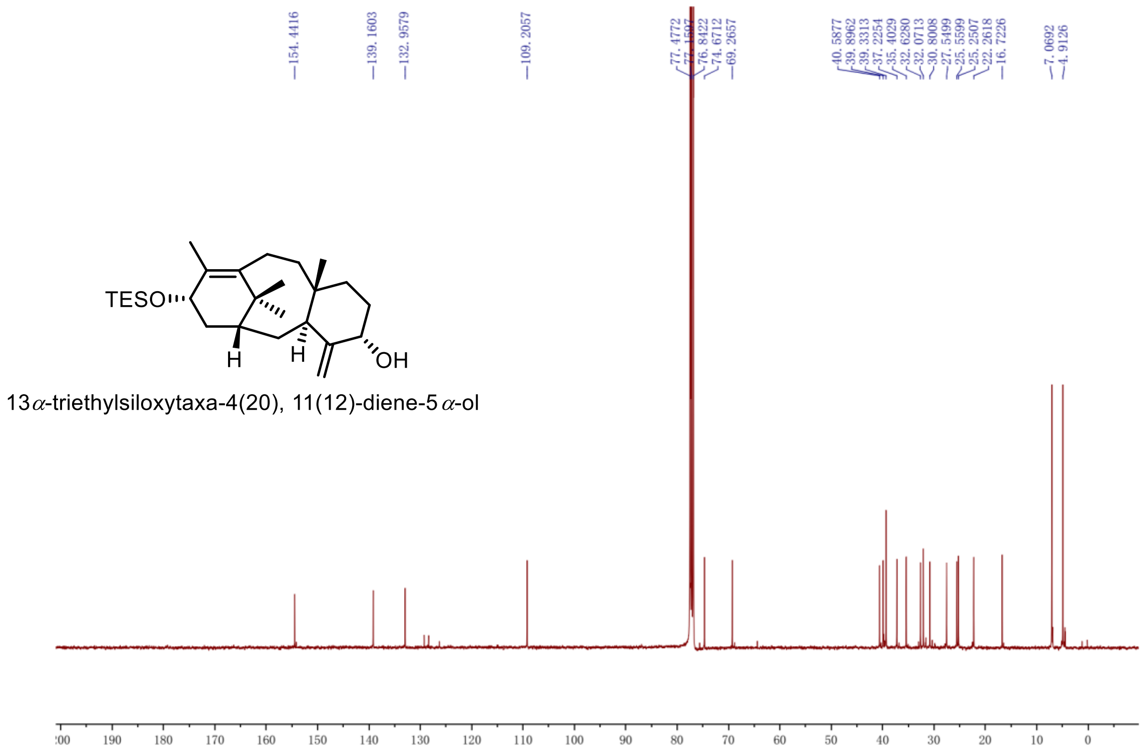

Supplementary Figure 96. ^{13}C NMR spectrum of 13α-triethylsiloxytaxa-4(20), 11(12)-diene-5α-ol (29).

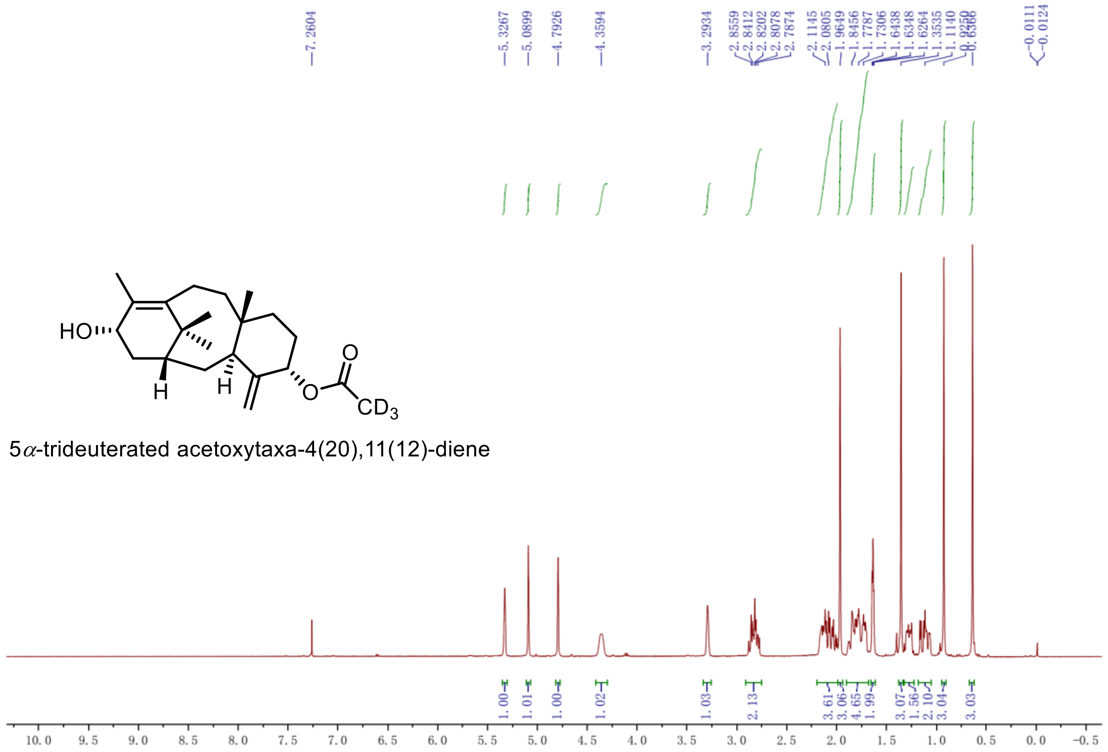

Supplementary Figure 97. ^{1}**H NMR spectrum of 5*α*-trideuterated acetoxytaxa-4(20),11(12)-diene (18).**

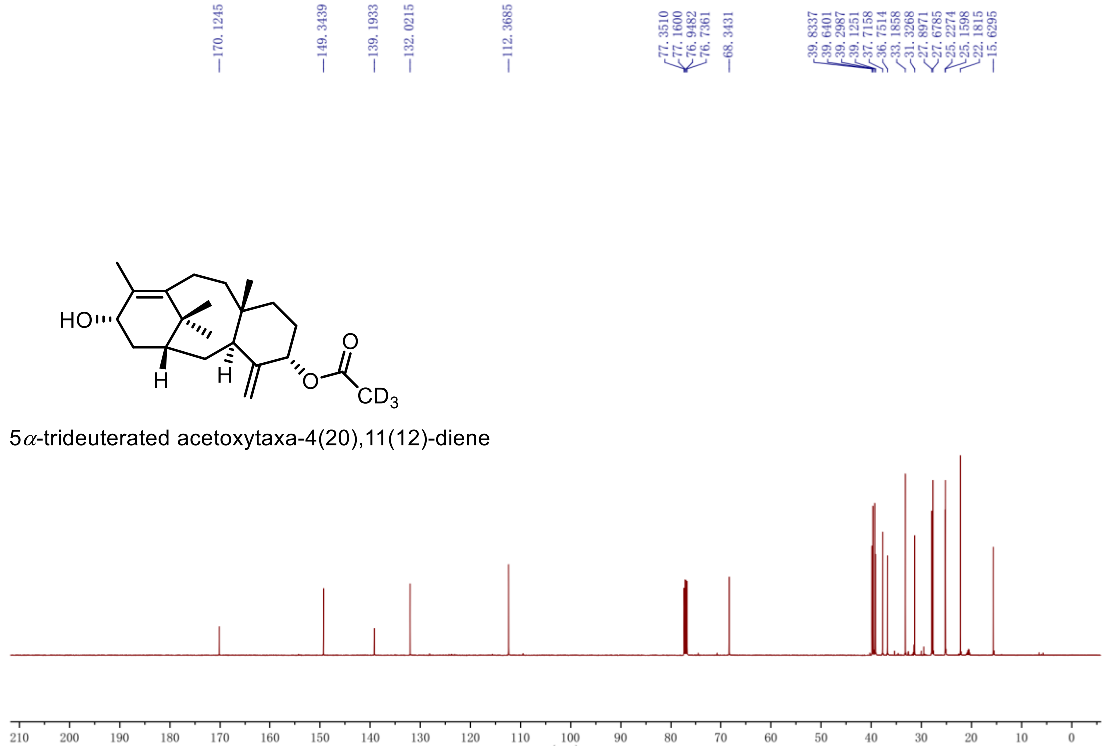

Supplementary Figure 98. ^{13}**C NMR spectrum of 5*α*-trideuterated acetoxytaxa-4(20),11(12)-diene (18).**

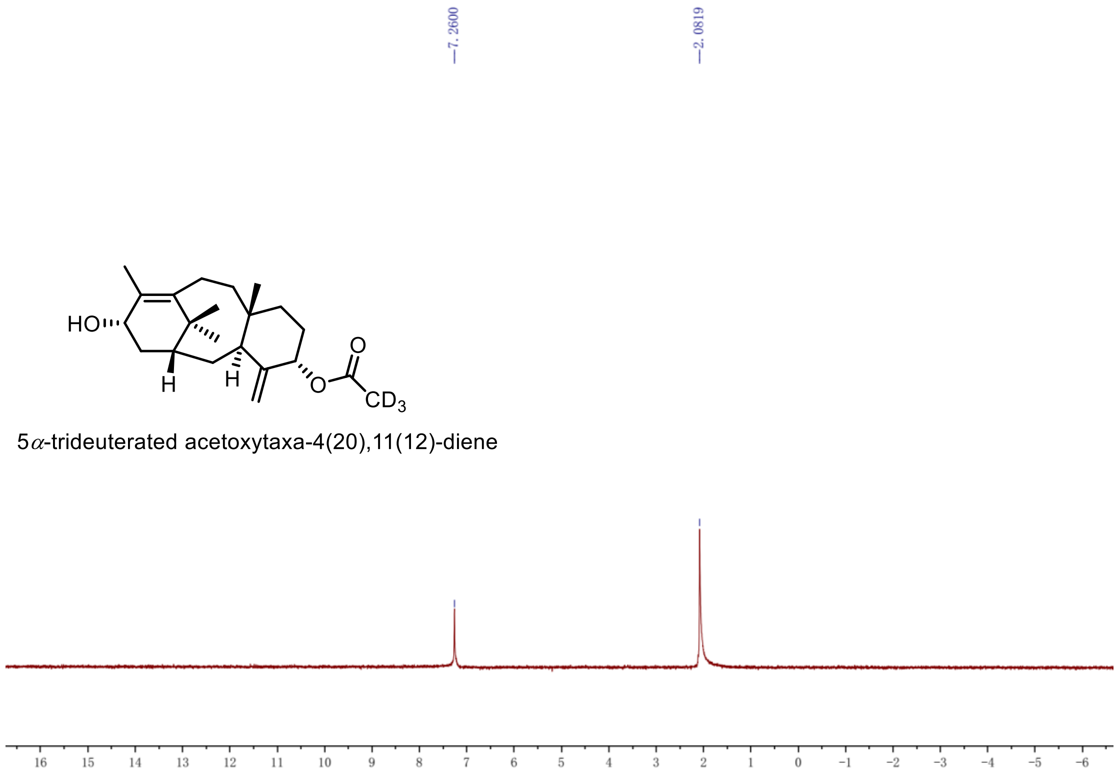

Supplementary Figure 99. ^{2}H NMR spectrum of 5*α*-trideuterated acetoxytaxa-4(20),11(12)-diene (18).

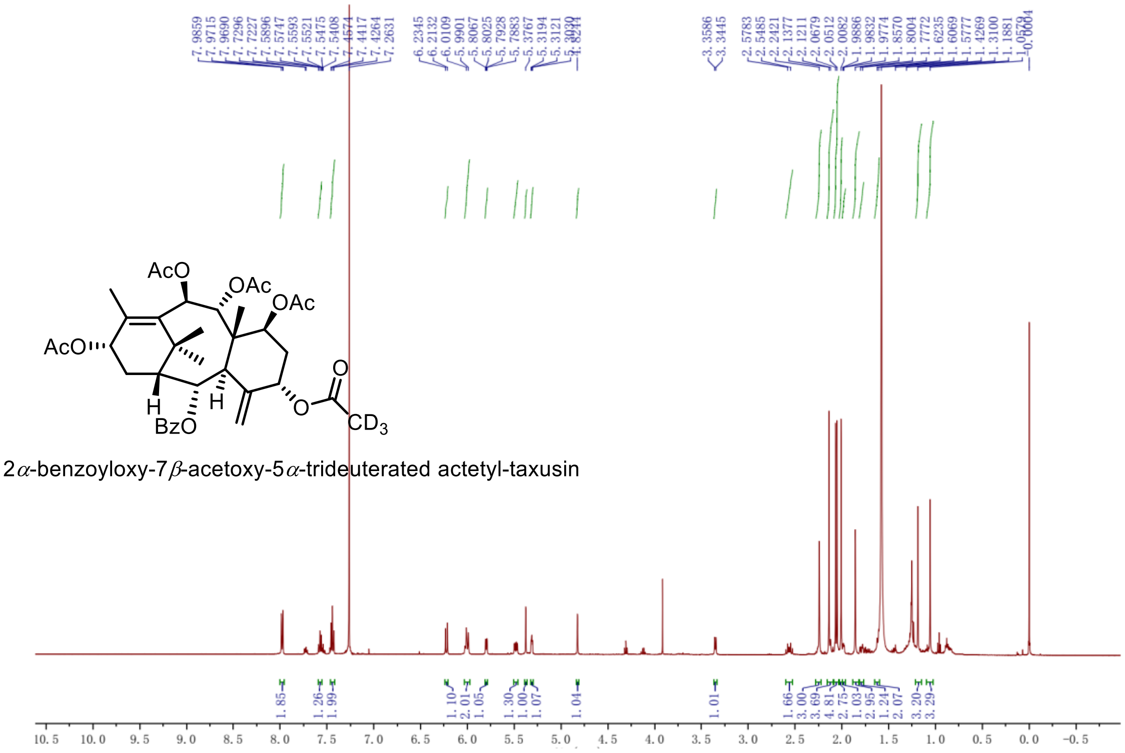

Supplementary Figure 100. ^{1}H NMR spectrum of 2*α*-benzoyloxy-7*β*-acetoxy-5*α*-trideuterated acetyl-taxusin (20).

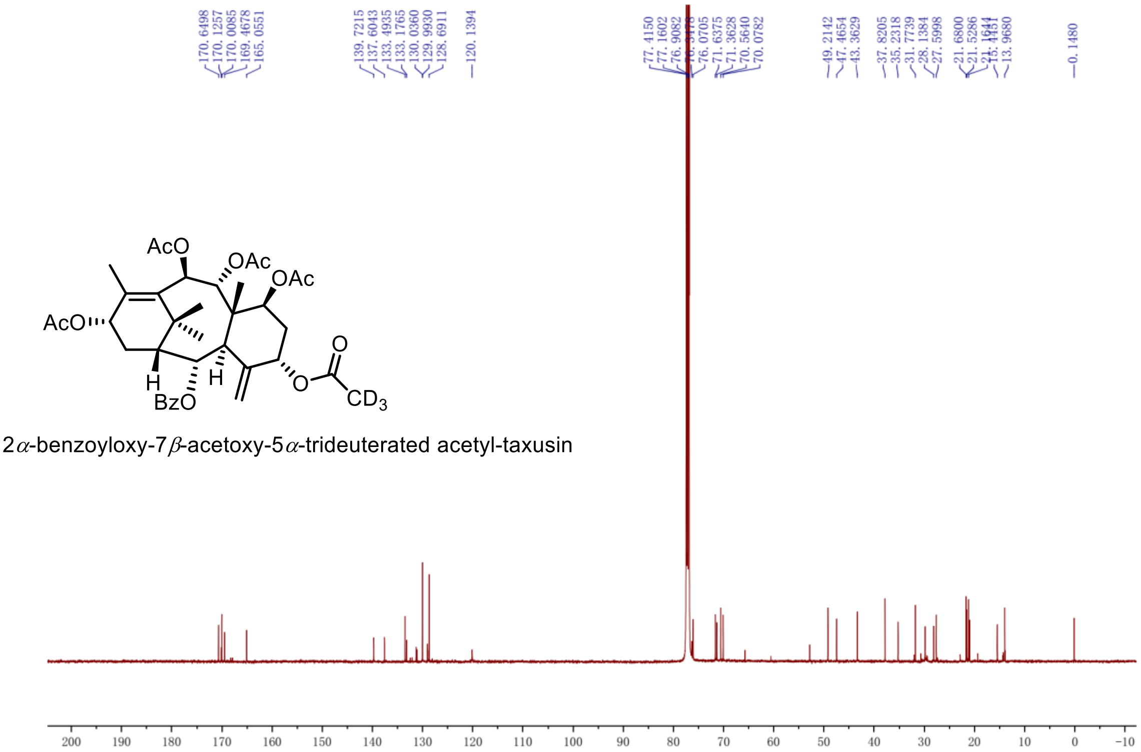

Supplementary Figure 101. **^{13}C NMR spectrum of 2*α*-benzoyloxy-7*β*-acetoxy-5*α*-trideuterated acetyl-taxusin (20).**

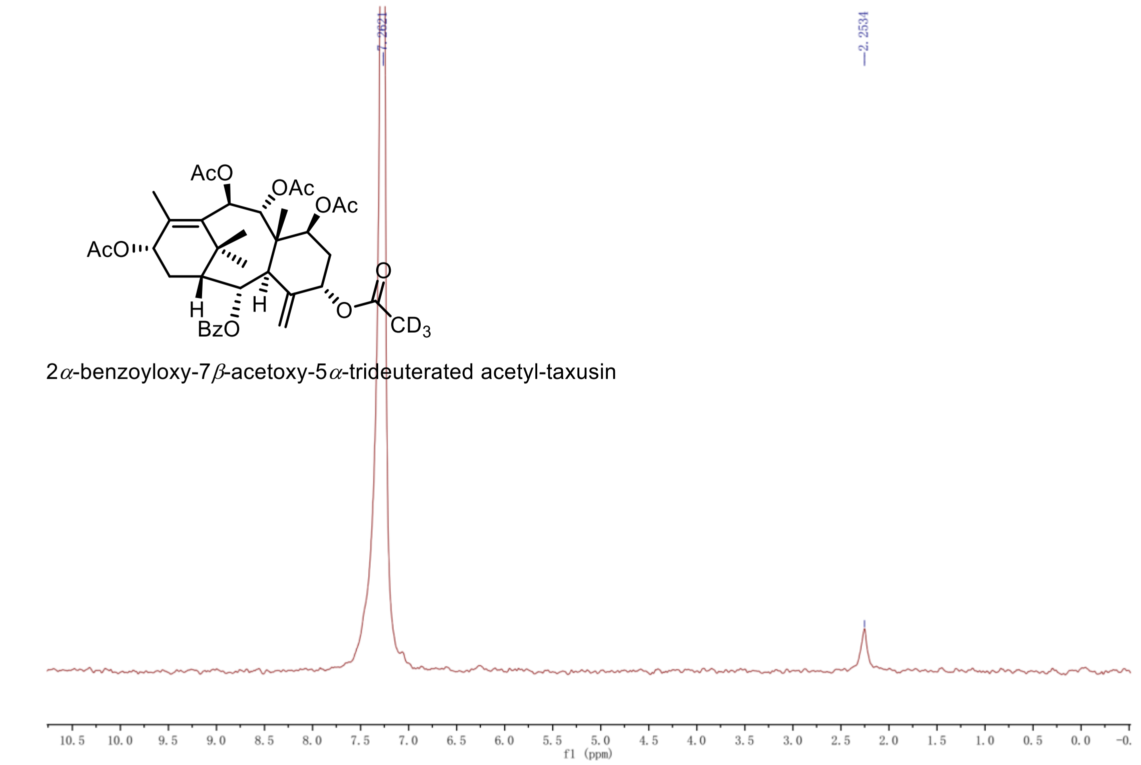

Supplementary Figure 102. **^{2}H NMR spectrum of 2*α*-benzoyloxy-7*β*-acetoxy-5*α*-trideuterated acetyl-taxusin (20).**

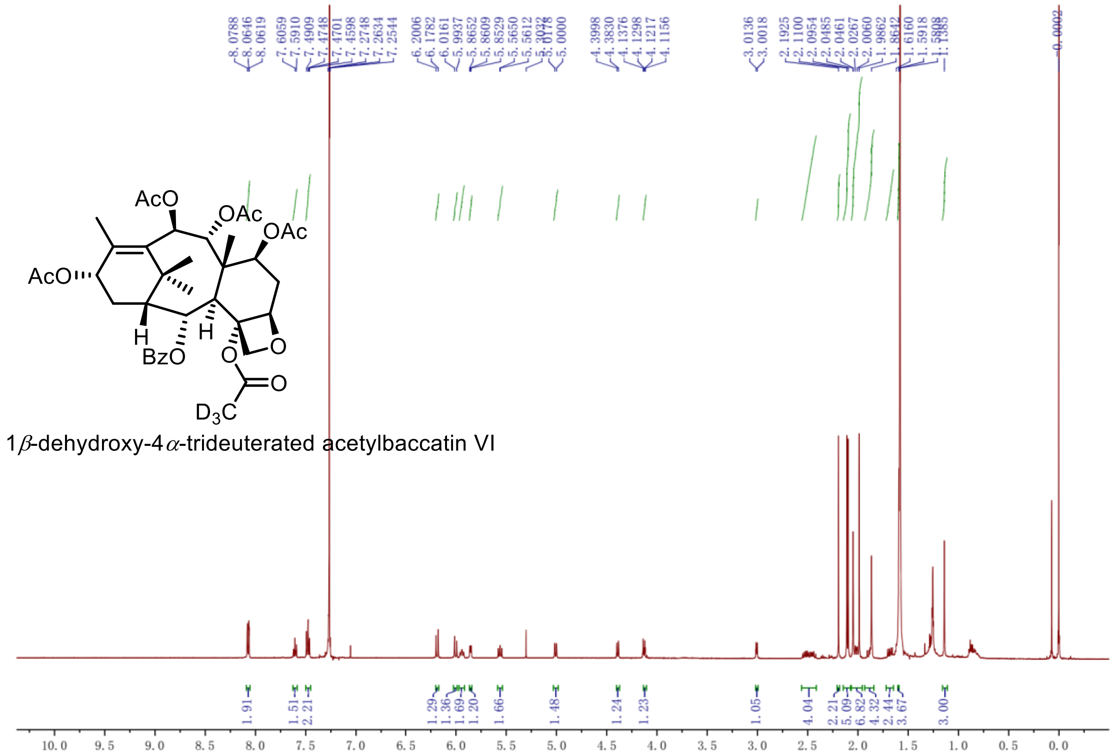

Supplementary Figure 103. ^{1}H NMR spectrum of 1*β*-dehydroxy-4α-trideuterated acetylbaccatin VI (21).

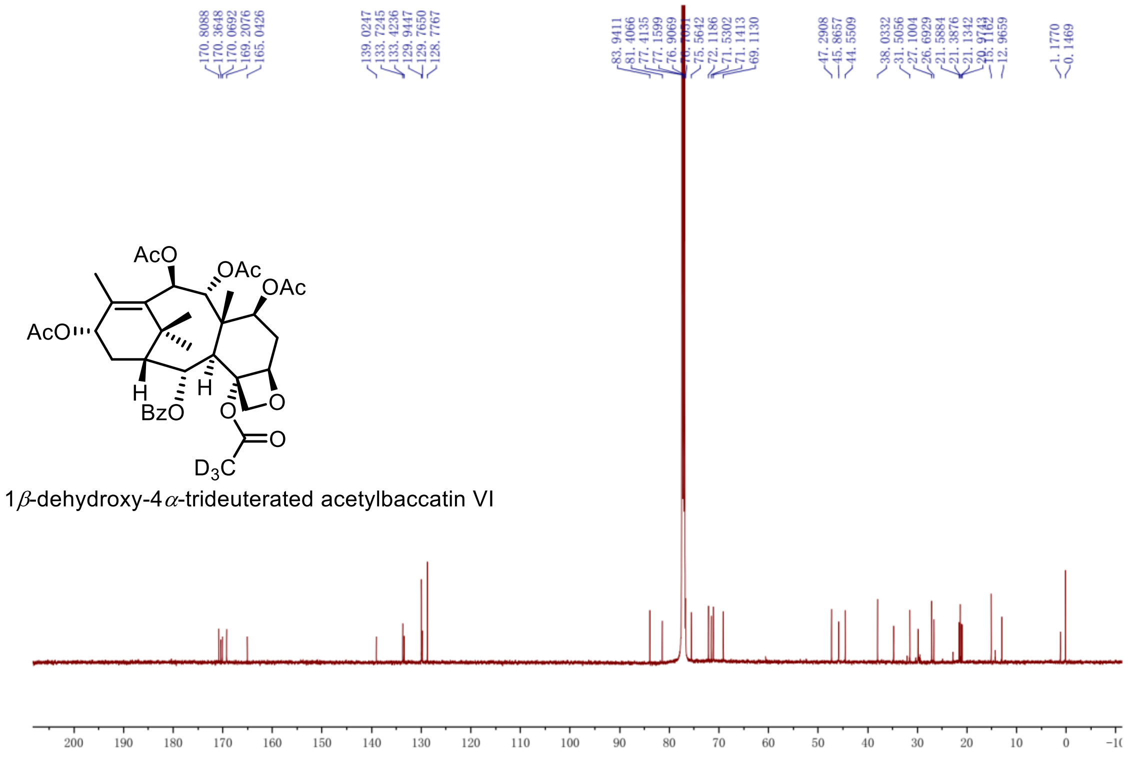

Supplementary Figure 104. ^{13}C NMR spectrum of 1*β*-dehydroxy-4α-trideuterated acetyl-baccatin VI (21).

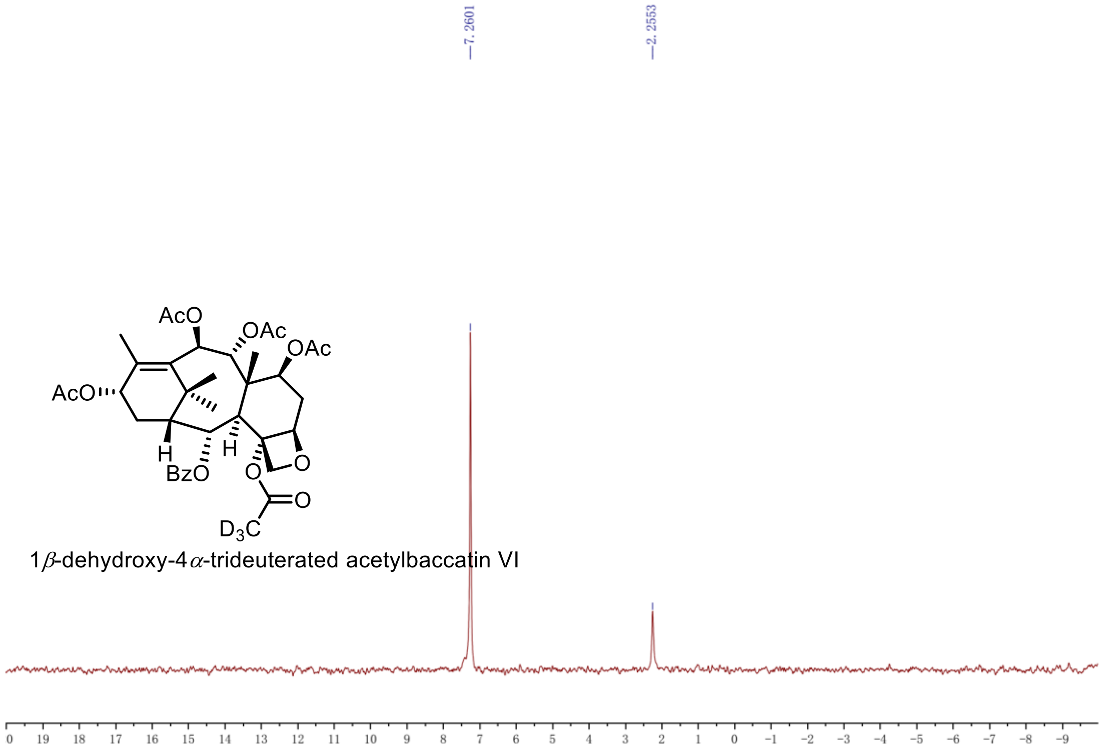

Supplementary Figure 105. ^{2}H NMR spectrum of 1β-dehydroxy-4α-trideuterated acetyl-baccatin VI (21).

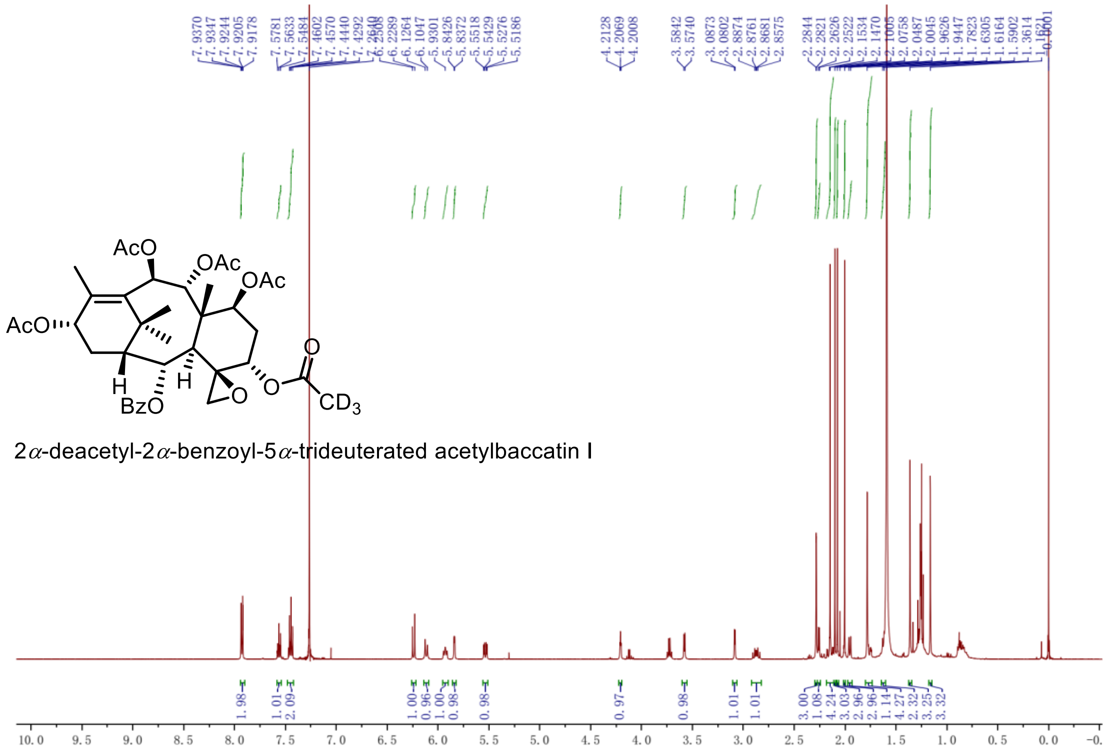

Supplementary Figure 106. ^{1}H NMR spectrum of 2*α*-deacetyl-2*α*-benzoyl-5*α*-trideuterated acetylbaccatin I (22).

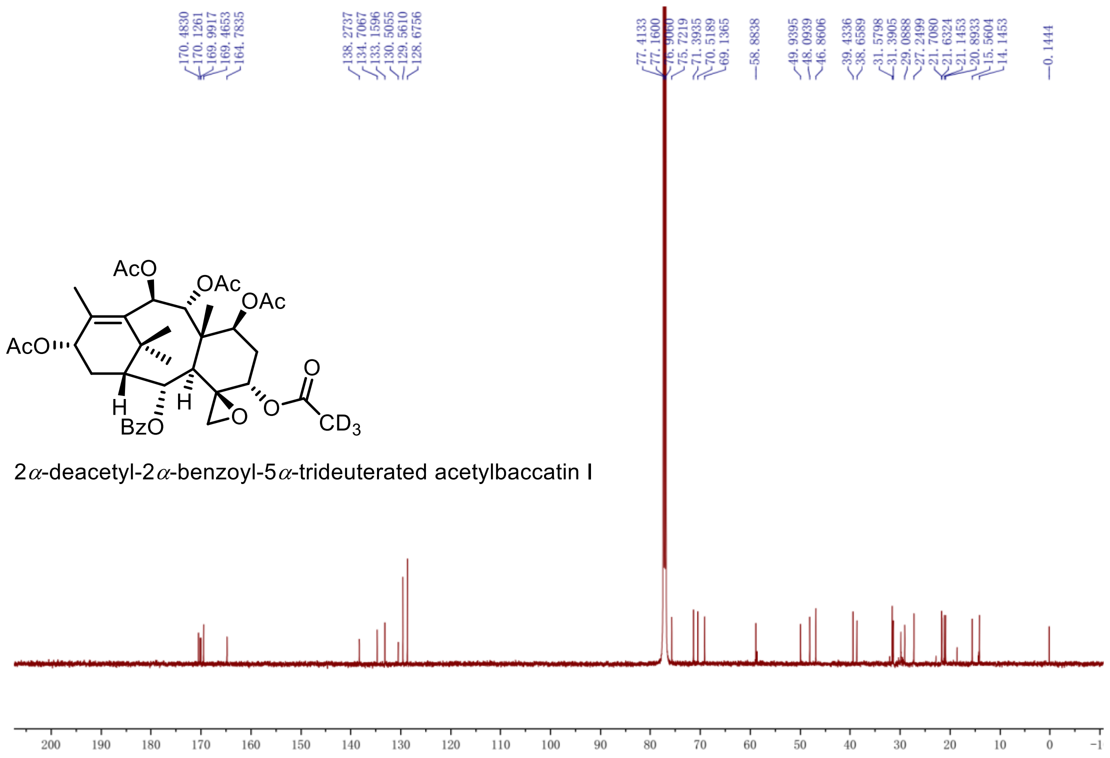

Supplementary Figure 107. ^{13}C NMR spectrum of 2*α*-deacetyl-2*α*-benzoyl-5*α*-trideuterated acetylbaccatin I (22).

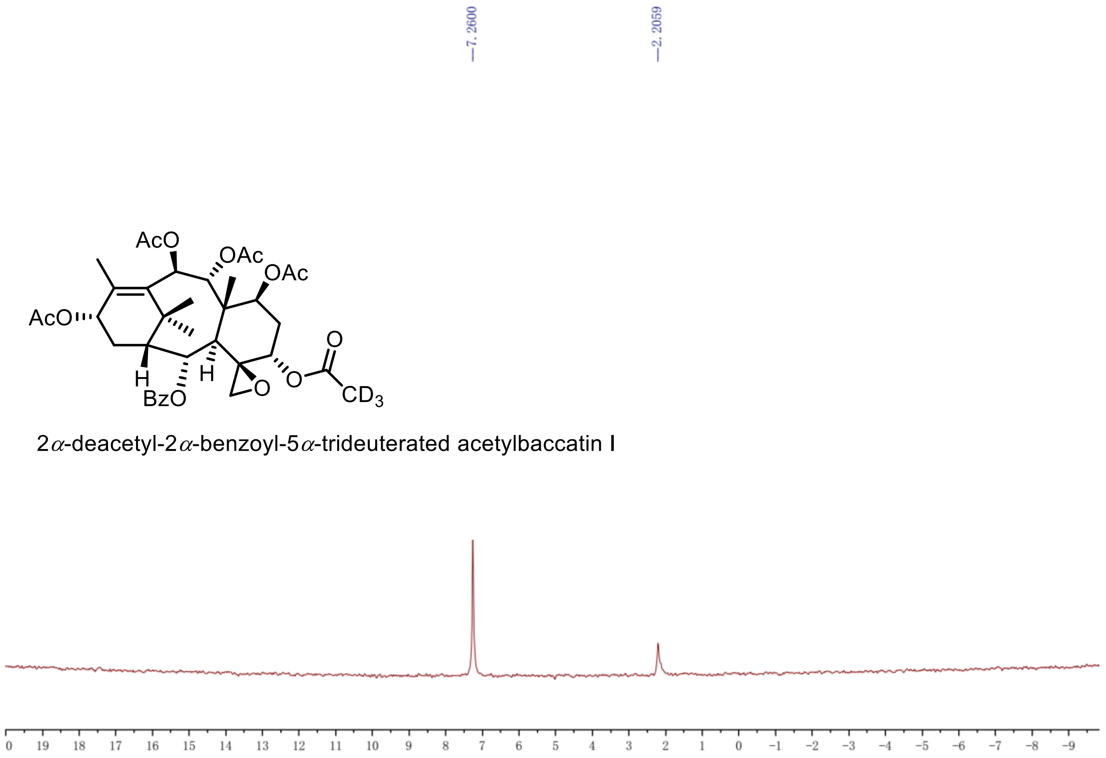

Supplementary Figure 108. ^{2}H NMR spectrum of 2*α*-deacetyl-2*α*-benzoyl-5*α*-trideuterated acetylbaccatin I (22).

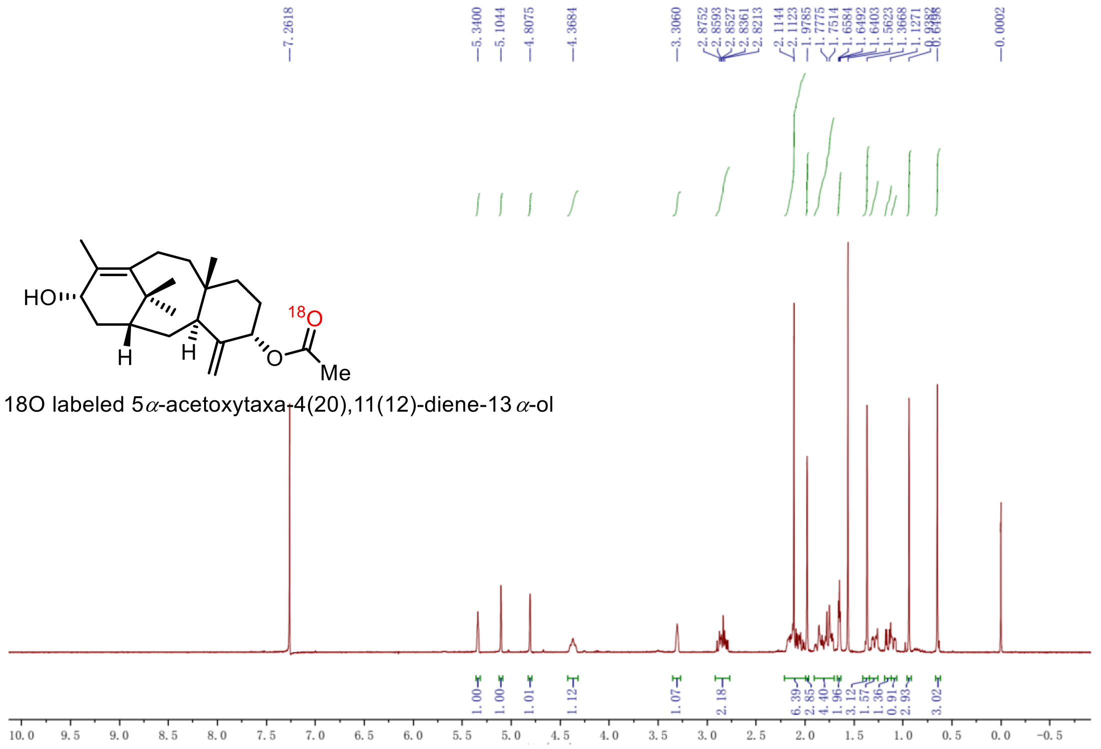

Supplementary Figure 109. 1H NMR spectrum of 18O labeled 5α-acetoxytaxa-4(20),11(12)-diene-13α-ol (23).

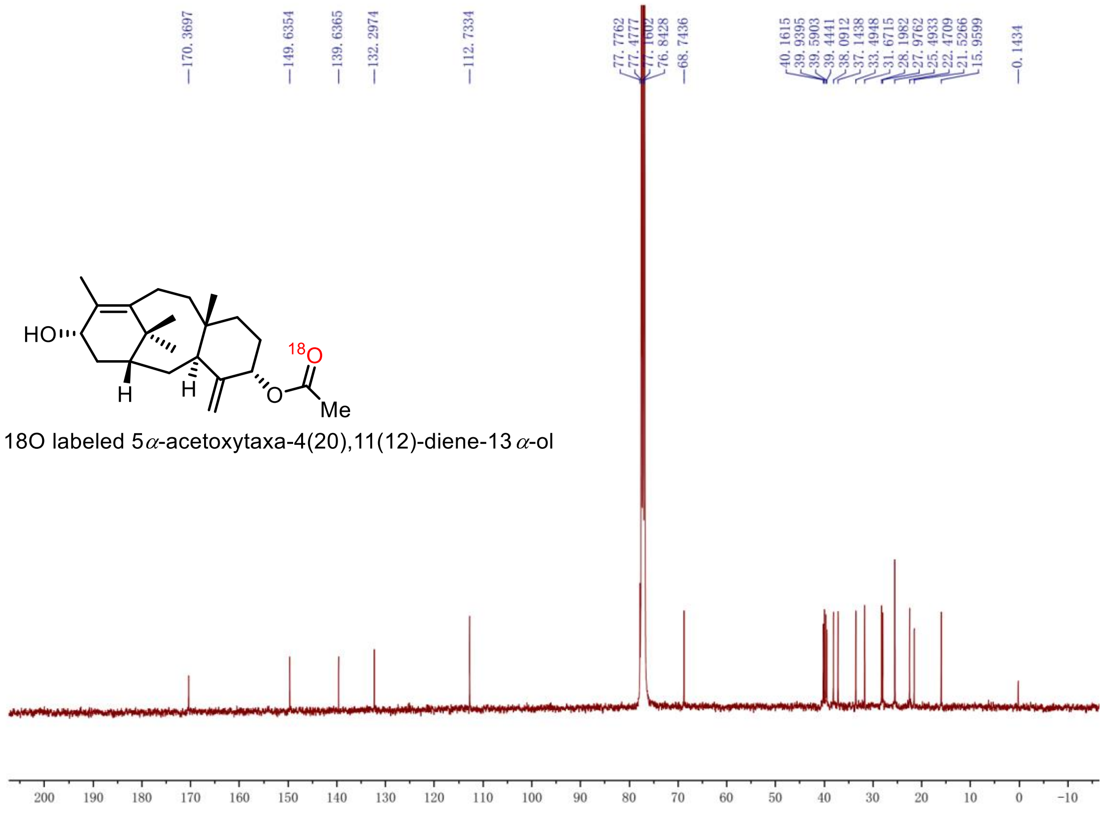

Supplementary Figure 110. ^{13}C NMR spectrum of ^{18}O labeled 5*α*-acetoxytaxa-4(20),11(12)-diene-13*α*-ol (23).

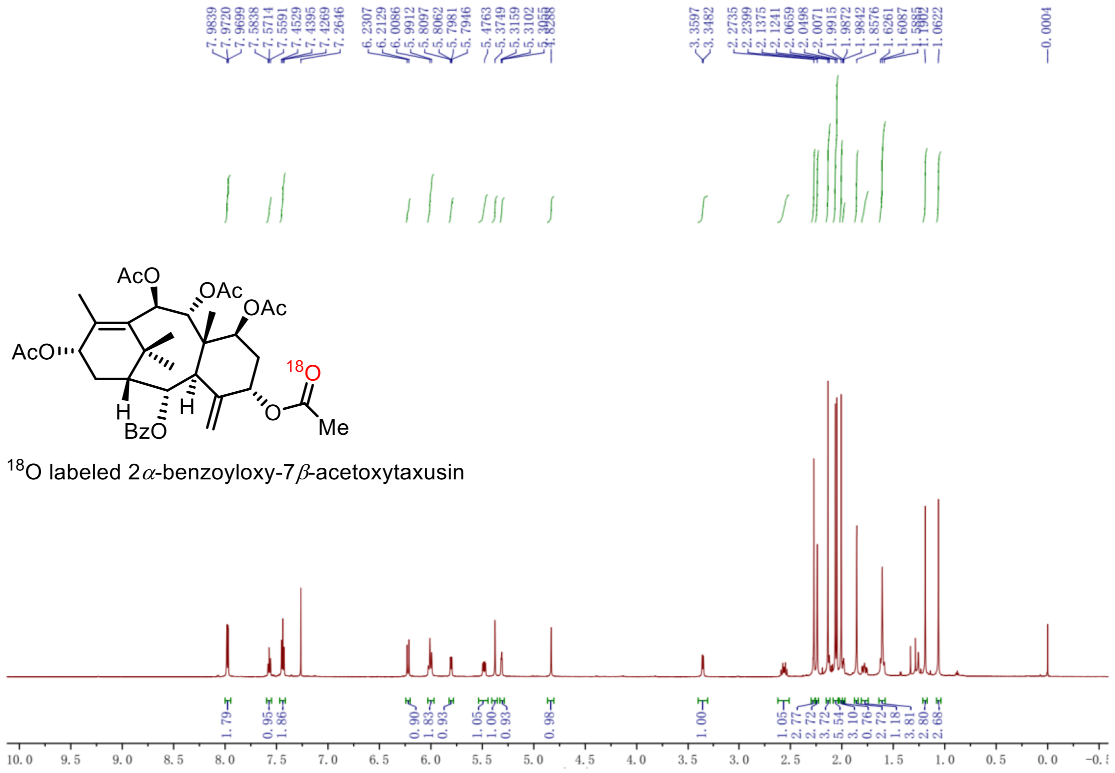

Supplementary Figure 111. ^{1}H NMR spectrum of ^{18}O labeled 2*α*-benzoyloxy-7*β*-acetoxytaxusin (25).

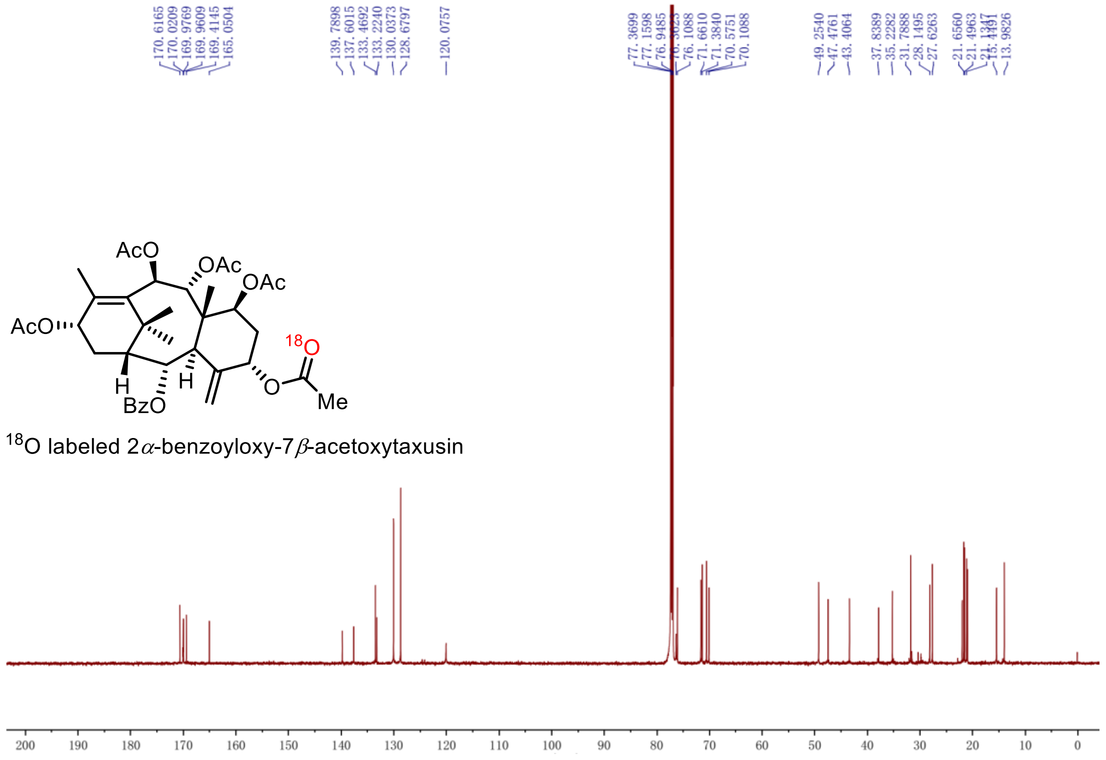

Supplementary Figure 112. ^{13}C NMR spectrum of ^{18}O labeled 2*α*-benzoyloxy-7*β*-acetoxytaxusin (25).

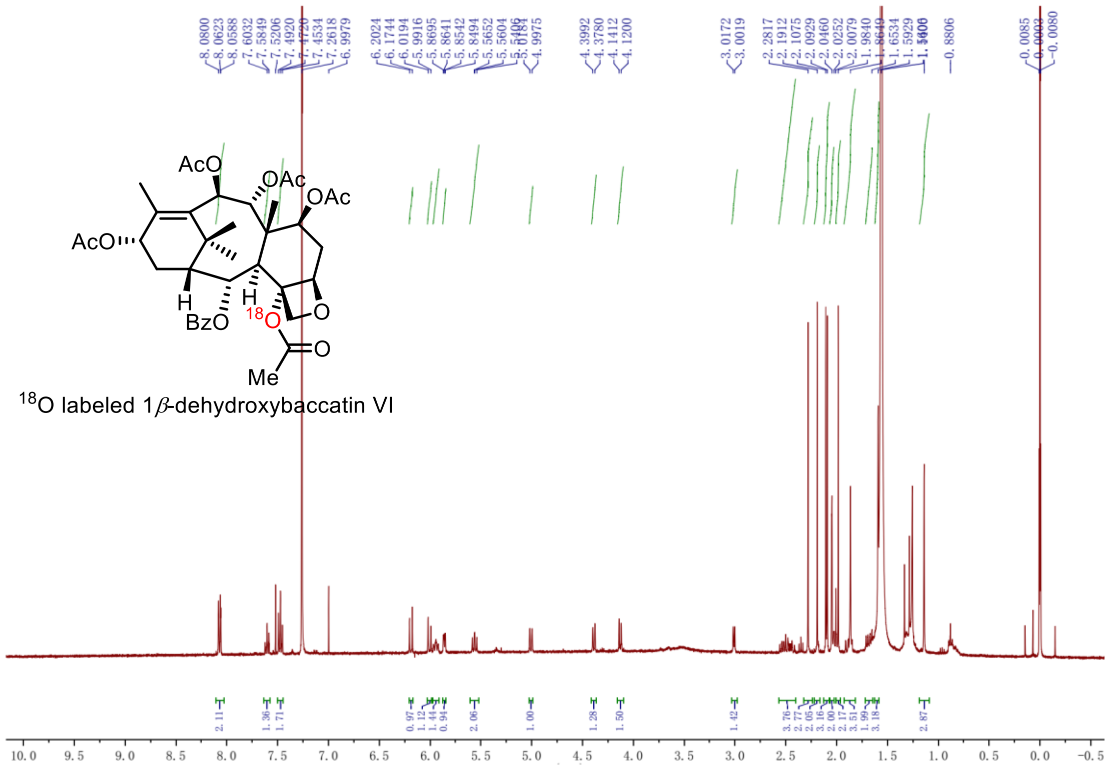

Supplementary Figure 113. ¹H NMR spectrum of ¹⁸O labeled 1β-dehydroxybaccatin VI (26a).

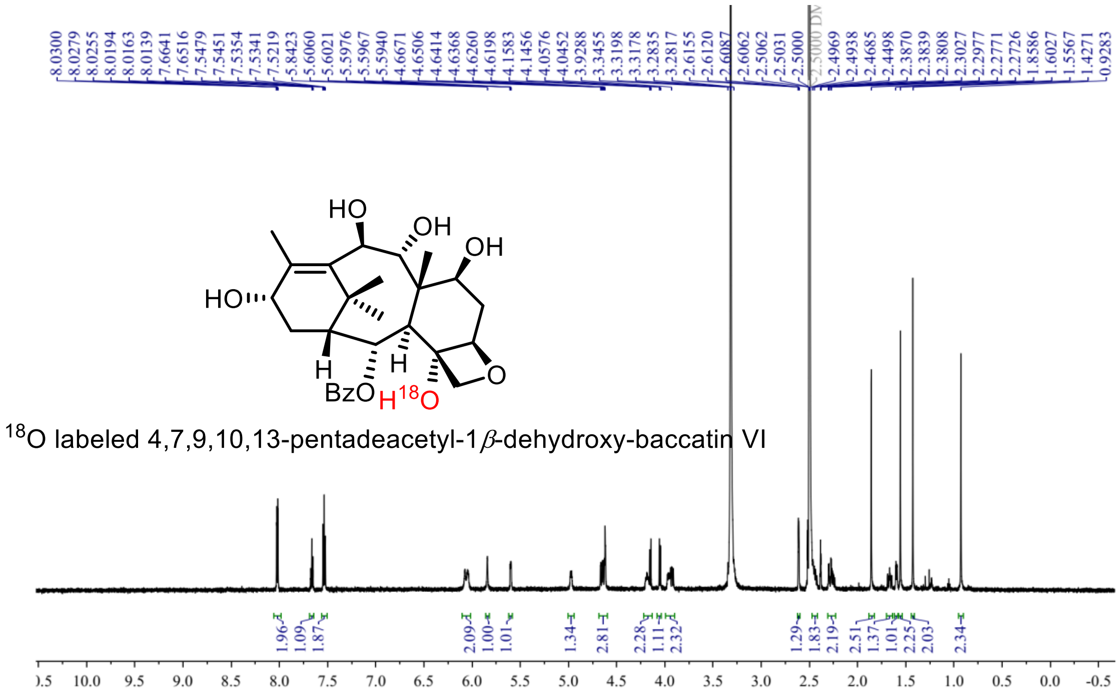

Supplementary Figure 114. ¹H NMR spectrum of ¹⁸O labeled 4,7,9,10,13-pentadeacetyl-1*β*-dehydroxybaccatin VI (28a).

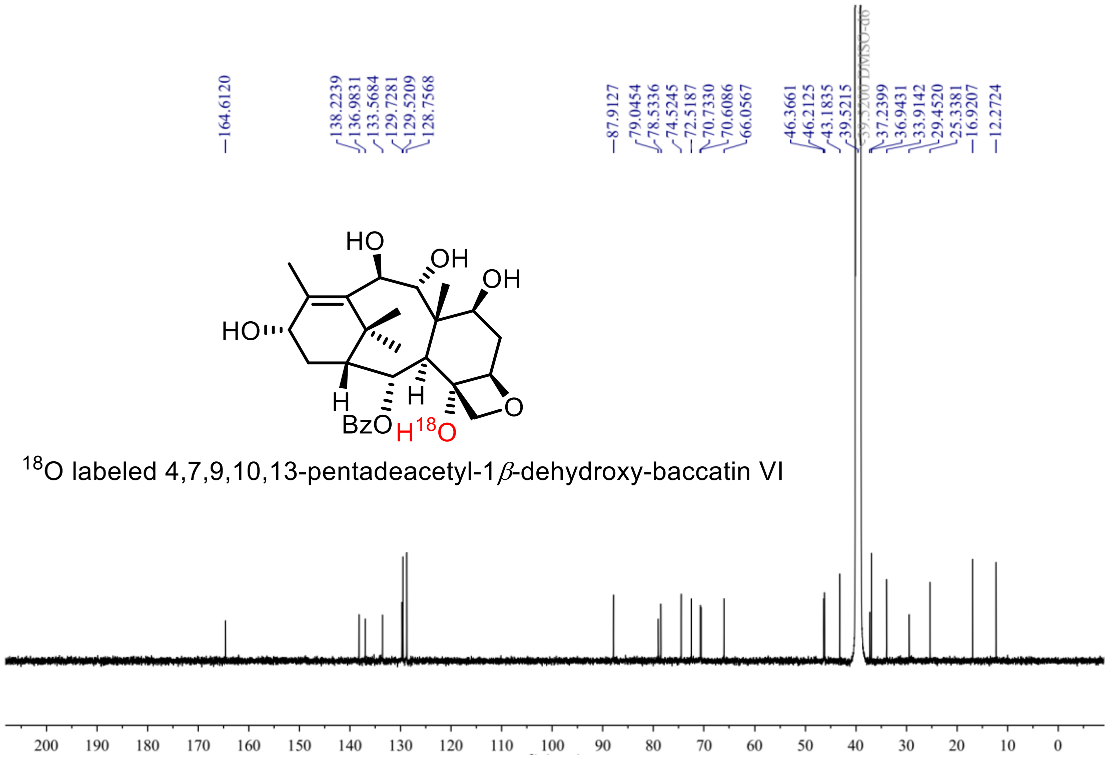

Supplementary Figure 115. ^{13}**C NMR spectrum of** ^{18}**O labeled 4,7,9,10,13-pentadeacetyl-1*β*-dehydroxybaccatin VI (28a).**

Supplementary Table 1. Summary of activities of characterized P450s towards different taxoid compounds.

**Enzyme**	**T5OH**	**T13OH**	**T10OH**	**T10OH2**	**CYP725A37**	**T7OH**	**T2OH**	**CYP725A55**	**T2'OH**
Compound **1**	Y(C5)	N	*	N	*	N	N	*	*
Compound **2**	N	Y(C13)	*	N	*	N	N	*	*
Compound **3**	N	U	Y(C10)	Y(C10)	*	N	N	*	*
Compound **4**	N	N	*	N	*	N	N	*	*
Compound **5**	N	*	N/Y(C10)	N/Y(C10)	*	N	*	*	*
Compound **6**	*	*	Y(C10)	Y(C10)	*	*	*	*	*
Compound **7**	*	*	*	*	*	*	*	*	*
Compound **8**	*	*	*	*	*	*	*	*	*
Compound **9**	*	*	Y(C10)	Y(C10)	*	*	*	*	*
Compound **10**	*	*	*	*	Y(C9)	*	*	*	*
Compound **11**	*	*	*	*	*	*	*	*	*
Compound **12**	N	*	*	*	*	Y(C7)	Y(C2)	*	*
Compound **13**	*	*	*	*	*	*	*	*	*
Compound **15**	*	*	*	*	*	*	*	Y(Oxetane)	*
Compound **16**	*	*	*	*	*	*	*	*	*
Compound **17**	*	*	*	*	*	*	*	N	*
**9,10,13-deacetyltaxusin**	*	*	*	*	*	N	N	*	*
**Taxadien-5*α*,10*β*-diol**	*	*	*	*	*	*	N	*	*
**Baccatin III**	*	*	*	*	*	*	*	*	Y(C2')
**Source**	Ref. 1	Ref. 2	Ref. 3 and this study	Ref. 4 and this study	This study	Ref. 5	Ref. 6	This study	Ref. 7

Y means the activity of enzyme towards this compound has been characterized; N means it was failed to catalyze this compound by corresponding enzyme; U means the product of this enzyme towards corresponding substrate is unidentified; * means there is no report about this reaction. Activities or enzymes marked in red were characterized in this study.

Supplementary Table 2. All the possible combinations of three continuous conversion from taxa-4(20),11(12)-diene-5α-ol (2) with T13OH, T10OHs, and T5AT.

Combination	A	B	C	D	E	F
**Substrate**	**2**	**2**	**2**	**2**	**2**	**2**
**1st step**	T5AT	T5AT	T10OHs	T10OHs	T13OH	T13OH
**2nd step**	T10OHs	T13OH	T5AT	T13OH	T5AT	T10OHs
**3rd step**	T13OH	T10OHs	T13OH	T5AT	T10OHs	T5AT
**Result**	Unsuccessful	hard	Unsuccessful	Unsuccessful	Unfinished	Unsuccessful
**Source**	Ref. 2	Ref. 2	Ref. 4	Ref. 4	This study	Ref. 4

The enzymes marked in red have been demonstrated to be failed or hard to catalyze the reaction. The enzymes marked in blue have been characterized to catalyze the reaction. The enzymes marked in gray have not been tested or characterized. The activities of T10OHs were mainly based on the activities of T10OH2 based on the extensive substrates testing for T10OH2. For combination B, due to the conversion rate of T13OH towards 5α-acetoxytaxa-4(20),11(12)-diene (**3**) was very low[2], this combination was regarded to be hard to complete this three-step conversion.

Supplementary Table 3. Summary of the compounds involved in this study.

Compound	Isolated from *Taxus* plants	Biosynthesized	Chemsynthesized	New compound
**1**		√	√	
**2**		√	√	
**3**		√	√	
**4**		√	√	
**5**		√		
**6**		This study		This study
**7**		This study		This study
**8**		This study	√	
**9**		This study	√	
**10**		This study		This study
**11**	√	This study	√	
**12**	√	This study	√	
**13**		√		
**15**		This study		This study
**16**	√	This study		
**17**		This study		This study
**18**				This study
**19**				This study
**20**				This study
**21**				This study
**22**				This study
**23**				This study
**24**				This study
**25**				This study
**26a**				This study
**27**				This study
**28a**				This study

Supplementary Table 4. Strains used in this study.

Stain	Genotype/Description	Source
BY4742	*MATa*, *his3*Δ1, *leu2*Δ0, *lys2*Δ0, *ura3*Δ0	EUROSCARF
YBD80	BY4742 {gal80::URA3}	This study
YTCPR	YBD80 {deltaDNA::GAL10p-*synTCPR*-TDH3t-LEU2}	This study
YT13OH	YBD80 {rDNA::GAL10p-synTCPR-TDH3t/GAL1p-synT13OH-GPM1t-HIS3}	This study
YT5AT	YBD80 {rDNA::GAL1p-synT5AT-GPM1t-HIS3}	This study
YT10OH	YBD80 {rDNA::GAL10p-synTCPR-TDH3t/GAL1p-synT10OH-GPM1t-HIS3}	This study
YT10OH2	YTCPR {rDNA:: GAL1p-T10OH2-GPM1t-HIS3}	This study
YTAX19	YBD80 {rDNA::GAL1p-synTAX19-GPM1t-HIS3}	This study
YCYP725A37	YTCPR {rDNA:: GAL1p-synCYP725A37-GPM1t-HIS3}	This study
YCYP725A55	YTCPR {rDNA:: GAL1p-CYP725A55-GPM1t-HIS3}	This study
YTBT	YBD80 {rDNA::GAL1p-synTBT-GPM1t-HIS3}	This study
YAAE	YBD80 {deltaDNA::GAL1p-synAAE-HXT7t-LEU2}	This study
YTA	YT01 {deltaDNA::GAL1p-tHMG1-CYC1t/GAL10p-ERG19-ADH1t/GAL1p-synTS-ENO2t/GAL10p-IDI1-PGIt/GAL1p-synGGPPs-FBA1t –LEU2}	This study
YT01	YBD80 {rDNA::GAL10p-synTCPR-TDH3t/GAL1p-synT13OH-TEF1t/GAL10p-synT5AT-TPI1t/GAL1p-synT10OH-TEF2t-HIS3}	This study
YT02	YT01 {deltaDNA::GAL1p-synTAX19-CYC1t/GAL10p-synCYP725A37-ADH1t/GAL1p-T2OH-ENO2t/GAL10p-synT7OH-PGIt-LEU2}	This study
YT03	YT01 {deltaDNA::GAL1p-synTAX19-PYK1t -LEU2}	This study
YT04	YT01 {deltaDNA::GAL1p-synTAX19-CYC1t/GAL10p-synCYP725A37-ADH1t-LEU2}	This study
YDBVI	YT02 {rDNA::GAL1p-synAAE4-TDH2t/GAL10p-synTBT-HXT7t/GAL1p-CYP725A55-PDC1t/GAL10p-AT5-PYK1t-KanMX}	This study
BL21(DE3)	*E.coli* expression chassis	Invitrogen
Y28A	BL21 {pET28a}	This study
YAT5	BL21 {pET28a-AT5}	This study

Supplementary Table 5. Concentration of substrate and estimated concentration of product in *in vivo* feeding assay experiments.

Substrate	Final concentration of substrate	Product	Estimated concentration of product
**2**	144 mg/L (500uM)	**5**	137 mg/L
**5**	152 mg/L (500uM)	**6**	129 mg/L
**6**	173 mg/L (500uM)	**7**	166 mg/L
**5**	152 mg/L (500uM)	**8**	48 mg/L
**8**	160 mg/L (500uM)	**7**	6 mg/L
**6**	173 mg/L (500uM)	**9**	115 mg/L
**9**	194 mg/L (500uM)	**10**	73 mg/L
**10**	202 mg/L (500uM)	**11**	34 mg/L
**11**	210 mg/L (500uM)	**12**	161 mg/L
**15**	17 mg/L (25uM)	**16** or **17**	3 mg/L

The concentration of product was estimated on the conversion rate and the assumption that the substrate and corresponding product shared the same chromatographic response.

Supplementary Table 6. The ^{1}H NMR and ^{13}C NMR data for the biosynthesized 1*β*-dehydroxybaccatin VI (16) by using $CDCl_3$ as solvent.

Position number	Biosynthesized 1*β*-dehroxybaccatin VI	
	^{1}H NMR (600 MHz)	^{13}C NMR (150 MHz)
1	2.01 (d, *J*= 8.9 Hz, 1H, 1*β*-H)	47.28
2	5.86 (d, *J*= 5.4 Hz, 1H, 2*β*-H)	71.52
3	3.01 (d, *J*= 5.6 Hz, 1H, 3*α*-H)	44.54
4		81.42
5	5.00 (d, *J*= 9.0 Hz, 1H, 5*α*-H)	83.93
6	2.55-2.49 (m, 1H, 6*α*-H), 1.92-1.86 (m, 1H, 6*β*-H)	34.78
7	5.58-5.54 (m, 1H, 7*α*-H)	72.10
8		45.86
9	6.00 (d, *J*= 11.0 Hz, 1H, 9*β*-H)	75.55
10	6.18 (d, *J*= 11.0 Hz, 1H, 10*α*-H)	71.13
11		133.41
12		139.02
13	5.96-5.92 (m, 1H, 13*β*-H)	69.11
14	2.49-2.42(m, 1H, 14*β*-H), 1.68 (dd, *J*= 15.0, 7.0 Hz, 1H, 14*α*-H)	26.69
15		38.03
16	1.86 (s, 3H)	27.09
17	1.14 (s, 3H)	31.50
18	2.04 (s, 3H)	15.11
19	1.59 (s, 3H)	12.96
20	4.38 (d, *J*= 8.2 Hz, 1H, 20*α*-H) 4.13 (d, *J*= 8.2 Hz, 1H, 20*β*-H)	76.70
2-OBz	8.09-8.06 (m, 2H), 7.62-7.58 (m, 1H) 7.50-7.44 (m, 2H)	165.03, 133.71, 129.94, 129.76, 128.77
4-OAc	2.28 (s, 3H)	169.17, 22.84
7-OAc	2.10 (s, 3H)	170.05, 21.58
9-OAc	2.09 (s, 3H)	170.35, 20.97
10-OAc	1.98(s, 3H)	169.17, 21.13
13-OAc	2.19 (s, 3H)	170.80, 21.39

Supplementary Note 1. General protocols for the construction of yeast and *E.coli* strains

(1) For the construction of strain YBD80, the ORF of GAL80 gene was replaced with URA3 marker. The upstream and downstream homologous arms of GAL80 gene were PCR amplified from *S. cerevisiae* BY4742 genomic DNA. URA3 marker was PCR amplified from the plasmid pUG72. These three fragments were co-transformed into BY4742 by LiAc approach and used to construct the strain YTBD80 through DNA assembler method.

(2) For the construction of strain YT13OH, upstream and downstream homologous arms of rDNA site, TDH3 and GPM1 terminators, and GAL10-GAL1 bidirectional promoter were PCR amplified from *S. cerevisiae* BY4742 genomic DNA. SynT13OH and synTCPR were PCR amplified using *S. cerevisiae* codon-optimized genes as templates. The HIS3 marker was PCR amplified from the plasmid pESC-his. Then, the secondary fusion PCR of three or two fragments was performed to reduce the number of fragments for transformation. These fused fragments were co-transformed into YBD80 by LiAc approach and used to construct the strain YT13OH through DNA assembler method.

(3) The strain YT10OH was constructed by using the similar method and replacing T13OH fragment with T10OH fragment. The strains YT5AT, YTAX19, and YTBT were constructed by using the similar method with some differences including replacement of T13OH fragment with T5AT, TAX19 or TBT fragment and removing the TDH3 terminator and TCPR fragments.

(4) For the construction of strain YAAE, the rDNA site was replaced with delta site. The strain YTAAE were constructed by using the similar method with some differences including replacement of T13OH and GPM1t fragments with AAE4 and HXT7t fragments and removing the TDH3 terminator and TCPR fragments. The HIS3 marker was replaced with the LEU2 marker and the chassis cell was changed with YTBT strain.

(5) For the construction of strain YTCPR, the rDNA site was replaced with delta site. The T13OH and GPM1 terminator fragments were removed, and the HIS3 marker was replaced with the LEU2 marker. Strains YT10OH2, YCYP725A37 and YCYP725A55 were constructed by using the similar method for the construction of YT13OH with

some modifications including the removing of the TDH3 terminator and TCPR fragments and the replacement of chassis cell YBD80 with YTCPR strain. For these above-mentioned strains, five colonies were verified by colony PCR and positive colonies were verified by Sanger sequencing.

(6) For the construction of strain YTA, upstream and downstream homologous arms of delta site, CYC1, ADH1, ENO2, PGI and FBA1 terminators, *S. cerevisiae* endogenous ERG19, IDI1 and truncated HMG1 genes and GAL10-GAL1 bidirectional promoter were PCR amplified from *S. cerevisiae* BY4742 genomic DNA. SynTS and synGGPPs were PCR amplified using corresponding *S. cerevisiae* codon-optimized genes as templates. LEU2 marker was PCR amplified from plasmid PCM217. Then, the secondary fusion PCR of three or two fragments was performed to reduce the number of fragments for transformation. The fused fragments were co-transformed into YBD80 by LiAc approach and used to construct the strain YTA through DNA assembler method.

(7) For the construction of strain YT01, upstream and downstream homologous arms of rDNA site, TDH3, TEF1, TPI1, and TEF2 terminators, and GAL10-GAL1 bidirectional promoter were PCR amplified from *S. cerevisiae* BY4742 genomic DNA. SynTCPR, synt13OH, synT5AT, and synT10OH were PCR amplified using corresponding *S. cerevisiae* codon-optimized genes as templates. The HIS3 marker was PCR amplified from the plasmid pESC-his. Then, the secondary fusion PCR of three or two fragments was performed to reduce the number of fragments for transformation. The fused fragments were co-transformed into YBD80 by LiAc approach and used to construct the strain YT01 through DNA assembler method.

(8) For the construction of strain YT02, upstream and downstream homologous arms of delta site, CYC1, ADH1, ENO2 and PGI terminators, and GAL10-GAL1 bidirectional promoter were PCR amplified from *S. cerevisiae* BY4742 genomic DNA. SynTAX19, synCYP725A37, and synT7OH were PCR amplified using corresponding *S. cerevisiae* codon-optimized genes as templates. T2OH was PCR amplified from corresponding cloning vector. LEU2 marker was PCR amplified from plasmid PCM217. Then, the secondary fusion PCR of three or two fragments was performed to reduce the number of fragments for transformation. The fused fragments were co-transformed into YT01 by LiAc approach and used to construct the strain YT02 through DNA assembler method. For the construction of strain YT03, the terminator of synTAX19 was changed to PYK1t and other expression modules were removed. For the construction of strain YT04, T2OH and synT7OH expression modules were removed.

(9) For the construction of strain YBDVI, upstream and downstream homologous arms of rDNA site, TDH2, HXT7, PDC1 and PYK1 terminators, and GAL10-GAL1 bidirectional promoter were PCR amplified from *S. cerevisiae* BY4742 genomic DNA. SynAAE4 and synTBT were PCR amplified using corresponding *S. cerevisiae* codon-optimized genes as templates. CYP725A55 and AT5 was PCR amplified from corresponding cloning vector. KanMX marker was PCR amplified from plasmid pLKan. Then, the secondary fusion PCR of three or two fragments was performed to reduce the number of fragments for transformation. The fused fragments were co-transformed into YT02 by LiAc approach and used to construct the strain YDBVI through DNA assembler method. For YTA, YT01, YT02 and YBDVI, 20 colonies were verified by colony PCR and positive colonies were verified by Sanger sequencing.

(10) For the construction of strain YAT5, AT5 was ligated into the pET-28a vector using ClonExpress II One Step Cloning Kit (C112-01, Vazyme Biotech) and then transformed into *E. coli* BL21 (DE3).

Supplementary Note 2. Preparation protocols and characterization data for compounds

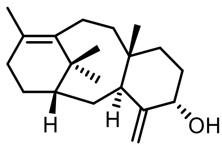

taxa-4(20),11(12)-diene-5α-ol (2)

The compound was obtained via deacetylation of chemical synthesized compound **3** under basic condition, and the stereochemistry of C5 was retained during this procedure. About 27 mg compound **2** was dissolved in $CDCl_3$ and subjected to NMR analysis. All the NMR data were consistent with the literature reported[8].

^{1}H NMR (500 MHz, $CDCl_3$) δ ppm: 4.93 (s, 1H), 4.63 (s, 1H), 4.24 (t, *J* = 2.6 Hz, 1H), 3.31 (d, *J* = 4.6 Hz, 1H), 2.84 (td, *J* = 13.6, 5.2 Hz, 1H), 2.39-2.21 (m, 2H), 2.12-1.96 (m, 3H), 1.88 (ddd, *J* = 18.4, 10.4, 2.6 Hz, 1H), 1.83 (s, 3H), 1.81-1.70 (m, 3H), 1.66-1.52 (m, 2H), 1.34 (s, 3H), 1.29-1.19 (m, 3H), 1.04 (s, 3H), 1.01-0.96 (s, 1H), 0.60 (s, 3H); ^{13}C NMR (125 MHz, $CDCl_3$) δ ppm: 156.04, 136.91, 130.69, 108.77, 74.69, 43.66, 40.18, 40.11, 39.36, 35.56, 32.80, 30.96, 30.30, 30.26, 28.26, 25.59, 24.88, 23.03, 22.24, 21.44; HRMS (ESI) *m/z* calculated for $C_{20}H_{31}$ [M-H_2O+H]$^+$: 271.2420, found:

271.2417.

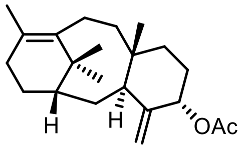

5*α*-acetoxytaxa-4(20),11(12)-diene (3)

The compound was chemically synthesized following the literature reported procedure[9]. In the literature, the stereochemistry of the 5*α*-acetoxy was further confirmed by X-ray of the downstream product. About 8 mg compound **3** was dissolved in $CDCl_3$ and subjected to NMR analysis. All the NMR spectra were consistent with the literature reported spectra[9].

^{1}H NMR (500 MHz, $CDCl_3$) δ ppm: 5.33 (t, *J* = 2.8 Hz, 1H), 5.07 (s, 1H), 4.76 (s, 1H), 3.12 (d, *J* = 4.9 Hz, 1H), 2.81 (td, *J* = 13.4, 5.3 Hz, 1H), 2.42-2.32 (m, 1H), 2.18-1.99 (m, 7H), 1.93-1.87 (m, 1H), 1.85 (s, 3H), 1.82-1.78 (m, 2H), 1.78-1.73 (m, 1H), 1.67-1.61 (m, 1H), 1.60 -1.54 (m, 1H), 1.34 (s, 3H), 1.29-1.21 (m, 2H), 1.09-1.03 (m, 4H), 0.63 (s, 3H); ^{13}C NMR (125 MHz, $CDCl_3$) δ ppm: 170.24, 150.82, 137.44, 130.08, 111.66, 77.36, 43.64, 40.14, 39.81, 39.34, 37.25, 33.44, 30.92, 30.46, 28.44, 28.27, 25.55, 24.90, 22.97, 22.41, 22.06, 21.41; HRMS (ESI) *m/z* calculated for $C_{20}H_{31}$ [M-AcOH+H]$^+$: 271.2420, found: 271.2419.

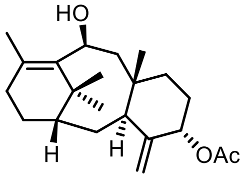

5*α*-acetoxytaxa-4(20), 11(12)-diene-10*β*-ol (4)

Compound **4** was prepared via bioconversion of 5*α*-acetoxytaxa-4(20),11(12)-diene (**3**) with strain YT10OH. 33 mg **3** was added to 2 L medium of strain YT10OH at 24 h, the aqueous medium was extracted with ethyl acetate (2 x 1 L) at 60 h. The combined organic phase was concentrated and the residue was purified on chromatography silica gel (PE/EA = 5/1) to get the crude product which was further purified by prep-HPLC. About 7 mg purified **4** was dissolved in $CDCl_3$ and subjected to NMR analysis. There was a NOE correlation between 3*α*-H (δ 2.85) and 10*α*-H (δ 5.14) which indicated the stereochemistry of the newly introduced C10-hydroxy was in *beta* position. All the NMR spectra were consistent with the literature reported[2].

^{1}H NMR (500 MHz, $CDCl_3$) δ ppm: 5.32 (t, *J* = 2.8 Hz, 5*β*-H), 5.14 (dd, *J* = 11.9, 5.4

Hz, 1H, 10α-H), 5.11 (s, 1H, 20-H), 4.79 (s, 1H, 20-H), 2.85 (d, J = 5.3 Hz, 1H, 3α-H), 2.47-2.37 (m, 1H, 13β-H), 2.28 (dd, J = 14.4, 12.0 Hz, 1H, 9β-H), 2.13-2.03 (m, 1H, 14β-H), 2.07 (s, 3H, 22-H), 2.02-1.87 (m, 2H, 7-H, 13α-H), 1.91 (s, 3H, 18-H), 1.81-1.73 (m, 3H, 6-H, 1β-H), 1.72-1.66 (m, 1H, 2-H), 1.62-1.56 (m, 9α-H, 2-H), 1.52 (s, 3H, 16-H), 1.1.0 (s, 3H, 17-H), 0.70 (s, 3H, 19-H); ^{13}C NMR (125 MHz, $CDCl_3$) δ ppm: 170.04(C21), 150.49(C4), 139.11(C11), 134.77(C12), 112.31(C20), 76.71(C5), 68.26(C10), 47.64(C9), 44.32(C1), 38.95(C15), 36.31(C3), 34.09(C7), 32.31(C17), 30.86(C13), 28.45(C6), 25.86(C16), 23.05(C14), 21.98(C9), 21.94(C22), 21.42(C18); HRMS (ESI) *m/z* calculated for $C_{20}H_{29}$ [M-AcOH-H_2O+H]$^+$: 269.2264, found: 269.2263.

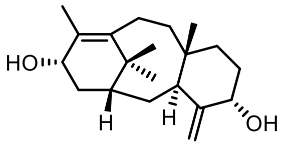

taxa-4(20),11(12)-diene-5α,13α-diol (5)

Compound **5** was prepared via bioconversion of taxa-4(20),11(12)-diene-5α-ol (**2**) with strain YT13OH. 28.8 mg **2** was added to 2 L medium of strain YT13OH at 24 h, and the aqueous medium was extracted with ethyl acetate (2 × 1 L) at 60 h. The combined organic phase was concentrated and the residue was purified on chromatography silica gel (PE/EA = 2/1) to get the crude product which was further purified by prep-HPLC. About 5 mg purified **5** was dissolved in C_6D_6 and subjected to NMR analysis. The spectra of the compound **5** were consistent with the literature reported spectra which suggested the stereochemistry of C5-OH and C13-OH were both in *alpha* position[10].

^{1}H NMR (500 MHz, C_6D_6) δ ppm: 4.92 (s, 1H, 20-H), 4.61 (s, 1H, 20-H), 4.38 (d, J = 9.8 Hz, 1H, 13β-H), 4.11-4.09 (m, 1H, 5β-H), 3.65 (s, 1H, 3α-H), 2.83 (td, J = 13.4, 5.4 Hz, 1H, 10α-H), 2.78-2.73 (m, 1H, 14β-H), 2.35-2.29 (m, 1H, 7α-H), 2.16 (s, 3H, 18-H), 2.07 (d, J = 12.8 Hz, 1H, 10β-H), 1.93-1.87 (m, 1H, 9β-H), 1.64-1.51 (m, 6H, 6-H, 2-H, 13-OH, 14α-H), 1.43 (dd, J = 15.4, 3.5 Hz, 1H, 14β-H), 1.24 (s, 3H, 17-H), 1.13 (ddd, J = 15.2, 5.4, 2.4 Hz, 1H, 14β-H), 0.91-0.90 (m, 1H, 7β-H), 0.88 (s, 3H, 16-H), 0.63 (s, 3H, 19-H); ^{13}C NMR (125 MHz, C_6D_6) δ ppm: 154.77(C4), 139.23(C11), 133.62(C12), 108.64(C20), 74.66(C5), 68.65(C13), 40.63(C8), 40.11(C1), 39.77(C9), 39.44(C15), 36.82(C14), 36.08(C3), 32.85(C7), 32.34(C16), 31.75(C6), 27.90(C2), 25.76(C10), 25.18(C17), 22.49(C19), 16.74(C18); HRMS (ESI) *m/z* calculated for $C_{20}H_{31}O$ [M-H_2O+H]$^+$: 287.2369, found: 287.2364.

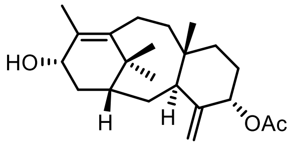

5α-acetoxytaxa-4(20),11(12)-diene-13α-ol (6)

Compound **6** was prepared via bioconversion of taxa-4(20),11(12)-diene-5α-ol (**2**) with strains YT13OH and YT5AT. 57.6 mg **2** was added to 4 L medium of strain YT13OH at 24 h, and the aqueous medium was extracted with ethyl acetate (2 × 2 L) at 60 h. The combined organic phase was concentrated. The resulted residue was dissolved in 20 mL DMSO and directly added to 4 L medium of strain YT5AT at 24 h. The aqueous medium was extracted with ethyl acetate (2 × 2 L) at 60 h. The combined organic phase was concentrated and the resulted residue was purified on chromatography silica gel (PE/EA = 5/1) to get the crude product which was further purified by prep-HPLC. About 10 mg purified **6** was dissolved in $CDCl_3$ and subjected to NMR analysis. The position of the introduced acetyl group was confirmed with 2D-HMBC (δ 170.39/5β-H (δ 5.34)).

^{1}H NMR (500 MHz, $CDCl_3$) δ (ppm): 5.34 (s, 1H, 5β-H), 5.10 (s, 1H, 20-H), 4.80 (s, 1H, 20-H), 4.37 (brs, 1H, 13β-H), 3.30 (s, 1H, 3α-H), 2.89-2.79 (m, 2H, 10α-H and 14β-H), 2.17-2.07 (m, 2H, 7α-H, 10β-H), 2.11 (s, 3H), 2.04 (dd, J = 13.7, 5.4 Hz, 1H, 9α-H), 1.98 (s, 3H, 18-H), 1.89-1.72 (m, 4H, 6-H, 13-OH and 1β-H), 1.66-1.64 (m, 2H, 2-H), 1.37 (s, 3H, 16-H), 1.31-1.26 (m, 1H, 9β-H), 1.15 (dd, J = 15, 4.4 Hz, 1H, 14α-H), 1.10 (dd, J = 13.5, 3.05 Hz, 1H, 7β-H), 0.94 (s, 3H, 17-H), 0.65 (s, 3H, 19-H); ^{13}C NMR (125 MHz, $CDCl_3$) δ ppm: 170.39, 149.61(C4), 139.59(C11), 132.28(C12), 112.71(C20), 77.75(C5), 68.70(C13), 40.14(C8), 39.91(C1), 39.57(C9), 39.42(C15), 38.06(C3), 37.11(C14), 33.47(C7), 31.65(C17), 28.17(C6), 27.95(C2), 25.48(C10), 25.45(C16), 22.45(C19), 21.51, 15.94(C18); HRMS (ESI) *m/z* calculated for $C_{22}H_{33}O_2$ $[M-H_2O+H]^+$: 329.2475, found: 329.2479.

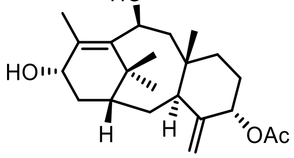

5α-acetoxytaxa-4(20),11(12)-diene-10β,13α-diol (7)

Compound **7** was prepared via bioconversion of taxa-4(20),11(12)-diene-5α-ol (**2**) with strains YT13OH, YT5AT and YT10OH. 86.4 mg **2** was added to 6 L medium of strain YT13OH at 24 h, and the aqueous medium was extracted with ethyl acetate (2 × 3 L) at 60 h. The combined organic phase was concentrated. The resulted residue was

dissolved in 30 mL DMSO and directly added to 6 L medium of strain YT5AT at 24 h. The aqueous medium was extracted with ethyl acetate (2 × 3 L) at 60 h. The combined organic phase was concentrated. The resulted residue was dissolved in 30 mL DMSO and directly added to 6 L medium of strain YT10OH at 24 h. The aqueous medium was extracted with ethyl acetate (2 × 3 L) at 60 h, and the combined organic phase was concentrated. The resulted residue was purified on chromatography silica gel (PE/EA = 2/1) to get the crude product which was further purified by prep-HPLC. About 2.5 mg purified **7** was dissolved in $CDCl_3$ and subjected to NMR analysis. There was a NOE correlation between 10*α*-H (δ 5.14) and 3*α*-H (δ 3.03) which indicated the stereochemistry of C10 hydroxyl group was in *beta* position.

^{1}H NMR (500 MHz, $CDCl_3$) δ ppm: 5.33 (s, 1H, 5*β*-H), 5.18-5.13 (m, 2H, 10*α*-H, 20-H), 4.83 (s, 1H, 20-H), 4.42 (dd, J = 10.3, 3.7 Hz, 1H, 13*β*-H), 3.03 (d, J = 4.7 Hz, 1H, 3*α*-H), 2.91-2.81 (m, 1H, 14*β*-H), 2.29 (dd, J = 14.3, 12.0 Hz, 1H, 9*β*-H), 2.09 (s, 3H, 22-H), 2.05 (s, 3H, 18-H), 1.97 (td, J = 13.3, 4.6 Hz, 1H, 7*α*-H), 1.88-1.74 (m, 2H, 6-H), 1.74-1.64 (m, 3H, 1*β*-H and 2-H), 1.61 (dd, J = 14.4, 5.2 Hz, 1H, 9*α*-H), 1.54 (s, 3H, 16-H), 1.23 (dd, J = 13.3, 2.5 Hz, 1H, 7*β*-H), 1.17 (dd, J = 15.3, 4.7 Hz, 1H, 14*α*-H), 1.00 (s, 3H, 17-H), 0.72 (s, 3H, 19-H); ^{13}C NMR (125 MHz, $CDCl_3$) δ ppm: 170.39(C21), 149.50(C4), 140.75(C11), 136.39(C12), 113.19(C20), 77.37(C5), 68.44(C13), 68.40(C10), 47.32(C9), 40.54(C1), 39.02(C15), 38.39(C8), 36.86(C3), 36.82(C14), 34.14(C7), 32.87(C17), 28.20(C2), 28.12(C6), 25.81(C16), 22.03(C19), 21.48(C22), 16.14(C18); HRMS (ESI) *m/z* calculated for $C_{22}H_{33}O_3$ $[M\text{-}H_2O\text{+}H]^+$: 345.2424, found: 345.2408.

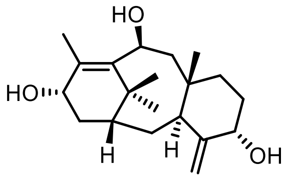

taxa-4(20),11(12)-diene-5*α*,10*β*,13*α*-triol (8)

Compound **8** was prepared via bioconversion of taxa-4(20),11(12)-diene-5*α*-ol (**2**) with strains YT13OH and YT10OH. 57.6 mg **2** was added to 4 L medium of strain YT13OH at 24 h, and the aqueous medium was extracted with ethyl acetate (2 × 2 L) at 60 h. The combined organic phase was concentrated. The resulted residue was dissolved in 20 mL DMSO and directly added to 4 L medium of strain YT10OH at 24 h. The aqueous medium was extracted with ethyl acetate (2 × 2 L) at 60 h. The combined organic phase was concentrated and the resulted was purified on chromatography silica gel (PE/EA = 1/1) to get the crude product which was further purified by prep-HPLC. About 3 mg purified **8** was dissolved in $CDCl_3$ and subjected to NMR analysis. There

was a NOE correlation between 10*α*-H (δ 5.18) and 3*α*-H (δ 3.25) which indicated the stereochemistry of C10 hydroxyl group was in *beta* position.

^{1}H NMR (500 MHz, $CDCl_3$) δ ppm: 5.18 (dd, *J* = 11.7, 5.6 Hz, 1H, 10*α*-H), 5.01 (s, 1H, 20-H), 4.68 (s, 1H, 20-H), 4.35 (d, *J* = 9.0 Hz, 1H, 13*β*-H), 4.29 (brs, 1H, 5*β*-H), 3.28-3.23 (m, 1H, 3*α*-H), 2.87-2.79 (m, 1H, 14*β*-H), 2.22 (dd, *J* = 14.2, 12.0 Hz, 1H, 9*β*-H), 2.11 (td, *J* = 13.4, 5.0 Hz, 1H, 7*α*-H), 2.04 (s, 3H, 18-H), 1.80-1.56 (m, 6H, 2-H, 6-H, 1*β*-H and 9*α*-H), 1.51 (s, 3H, 16-H), 1.29-1.24 (m, 1H, 14*α*-H), 1.17-1.12 (m, 1H, 7*β*-H), 0.96 (s, 3H, 17-H), 0.69 (s, 3H, 19-H); ^{13}C NMR (125 MHz, $CDCl_3$) δ ppm: 153.61(C4), 141.01(C11), 136.82(C12), 109.92 (C20), 74.76(C5), 68.77(C10), 68.70(C13), 47.27(C9), 40.40(C1), 38.95(C15), 38.92(C8), 36.28(C14), 34.77(C3), 33.53(C17), 33.13(C7), 31.42(C6), 27.76 (C6), 25.50(C16), 21.88(C19), 16.79(C18); HRMS (ESI) *m/z* calculated for $C_{20}H_{31}O_2$ $[M-H_2O+H]^+$: 303.2319, found: 303.2309.

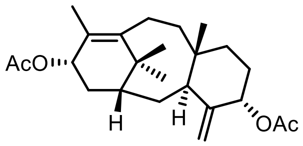

5*α*,13*α*-diacetoxytaxa-4(20),11(12)-diene (9)

Compound **9** was prepared via bioconversion of taxa-4(20),11(12)-diene-5*α*-ol (**2**) with strain YT13OH, YT5AT and YTAX19. 86.4 mg **2** was added to 6 L medium of strain YT13OH at 24 h, and the aqueous medium was extracted with ethyl acetate (2 × 3 L) at 60 h. The combined organic phase was concentrated. The resulted residue was dissolved in 30 mL DMSO and directly added to 6 L medium of strain YT5AT at 24 h. The aqueous medium was extracted with ethyl acetate (2 × 3 L) at 60 h, and the combined organic phase was concentrated. The concentrated residue was dissolved in 30 mL DMSO and directly added to 6 L medium of strain YTAX19 at 24 h. The aqueous medium was extracted with ethyl acetate (2 × 3 L) at 60 h. The combined organic phase was concentrated and the residue was purified on chromatography silica gel (PE/EA = 7/1) to get the crude product which was further purified by prep-HPLC to provide 3 mg 5*α*,13*α*-diacetoxytaxa-4(20),11(12)-diene (**9**). About 3 mg prepared **9** was dissolved in $CDCl_3$ and subjected to NMR analysis.

^{1}H NMR (600 MHz, $CDCl_3$) δ ppm: 5.84 (t, *J* = 7.9 Hz, 1H, 13*α*-H), 5.33 (t, *J* = 2.8 Hz, 1H, 5*β*-H), 5.09 (s, 1H, 20-H), 4.76 (s, 1H, 20-H), 3.18 (d, *J* = 5.7 Hz, 1H, 3*α*-H), 2.88-2.79 (m, 1H, 10*α*-H), 2.17-2.08 (dd, *J* = 14.4, 9.6 Hz, 1H, 14*β*-H), 2.16 (s, 3H, 22-H), 2.17-2.08 (m, 3H, 7*α*-H, 10*β*-H and 9*α*-H), 2.06 (s, 3H), (s, 3H), 1.87 (s, 3H, 18-H), 1.83-1.78 (m, 3H, 1*β*-H and 6-H), 1.68-1.58 (m, 2H, 2-H), 1.42 (s, 3H, 16-H), 1.34-

1.25 (m, 1H, 9*β*-H), 1.13-1.05 (m, 2H, 7-H and 14*α*-H), 1.09 (s, 3H, 17-H), 0.67 (s, 3H, 19-H); ^{13}C NMR (150 MHz, $CDCl_3$) δ ppm: 170.98, 170.40, 150.32(C4), 139.78(C11), 128.00(C12), 112.42(C20), 76.78(C5), 71.56(C13), 40.63(C1), 40.14(C9), 40.08, 40.00, 37.52(C3), 34.18(C7), 32.05(C14), 30.49(C16), 28.62(C2), 28.38(C6), 26.77(C17), 25.34(C10), 22.64(C19), 22.02, 21.75, 14.72(C18); HRMS (ESI) *m/z* calculated for $C_{22}H_{33}O_2$ [M-AcOH+H]$^+$:329.2475, found: 329.2480.

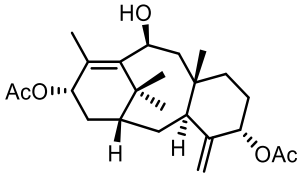

5*α*,13*α*-diacetoxytaxa-4(20),11(12)-diene-10*β*-ol (10)

Compound **10** was prepared via bioconversion of taxa-4(20),11(12)-diene-5*α*-ol (**2**) with strains YT13OH, YT5AT, YTAX19 and YT10OH. 86.4 mg **2** was added to 6 L medium of strain YT13OH at 24 h, and the aqueous medium was extracted with ethyl acetate (2 × 3 L). The combined organic phase was concentrated. The residue was dissolved in 30 mL DMSO and directly added to 6 L medium of strain YT5AT. The aqueous medium was extracted with ethyl acetate (2 × 3 L) at 60 h, and the combined organic phase was concentrated. The concentrated residue was dissolved in 30mL DMSO and directly added to 6 L medium of strain YTAX19. The aqueous medium was extracted with ethyl acetate (2 × 3 L) at 60 h, and the combined organic phase was concentrated. The residue was dissolved in 30mL DMSO and directly added to 6L medium of strain YT10OH. The combined organic phase was concentrated and the residue was purified on chromatography silica gel (PE/EA = 1/1) to get the crude product 5*α*,13*α*-diacetoxytaxa-4(20),11(12)-diene-10*β*-ol (**10**) which was further purified by prep-HPLC. About 8 mg prepared **10** was dissolved in $CDCl_3$ and subjected to NMR analysis. There was a NOE correlation between 10*α*-H (δ 5.14) and 3*α*-H (δ 2.95) which indicated the stereochemistry of C10 hydroxyl group was in *beta* position.

^{1}H NMR (500 MHz, $CDCl_3$) δ ppm: 5.87 (t, *J* = 8.6 Hz, 1H, 13*β*-H), 5.32 (brs, 1H, 5*β*-H), 5.16-5.11 (m, 2H, 10*α*-H, 20-H), 4.78 (brs, 1H, 20-H), 2.95 (d, *J* = 6.2 Hz, 1H, 3*α*-H), 2.71-2.62 (m, 1H, 14*β*-H), 2.36 (dd, *J* = 14.1, 12.0 Hz, 1H, 9*β*-H), 2.13 (s, 3H), 2.05 (s, 3H), 2.03-1.94 (m, 1H, 7*α*-H), 1.92 (s, 3H, 18-H), 1.81-1.76 (m, 3H, 6-H, 1*β*-H), 1.74-1.66 (m, 2H, 2-H), 1.64-1.59 (m, 1H, 9*α*-H), 1.58 (s, 3H, 17-H), 1.24-1.19 (m, 1H, 7*β*-H), 1.14 (s, 3H, 16-Me), 1.09 (dd, *J* = 14.5, 7.5 Hz, 1H, 14*α*-H), 0.73 (s, 3H, 19-H); ^{13}C NMR (125 MHz, $CDCl_3$) δ ppm: 170.76, 170.17, 150.06(C4), 141.12(C11),

132.38(C12), 113.02(C20), 76.60(C5), 71.15(C13), 67.93(C10), 47.66(C9), 41.11(C1), 39.59(C15), 38.21(C8), 36.49(C3), 34.82(C7), 32.22(C14), 31.85(C17), 28.88(C2), 28.29(C6), 26.82(C16), 22.14(C19), 21.90, 21.61, 14.83(C18); HRMS (ESI) *m/z* calculated for $C_{24}H_{35}O_4$ [M-H_2O+H]$^+$: 387.2530, found: 387.2530.

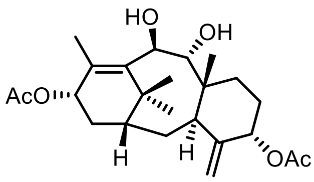

9,10-deacetyltaxusin (11)

Compound **11** was prepared via bioconversion of taxa-4(20),11(12)-diene-5*α*-ol (**2**) with strains YT13OH, YT5AT, YTAX19, YT10OH and YCYP725A37. 86.4 mg **2** was added to 6 L medium of strain YT13OH at 24 h, and the aqueous medium was extracted with ethyl acetate (2 × 3 L) at 60 h. The combined organic phase was concentrated. The concentrated residue was dissolved in 30 mL DMSO and directly added to 6 L medium of strain YT5AT. The aqueous medium was extracted with ethyl acetate (2 × 3 L), and the combined organic phase was concentrated. The concentrated residue was dissolved in 30 mL DMSO and directly added to 6 L medium of strain YTAX19. The aqueous medium was extracted with ethyl acetate (2 × 3 L) at 60 h, and the combined organic phase was concentrated. The residue was dissolved in 30 mL DMSO and directly added to 6 L medium of strain YT10OH. The aqueous medium was extracted with ethyl acetate (2 × 3 L), and the combined organic phase was concentrated. The residue was dissolved in 30 mL DMSO and directly added to 6 L medium of strain YCYP725A37. The aqueous medium was extracted with ethyl acetate (2 × 3 L). The combined organic phase was concentrated and the residue was purified on chromatography silica gel (PE/EA = 1/1) to get the crude product 9,10-deacetyltaxusin (**11**) which was further purified by prep-HPLC. About 2 mg purified **11** was dissolved in $CDCl_3$ and subjected to NMR analysis. There was a strong NOE correlation between 9*β*-H (δ 4.14) and 19-H (δ 0.92) and the spectra of its acetyl product taxusin (**12**) were consistent with literature reported spectra[5], both of those two results indicated the newly introducing C9-OH was in *alpha* position.

^{1}H NMR (600 MHz, $CDCl_3$) δ ppm: 5.89 (t, *J* = 8.1 Hz, 1H, 13*β*-H), 5.35 (t, *J* = 2.4 Hz, 1H, 5*β*-H), 5.19 (s, 1H, 20-H), 4.82 (d, *J* = 1.6 Hz, 1H, 20-H), 4.80 (d, *J* = 9.7 Hz, 1H, 10*α*-H), 4.14 (d, *J* = 9.7 Hz, 1H, 9*β*-H), 2.94 (d, *J* = 4.9 Hz, 1H, 3*α*-H), 2.67 (dt, *J* = 14.5, 9.8 Hz, 1H, 14*β*-H), 2.15 (s, 3H), 2.07 (s, 3H), 1.96 (d, *J* = 1.3 Hz, 3H, 18-H),

1.86-1.81 (m, 2H, 7β-H and 6α-H), 1.81-1.77 (m, 1H, 1β-H), 1.74-1.64 (m, 3H, 6β-H and 2-H), 1.55 (dd, J = 13.0, 4.0 Hz, 1H, 7α-H), 1.52(s, 3H, 16-H), 1.18 (s, 3H, 17-H), 1.55 (dd, J = 14.8, 7.6 Hz, 1H, 14α-H), 0.92 (s, 3H, 19-H); ^{13}C NMR (150 MHz, $CDCl_3$) δ ppm: 170.76, 170.19, 149.55(C4), 138.89(C11), 134.09(C12), 113.70(C20), 78.90C9), 76.91(C5), 72.28(C10), 71.17(C13), 42.93(C8), 40.71(C1), 39.61(C15), 38.15(C3), 32.06(C14), 31.65(C17), 28.36(C2), 27.78(C6), 27.53(C16), 26.25(C7), 21.96, 21.62, 18.24(C19), 15.13(C18); HRMS (ESI) *m/z* $C_{24}H_{35}O_5$ $[M-H_2O+H]^+$: 403.2479, found: 403.2480.

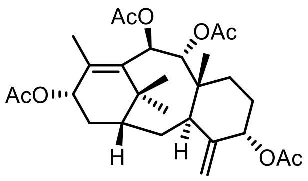

taxusin (12)

Compound **12** was prepared via bioconversion of taxa-4(20),11(12)-diene-5α-ol (**2**) with strains YT13OH, YT5AT, YTAX19, YT10OH, and YCYP725A37. 86.4 mg **2** was added to 6 L medium of strain YT13OH at 24 h, and the aqueous medium was extracted with ethyl acetate (2 × 3 L). The combined organic phase was concentrated. The residue was dissolved in 30 mL DMSO and directly added to 6 L medium of strain YT5AT. The aqueous medium was extracted with ethyl acetate (2 × 3 L), and the combined organic phase was concentrated. The residue was dissolved in 30 mL DMSO and directly added to 6 L medium of strain YTAX19. The aqueous medium was extracted with ethyl acetate (2 × 3 L), and the combined organic phase was concentrated. The residue was dissolved in 30 mL DMSO and directly added to 6 L medium of strain YT10OH. The aqueous medium was extracted with ethyl acetate (2 × 3 L), and the combined organic phase was concentrated. The residue was dissolved in 30 mL DMSO and directly added to 6 L medium of strain YCYP725A37. The aqueous medium was extracted with ethyl acetate (2 × 3 L), and the combined organic phase was concentrated. The residue was dissolved in 30 ml DMSO and directly added to 6 L medium of strain YT5AT. The combined organic phase was concentrated and the residue was purified on chromatography silica gel (PE/EA = 2/1) to get the crude product taxusin (**12**) which was further purified by prep-HPLC. About 5 mg purified **12** was dissolved in $CDCl_3$ and subjected to NMR analysis. The spectra of the biosynthesized taxusin were consistent with literature reported spectra[11]. This result also indicated that the stereochemistries of C9 and C10 in compound **7, 8, 10, 11** were correct.

^{1}H NMR (600 MHz, $CDCl_3$) δ ppm: 6.08 (d, *J* = 10.6 Hz, 1H, 10*α*-H), 5.90-5.84 (m, 2H, 9*β*-H and 13*β*-H), 5.36 (t, *J* = 2.5 Hz, 1H, 5*β*-H), 5.21 (s, 1H, 20-H), 4.85 (s, 1H, 20-H), 3.0 (d, *J* = 6.1 Hz, 1H, 3*α*-H), 2.66 (dt, *J* = 14.6, 9.8 Hz, 1H, 14*β*-H), 2.16 (s, 3H), 2.11 (s, 3H, 18-H), 2.07 (s, 3H), 2.05 (s, 3H), 2.01 (s, 3H), 1.89-1.65 (m, 7H, 1*β*-H, 2-H, 6-H and 7-H), 1.62(s, 3H, 16-H), 1.11 (s, 3H, 17-H), 1.07 (dd, *J* = 14.4, 7.5 Hz, 1H, 14*α*-H), 0.75 (s, 3H, 19-H); ^{13}C NMR (150 MHz, $CDCl_3$) δ ppm: 170.57, 170.55, 170.12, 170.08, 148.94(C4), 137.16(C12), 135.00(C11), 114.22(C20), 77.61(C9), 76.47(C5), 72.68(C10), 43.07(C8), 40.40(C1), 39.42(C15), 38.14(C3), 32.06(C14), 31.26(C17), 28.46, 27.50, 27.46, 27.42(C16), 21.94, 21.58, 21.19, 21.00, 17.88(C19), 14.99(C18); HRMS (ESI) *m/z* calculated for $C_{28}H_{44}NO_8$ $[M+NH_4]^+$: 522.3061, found: 522.3062.

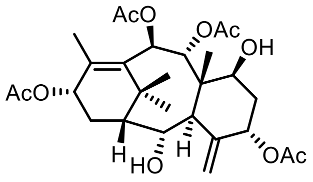

2*α*,7*β*-dihydroxytaxusin (13)

Compound **13** was prepared via bioconversion of taxa-4(20),11(12)-diene-5*α*-ol (**2**) with strains YT13OH, YT5AT, YTAX19, YT10OH, YCYP725A37,YT5AT, YT2OH and YT7OH. 144 mg **2** was added to 10 L medium of strain YT13OH at 24 h, and the aqueous medium was extracted with ethyl acetate (2 × 5 L). The combined organic phase was concentrated, the concentrated residue was dissolved in 50 mL DMSO and directly added to 10 L medium of strain YT5AT. The aqueous medium was extracted with ethyl acetate (2 × 5 L), and the combined organic phase was concentrated. The residue was dissolved in 50 mL DMSO and directly added to 10 L medium of strain YTAX19. The aqueous medium was extracted with ethyl acetate (2 × 5 L), and the combined organic phase was concentrated. The residue was dissolved in 50 mL DMSO and directly added to 10 L medium of strain YT10OH. The aqueous medium was extracted with ethyl acetate (2 × 5 L), and the combined organic phase was concentrated. The residue was dissolved in 50 mL DMSO and directly added to 10 L medium of strain YCYP725A37. The aqueous medium was extracted with ethyl acetate (2 × 5 L), and the combined organic phase was concentrated. The residue was dissolved in 50 mL DMSO and directly added to 10L medium of strain YT5AT. The aqueous medium was extracted with ethyl acetate (2 × 5 L), and the combined organic phase was concentrated. The residue was dissolved in 50 mL DMSO and directly added to 10 L medium of strain YT2OH. The aqueous medium was extracted with ethyl acetate (2 × 5 L), and the

combined organic phase was concentrated. The residue was dissolved in 50 mL DMSO and directly added to 10 L medium of strain YT7OH. The aqueous medium was extracted with ethyl acetate (2 × 5 L), the combined organic phase was concentrated and the residue was purified on chromatography silica gel (EA) to get the crude product taxusin-2*α*,7*β*-diol (**13**) which was further purified by prep-HPLC. About 10 mg purified **13** was dissolved in $CDCl_3$ and subjected to NMR analysis. The stereochemistry of newly introduced C7-hydroxy were confirmed by 2D-NOE (7*α*-H (δ 4.23)/3*α*-H (δ 2.96)), there is a strong NOE correlation between 7*α*-H (δ 4.23)/10*α*-H (δ 6.18) implied the stereochemistry of C10-hydroxy was resistant and still in *beta* position. The NOE correlation of 2*β*-H (δ 4.26)/19-H (δ 0.97) indicated the hydroxyl group in C2 was in *alpha* position.

^{1}H NMR (500 MHz, $CDCl_3$) δ ppm: 6.18 (d, *J* = 11.0 Hz, 1H, 10*α*-H), 6.04-6.00 (m, 1H, 13*β*-H), 6.00 (d, *J* = 11.0 Hz, 1H, 9*β*-H), 5.50 (d, *J* = 1.0 Hz, 1H, 20-H), 5.47 (s, 1H, 20-H), 5.30 (s, 1H, 5*β*-H), 4.27-4.21 (m, 2H, 2*β*-H and 7*α*-H), 2.95 (d, *J* = 6.4 Hz, 1H, 3*α*-H), 2.60-2.52(m, 1H, 14*β*-H), 2.20 (s, 3H), 2.17-2.12 (m, 2H, 1*β*-H and 6*α*-H), 2.10 (s, 3H), 2.09 (s, 6H, 18-H and 13*α*-acetoxyl-H), 2.01 (s, 3H), 1.72 (s, 3H, 16-H), 1.65-1.62 (m, 1H, 6*β*-H), 1.35-1.30 (m, 1H, 14*α*-H), 1.21(s, 3H, 17-H), 0.97 (s, 3H, 19-H); ^{13}C NMR (125 MHz, $CDCl_3$) δ ppm: 170.55, 170.00, 169.75, 168.94, 141.62(C4), 137.44(C12), 132.99(C11), 120.80(C20), 78.45(C9), 76.98(C5), 71.40(C10), 70.66(C13), 70.60(C7), 69.68(C2), 51.86(C1), 48.03(C8), 44.21(C3), 37.69(C15), 37.03(C6), 31.98(C17), 28.00(C14), 27.57(C16), 21.92, 21.57, 21.08, 20.88, 15.23(C18), 13.00(C19). HRMS (ESI) *m/z* calculated for $C_{28}H_{44}NO_{10}$ $[M+NH_4]^+$: 554.2960, found: 554.2961.

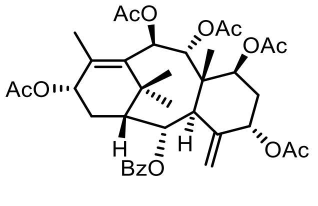

2*α*-benzoyloxy-7*β*-acetoxytaxusin (15)

Compound **15** was prepared via bioconversion of taxa-4(20),11(12)-diene-5*α*-ol (**2**) with strains YT13OH, YT5AT, YTAX19, YT10OH, YCYP725A37,YT5AT, YT2OH, and YT7OH as well as in vitro catalytic reaction of YTBT and chemical synthesis. According to the above protocol (biosynthesis of compound **13** from compound **2**), crude product taxusin-2*α*,7*β*-diol (**13**) was obtained after chromatography silica gel purification, and then incubated with 1 L cured enzyme of

YAAE, 2.5 mM $MgCl_2$, 2.5mM ATP, 0.4 mM benzoic acid, and 0.4 mM coenzyme A. The aqueous medium was extracted with ethyl acetate (2 × 1 L), and the combined organic phase was concentrated and purified on chromatography silica gel (EA) to give the crude product 2α-benzoyloxy-taxusin-7*β*-ol (**14**). The crude product was dissolved in acetic anhydride (2.0 mL) and pyridine (2.0 mL) and stirred overnight, the reaction mixture was concentrated and purified on chromatography silica gel (EA) to give the crude product 2*α*-benzoyloxy-7*β*-acetoxytaxusin (**15**) which was further purified by prep-HPLC. About 2 mg purified **15** was dissolved in $CDCl_3$ and subjected to NMR analysis. The stereochemistry of C2 and C7 were confirmed by 2D-NOE (7*α*-H (δ 5.42)/3*α*-H (δ 3.35), 2*β*-H (δ 5.80)/1*β*-H (δ 2.14)).

^{1}H NMR (600 MHz, $CDCl_3$) δ ppm: 8.00-7.96 (m, 2H), 7.59-7.55 (m, 1H), 7.46-7.42 (m, 2H), 6.22 (d, *J* = 10.6 Hz, 1H, 10*α*-H), 6.04-5.97 (m, 2H, 9*β*-H and 13*β*-H), 5.80 (dd, *J* = 7.0, 2.2 Hz, 1H, 2*β*-H), 5.42 (dd, *J* = 11.4, 5.4 Hz, 1H, 7*α*-H), 5.37 (s, 1H, 20-H), 5.31 (t, *J* = 4.6 Hz, 1H, 5*β*-H), 4.83 (s, 1H, 20-H), 3.35 (d, *J* = 6.8 Hz, 1H, 3*α*-H), 2.56(dt, *J* = 14.8, 9.4 Hz, 1H, 14*β*-H), 2.27 (s, 3H), 2.24 (s, 3H, 18-H), 2.16-2.12 (m, 1H, 1*β*-H), 2.14 (s, 3H), 2.06 (s, 3H), 2.05 (s, 3H), 2.01 (s, 3H), 2.02-1.96 (m, 1H, 6*α*-H), 1.86 (s, 3H, 16-H), 1.82-1.74 (m, 1H, 6*β*-H), 1.63-1.59 (m, 1H, 14*α*-H), 1.19(s, 3H, 17-H), 1.06 (s, 3H, 19-H); ^{13}C NMR (150 MHz, $CDCl_3$) δ ppm: 170.65, 170.10, 170.00 (two carbonyl carbons), 169.46, 165.05, 139.72(C4), 137.60(C12), 133.49, 133.17(C11), 130.03, 129.99, 128.69, 120.13(C20), 76.37(C5), 76.07(C9), 71.64(C10), 71.36(C2), 70.56(C13), 70.07(C7), 49.21(C1), 47.46(C8), 43.36(C3), 37.81(C15), 35.22(C6), 31.77(C17), 28.13(C14), 27.60(C16), 22.02, 21.68, 21.52, 21.16, 20.96, 15.45(C18), 13.96(C19); HRMS (ESI) *m/z* calculated for $C_{37}H_{50}NO_{12}$ $[M+NH_4]^+$: 700.3328, found: 700.3328.

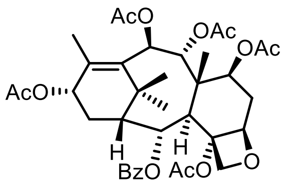

1*β*-dehroxybaccatin VI (16)

Compounds **16** and **17** was prepared via bioconversion of taxa-4(20),11(12)-diene-5*α*-ol (**2**) with strains YT13OH, YT5AT, YTAX19, YT10OH, YCYP725A37,YT5AT, YT2OH, YT7OH and YCYP725A55 as well as in vitro catalytic reaction of YTBT and chemical synthesis. According to the above protocol (biosynthesis of compound **13** from compound **2**), crude product taxusin-2*α*,7*β*-diol (**13**)

was obtained after chromatography silica gel purification, and then incubated with 1L cured enzyme of YAAE, 2.5 mM $MgCl_2$, 2.5 mM ATP, 0.4 mM benzoic acid, and 0.4 mM coenzyme A. The aqueous medium was extracted with ethyl acetate (2 × 1 L), and the combined organic phase was concentrated and purified on chromatography silica gel (EA) to give the crude product 2α-benzoyloxy-taxusin-7*β*-ol (**14**). The crude product was dissolved in acetic anhydride (2.0 mL) and pyridine (2.0 mL) and stirred overnight, the reaction mixture was concentrated and purified on chromatography silica gel (EA) to give the crude product 2*α*-benzoyloxy-7*β*-acetoxytaxusin (**15**). The crude product **15** was dissolved in 10 mL DMSO and directly added to 2 L medium of strain YCYP725A55. The aqueous medium was extracted with ethyl acetate (2 × 1 L). The combined organic phase was concentrated and the residue was purified on chromatography silica gel (EA) and the resulted crude product was further purified by prep-HPLC to obtain 1*β*-dehroxybaccatin VI (**16**) and 2*α*-deacetyl-2*α*-benzoylbaccatin I (**17**). About 0.8 mg prepared **16** was dissolved in $CDCl_3$ and subjected to NMR analysis. The spectrum of the biosynthesized compound **16** were consistent with the literature reported[12].

^{1}H NMR (600 MHz, $CDCl_3$) δ ppm: 8.09-8.06 (m, 2H), 7.62-7.58 (m, 1H), 7.50-7.44 (m, 2H), 6.18 (d, J = 11.0 Hz, 1H, 10*α*-H), 6.00 (d, J = 11.0 Hz, 1H, 9*β*-H), 5.96-5.92 (m, 1H, 13*β*-H), 5.86 (d, J = 5.4 Hz, 1H, 2*β*-H), 5.58-5.54 (m, 1H, 7*α*-H), 5.00 (d, J = 9.0 Hz, 1H, 5*α*-H), 4.38 (d, J = 8.2 Hz, 1H, 20*a*-H), 4.13 (d, J = 8.2 Hz, 1H, 20*β*-H), 3.01 (d, J = 5.6 Hz, 1H, 3*α*-H), 2.55-2.49 (m, 1H, 6*α*-H), 2.49-2.42(m, 1H, 14*β*-H), 2.28 (s, 3H), 2.19 (s, 3H), 2.10 (s, 3H), 2.09 (s, 3H), 2.04 (s, 3H), 1.98(s, 3H), 1.92-1.86 (m, 1H, 6*β*-H), 1.86 (s, 3H), 1.68 (dd, J = 15.0, 7.0 Hz, 1H, 14*α*-H), 1.59 (s, 3H), 1.14 (s, 3H); ^{13}C NMR (150 MHz, $CDCl_3$) δ ppm: 170.80, 170.35, 170.05, 169.17 (two carbons), 165.03, 139.02(C12), 133.71, 133.41(C11), 129.94, 129.76, 128.77, 83.93(C5), 81.42(C4), 76.70(C20), 75.55(C9), 72.10(C7), 71.52(C2), 71.13(C10), 69.11(C13), 47.29(C1), 45.86(C8), 44.54(C3), 38.03(C15), 34.78(C6), 31.50 (C17), 27.09(C16), 26.69(C14), 22.84, 21.58, 21.39, 21.13, 20.97, 15.11(C18) , 12.96(C19); HRMS (ESI) *m/z* calculated for $C_{37}H_{50}NO_{13}$ $[M+NH_4]^+$: 716.3276, found: 716.3285.

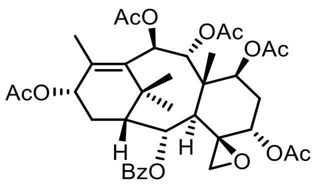

2*α*-deacetyl-2*α*-benzoylbaccatin I (17)

The 2α-deacetyl-2α-benzoylbaccatin I (**17**) was synthesized as above protocol (synthesis of compound **16** from **2**). About 0.6 mg purified **17** was dissolved in $CDCl_3$ and subjected to NMR analysis. From the following figure, 5β-H(δ 4.20) and 20b-H (δ 2.26) has middle NOE correlation (The distance of the two hydrogen is calculated as 2.41 Å).The distance of 20a-H (δ 3.58) and 3α-H (δ 3.08) is about 3.19 Å, and these two hydrogens have a weak NOE correlation. The 20a-H (δ 3.58) also shows strong NOE correlations with 14α-H (δ 1.66) and Ph-H (δ 7.94). All those NOE correlations indicated the newly introduced C4(20) epoxide was in *beta* position.

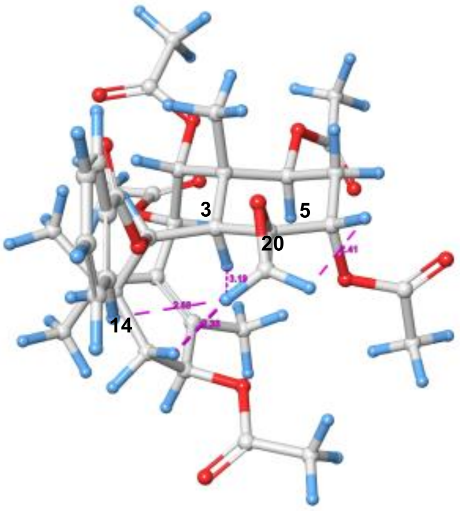

^1H NMR (500 MHz, $CDCl_3$) δ ppm: 7.96-7.92 (m, 2H), 7.57-7.53 (m, 1H), 7.48-7.40 (m, 2H), 6.24 (d, J = 11.0 Hz, 1H, 10α-H), 6.12 (d, J = 11.0 Hz, 1H, 9β-H), 5.93 (t, J = 8.0 Hz, 1H, 13β-H), 5.84 (d, J = 3.0 Hz, 1H, 2β-H), 5.54 (dd, J = 12.0, 4.4 Hz, 1H, 7α-H), 4.20 (t, J = 2.8 Hz, 1H, 5β-H), 3.58 (d, J = 5.2 Hz, 1H, 20α-H), 3.08 (d, J = 3.5 Hz, 1H, 3α-H), 2.41 (dt, J = 14.8, 9.4 Hz, 1H, 14β-H), 2.28 (s, 3H, 18-H), 2.26 (d, J = 5.2 Hz, 1H, 20β-H), 2.19-2.11 (m, 1H, 6β-H), 2.23 (s, 3H), 2.15 (s, 3H), 2.10 (s, 3H), 2.08 (s, 3H), 2.00 (s, 3H), 1.95 (d, J = 8.8 Hz, 1H, 1β-H), 1.78 (s, 3H, 16-H), 1.75-1.70 (m, 1H, 6α-H), 1.66-1.60 (m, 1H, 14α-H), 1.36 (s, 3H, 19-H), 1.16 (s, 3H, 17-H); ^{13}C NMR (125 MHz, $CDCl_3$) δ ppm: 170.48, 170.13, 169.66, 169.46, 169.44, 164.78, 138.27(C12), 134.70(C11), 133.16, 130.50, 129.56, 128.67, 77.42(C5), 75.72(C9), 71.39(C10), 71.37(C2), 70.52(C13), 69.14(C7), 58.88(C4), 49.94(C20), 48.10(C1), 46.86(C8), 39.44(C3), 38.66(C15), 31.58(C17), 31.39(C6), 29.09(C14), 27.25(C16), 21.83, 21.72, 21.63, 21.15, 20.90, 15.57(C18) , 14.15(C19); HRMS (ESI) *m/z* calculated for $C_{37}H_{50}NO_{13}$ $[M+NH_4]^+$: 716.3276, found: 716.3284.

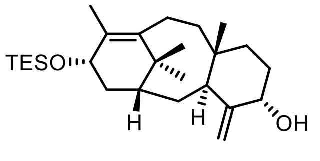

13α-triethylsiloxytaxa-4(20), 11(12)-diene-5α-ol (29)

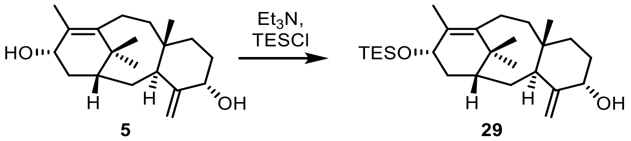

Triethyl amine (0.69 mL, 10.2 mmol), triethylchlorosilane (0.43 mL, 2.3 mmol) was dropped to the solution of taxa-4(20),11(12)-diene-5α,13β-diol (**5**) (650 mg, 2.1 mmol) in dichloromethane (15 mL) at 0 °C, subsequently. Then DMAP (131 mg, 1.0 mmol) was added in one portion and the resulted reaction system was stirred at room temperature with 2 h. Then water was added to quench the reaction and separated. The aqueous phase was extracted with dichloromethane (3 × 30 mL), the combined organic phase was washed with bine, dried with sodium sulfate, filtered and concentrated in vacuum. The resulted residue was purified on chromatography silica gel (PE/EA = 20/1) to get the compound **29** (300 mg, 34% yield). About 9 mg of chemical synthesized compound **29** was dissolved in $CDCl_3$ and subjected to NMR analysis.

^{1}H NMR (400 MHz, $CDCl_3$) δ ppm: 5.02 (brs, 1H, 5*β*-H), 4.67 (brs, 1H, 20-H), 4.49-4.42 (m, 1H, 13*β*-H), 4.23 (brs, 1H, 20-H), 3.62 (brs, 1H), 2.95-2.85 (m, 1H), 2.75-2.63 (m, 1H), 2.36-2.28 (m, 1H), 2.20-2.12 (m, 1H), 2.04-1.92 (m, 1H), 1.96 (s, 3H), 1.85-1.76 (m, 1H), 1.71-1.60 (m, 4H), 1.37 (s, 3H), 1.33-1.22 (m, 2H), 1.04-0.94 (m, 2H), 0.98 (t, *J* = 7.8 Hz, 9H), 0.92 (s, 3H), 0.70-0.61 (m, 6H), 0.63 (s, 3H); ^{13}C NMR (100 MHz, $CDCl_3$) δ ppm: 154.44, 139.16, 132.96, 109.20, 74.67, 69.27, 40.59, 39.90, 39.33 (two carbons), 37.22, 35.40, 32.63, 32.07, 30.80, 27.55, 25.56, 25.25, 22.26, 16.72, 7.07, 4.91.

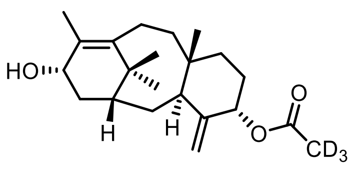

5α-trideuterated acetoxytaxa-4(20),11(12)-diene (18)

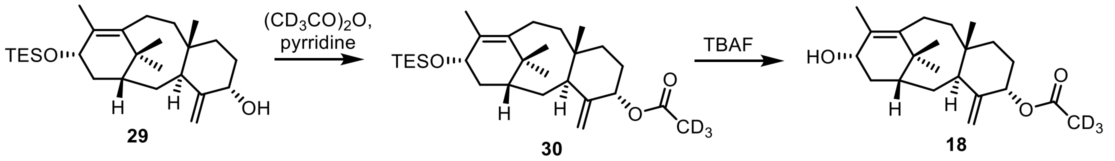

The mixture of compound **29** (300mg, 0.72 mmol), pyridine (2 mL) and D6-acetic anhydride (2 mL) was stirred at room temperature overnight and then concentrated in vacuum, the residue was purified on chromatography silica gel (PE/EA = 20/1) to provide the compound **30** (250 mg).

Tetrabutylammonium fluoride (1.0 mL, 1 M solution in THF, 1 mmol) was dropped to the solution of compound **30** (240 mg, 0.5 mmol) in THF (5 mL) at room temperature and the reaction system was stirred with 1 h and quenched with water (10 mL). The organic phase was separated and the aqueous phase was extracted with EA (3 x 20 mL), the combined organic phase was washed with brine, dried with sodium sulfate, filtered and concentrated in vacuum. The resulted residue was purified on chromatography silica gel (PE/EA = 7/1) to obtain the desired deuterated substrate **18** as colorless solid (150 mg, 67% yield for two steps). About 14 mg of chemical synthesized compound **18** was dissolved in $CDCl_3$ and subjected to NMR analysis. About 14 mg of chemical synthesized compound **18** and 2 μL $CDCl_3$ were dissolved in $CHCl_3$ and subjected to ^{2}H NMR analysis.

^{1}H NMR (400 MHz, $CDCl_3$) δ ppm: 5.33 (brs, 1H, 5*β*-H), 4.09 (brs, 1H, 20-H), 4.79 (s, 1H, 13*β*-H), 4.35 (brs, 1H,20-H), 3.29 (brs, 1H), 2.91-2.75(m, 2H), 2.20-1.99 (m, 3H), 1.96 (s, 3H), 1.90-1.68 (m, 4H), 1.66-1.61(m, 2H), 1.35 (s, 3H), 1.32-1.24 (m, 1H), 1.16-1.04 (m, 1H), 0.92 (s, 3H), 0.64 (s, 3H); ^{13}C NMR (150 MHz, CDCl3) δ ppm: 170.12, 149.34, 139.19, 112.37, 74.74, 68.34, 39.83, 39.64, 39.30, 39.12, 37.72, 36.75, 33.18, 31.33, 27.90, 27.68, 25.23, 25.16, 22.18, 15.63; ^{2}H NMR (64 MHz, $CHCl_3$) δ ppm: 2.08; HRMS (ESI) *m/z* calculated for $C_{22}H_{30}D_3O_2$ $[M-H_2O+H]^+$: 332.2663, found: 332.2664.

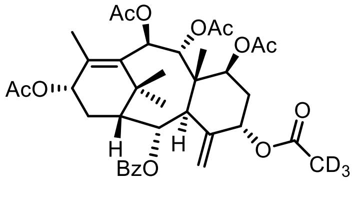

2*α*-benzoyloxy-7*β*-acetoxy-5*α*-trideuterated acetyl-taxusin (20)

Deuterated compound **20** was prepared as the same protocol for the synthesis of compound **15** by using 5*α*-trideuterated acetoxytaxa-4(20),11(12)-diene (**18**) as starting material. About 1 mg purified **20** was dissolved in $CDCl_3$ and subjected to NMR analysis. About 1 mg of chemical synthesized compound 20 and 2 μL $CDCl_3$ were dissolved in $CHCl_3$ and subjected to ^{2}H NMR analysis.

^{1}H NMR (500 MHz, $CDCl_3$) δ ppm: 8.00-7.96 (m, 2H), 7.59-7.55 (m, 1H), 7.46-7.42 (m, 2H), 6.22 (d, *J* = 10.6 Hz, 1H, 10*α*-H), 6.04-5.97 (m, 2H, 9*β*-H and 13*β*-H), 5.80 (dd, *J* = 7.0, 2.2 Hz, 1H, 2*β*-H), 5.42 (dd, *J* = 11.4, 5.4 Hz, 1H, 7*α*-H), 5.37 (s, 1H, 20-H), 5.31 (t, *J* = 4.6 Hz, 1H, 5*β*-H), 4.83 (s, 1H, 20-H), 3.35 (d, *J* = 6.8 Hz, 1H, 3*α*-H), 2.56(dt, *J* = 14.8, 9.4 Hz, 1H, 14*β*-H), 2.24 (s, 3H, 18-H), 2.16-2.12 (m, 1H, 1*β*-H), 2.14 (s, 3H), 2.12 (s, 3H), 2.07 (s, 3H), 2.05 (s, 3H), 2.01 (s, 3H), 2.02-1.96 (m, 1H, 6*α*-H), 1.86 (s, 3H, 16-H), 1.82-1.74 (m, 1H, 6*β*-H), 1.63-1.59 (m, 1H, 14*α*-H), 1.19(s, 3H, 17-H), 1.06 (s, 3H, 19-H); ^{13}C NMR (150 MHz, $CDCl_3$) δ ppm: 170.65, 17.12, 170.01 (two carbonyl carbons), 169.47, 165.06, 139.72(C4), 137.60(C12), 133.49, 133.18(C11), 130.04, 129.99, 128.69, 120.14(C20), 76.35(C5), 76.07(C9), 71.64(C10), 71.36(C2), 70.56(C13), 70.08(C7), 49.21(C1), 47.47(C8), 43.36(C3), 37.82(C15), 35.23(C6), 31.78(C17), 28.14(C14), 27.60(C16), 21.68, 21.53, 21.16, 20.96, 15.44(C18), 13.97(C19); ^{2}H NMR (77 MHz, $CHCl_3$) δ ppm: 2.25; HRMS (ESI) *m/z* calculated for $C_{37}H_{47}D_3NO_{12}$ $[M+NH_4]^+$: 703.3516, found: 703.3522.

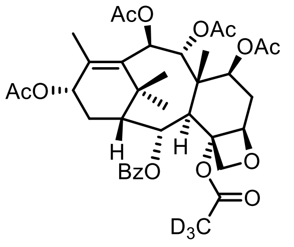

1*β*-dehydroxy-4α-trideuterated acetyl-baccatin VI (21)

Deuterated compound **21** was prepared as the same protocol for the synthesis of compound **16** by using the 5*α*-trideuterated acetoxytaxa-4(20),11(12)-diene (**18**) as starting material. About 0.7 mg prepared **21** was dissolved in $CDCl_3$ and subjected to NMR analysis. About 0.7 mg of chemical synthesized compound 20 and 2 μL $CDCl_3$ were dissolved in $CHCl_3$ and subjected to ^{2}H NMR analysis.

^{1}H NMR (600 MHz, $CDCl_3$) δ ppm: 8.09-8.06 (m, 2H), 7.62-7.58 (m, 1H), 7.50-7.44 (m, 2H), 6.19 (d, *J*= 11.2 Hz, 10*α*-H), 6.00 (d, *J*= 11.2 Hz, 9*β*-H), 5.97-5.92 (m, 1H, 13*β*-H), 5.86 (d, *J*= 5.4 Hz, 2*β*-H), 5.58-5.54 (m, 7*α*-H), 5.00 (d, *J*= 9.0 Hz, 5*α*-H), 4.38 (d, *J*= 8.2 Hz, 1H, 20*α*-H), 4.13 (d, *J*= 8.2 Hz, 1H, 20*β*-H), 3.01 (d, *J*= 5.6 Hz, 3*α*-H), 2.55-2.49 (m, 6*α*-H), 2.49-2.42(m, 14*β*-H), 2.28 (s, 3H), 2.19 (s, 3H), 2.10 (s, 6H), 2.04 (s, 3H, 18-H), 1.98(s, 3H), 1.92-1.86 (m, 6*β*-H), 1.86 (s, 3H, 16-H), 1.68 (dd, *J*= 15.0, 7.0 Hz, 14*α*-H), 1.59 (s, 3H, 19-H), 1.14 (s, 3H, 17-H); ^{13}C NMR (150 MHz, $CDCl_3$) δ ppm: 170.81, 170.36, 170.07, 169.21, 165.04, 139.02(C12), 133.72, 133.42(C11), 129.94, 129.76, 128.78, 83.94(C5), 81.41(C4), 76.70(C20), 75.56(C9), 72.12(C7),

71.53(C2), 71.14(C10), 69.11(C13), 47.29(C1), 45.86(C8), 44.55(C3), 38.03(C15), 34.78(C6), 31.50 (C17), 27.10(C16), 26.69(C14), 21.59, 21.39, 21.13, 20.97, 15.12(C18) , 12.97(C19); ^{2}H NMR (77 MHz, $CHCl_3$) δ ppm: 2.26; HRMS (ESI) *m/z* calculated for $C_{37}H_{47}D_3NO_{13}$ $[M+NH_4]^+$: 719.3465, found: 716.3463.

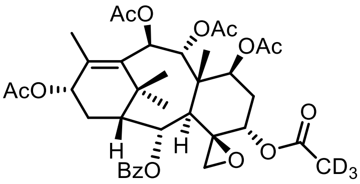

2α-deacetyl-2α-benzoyl-5α-trideuterated acetylbaccatin I (22)

Deuterated compound **22** was prepared as the same protocol for the synthesis of compound **16** by using the 5α-trideuterated acetoxytaxa-4(20),11(12)-diene (**18**) as starting material. About 0.8 mg prepared **22** was dissolved in $CDCl_3$ and subjected to NMR analysis. About 0.8 mg of chemical synthesized compound **22** and 2 μL $CDCl_3$ were dissolved in $CHCl_3$ and subjected to ^{2}H NMR analysis.

^{1}H NMR (500 MHz, $CDCl_3$) δ ppm: 7.96-7.92 (m, 2H), 7.57-7.53 (m, 1H), 7.48-7.40 (m, 2H), 6.24 (d, *J* = 11.0 Hz, 1H, 10α-H), 6.12 (d, *J* = 11.0 Hz, 1H, 9β-H), 5.93 (t, *J* = 8.0 Hz, 1H, 13β-H), 5.84 (d, *J* = 3.0 Hz, 1H, 2β-H), 5.54 (dd, *J* = 12.0, 4.4 Hz, 1H, 7α-H), 4.20 (t, *J* = 2.8 Hz, 1H, 5β-H), 3.58 (d, *J* = 5.2 Hz, 1H, 20α-H), 3.08 (d, *J* = 3.5 Hz, 1H, 3α-H), 2.41 (dt, *J* = 14.8, 9.4 Hz, 1H, 14β-H), 2.28 (s, 3H, 18-H), 2.26 (d, *J* = 5.2 Hz, 1H, 20β-H), 2.19-2.11 (m, 1H, 6β-H), 2.15 (s, 3H), 2.10 (s, 3H), 2.08 (s, 3H), 2.00 (s, 3H), 1.95 (d, *J* = 8.8 Hz, 1H, 1β-H), 1.78 (s, 3H, 16-H), 1.75-1.70 (m, 1H, 6α-H), 1.66-1.60 (m, 1H, 14α-H), 1.36 (s, 3H, 19-H), 1.16 (s, 3H, 17-H); ^{13}C NMR (125 MHz, $CDCl_3$) δ ppm: 170.48, 170.13, 169.66, 169.46, 164.78, 138.27(C12), 134.71(C11), 133.16, 130.50, 129.56, 128.68, 77.50(C5), 75.72(C9), 71.39(C10), 71.39(C2), 70.52(C13), 69.14(C7), 58.88(C4), 49.94(C20), 48.09(C1), 46.86(C8), 39.43(C3), 38.66(C15), 31.58(C7), 31.39(C6), 29.09(C14), 27.25(C16), 21.71, 21.62, 21.14, 20.89, 15.07, 15.56(C18) , 14.14(C19); ^{2}H NMR (77 MHz, $CHCl_3$) δ ppm: 2.21; HRMS (ESI) *m/z* calculated for $C_{37}H_{47}D_3NO_{13}$ $[M+NH_4]^+$: 719.3465, found: 716.3464.

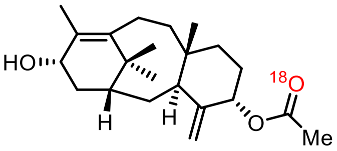

^{18}O labeled 5α-acetoxytaxa-4(20),11(12)-diene-13α-ol (23)

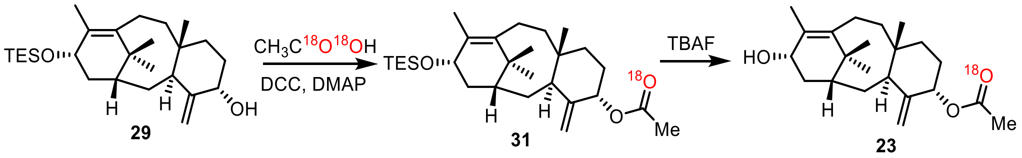

To the solution of **29** (1.2g, 2.87 mmol) and acetic acid-$^{18}O_2$ (732mg, 4.6 mM) in anhydrous dichloromethane (16 mL), triethyl amine (2.0 mL, 14 mmol), 1,3-dicyclohexylcarbodiimide (1.18g, 5.74 mmol), 4-dimethylaminopyridine (350.6 mg, 2.87 mmol) were added sequentially at room temperature. The resulted reaction mixture was stirred at room temperature with 2 days and then quenched by water (30 mL). The organic phase was separated and the aqueous phase was extracted with dichloromethane (3× 40 mL). The combined organic phase was washed with brine, dried with anhydrous sodium sulfate, filtered concentrated in vacuum. The resulted residue was purified on silica chromatography gel (PE/EA = 15/1) to obtain the inseparable mixture of **29** and **31** (1.2 g).

The mixture of **29** and **31** was dissolved in anhydrous THF (10 mL) and Tetrabutylammonium fluoride (10.0 mL, 1M solution in THF, 10.0 mmol) was dropped at room temperature. The reaction system was stirred at room temperature with 1 h and quenched with water (20 mL). The organic phase was separated and the aqueous phase was extracted with ethyl acetate (3 x 20 mL), the combined organic phase was washed with brine, dried with sodium sulfate, filtered and concentrated in vacuum. The resulted residue was purified on chromatography silica gel (PE/EA = 7/1) to obtain the desired ^{18}O labeled substrate **23** as colorless solid (422 mg, 42% yield for two steps). About 6 mg of chemical synthesized compound **23** was dissolved in $CDCl_3$ and subjected to NMR analysis.

^1H NMR (400 MHz, $CDCl_3$) δ (ppm): 5.34 (s, 1H, 5*β*-H), 5.10 (s, 1H, 20-H), 4.81 (s, 1H, 20-H), 4.37 (brs, 1H, 13*β*-H), 3.31 (s, 1H, 3*α*-H), 2.90-2.79 (m, 2H, 10*α*-H and 14*β*-H), 2.17-2.01 (m, 3H, 7*α*-H, 10*β*-H and 9*α*-H), 2.11 (s, 3H), 1.98 (s, 3H, 18-H), 1.89-1.72 (m, 4H, 6-H, 13-OH and 1*β*-H), 1.66-1.64 (m, 2H, 2-H), 1.38 (s, 3H, 16-H), 1.31-1.26 (m, 1H, 9*β*-H), 1.15 (dd, *J* = 15.0, 4.4 Hz, 1H, 14*α*-H), 1.12-1.07 (m, 1H, 7*β*-H), 0.94 (s, 3H, 17-H), 0.65 (s, 3H, 19-H); ^{13}C NMR (100 MHz, $CDCl_3$) δ ppm: 170.37, 149.63(C4), 139.64(C11), 132.30(C12), 112.73(C20), 77.78(C5), 68.74(C13), 40.16(C8), 39.94(C1), 39.59(C9), 39.44(C15), 38.09(C3), 37.14(C14), 33.49(C7), 31.67(C17), 28.20(C6), 27.98(C2), 25.49(C10 and C16), 22.48(C19), 21.53(C22), 15.96(C18); HRMS (ESI) *m/z* calculated for $C_{22}H_{33}O^{18}O$ [M-H_2O+H]$^+$: 331.2518, found: 331.2507.

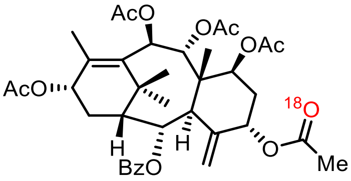

^{18}O labeled 2*α*-benzoyloxy-7*β*-acetoxytaxusin (25)

The ^{18}O labeled compound **25** was prepared as the same protocol for the synthesis of compound **15** by using ^{18}O labeled 5*α*-acetoxytaxa-4(20),11(12)-diene-13*α*-ol (**23**) as starting material. About 0.7 mg purified **25** was dissolved in $CDCl_3$ and subjected to NMR analysis.

^{1}H NMR (600 MHz, $CDCl_3$) δ ppm: 7.99-7.97 (m, 2H), 7.58-7.56 (m, 1H), 7.45-7.43 (m, 2H), 6.22 (d, *J* = 10.6 Hz, 1H, 10*α*-H), 6.02-5.99 (m, 2H, 9*β*-H and 13*β*-H), 5.80 (dd, *J* = 7.0, 2.2 Hz, 1H, 2*β*-H), 5.42 (dd, *J* = 11.4, 5.4 Hz, 1H, 7*α*-H), 5.37 (s, 1H, 20-H), 5.31 (t, *J* = 4.6 Hz, 1H, 5*β*-H), 4.83 (s, 1H, 20-H), 3.35 (d, *J* = 6.8 Hz, 1H, 3*α*-H), 2.56(dt, *J* = 14.8, 9.4 Hz, 1H, 14*β*-H), 2.28 (s, 3H), 2.24 (s, 3H, 18-H), 2.14-2.09 (m, 1H, 1*β*-H), 2.14 (s, 3H), 2.06 (s, 3H), 2.05 (s, 3H), 2.01 (s, 3H), 2.02-1.96 (m, 1H, 6*α*-H), 1.86 (s, 3H, 16-H), 1.80-1.76 (m, 1H, 6*β*-H), 1.63-1.59 (m, 1H, 14*α*-H), 1.19(s, 3H, 17-H), 1.06 (s, 3H, 19-H); ^{13}C NMR (150 MHz, $CDCl_3$) δ ppm: 170.62, 170.03, 169.98, 169.96, 169.41, 165.05, 139.79(C4), 137.60(C12), 133.47, 133.22(C11), 130.04 (two carbons), 128.68, 120.08(C20), 76.36(C5), 76.11(C9), 71.66(C10), 71.38(C2), 70.57(C13), 70.11(C7), 49.25(C1), 47.48(C8), 43.41(C3), 37.84(C15), 35.23(C6), 31.79(C17), 28.15(C14), 27.63(C16), 21.99, 21.66, 21.50, 21.13, 20.94, 15.45(C18), 13.98(C19); HRMS (ESI) *m/z* calculated for $C_{37}H_{50}NO_{11}{}^{18}O$ $[M+NH_4]^+$: 702.3370, found: 702.3361.

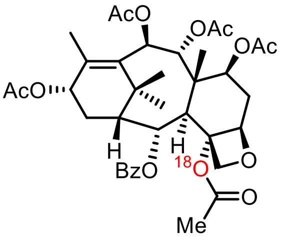

^{18}O labeled 1β-dehydroxybaccatin VI (26a)

The ^{18}O labeled compound **26a** was prepared as the same protocol for the synthesis of compound **15** by using ^{18}O labeled 5*α*-acetoxytaxa-4(20),11(12)-diene-13*α*-ol (**23**) as starting material. About 0.8 mg purified **26a** was dissolved in $CDCl_3$ and subjected to NMR analysis.

^{1}H NMR (400 MHz, $CDCl_3$) δ ppm: 8.09-8.06 (m, 2H), 7.62-7.58 (m, 1H), 7.50-7.44

(m, 2H), 6.19 (d, J = 11.2 Hz, 1H, 10α-H), 6.00 (d, J = 11.2 Hz, 1H, 9β-H), 5.96-5.92 (m, 1H, 13β-H), 5.86 (dd, J = 6.0, 2.4 Hz, 1H, 2β-H), 5.58-5.54 (m, 1H, 7α-H), 5.00 (d, J = 9.0 Hz, 1H, 5α-H), 4.38 (d, J = 8.2 Hz, 1H, 20α-H), 4.13 (d, J = 8.2 Hz, 1H, 20β-H), 3.01 (d, J = 5.6 Hz, 1H, 3α-H), 2.55-2.49 (m, 1H, 6α-H), 2.50-2.40(m, 1H, 14β-H), 2.28 (s, 3H), 2.19 (s, 3H), 2.11 (s, 3H), 2.09 (s, 3H), 2.04 (s, 3H), 1.98(s, 3H), 1.92-1.86 (m, 1H, 6β-H), 1.86 (s, 3H), 1.68 (dd, J = 15.6, 7.2 Hz, 1H, 14α-H), 1.59 (s, 3H), 1.14 (s, 3H); HRMS (ESI) *m/z* calculated for $C_{37}H_{50}NO_{12}{}^{18}O$ [M+NH$_4$]$^+$: 718.3319, found: 718.3294.

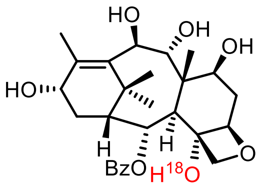

^{18}O labeled 4,7,9,10,13-pentadeacetyl-1β-dehydroxy-baccatin VI (28a)

The deactylation reaction was done as literature reported procedure[13]. To the solution of ^{18}O labeled 1β-dehydroxybaccatin VI **(26a)** (2.4 mg, 3.4 μmol) in ethanol (200 μL), hydrazine hydrate (200 μL) was added. The resulted solution was stirred at room temperature over 32 hours and then diluted with water (2 mL), neutralized with 1M HCl aqueous solution. The resulted aqueous solution was extracted with ethyl acetate (3 × 10 mL). The combined organic phase was washed with brine and dried with sodium sulfate, filtered and purified by prep-HPLC to obtain the desired ^{18}O labeled 4,7,9,10,13-pentadeacetyl-1β-dehydroxybaccatinVI **(28a)** (0.8 mg, 48% yield). About 0.8 mg synthesized **28a** was dissolved in D$_6$-DMSO and subjected to NMR analysis.

^{1}H NMR (500 MHz, D$_6$-DMSO) δ ppm: 8.05-7.98 (m, 2H), 7.69-7.64 (m, 1H), 7.56-7.50 (m, 2H), 6.10-6.02 (m, 2H, -OH), 5.84 (brs, 1H, -OH), 5.62-5.58 (m, 1H, 2β-H), 5.01-4.94 (m, 1H, -OH), 4.68-4.60 (m, 1H, 5α-H, 10β-H and –OH), 4.22-4.16 (m, 1H, 13β-H), 4.15 (dd, J = 6.4 Hz, 1H, 20-H), 4.05 (dd, J = 6.4 Hz, 1H, 20-H), 3.98-3.90 (m, 2H, 9β-H and 7α-H), 2.62-2.60 (m, 1H, 3α-H), 2.47-2.42 (m, 1H, 14β-H), 2.31-2.23 (m, 2H, 6α-H and 14α-H), 1.86 (s, 3H, 18-H), 1.70-1.64 (m, 1H, 6β-H), 1.62-1.58 (m, 1H, 1β-H), 1.56 (s, 3H, 16-H), 1.43 (s, 3H, 19-H), 0.93 (s, 3H, 17-H); ^{13}C NMR (125 MHz, D$_6$-DMSO) δ ppm: 164.61, 138.22(C11), 136.98(C12), 133.57, 129.73, 129.52, 128.76, 87.91(C5), 79.04(C20), 78.53(C9), 74.52 (C4), 72.52(C7), 70.73(C10), 70.61(C2), 66.06(C13), 46.37(C1), 46.21(C3), 43.18(C8), 37.24(C6), 36.94(C15), 33.91(C17), 29.45(C14), 25.34(C16), 16.92(C18), 12.27(C19); HRMS (ESI) *m/z* calculated for $C_{27}H_{40}NO_7{}^{18}O$ [M+NH$_4$]$^+$: 508.2791, found: 508.2778.

Supplementary references

1 Jennewein, S., Long, R. M., Williams, R. M. & Croteau, R. Cytochrome p450 taxadiene 5alpha-hydroxylase, a mechanistically unusual monooxygenase catalyzing the first oxygenation step of Taxol biosynthesis. *Chem. Biol.* **11**, 379-387 (2004).

2 Jennewein, S., Rithner, C. D., Williams, R. M. & Croteau, R. B. Taxol biosynthesis: taxane 13 alpha-hydroxylase is a cytochrome P450-dependent monooxygenase. *Proc. Natl. Acad. Sci. USA* **98**, 13595-13600 (2001).

3 Schoendorf, A., Rithner, C. D., Williams, R. M. & Croteau, R. B. Molecular cloning of a cytochrome P450 taxane 10 beta-hydroxylase cDNA from taxus and functional expression in yeast. *Proc. Natl. Acad. Sci. USA* **98**, 1501-1506 (2001).

4 Jennewein, S., Wildung, M. R., Chau, M., Walker, K. & Croteau, R. Random sequencing of an induced Taxus cell cDNA library for identification of clones involved in Taxol biosynthesis. *Proc. Natl. Acad. Sci. USA* **101**, 9149-9154 (2004).

5 Chau, M., Jennewein, S., Walker, K. & Croteau, R. Taxol biosynthesis: Molecular cloning and characterization of a cytochrome P450 taxoid 7 beta-hydroxylase. *Chem. Biol.* **11**, 663-672 (2004).

6 Chau, M. & Croteau, R. Molecular cloning and characterization of a cytochrome P450 taxoid 2alpha-hydroxylase involved in Taxol biosynthesis. *Arch. Biochem. Biophys.* **427**, 48-57 (2004).

7 Sanchez-Munoz, R. *et al.* A novel hydroxylation step in the taxane biosynthetic pathway: A new approach to paclitaxel production by synthetic biology. *Front Bioeng. Biotechnol.* **8**, 410 (2020).

8 Huang, Q., Pennington, J. D., Williams, H. J. & Scott, A. I. Models for Taxol biosynthesis: SeO_2 oxidation of taxadiene. *Synth. Commun.* **36**, 2577-2585 (2005).

9 Wilde, N. C., Isomura, M., Mendoza, A. & Baran P. S. Two-phase synthesis of (−)-taxuyunnanine D. *J. the Am. Chem. Soc.* **136**, 4909-4912 (2014).

10 Wheeler, A. L., Long, R. M., Ketchum, R. E. B., Rithner, C. D., Williams, R. M. & Croteau, R. Taxol biosynthesis: Differential transformations of taxadien-5a-ol and its acetate ester by cytochrome P450 hydroxylases from taxus suspension cells. *Arch. Biochem. Biophys.* **390**, 265-278 (2001).

11 Hara, R., Furukawa, T., Horiguchi, Y. & Kuwajima, I. Total synthesis of (±)-taxusin. *J. Am. Chem. Soc.* **118**, 9186-9187 (1996).

12 Min, A. D., Jiang, H. & Liang, J. Y. Studies on the taxane diterpenes of the heart-wood from *Taxus mairei*. *Acta Pharm. Sin.* **24**, 673-677 (1989).

13 Lin, H.-X., Jiang, Y., Chen, J.-M., Chen, J.-K. & Chen, M.-Q. Synthesis and crystal structure of 2-debenzoyl and 4-deacetyl 1-deoxybaccatin VI derivatives. *J. Mol. Struct.* **738**, 59–65 (2005).
