# Supplementary material for: Biosynthesis of the highly oxygenated tetracyclic core skeleton of Taxol
Source: Nat Commun. 2024 Mar 15;15:2339. doi: 10.1038/s41467-024-46583-3 (PMC10942993; doi:10.1038/s41467-024-46583-3)
Supplement: Supplementary file 3 — Description of Additional Supplementary Files [file 41467_2024_46583_MOESM3_ESM.pdf]

Description of Additional Supplementary Files

Supplementary Data 1: Summary of activities of characterized acyltransferases towards different taxoid compounds.

Supplementary Data 2: Primers used in this study.

Supplementary Data 3: Public datasets used in this study.

Supplementary Data 4: Nucleotide sequences for genes used in this study.
