# Supplementary material for: Biosynthesis of the highly oxygenated tetracyclic core skeleton of Taxol
Source: Nat Commun. 2024 Mar 15;15:2339. doi: 10.1038/s41467-024-46583-3 (PMC10942993; doi:10.1038/s41467-024-46583-3)
Supplement: Supplementary file 8 — Report Summary [file 41467_2024_46583_MOESM8_ESM.pdf]

Corresponding author(s): Zhihua Zhou

Last updated by author(s): Mar 2, 2024

Reporting Summary

Nature Portfolio wishes to improve the reproducibility of the work that we publish. This form provides structure for consistency and transparency in reporting. For further information on Nature Portfolio policies, see our Editorial Policies and the Editorial Policy Checklist.

Statistics

For all statistical analyses, confirm that the following items are present in the figure legend, table legend, main text, or Methods section.

n/a	Confirmed	
☐	☒	The exact sample size (*n*) for each experimental group/condition, given as a discrete number and unit of measurement
☐	☒	A statement on whether measurements were taken from distinct samples or whether the same sample was measured repeatedly
☒	☐	The statistical test(s) used AND whether they are one- or two-sided<br>*Only common tests should be described solely by name; describe more complex techniques in the Methods section.*
☒	☐	A description of all covariates tested
☒	☐	A description of any assumptions or corrections, such as tests of normality and adjustment for multiple comparisons
☐	☒	A full description of the statistical parameters including central tendency (e.g. means) or other basic estimates (e.g. regression coefficient) AND variation (e.g. standard deviation) or associated estimates of uncertainty (e.g. confidence intervals)
☒	☐	For null hypothesis testing, the test statistic (e.g. *F*, *t*, *r*) with confidence intervals, effect sizes, degrees of freedom and *P* value noted<br>*Give P values as exact values whenever suitable.*
☒	☐	For Bayesian analysis, information on the choice of priors and Markov chain Monte Carlo settings
☒	☐	For hierarchical and complex designs, identification of the appropriate level for tests and full reporting of outcomes
☒	☐	Estimates of effect sizes (e.g. Cohen's *d*, Pearson's *r*), indicating how they were calculated

Our web collection on statistics for biologists contains articles on many of the points above.

Software and code

Policy information about availability of computer code

Data collection	GC-FID data was collected from a GC2010 pro equipped with a flame ionization detector (FID), HPLC data was collected from a Shimadzu LC20A system; LC-MS data was obtain from Dionex Ultimate 3000 RSLC (HPG) ultra-performance liquid chromatography system (Thermo Fisher Scientific) with Q Exactive quadrupole orbitrap high resolution mass spectrometry with a HESI ionization source ; Bruker AM–500 (500 MHz) or Bruker AV Neo 500-, 600-MHz spectrometers were employed for NMR data acquisition.
Data analysis	Trimmomatic (version 0.38), Trinity (version 2.9.0), sratoolkit (version 2.10.5), Newbler (version 3.0, Roche), CD-HIT-EST (version 4.8.1), Diamond(version 0.9.36), TransDecoder (version 5.0.5) , SMRTlink (version 8.0.0) , IsoSeq3 low-level workflow pipeline (3.3.0, https://github.com/PacificBiosciences/IsoSeq) , and FASTQC (version 0.11.9) were used for pan-transcriptome analysis and RNA-seq data analysis.Gene sequence was analysed on Geneious (Version R8); GC-FID analysis was performed on LabSolution LE (version 5.90) ; HPLC analysis was performed on LabSolution LC-GC (version 5.97); Xcalibur (version 4.2) software was used for LC-MS analysis; ChemDraw (version 15.1) was used to calculate molecular weight and draw chemical structure; NMR data was processed and visualized on MestRenova (version 14.1.0); Figures were processed using Adobe illustrator 2023.

For manuscripts utilizing custom algorithms or software that are central to the research but not yet described in published literature, software must be made available to editors and reviewers. We strongly encourage code deposition in a community repository (e.g. GitHub). See the Nature Portfolio guidelines for submitting code & software for further information.

Data

Policy information about availability of data

All manuscripts must include a data availability statement. This statement should provide the following information, where applicable:

- Accession codes, unique identifiers, or web links for publicly available datasets
- A description of any restrictions on data availability
- For clinical datasets or third party data, please ensure that the statement adheres to our policy

RNA sequencing data generated in this study have been deposited at NCBI under BioProject no. PRJNA1062083 (https://www.ncbi.nlm.nih.gov/bioproject/?term=PRJNA1062083). CYP725A37, codon-optimized CYP725A37, CYP725A55 and AT5 have been deposited at Genbank under accession numbers PP197199 (https://www.ncbi.nlm.nih.gov/nuccore/PP197199), PP197200 (https://www.ncbi.nlm.nih.gov/nuccore/PP197200), PP197201 (https://www.ncbi.nlm.nih.gov/nuccore/PP197201) and PP197202 (https://www.ncbi.nlm.nih.gov/nuccore/PP197202), respectively. Sequences of genes used in this study were derived from Genbank under the following accession numbers: GGPPs (D28748, https://www.ncbi.nlm.nih.gov/nuccore/D28748.1), TS (U48796, https://www.ncbi.nlm.nih.gov/nuccore/U48796), T13OH (AY056019, https://www.ncbi.nlm.nih.gov/nuccore/AY056019), T10OH (AF318211, https://www.ncbi.nlm.nih.gov/nuccore/AF318211), T10OH2 (AY563635, https://www.ncbi.nlm.nih.gov/nuccore/AY563635), T7OH (AY307951, https://www.ncbi.nlm.nih.gov/nuccore/AY307951), T2OH (AY518383, https://www.ncbi.nlm.nih.gov/nuccore/AY518383), T5AT (AF190130, https://www.ncbi.nlm.nih.gov/nuccore/AF190130), TAX19 (AY628434, https://www.ncbi.nlm.nih.gov/nuccore/AY628434), TCPR (AY571340, https://www.ncbi.nlm.nih.gov/nuccore/AY571340), AAE4 (MN961507, https://www.ncbi.nlm.nih.gov/nuccore/MN961507), and TBT (AF297618, https://www.ncbi.nlm.nih.gov/nuccore/AF297618). All reported data in this study are available. Source data are provided with this paper. All the NMR spectra (Supplementary Fig.13 to 117) and data are available in Supplementary Information.

Research involving human participants, their data, or biological material

Policy information about studies with human participants or human data. See also policy information about sex, gender (identity/presentation), and sexual orientation and race, ethnicity and racism.

Reporting on sex and gender	not applicable
Reporting on race, ethnicity, or other socially relevant groupings	not applicable
Population characteristics	not applicable
Recruitment	not applicable
Ethics oversight	not applicable

Note that full information on the approval of the study protocol must also be provided in the manuscript.

Field-specific reporting

Please select the one below that is the best fit for your research. If you are not sure, read the appropriate sections before making your selection.

☒ Life sciences ☐ Behavioural & social sciences ☐ Ecological, evolutionary & environmental sciences

For a reference copy of the document with all sections, see nature.com/documents/nr-reporting-summary-flat.pdf

Life sciences study design

All studies must disclose on these points even when the disclosure is negative.

Sample size	All experiments in this study have a sample size of three replicates from different clones. The experiments for analysis of intermediates (Supplementary Fig. S11d) has a sample size of nine to reduce the effect of different clones.
Data exclusions	No data were excluded from the analysis.
Replication	All in vivo and in vitro experiments were performed more than three times with similar results.
Randomization	Randomization is not relevant to the experiments in this study because experimental conditions were precisely set to demonstrate the function of different enzymes and the grouping of samples was not random.
Blinding	Blinding was not relevant for this study. Functional characterization of enzymes needed the understanding of researchers about the tested samples and required researchers to know the tested samples contained which enzymes and substrates. So, blinding was not relevant to our studies.

Reporting for specific materials, systems and methods

We require information from authors about some types of materials, experimental systems and methods used in many studies. Here, indicate whether each material, system or method listed is relevant to your study. If you are not sure if a list item applies to your research, read the appropriate section before selecting a response.

Materials & experimental systems

n/a	Involved in the study	
☒	☐	Antibodies
☒	☐	Eukaryotic cell lines
☒	☐	Palaeontology and archaeology
☒	☐	Animals and other organisms
☒	☐	Clinical data
☒	☐	Dual use research of concern
☐	☒	Plants

Methods

n/a	Involved in the study	
☒	☐	ChIP-seq
☒	☐	Flow cytometry
☒	☐	MRI-based neuroimaging

Plants

Seed stocks	not applicable
Novel plant genotypes	not applicable
Authentication	not applicable
